# Comparing researchers’ degree of dichotomous thinking using frequentist versus Bayesian null hypothesis testing

**DOI:** 10.1038/s41598-024-62043-w

**Published:** 2024-05-27

**Authors:** Jasmine Muradchanian, Rink Hoekstra, Henk Kiers, Dustin Fife, Don van Ravenzwaaij

**Affiliations:** 1 Behavioural and Social Sciences, University of Groningen, Groningen, The Netherlands; 2 Psychology, Rowan University, Glassboro, USA

**Keywords:** Human behaviour, Neuroscience

## Abstract

**Abstract:**

A large amount of scientific literature in social and behavioural sciences bases their conclusions on one or more hypothesis tests. As such, it is important to obtain more knowledge about how researchers in social and behavioural sciences interpret quantities that result from hypothesis test metrics, such as *p*-values and Bayes factors. In the present study, we explored the relationship between obtained statistical evidence and the degree of belief or confidence that there is a positive effect in the population of interest. In particular, we were interested in the existence of a so-called cliff effect: A qualitative drop in the degree of belief that there is a positive effect around certain threshold values of statistical evidence (e.g., at *p* = 0.05). We compared this relationship for *p*-values to the relationship for corresponding degrees of evidence quantified through Bayes factors, and we examined whether this relationship was affected by two different modes of presentation (in one mode the functional form of the relationship across values was implicit to the participant, whereas in the other mode it was explicit). We found evidence for a higher proportion of cliff effects in *p*-value conditions than in BF conditions (N = 139), but we did not get a clear indication whether presentation mode had an effect on the proportion of cliff effects.

**Protocol registration:**

The stage 1 protocol for this Registered Report was accepted in principle on 2 June 2023. The protocol, as accepted by the journal, can be found at: https://doi.org/10.17605/OSF.IO/5CW6P.

## Introduction

In applied science, researchers typically conduct statistical tests to learn whether an effect of interest differs from zero. Such tests typically tend to quantify evidence by means of *p*-values (but see e.g., Lakens^1^ who warns against such an interpretation of *p*-values). A Bayesian alternative to the *p*-value is the Bayes factor (BF), which is a tool used for quantifying statistical evidence in hypothesis testing^2,3^. *P*-values and BFs are related to one another^4^, with BFs being used much less frequently. Having two contrasting hypotheses (i.e., a null hypothesis, H_0_, and an alternative hypothesis, H_1_), a *p*-value is the probability of getting a result as extreme or more extreme than the actual observed sample result, given that H_0_ were true (and given that the assumptions hold). A BF on the other hand, quantifies the probability of the data given H_1_ relative to the probability of the data given H_0_ (called BF_10_^3^).

There is ample evidence that researchers often find it difficult to interpret quantities such as *p*-values^5–7^. Although there has been growing awareness of the dangers of misinterpreting *p*-values, these dangers seem to remain prevalent. One of the key reasons for these misinterpretations is that these concepts are not simple or intuitive, and the correct interpretation of them would require more cognitive effort. Because of this high cognitive demand academics have been using shortcut interpretations, which are simply wrong^6^. An example of such a misinterpretation is that the *p*-value would represent the probability of the null hypothesis being true^6^. Research is typically conducted in order to reduce uncertainty around the existence of an effect in the population of interest. To do this, we use measures such as *p*-values and Bayes factors as a tool. Hence, it might be interesting (especially given the mistakes that are made by researchers when interpreting quantities such as *p*-values) to study how these measures affect people’s beliefs regarding the existence of an effect in the population of interest, so one can study how outcomes like *p*-values and Bayes factors translate to subjective beliefs about the existence of an effect in practice.

One of the first studies that focused on how researchers interpret statistical quantities was conducted by Rosenthal and Gaito^8^, in which they specifically studied how researchers interpret *p*-values of varying magnitude. Nineteen researchers and graduate students at their psychology faculty were requested to indicate their degree of belief or confidence in 14 *p*-values, varying from 0.001 to 0.90, on a 6-point scale ranging from “5 extreme confidence or belief” to “0 complete absence of confidence or belief”^8^^, pp. 33–34^. These individuals were shown *p*-values for sample sizes of 10 and 100. The authors wanted to measure the degree of belief or confidence in research findings as a function of associated *p*-values, but stated as such it is not really clear what is meant here. We assume that the authors actually wanted to assess degree of belief or confidence in the existence of an effect, given the *p*-value. Their findings suggested that subjects’ degree of belief or confidence appeared to be a decreasing exponential function of the *p-*value. Additionally, for any *p*-value, self-rated confidence was greater for the larger sample size (i.e., *n* = 100). Furthermore, the authors argued in favor of the existence of a *cliff* effect around *p* = 0.05, which refers to an abrupt drop in the degree of belief or confidence in a *p*-value just beyond the 0.05 level^8,9^. This finding has been confirmed in several subsequent studies^10–12^. The studies described so far have been focusing on the average, and have not taken individual differences into account.

The cliff effect suggests *p*-values invite dichotomous thinking, which according to some authors seems to be a common type of reasoning when interpreting *p*-values in the context of Null Hypothesis Significance Testing (NHST^13^). The outcome of the significance test seems to be usually interpreted dichotomously such as suggested by studies focusing on the cliff effect^8–13^, where one makes a binary choice between rejecting or not rejecting a null hypothesis^14^. This practice has taken some academics away from the main task of finding out the size of the effect of interest and the level of precision with which it has been measured^5^. However, Poitevineau and Lecoutre^15^ argued that the cliff effect around *p* = 0.05 is probably overstated. According to them, previous studies paid insufficient attention to individual differences. To demonstrate this, they explored the individual data and found qualitative heterogeneity in the respondents’ answers. The authors identified three categories of functions based on 12 *p*-values: (1) a decreasing exponential curve, (2) a decreasing linear curve, and (3) an all-or-none curve representing a very high degree of confidence when *p* ≤ 0.05 and quasi-zero confidence otherwise. Out of 18 participants, they found that the responses of 10 participants followed a decreasing exponential curve, 4 participants followed a decreasing linear curve, and 4 participants followed an all-or-none curve. The authors concluded that the cliff effect may be an artifact of averaging, resulting from the fact that a few participants have an all-or-none interpretation of statistical significance^15^.

Although NHST has been used frequently, it has been argued that it should be replaced by effect sizes, confidence intervals (CIs), and meta-analyses. Doing so may allegedly invite a shift from dichotomous thinking to estimation and meta-analytic thinking^14^. Lai et al.^13^ studied whether using CIs rather than *p*-values would reduce the cliff effect, and thereby dichotomous thinking. Similar to the classification by Poitevineau and Lecoutre^15^, the responses were divided into three classes: decreasing exponential, decreasing linear, or all-or-none. In addition, Lai et al.^13^ found patterns in the responses of some of the participants that corresponded with what they called a “moderate cliff model”, which refers to using statistical significance as both a decision-making criterion and a measure of evidence^13^.

In contrast to Poitevineau and Lecoutre^15^, Lai et al.^13^ concluded that the cliff effect is probably not just a byproduct resulting from the all-or-none class, because the cliff models were accountable for around 21% of the responses in NHST interpretation and for around 33% of the responses in CI interpretation. Furthermore, a notable finding was that the cliff effect prevalence in CI interpretations was more than 50% higher than that of NHST^13^. Something similar was found in a study by Hoekstra, Johnson, and Kiers^16^. They also predicted that the cliff effect would be stronger for results presented in the NHST format compared to the CI format, and like Lai et al.^13^, they actually found more evidence of a cliff effect in the *CI format* compared to the *NHST format*^16^.

The studies discussed so far seem to provide evidence for the existence of a cliff effect around *p* = 0.05. Table 1 shows an overview of evidence related to the cliff effect. Interestingly, in a recent study, Helske et al.^17^ examined how various visualizations can aim in reducing the cliff effect when interpreting inferential statistics among researchers. They found that compared to textual representation of the CI with *p*-values and classic CI visualization, including more complex visual information to classic CI representation seemed to decrease the cliff effect (i.e., dichotomous interpretations^17^).

**Table 1:** Overview of cliff effect studies.

Authors	Year	N	Analyzed method	Studied sample size	Number of p-values	Results: average or individual	Cliff
Rosenthal and Gaito	1963	19	*p*-values	10, 100	14	Average	Yes, around *p* = 0.05
Beauchamp and May	1964	20	*p*-values	10, 100	12	Average	Yes, around *p* = 0.05
Minturn, Lansky, and Dember	1972	51	*p*-values	20, 200	12	Average	Yes, around *p* = 0.01, 0.05, and 0.10
Nelson, Rosenthal, and Rosnow	1986	85	*p*-values	10, 100	20	Average	Yes, around *p* = 0.05 and 0.10
Poitevineau and Lecoutre	2001	18	*p*-values	10, 100	12	Average and individual	Yes, around *p* = 0.05, but only For some participants
Lai, Kalinowski, Fidler, and Cumming	2010	172	*p-*values, CIs	15, 50	8	Individual	Yes, around *p* = 0.05 for both *p*-values and CIs
Hoekstra, Johnson, and Kiers	2012	65	*p-*values, CIs	250	4	Average	Yes, around *p* = 0.05 for both *p*-values and CIs

Beauchamp and May^10^ suggested that they did not find statistically significant cliff effects. Although statistically non-significant, Rosenthal and Gaito^9^ suggested that Beauchamp and May’s data were consistent with cliff characteristics around *p* = 0.05.

Although Bayesian methods have become more popular within different scientific fields^18,19^, we know of no studies that have examined whether self-reported degree of belief of the existence of an effect when interpreting BFs by researchers results in a similar cliff effect to those obtained for *p*-values and CIs. Another matter that seems to be conspicuously absent in previous examinations of the cliff effect is a comparison between the presentation methods that are used to investigate the cliff effect. In some cliff effect studies the *p*-values were presented to the participants on separate pages^15^ and in other cliff effect studies the *p*-values were presented on the same page^13^. It is possible that the cliff effect manifests itself in (some) researchers without explicit awareness. It is possible that for those researchers presenting *p*-values/Bayes factors in isolation would lead to a cliff effect, whereas presenting all *p*-values/Bayes factors at once would lead to a cognitive override. Perhaps when participants see their cliff effect, they might think that they should not think dichotomously, and might change their results to be more in line with how they believe they should think, thereby removing their cliff effect. To our knowledge, no direct comparison of *p*-values/Bayes factors in isolation and all *p*-values/Bayes factors at once has yet been conducted. Therefore, to see whether the method matters, both types of presentation modes will be included in the present study.

All of this gives rise to the following three research questions: (1) What is the relation between obtained statistical evidence and the degree of belief or confidence that there is a positive effect in the population of interest across participants? (2) What is the difference in this relationship when the statistical evidence is quantified through *p*-values versus Bayes factors? (3) What is the difference in this relationship when the statistical evidence is presented in isolation versus all at once?

In the present study, we will investigate the relationship between method (i.e., *p*-values and Bayes factors) and the degree of belief or confidence that there is a positive effect in the population of interest, with special attention for the cliff effect. We choose this specific wording (“positive effect in the population of interest”) as we believe that this way of phrasing is more specific than those used in previous cliff effect studies. We will examine the relationship between different levels of strength of evidence using *p*-values or corresponding Bayes factors and measure participants' degree of belief or confidence in the following two scenarios: (1) the scenario in which values will be presented in isolation (such that the functional form of the relationship across values is implicit to the participant) and (2) the scenario in which all values will be presented simultaneously (such that the functional form of the relationship across values is explicit to the participant).

In what follows, we will first describe the set-up of the present study. In the results section, we will explore the relationship between obtained statistical evidence and the degree of belief or confidence, and in turn, we will compare this relationship for *p*-values to the corresponding relationship for BFs. All of this will be done in scenarios in which researchers are either made aware or not made aware of the functional form of the relationship. In the discussion, we will discuss implications for applied researchers using *p*-values and/or BFs in order to quantify statistical evidence.

## Method

### Ethics information

Our study protocol has been approved by the ethics committee of the University of Groningen and our study complies with all relevant ethical regulations of the University of Groningen. Informed consent will be obtained from all participants. As an incentive for participating, we will raffle 10 Amazon vouchers with a worth of 25USD among participants that successfully completed our study.

### Sampling plan

Our target population will consist of researchers in the social and behavioural sciences who are at least somewhat familiar with interpreting Bayes factors. We will obtain our prospective sample by collecting the e-mail addresses of (approximately) 2000 corresponding authors from 20 different journals in social and behavioural sciences with the highest impact factor. Specifically, we will collect the e-mail addresses of 100 researchers who published an article in the corresponding journal in 2021. We will start with the first issue and continue until we have 100 e-mail addresses per journal. We will contact the authors by e-mail. In the e-mail we will mention that we are looking for researchers who are familiar with interpreting Bayes factors. If they are familiar with interpreting Bayes factors, then we will ask them to participate in our study. If they are not familiar with interpreting Bayes factors, then we will ask them to ignore our e-mail.

If the currently unknown response rate is too low to answer our research questions, we will collect additional e-mail addresses of corresponding authors from articles published in 2022 in the same 20 journals. Based on a projected response rate of 10%, we expect a final completion rate of 200 participants. This should be enough to obtain a BF higher than 10 in favor of an effect if the proportions differ by 0.2 (see section “Planned analyses” for details).

### Materials and procedure

The relationship between the different magnitudes of *p*-values/BFs and the degree of belief or confidence will be examined in a scenario in which values will be presented in isolation and in a scenario in which the values will be presented simultaneously. This all will result in four different conditions: (1) *p*-value questions in the isolation scenario (isolated *p*-value), (2) BF questions in the isolation scenario (isolated BF), (3) *p*-value questions in the simultaneous scenario (all at once *p*-value), and (4) BF questions in the simultaneous scenario (all at once BF). To reduce boredom, and to try to avoid making underlying goals of the study too apparent, each participant will receive randomly one out of four scenarios (i.e., all at once *p*-value, all at once BF, isolated *p*-value, or isolated BF), so the study has a between-person design.

The participants will receive an e-mail with an anonymous Qualtrics survey link. The first page of the survey will consist of the informed consent. We will ask all participants to indicate their level of familiarity with both Bayes factors and *p*-values on a 3-point scale with “completely unfamiliar/somewhat familiar/very familiar” and we will include everyone who is at least somewhat familiar on both. To have a better picture of our sample population, we will include the following demographic variables in the survey: gender, main continent, career stage, and broad research area. Then we will randomly assign respondents to one of four conditions (see below for a detailed description). After completing the content-part of the survey, all respondents will receive a question about providing their e-mail address if they are interested in (1) being included in the random draw of the Amazon vouchers; or (2) receiving information on our study outcomes.

In the isolated *p*-value condition, the following fabricated experimental scenario will be presented:

“Suppose you conduct an experiment comparing two independent groups, with n = 250 in each group. The null hypothesis states that the population means of the two groups do not differ. The alternative hypothesis states that the population mean in group 1 is larger than the population mean in group 2. Suppose a two-sample t test was conducted and a one-sided p value calculated.”

Then a set of possible findings of the fabricated experiment will be presented at different pages. We varied the strength of evidence for the existence of a positive effect with the following ten *p*-values in a random order: 0.001, 0.002, 0.004, 0.008, 0.016, 0.032, 0.065, 0.131, 0.267, and 0.543. A screenshot of a part of the isolated *p*-value questions is presented in S1 in the Supplementary Information.

In the all at once BF condition, a fabricated experimental scenario will be presented identical to that in the isolated *p*-value condition, except the last part is replaced by:

“Suppose a Bayesian two-sample t test was conducted and a one-sided Bayes factor (BF) calculated, with the alternative hypothesis in the numerator and the null hypothesis in the denominator, denoted BF_10_.”

A set of possible findings of the fabricated experiment will be presented at the same page. These findings vary in terms of the strength of evidence for the existence of a positive effect, quantified with the following ten BF_10_ values in the following order: 22.650, 12.008, 6.410, 3.449, 1.873, 1.027, 0.569, 0.317, 0.175, and 0.091. These BF values correspond one-on-one to the *p*-values presented in the isolated *p*-value condition (the R code for the findings of the fabricated experiment can be found on https://osf.io/sq3fp). A screenshot of a part of the all at once BF questions can be found in S2 in the Supplementary Information.

In both conditions, the respondents will be asked to rate their degree of belief or confidence that there is a positive effect in the population of interest based on these findings on a scale ranging from 0 (completely convinced that there is no effect), through 50 (somewhat convinced that there is a positive effect), to 100 (completely convinced that there is a positive effect).

The other two conditions (i.e., isolated BF condition and the all at once *p*-value condition) will be the same as the previously described conditions. The only difference between these two conditions and the previously described conditions is that in the isolated BF condition, the findings of the fabricated experiment for the BF questions will be presented at different pages in a random order, and in the all at once *p*-value condition, the findings for the *p*-value questions will be presented at the same page in a non-random order.

To keep things as simple as possible for the participants, all fictitious scenarios will include a two-sample *t* test with either a one-tailed *p*-value or a BF. The total sample size will be large (*n* = 250 in each group) in order to have sufficiently large power to detect even small effects.

### Planned analyses

**Figure 1:**
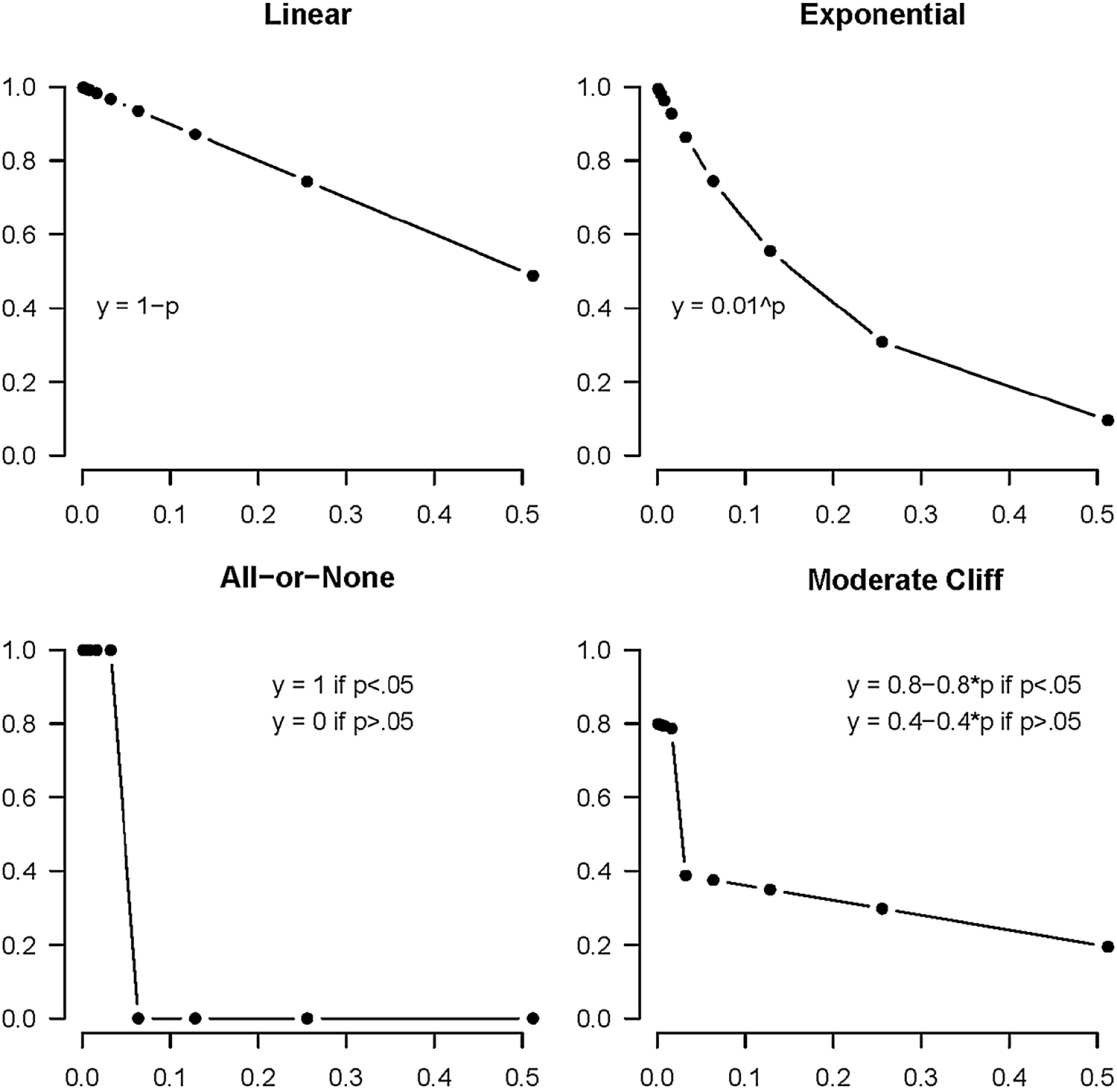
Plots are shown for fictitious outcomes for the four models (all-or-none, linear, exponential, and moderate cliff). The x-axis represents the different *p*-values. In the two BF conditions, the x-axis represents the different BF values. The y-axis represents the proportion of degree of belief or confidence that there is a positive effect in the population of interest. Note that these are prototype responses; different variations on these response patterns are possible.

Poitevineau and Lecoutre^15^ have suggested the following three models for the relationships between the different levels of statistical evidence and researchers’ subjective belief that a non-zero effect exists: all-or-none (*y* = *a* for *p* < 0.05, *y* = *b* for *p* ≥ 0.05), linear (*y* = *a* + *bp*), and exponential (*y* = exp(*a* + *bp*)). In addition, Lai et al.^13^ have suggested the moderate cliff model (a more gradual version of all-or-none), which they did not define more specifically. In the study by Lai et al.^13^ (Fig. 4), the panel that represents the moderate cliff seems to be a combination of the exponential and the all-or-none function. In the present study, we will classify responses as moderate cliff if we observe a steep drop in the degree of belief or confidence around a certain *p*-value/BF, while for the remaining *p*-values/BFs the decline in confidence is more gradual. So, for example, a combination of the decreasing linear and the all-or-none function will also be classified as moderate cliff in the present study. Plots of the four models with examples of reasonable choices for the parameters are presented in Fig. 1 (the R code for Fig. 1 can be found on https://osf.io/j6d8c).

We will manually classify data for each participant for each scenario as one of the relationship models. We will do so by blinding the coders as to the conditions associated with the data. Specifically, author JM will organize the data from each of the four conditions and remove the *p*-value or BF labels. Subsequently, authors DvR and RH will classify the data independently from one another. In order to improve objectivity regarding the classification, authors DvR and RH will classify the data according to specific instructions that are constructed before collecting the data (see Appendix 1). After coding, we will compute Cohen’s kappa for these data. For each set of scores per condition per subject for which there was no agreement on classification, authors DvR and RH will try to reach consensus in a discussion of no longer than 5 min. If after this discussion no agreement is reached, then author DF will classify these data. If author DF will choose the same class as either DvR or RH, then the data will be classified accordingly. However, if author DF will choose another class, then the data will be classified in a so-called rest category. This rest category will also include data that extremely deviate from the four relationship models, and we will assess these data by running exploratory analyses. Before classifying the real data, we will conduct a small pilot study in order to provide authors DvR and RH with the possibility to practice classifying the data. In the Qualtrics survey, the respondents cannot continue with the next question without answering the current question. However, it might be possible that some of the respondents quit filling out the survey. The responses of the participants who did not answer all questions will be removed from the dataset. This means that we will use complete case analysis in order to deal with missing data, because we do not expect to find specific patterns in the missing values.

Our approach to answer Research Question 1 (RQ1; “What is the relation between obtained statistical evidence and the degree of belief or confidence that there is a positive effect in the population of interest across participants?”) will be descriptive in nature. We will explore the results visually, by assessing the four models (i.e., all-or-none, linear, exponential, and moderate cliff) in each of the four conditions (i.e., isolated *p*-value, all at once *p*-value, isolated BF, and all at once BF), followed by zooming in on the classification ‘cliff effect’. This means that we will compare the frequency of the four classification models with one another within each of the four conditions.

In order to answer Research Question 2 (RQ2; “What is the difference in this relationship when the statistical evidence is quantified through *p*-values versus Bayes factors?”), we will first combine categories as follows: the *p*-value condition will encompass the data from both the isolated and the all at once *p*-value conditions, and the BF condition will encompass the data from both the isolated and the all at once BF conditions. Furthermore, the cliff condition will encompass the all-or-none and the moderate cliff models, and the non-cliff condition will encompass the linear and the exponential models. This classification ensures that we distinguish between curves that reflect a sudden change in the relationship between the level of statistical evidence and the degree of confidence that a positive effect exists in the population of interest, and those that represent a gradual relationship between the level of statistical evidence and the degree of confidence. We will then compare the proportions of cases with a cliff in the *p*-value conditions to those in the BF conditions, and we will add inferential information for this comparison by means of a Bayesian chi square test on the 2 × 2 table (*p*-value/BF x cliff/non-cliff), as will be specified below.

Finally, in order to answer Research Question 3 (RQ3; “What is the difference in this relationship when the statistical evidence is presented in isolation versus all at once?”), we will first combine categories again, as follows: the isolation condition will encompass the data from both the isolated *p*-value and the isolated BF conditions, and the all at once condition will encompass the data from both the all at once *p*-value and the all at once BF conditions. The cliff/non-cliff distinction is made analogous to the one employed for RQ2. We will then compare the proportions of cases with a cliff in the isolated conditions to those in the all at once conditions, and we will add inferential information for this comparison by means of a Bayesian chi square test on the 2 × 2 table (all at once/isolated x cliff/non-cliff), as will be specified below.

For both chi square tests, the null hypothesis states that there is no difference in the proportion of cliff classifications between the two conditions, and the alternative hypothesis states that there is a difference in the proportion of cliff classifications between the two conditions. Under the null hypothesis, we specify a single beta(1,1) prior for the proportion of cliff classifications and under the alternative hypothesis we specify two independent beta(1,1) priors for the proportion of cliff classifications^20,21^. A beta(1,1) prior is a flat or uniform prior from 0 to 1. The Bayes factor that will result from both chi square tests gives the relative evidence for the alternative hypothesis over the null hypothesis (BF_10_) provided by the data. Both tests will be carried out in RStudio^22^ (the R code for calculating the Bayes factors can be found on https://osf.io/5xbzt). Additionally, the posterior of the difference in proportions will be provided (the R code for the posterior of the difference in proportions can be found on https://osf.io/3zhju).

If, after having computed results on the obtained sample, we observe that our BFs are not higher than 10 or smaller than 0.1, we will expand our sample in the way explained at the end of section “Sampling Plan”. To see whether this approach will likely lead to useful results, we have conducted a Bayesian power simulation study for the case of population proportions of 0.2 and 0.4 (e.g., 20% cliff effect in the *p*-value group, and 40% cliff effect in the BF group) in order to determine how large the Bayesian power would be for reaching the BF threshold for a sample size of *n* = 200. Our results show that for values 0.2 and 0.4 in both populations respectively, our estimated sample size of 200 participants (a 10% response rate) would lead to reaching a BF threshold 96% of the time, suggesting very high power under this alternative hypothesis. We have also conducted a Bayesian power simulation study for the case of population proportions of 0.3 (i.e., 30% cliff effect in the *p*-value group, and 30% cliff effect in the BF group) in order to determine how long sampling takes for a zero effect. The results show that for values of 0.3 in both populations, our estimated sample size of 200 participants would lead to reaching a BF threshold 7% of the time. Under the more optimistic scenario of a 20% response rate, a sample size of 400 participants would lead to reaching a BF threshold 70% of the time (the R code for the power can be found on https://osf.io/vzdce). It is well known that it is harder to find strong evidence for the absence of an effect than for the presence of an effect^23^. In light of this, we deem a 70% chance of reaching a BF threshold under the null hypothesis given a 20% response rate acceptable. If, after sampling the first 2000 participants and factoring in the response rate, we have not reached either BF threshold, we will continue sampling participants in increments of 200 (10 per journal) until we reach a BF threshold or until we have an effective sample size of 400, or until we reach a total of 4000 participants.

In sum, RQ1 is exploratory in nature, so we will descriptively explore the patterns in our data. For RQ2, we will determine what proportion of applied researchers make a binary distinction regarding the existence of a positive effect in the population of interest, and we will test whether this binary distinction is different when research results are expressed in the *p*-value versus the BF condition. Finally, for RQ3, we will determine whether this binary distinction is different in the isolated versus all at once condition (see Table 2 for a summary of the study design).

**Table 2:** Summary of the study design.

Question	Hypothesis	Participants	Analysis plan	Interpretation given to different outcomes
RQ1: What is the relation between obtained statistical evidence and the degree of belief or confidence that there is a positive effect in the population of interest across participants?	No hypothesis, because this question is exploratory	2000 researchers in the social and behavioural sciences who are familiar with interpreting BFs	The frequency of the four classification models (i.e., all-or-none, linear, exponential, and moderate cliff) will be compared with one another within each of the four conditions (i.e., isolated *p*-value, isolated BF, all at once *p*-value, and all at once BF)	The interpretation will be descriptive in nature, using estimated proportions
RQ2: What is the difference in this relationship when the statistical evidence is quantified through *p*-values versus Bayes factors?	H_0_: there is no difference in the proportion of cliff classifications between the *p*-value and BF condition; H_1_: there is a difference in the proportion of cliff classifications between the *p*-value and BF condition	2000 researchers in the social and behavioural sciences who are familiar with interpreting BFs	A Bayesian chi square test will be carried out. Under the null hypothesis, a single beta(1,1) prior will be specified for the proportion of cliff classifications; under the alternative hypothesis two independent beta(1,1) priors will be specified for the proportion of cliff classifications. Also, the posterior of the difference in proportions will be provided	The BF that will result from the chi square test gives the relative evidence for the alternative hypothesis over the null hypothesis (BF_10_) provided by the data. Credible intervals will be added for further interpretation of the results
RQ3: What is the difference in this relationship when the statistical evidence is presented in isolation versus all at once?	H_0_: there is no difference in the proportion of cliff classifications between the isolation and all at once condition; H_1_: there is a difference in the proportion of cliff classifications between the isolation and all at once condition	2000 researchers in the social and behavioural sciences who are familiar with interpreting BFs	A Bayesian chi square test will be carried out. Under the null hypothesis, a single beta(1,1) prior will be specified for the proportion of cliff classifications; under the alternative hypothesis two independent beta(1,1) priors will be specified for the proportion of cliff classifications. Also, the posterior of the difference in proportions will be provided	The BF that will result from the chi square test gives the relative evidence for the alternative hypothesis over the null hypothesis (BF_10_) provided by the data Credible intervals will be added for further interpretation of the results

### Sampling process

We deviated from our preregistered sampling plan in the following ways: we collected the e-mail address of all corresponding authors who published in the 20 journals in social and behavioural sciences in 2021 and 2022 *at the same time*. In total, we contacted 3152 academics, and 89 of them completed our survey (i.e., 2.8% of the contacted academics). We computed the BFs based on the responses of these 89 academics, and it turned out that the BF for RQ2 was equal to BF_10_ = 16.13 and the BF for RQ3 was equal to BF_10_ = 0.39, so the latter was neither higher than 10 nor smaller than 0.1.

In order to reach at least 4000 potential participants (see “Planned analyses” section), we decided to collect additional e-mail addresses of corresponding authors from articles published in 2019 and 2020 in the same 20 journals. In total, we thus reached another 2247 academics (total N = 5399), and 50 of them completed our survey (i.e., 2.2% of the contacted academics, effective N = 139).

In light of the large number of academics we had contacted at this point, we decided to do an ‘interim power analysis’ to calculate the upper and lower bounds of the BF for RQ3 to see if it made sense to continue collecting data up to N = 200. The already collected data of 21 cliffs out of 63 in the isolated conditions and 13 out of 65 in the all-at-once conditions yields a Bayes factor of 0.8 (see “Results” section below). We analytically verified that by increasing the number of participants to a total of 200, the strongest possible pro-null evidence we can get *given the data we already had* would be BF_10_ = 0.14, or BF_01_ = 6.99 (for 21 cliffs out of 100 in both conditions). In light of this, our judgment was that it was not the best use of human resources to continue collecting data, so we proceeded with a final sample of N = 139.

To summarize our sampling procedure, we contacted 5399 academics in total. Via Qualtrics, 220 participants responded. After removing the responses of the participants who did not complete the content part of our survey (i.e., the questions about the *p*-values or BFs), 181 cases remained. After removing the cases who were completely unfamiliar with *p*-values, 177 cases remained. After removing the cases who were completely unfamiliar with BFs, 139 cases remained. Note that there were also many people who responded via e-mail informing us that they were not familiar with interpreting BFs. Since the Qualtrics survey was anonymous, it was impossible for us to know the overlap between people who contacted us via e-mail and via Qualtrics that they were unfamiliar with interpreting BFs.

## Results

**Figure 2:**
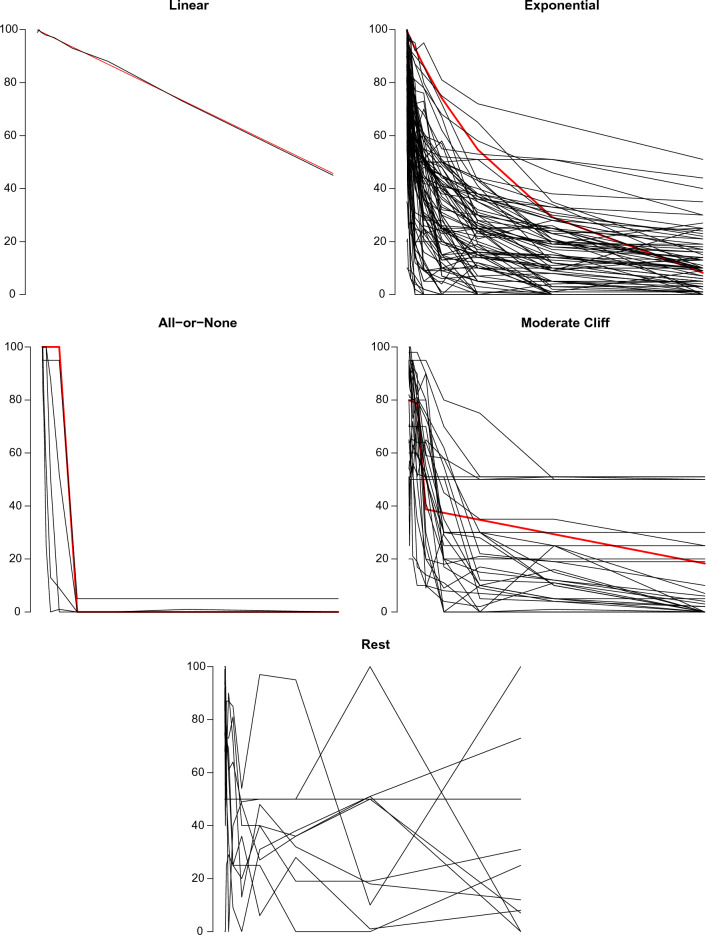
Plots including the prototype lines and the actual responses.

We contacted a total number of 5399 participants. The total number of participants who filled out the survey completely was N = 139, so 2.6% of the total sample (note that this is a result of both response rate and our requirement that researchers needed to self-report familiarity with interpreting BFs). Our entire Qualtrics survey can be found on https://osf.io/6gkcj. Five “difficult to classify” pilot plots were created such that authors RH and DvR could practice before classifying the real data. These plots can be found on https://osf.io/ndaw6/ (see folder “Pilot plots”). Authors RH and DvR had a qualitative discussion about these plots; however, no adjustments were made to the classification protocol. We manually classified data for each participant for each scenario as one of the relationship models (i.e., all-or-none, moderate cliff, linear, and exponential). Author JM organized the data from each of the four conditions and removed the *p*-value or BF labels. Authors RH and DvR classified the data according to the protocol provided in Appendix 1, and the plot for each participant (including the condition each participant was in and the model in which each participant was classified) can be found in Appendix 2. After coding, Cohen’s kappa was determined for these data, which was equal to κ = 0.47. Authors RH and DvR independently reached the same conclusion for 113 out of 139 data sets (i.e., 81.3%). For the remaining 26 data sets, RH and DvR were able to reach consensus within 5 min per data set, as laid out in the protocol. In Fig. 2, plots are provided which include the prototype lines as well as the actual responses plotted along with them. This way, all responses can be seen at once along with how they match up with the prototype response for each category. To have a better picture of our sample population, we included the following demographic variables in the survey: gender, main continent, career stage, and broad research area. The results are presented in Table 3. Based on these results it appeared that most of the respondents who filled out our survey were male (71.2%), living in Europe (51.1%), had a faculty position (94.1%), and were working in the field of psychology (56.1%). The total responses (i.e., including the responses of the respondents who quit filling out our survey) were very similar to the responses of the respondents who did complete our survey.

**Table 3:** Demographic variables.

Demographic variable	Frequency (%)	Frequency total (%)
Gender	N = 139	N = 175
Male	99 (71.2%)	124 (70.9%)
Female	37 (26.6%)	47 (26.9%)
Other	1 (0.7%)	1 (0.6%)
Prefer not to answer	2 (1.4%)	3 (1.7%)
Main continent	N = 139	N = 174
Asia	7 (5.0%)	7 (4.0%)
Africa	2 (1.4%)	3 (1.7%)
North America	52 (37.4%)	66 (37.9%)
South America	3 (2.2%)	3 (1.7%)
Europe	71 (51.1%)	91 (52.3%)
Australia	4 (2.9%)	4 (2.3%)
Career stage	N = 136	N = 171
PhD student	8 (5.9%)	8 (4.7%)
Faculty	128 (94.1%)	163 (95.3%)
Broad research area	N = 139	N = 175
Sociology	5 (3.6%)	6 (3.4%)
Political science	14 (10.1%)	19 (10.9%)
Psychology	78 (56.1%)	94 (53.7%)
Other	42 (30.2%)	56 (32%)

**Figure 3:**
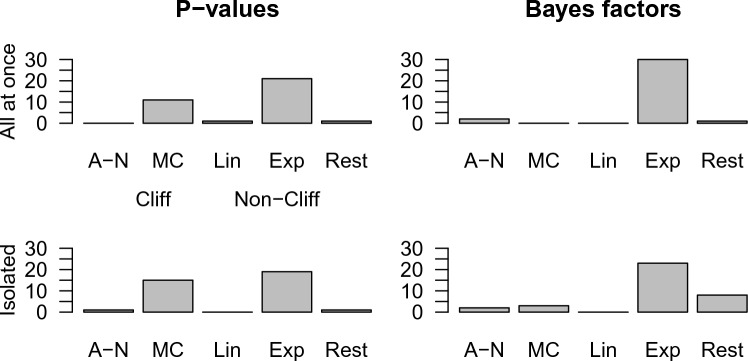
Plotted frequency of classification models within each condition.

To answer RQ1 (“What is the relation between obtained statistical evidence and the degree of belief or confidence that there is a positive effect in the population of interest across participants?”), we compared the frequency of the four classification models (i.e., all-or-none, moderate cliff, linear, and exponential) with one another within each of the four conditions (i.e., all at once and isolated *p*-values, and all at once and isolated BFs). The results are presented in Table 4. In order to enhance the interpretability of the results in Table 4, we have plotted them in Fig. 3.

**Table 4:** Frequency of classification models within each condition.

	*p*-Values		BFs	
	All at once	Isolated	All at once	Isolated
Cliff				
All-or-none	0	1	2	2
Moderate cliff	11	15	0	3
Total Cliff	11	16	2	5
Non-cliff				
Linear	1	0	0	0
Exponential	21	19	30	23
Total non-cliff	22	19	30	23
*N*				
Overall total	33	35	32	28

11 respondents were in the rest category.

We observe that within the all at once *p*-value condition, the cliff models accounted for a proportion of (0 + 11)/33 = 0.33 of the responses. The non-cliff models accounted for a proportion of (1 + 21)/33 = 0.67 of the responses. Looking at the isolated *p*-value condition, we can see that the cliff models accounted for a proportion of (1 + 15)/35 = 0.46 of the responses. The non-cliff models accounted for a proportion of (0 + 19)/35 = 0.54 of the responses. In the all at once BF condition, we observe that the cliff models accounted for a proportion of (2 + 0)/32 = 0.06 of the responses. The non-cliff models accounted for a proportion of (0 + 30)/32 = 0.94 of the responses. Finally, we observe that within the isolated BF condition, the cliff models accounted for a proportion of (2 + 3)/28 = 0.18 of the responses. The non-cliff models accounted for a proportion of (0 + 23)/28 = 0.82 of the responses.

Thus, we observed a higher proportion of cliff models in *p*-value conditions than in BF conditions (27/68 = 0.40 vs 7/60 = 0.12), and we observed a higher proportion of cliff models in isolated conditions than in all-at-once conditions (21/63 = 0.33 vs 13/65 = 0.20). Next, we conducted statistical inference to dive deeper into these observations.

To answer RQ2 (“What is the difference in this relationship when the statistical evidence is quantified through *p*-values versus Bayes factors?”), we compared the sample proportions mentioned above (27/68 = 0.40 and 7/60 = 0.12, respectively, with a difference between these proportions equal to 0.40–0.12 = 0.28), and we tested whether the proportion of cliff classifications in the *p*-value conditions differed from that in the BF conditions in the population by means of a Bayesian chi square test. For the chi square test, the null hypothesis was that there is no difference in the proportion of cliff classifications between the two conditions, and the alternative hypothesis was that there is a difference in the proportion of cliff classifications between the two conditions.

The BF that resulted from the chi square test was equal to BF_10_ = 140.01 and gives the relative evidence for the alternative hypothesis over the null hypothesis provided by the data. This means that the data are 140.01 times more likely under the alternative hypothesis than under the null hypothesis: we found strong support for the alternative hypothesis that there is a difference in the proportion of cliff classifications between the *p*-value and BF condition. Inspection of Table 4 or Fig. 3 shows that the proportion of cliff classifications is higher in the *p*-value conditions.

**Figure 4:**
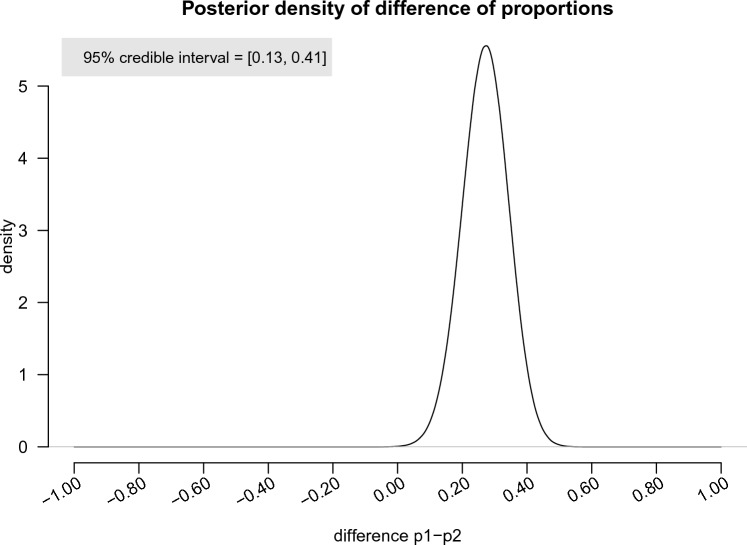
The posterior density of difference of proportions of cliff models in *p*-value conditions versus BF conditions.

Additionally, the posterior distribution of the difference in proportions is provided in Fig. 4, and the 95% credible interval was found to be [0.13, 0.41]. This means that there is a 95% probability that the population parameter for the difference of proportions of cliff classifications between *p*-value conditions and BF conditions lies within this interval, given the evidence provided by the observed data.

To answer RQ3 (“What is the difference in this relationship when the statistical evidence is presented in isolation versus all at once?”), we compared the sample proportions mentioned above (21/63 = 0.33 vs 13/65 = 0.20, respectively with a difference between these proportions equal to 0.33–0.20 = 0.13), and we tested whether the proportion of cliff classifications in the all or none conditions differed from that in the isolated conditions in the population by means of a Bayesian chi square test analogous to the test above.

The BF that resulted from the chi square test was equal to BF_10_ = 0.81, and gives the relative evidence for the alternative hypothesis over the null hypothesis provided by the data. This means that the data are 0.81 times more likely under the alternative hypothesis than under the null hypothesis: evidence on whether there is a difference in the proportion of cliff classifications between the isolation and all at once conditions is ambiguous.

**Figure 5:**
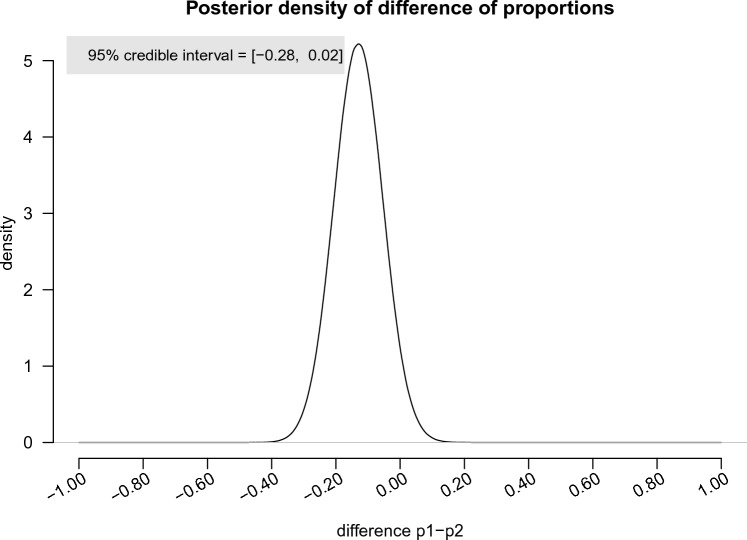
The posterior density of difference of proportions of cliff models in all at once conditions versus isolated conditions.

Additionally, the posterior distribution of the difference in proportions is provided in Fig. 5. The 95% credible interval is [− 0.28, 0.02].

There were 11 respondents who provided responses that extremely deviated from the four relationship models, so they were included in the rest category, and were left out of the analyses. Eight of these were in the isolated BF condition, one was in the isolated *p*-value condition, one was in the all at once BF condition, and one was in the all at once *p*-value condition. For five of these, their outcomes resulted in a roughly decreasing trend with significant large bumps. For four of these, there were one or more considerable increases in the plotted outcomes. For two of these, the line was flat. All these graphs are available in Appendix 2.

## Discussion

In the present study, we explored the relationship between obtained statistical evidence and the degree of belief or confidence that there is a positive effect in the population of interest. We were in particular interested in the existence of a cliff effect. We compared this relationship for *p*-values to the relationship for corresponding degrees of evidence quantified through Bayes factors, and we examined whether this relationship was affected by two different modes of presentation. In the isolated presentation mode a possible clear functional form of the relationship across values was not visible to the participants, whereas in the all-at-once presentation mode, such a functional form could easily be seen by the participants.

The observed proportions of cliff models was substantially higher for the *p*-values than for the BFs, and the credible interval as well as the high BF test value indicate that a (substantial) difference will also hold more generally at the population level. Based on our literature review (summarized in Table 1), we did not know of studies that have compared the prevalence of cliff effect when interpreting *p*-values to that when interpreting BFs, so we think that this part is new in the literature. However, our findings are consistent with previous literature regarding the presence of a cliff effect when using *p*-values. Although we observed a higher proportion of cliff models for isolated presentations than for all-at-once presentation, we did not get a clear indication from the present results whether or not, at the population level, these proportion differences will also hold. We believe that this comparison between the presentation methods that have been used to investigate the cliff effect is also new. In previous research, the *p*-values were presented on separate pages in some studies^15^, while in other studies the *p*-values were presented on the same page^13^.

We deviated from our preregistered sampling plan by collecting the e-mail addresses of all corresponding authors who published in the 20 journals in social and behavioural sciences in 2021 and 2022 simultaneously, rather than sequentially. We do not believe that this approach created any bias in our study results. Furthermore, we decided that it would not make sense to collect additional data (after approaching 5399 academics who published in 2019, 2020, 2021, and 2022 in the 20 journals) in order to reach an effective sample size of 200. Based on our interim power analysis, the strongest possible pro-null evidence we could get if we continued collecting data up to an effective sample size of 200 given the data we already had would be BF_10_ = 0.14 or BF_01_ = 6.99. Therefore, we decided that it would be unethical to continue collecting additional data.

There were several limitations in this study. Firstly, the response rate was very low. This was probably the case because many academics who we contacted mentioned that they were not familiar with interpreting Bayes factors. It is important to note that our findings apply only to researchers who are at least somewhat familiar with interpreting Bayes factors, and our sample does probably not represent the average researcher in the social and behavioural sciences. Indeed, it is well possible that people who are less familiar with Bayes factors (and possibly with statistics in general) would give responses that were even stronger in line with cliff models, because we expect that researchers who exhibit a cliff effect will generally have less statistical expertise or understanding: there is nothing special about certain *p*-value or Bayes factor thresholds that merits a qualitative drop in the perceived strength of evidence. Furthermore, a salient finding was that the proportion of graduate students was very small. In our sample, the proportion of graduate students showing a cliff effect is 25% and the proportion of more senior researchers showing a cliff effect is 23%. Although we see no clear difference in our sample, we cannot rule out that our findings might be different if the proportion of graduate students in our sample would be higher.

There were several limitations related to the survey. Some of the participants mentioned via e-mail that in the scenarios insufficient information was provided. For example, we did not provide effect sizes and any information about the research topic. We had decided to leave out this information to make sure that the participants could only focus on the *p*-values and the Bayes factors. Furthermore, the questions in our survey referred to posterior probabilities. A respondent noted that without being able to evaluate the prior plausibility of the rival hypotheses, the questions were difficult to answer. Although this observation is correct, we do think that many respondents think they can do this nevertheless.

The respondents could indicate their degree of belief or confidence that there is a positive effect in the population of interest based on the fictitious findings on a scale ranging from 0 (completely convinced that there is no effect), through 50 (somewhat convinced that there is a positive effect), to 100 (completely convinced that there is a positive effect). A respondent mentioned that it might be unclear where the midpoint is between somewhat convinced that there is no effect and somewhat convinced that there is a positive effect, so biasing the scale towards yes response. Another respondent mentioned that there was no possibility to indicate no confidence in either the null or the alternative hypothesis. Although this is true, we do not think that many participants experienced this as problematic.

In our exploratory analyses we observed that eight out of eleven unclassifiable responses were in the isolated BF condition. In our survey, the all at once and isolated presentation conditions did not only differ in the way the pieces of statistical evidence were presented, but they also differed in the order. In all at once, the different pieces were presented in sequential order, while in the isolated condition, they were presented in a random order. Perhaps this might be an explanation for why the isolated BF condition contained most of the unclassifiable responses. Perhaps academics are more familiar with single *p*-values and can more easily place them along a line of “possible values” even if they are presented out of order.

This study indicates that a substantial proportion of researchers who are at least somewhat familiar with interpreting BFs experience a sharp drop in confidence when an effect exists around certain *p*-values and to a much smaller extent around certain Bayes factor values. But how do people act on these beliefs? In a recent study by Muradchanian et al.^24^, it was shown that editors, reviewers, and authors alike are much less likely to accept for publication, endorse, and submit papers with non-significant results than with significant results, suggesting these believes about the existence of an effect translate into considering certain findings more publication-worthy.

Allowing for these caveats, our findings showed that cliff models were more prevalent when interpreting *p*-values than when interpreting BFs, based on a sample of academics who were at least somewhat familiar with interpreting BFs. However, the high prevalence of the non-cliff models (i.e., linear and exponential) implied that *p*-values do not necessarily entail dichotomous thinking for everyone. Nevertheless, it is important to note that the cliff models were still accountable for 37.5% of responses in *p*-values, whereas in BFs, the cliff models were only accountable for 12.3% of the responses.

We note that dichotomous thinking has a place in interpreting scientific evidence, for instance in the context of decision criteria (if the evidence is more compelling than some a priori agreed level, then we bring this new medicine to the market), or in the context of sampling plans (we stop collecting data once the evidence or level of certainty hits some a priori agreed level). However, we claim that it is not rational for someone’s subjective belief that some effect is non-zero to make a big jump around for example a *p*-value of 0.05 or a BF of 10, but not at any other point along the range of potential values.

Based on our findings, one might think replacing *p*-values with BFs might be sufficient to overcome dichotomous thinking. We think that this is probably too simplistic. We believe that rejecting or not rejecting a null hypothesis is probably so deep-seated in the academic culture that dichotomous thinking might become more and more prevalent in the interpretation of BFs in time. In addition to using tools such as *p*-values or BFs, we agree with Lai et al.^13^ that several ways to overcome dichotomous thinking in *p*-values, BFs, etc. are to focus on teaching (future) academics to formulate research questions requiring quantitative answers such as, for example, evaluating the extent to which therapy A is superior to therapy B rather than only evaluating that therapy A is superior to therapy B, and adopting effect size estimation in addition to statistical hypotheses in both thinking and communication.

In light of the results regarding dichotomous thinking among researchers, future research can focus on, for example, the development of comprehensive teaching methods aimed at cultivating the skills necessary for formulating research questions that require quantitative answers. Pedagogical methods and curricula can be investigated that encourage adopting effect size estimation in addition to statistical hypotheses in both thinking and communication.

### Supplementary Information

Supplementary Information 1.

Supplementary Information 2.

Supplementary Information 3.

Supplementary Information 4.

## Data Availability

The raw data are available within the OSF repository: https://osf.io/ndaw6/.

## Code availability

For the generation of the *p*-values and BFs, the R file “2022-11-04 psbfs.R” can be used; for Fig. 1, the R file “2021-06-03 ProtoCliffPlots.R” can be used; for the posterior for the difference between the two proportions in RQ2 and RQ3, the R file “2022-02-17 R script posterior for difference between two proportions.R” can be used; for the Bayesian power simulation, the R file “2022-11-04 Bayes Power Sim Cliff.R” can be used; for calculating the Bayes factors in RQ2 and RQ3 the R file “2022-10-21 BFs RQ2 and RQ3.R” can be used; for the calculation of Cohen’s kappa, the R file “2023-07-23 Cohens kappa.R” can be used; for data preparation, the R file “2023-07-23 data preparation.R” can be used; for Fig. 2, the R file “2024-03-11 data preparation including Fig. 2.R” can be used; for the interim power analysis, the R file “2024-03-16 Interim power analysis.R” can be used; for Fig. 3, the R file “2024-03-16 Plot for Table 4 R” can be used. The R codes were written in R version 2022.2.0.443, and are uploaded as part of the supplementary material. These R codes are made available within the OSF repository: https://osf.io/ndaw6/.
