# Supplementary material for: Comparing researchers’ degree of dichotomous thinking using frequentist versus Bayesian null hypothesis testing
Source: Sci Rep. 2024 May 27;14:12120. doi: 10.1038/s41598-024-62043-w (PMC11130270; doi:10.1038/s41598-024-62043-w)
Supplement: Supplementary file 1 — Supplementary Information 1. [file 41598_2024_62043_MOESM1_ESM.pdf]

Appendix 1: Instructions classification

Please try to classify the data into one of the four relationship models as much as possible. The main focus is on the nature of the relationship between the *p*-values/BFs and the degree of belief or confidence, rather than on other features such as how high (or low) the plots are on the y-axis.

Linear model: all data that show a linear relationship between the *p*-values or corresponding BF values and the degree of belief or confidence. The relationship can be both positive or negative. Small drops in the degree of confidence are allowed.

Exponential: all data that show an exponential relationship between the *p*-values or corresponding BF values and the degree of belief or confidence. The relationship can be both increasing or decreasing. Small drops in the degree of confidence are allowed.

All-or-none: all data that show a dichotomous relationship between the *p*-values or corresponding BF values and the degree of belief or confidence. The cliff can be of both a positive or a negative nature. The very high and low levels of confidence should be quite stable (i.e., no increase or decrease in the level of confidence when moving from one *p*-value/BF level to the next *p*-value/BF level). Only a single cliff per individual is allowed.

Moderate cliff: all data that show a relationship that represents a combination of the linear and the all-or-none function, and a relationship that represents a combination of the exponential and the all-or-none function. The relationship can be both of a positive or a negative nature.

Rest category: if the data deviate extremely from the previously described four relationship models, then include these data in the rest category. Possible examples of such relationships would be sinusoidal and parabola functions.
