# Supplementary material for: Comparing researchers’ degree of dichotomous thinking using frequentist versus Bayesian null hypothesis testing
Source: Sci Rep. 2024 May 27;14:12120. doi: 10.1038/s41598-024-62043-w (PMC11130270; doi:10.1038/s41598-024-62043-w)
Supplement: Supplementary file 2 — Supplementary Information 2. [file 41598_2024_62043_MOESM2_ESM.pdf]

Appendix 2: Plot for each participant

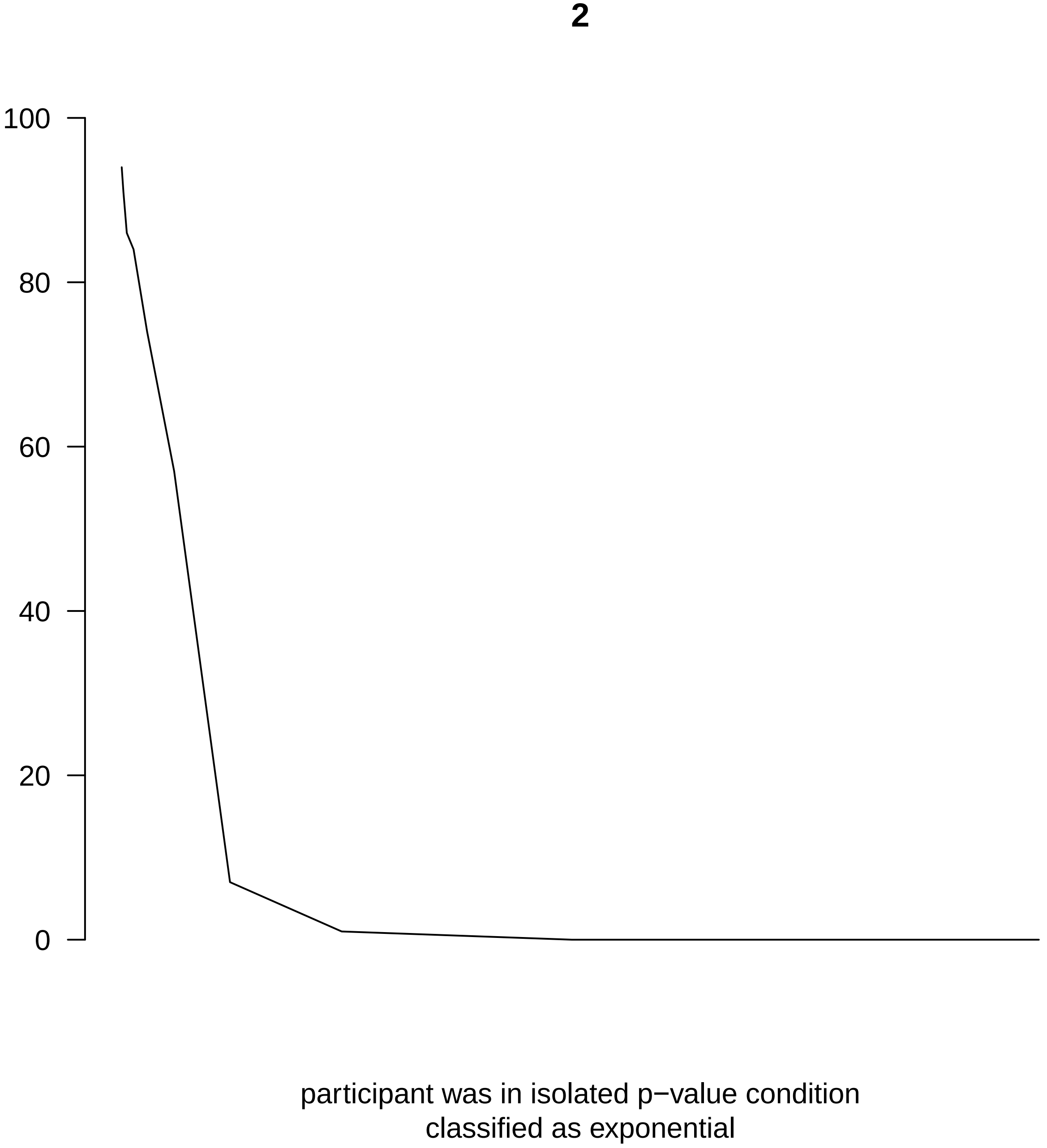

6

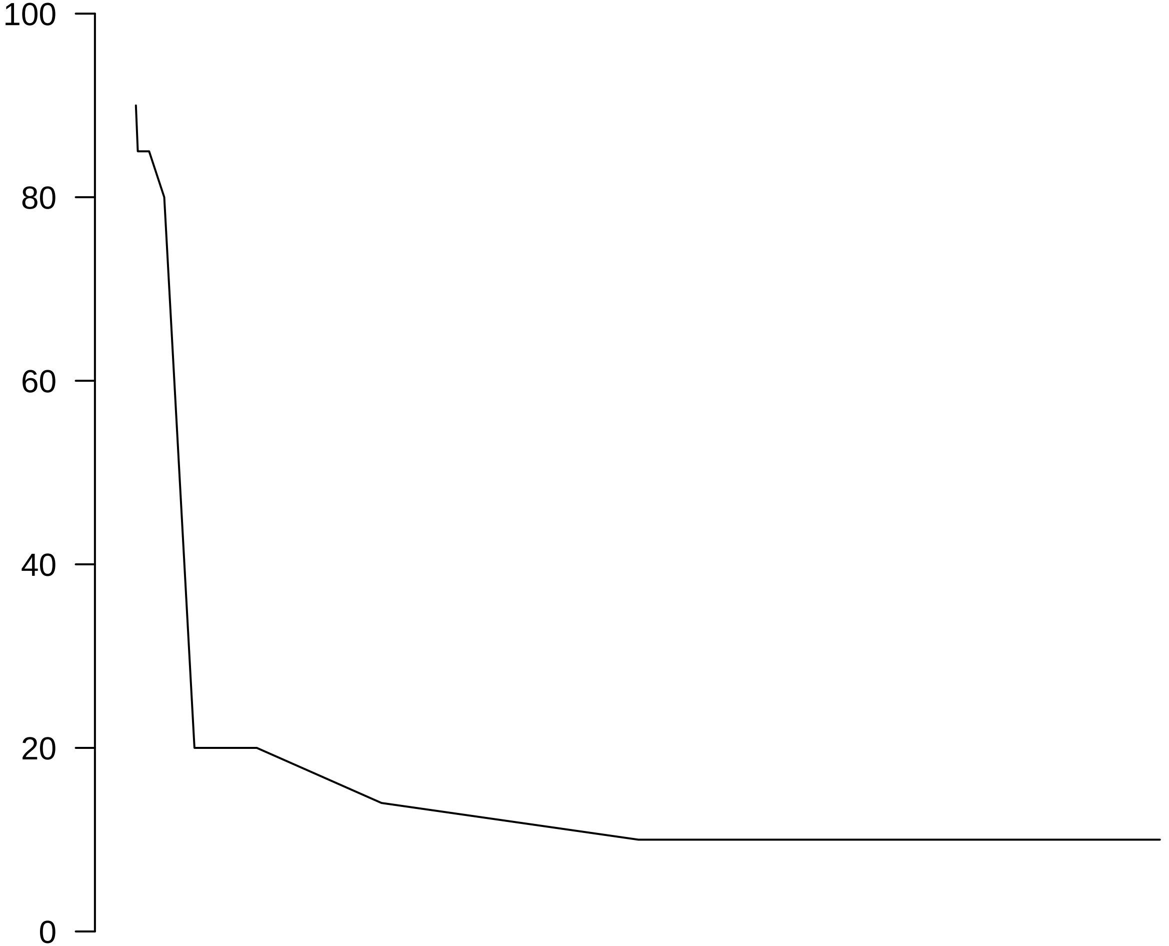

participant was in isolated p−value condition
classified as exponential

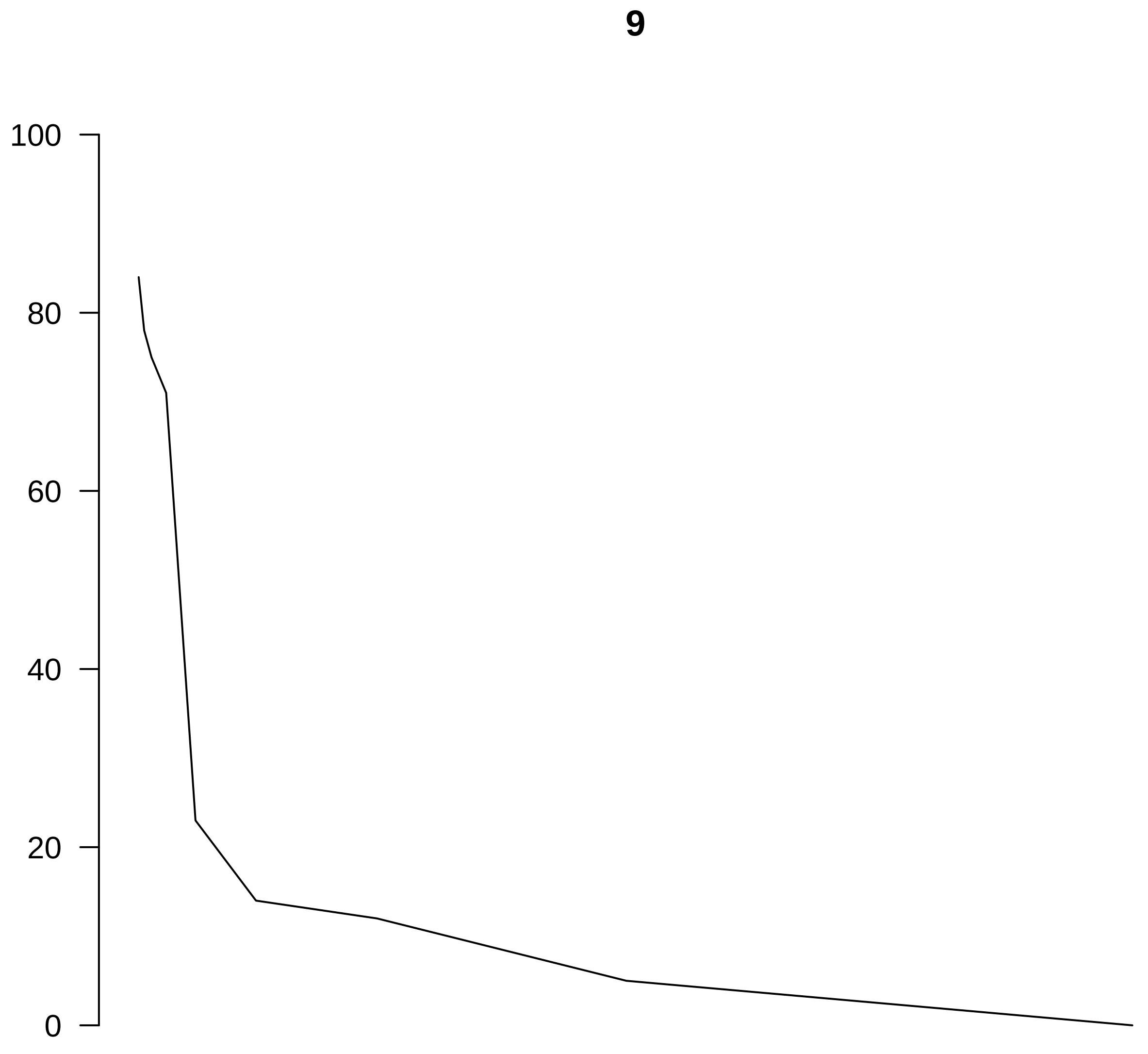

participant was in isolated p−value condition
classified as exponential

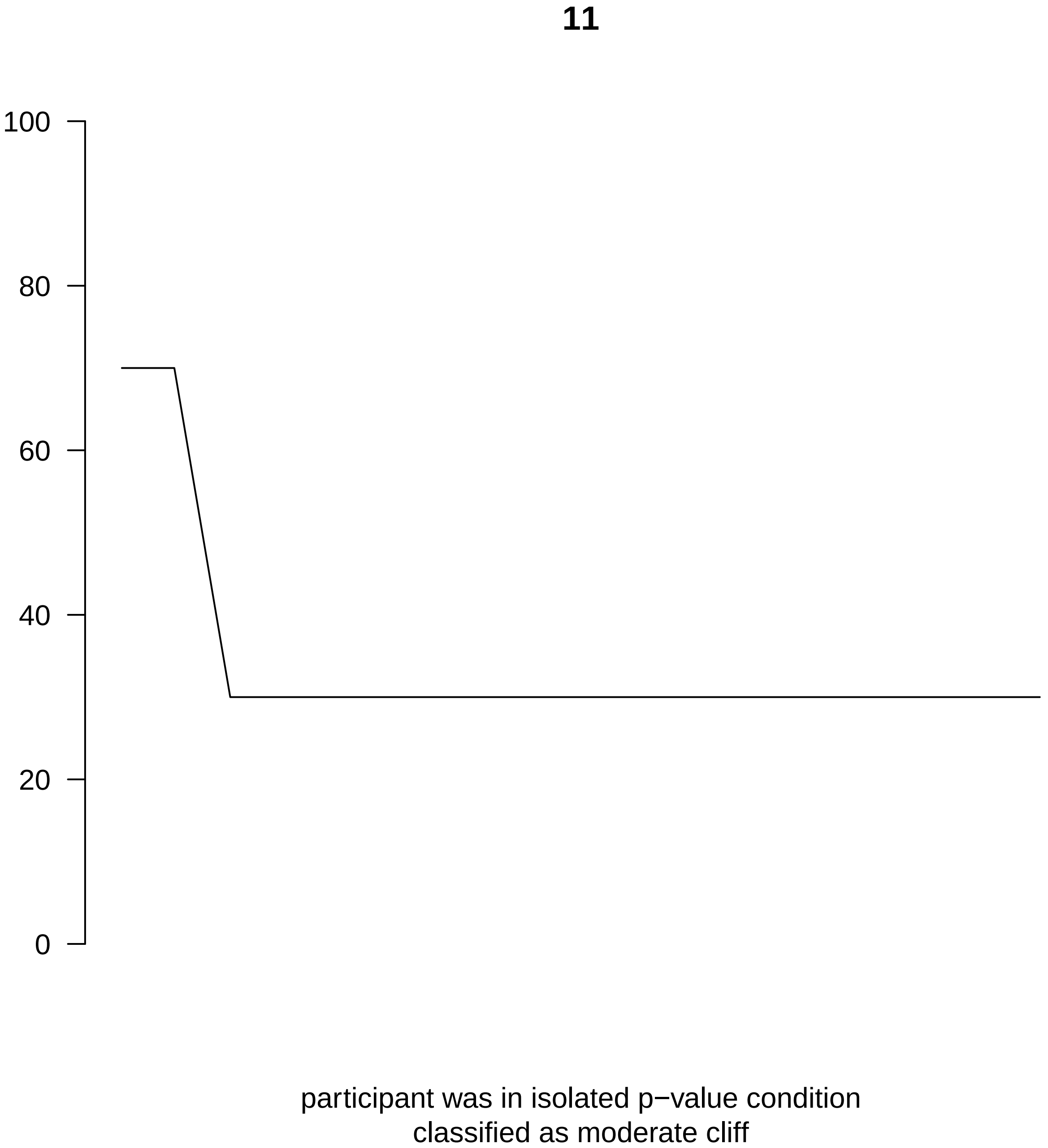
11
100
80
60
40
20
0
participant was in isolated p−value condition
classified as moderate cliff

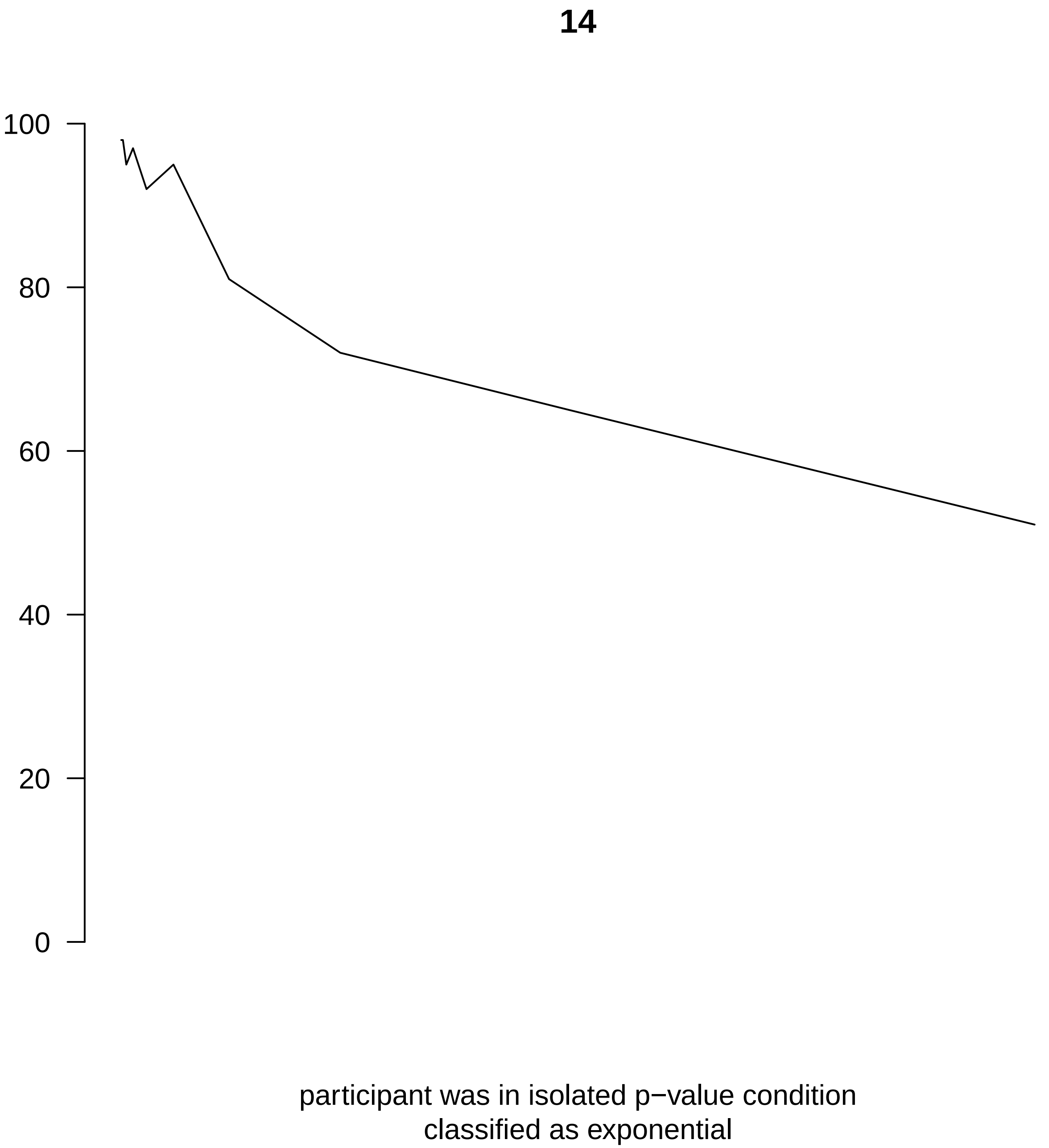
14
100
80
60
40
20
0
participant was in isolated p−value condition
classified as exponential

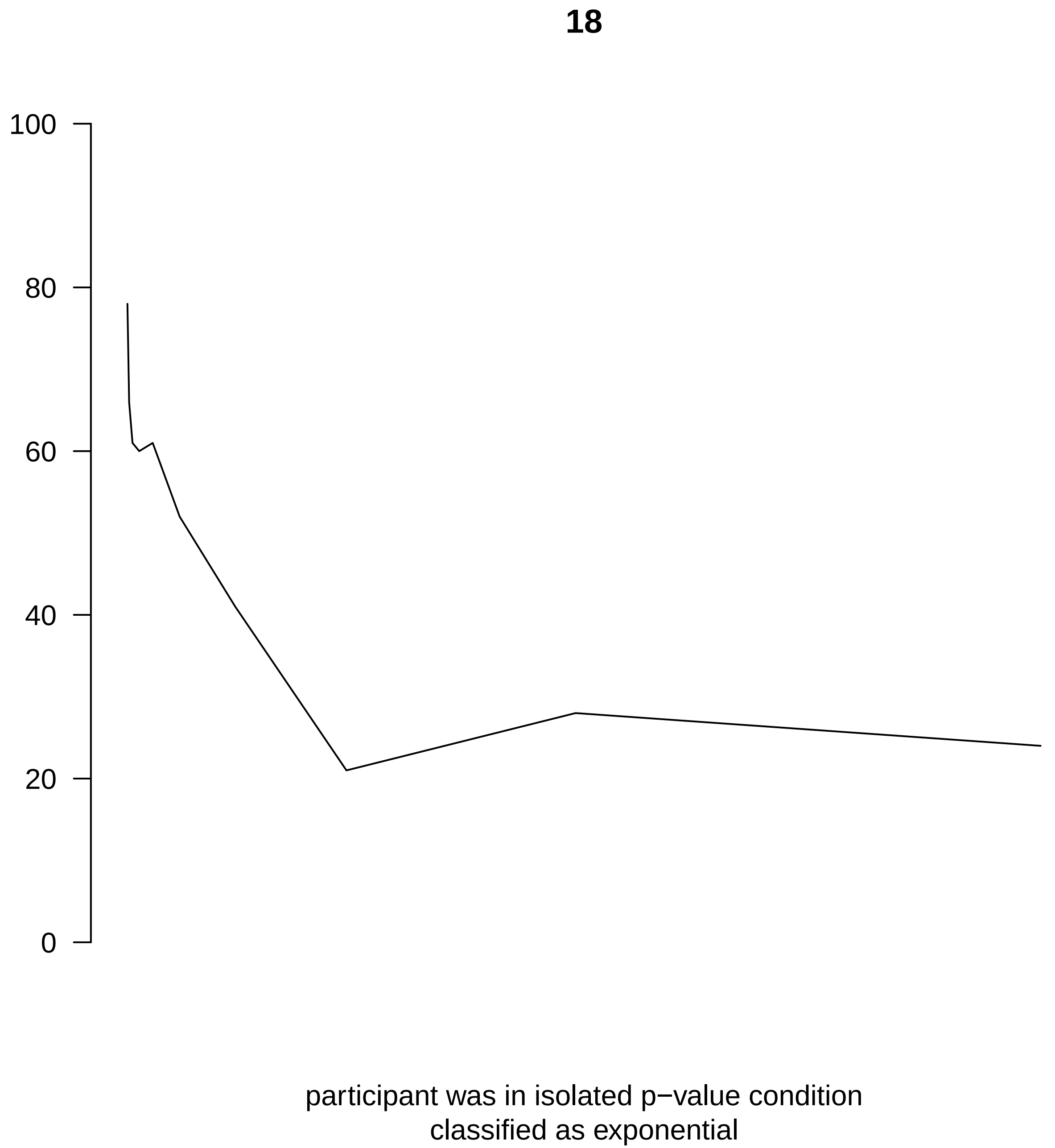
18
100
80
60
40
20
0
participant was in isolated p−value condition
classified as exponential

20

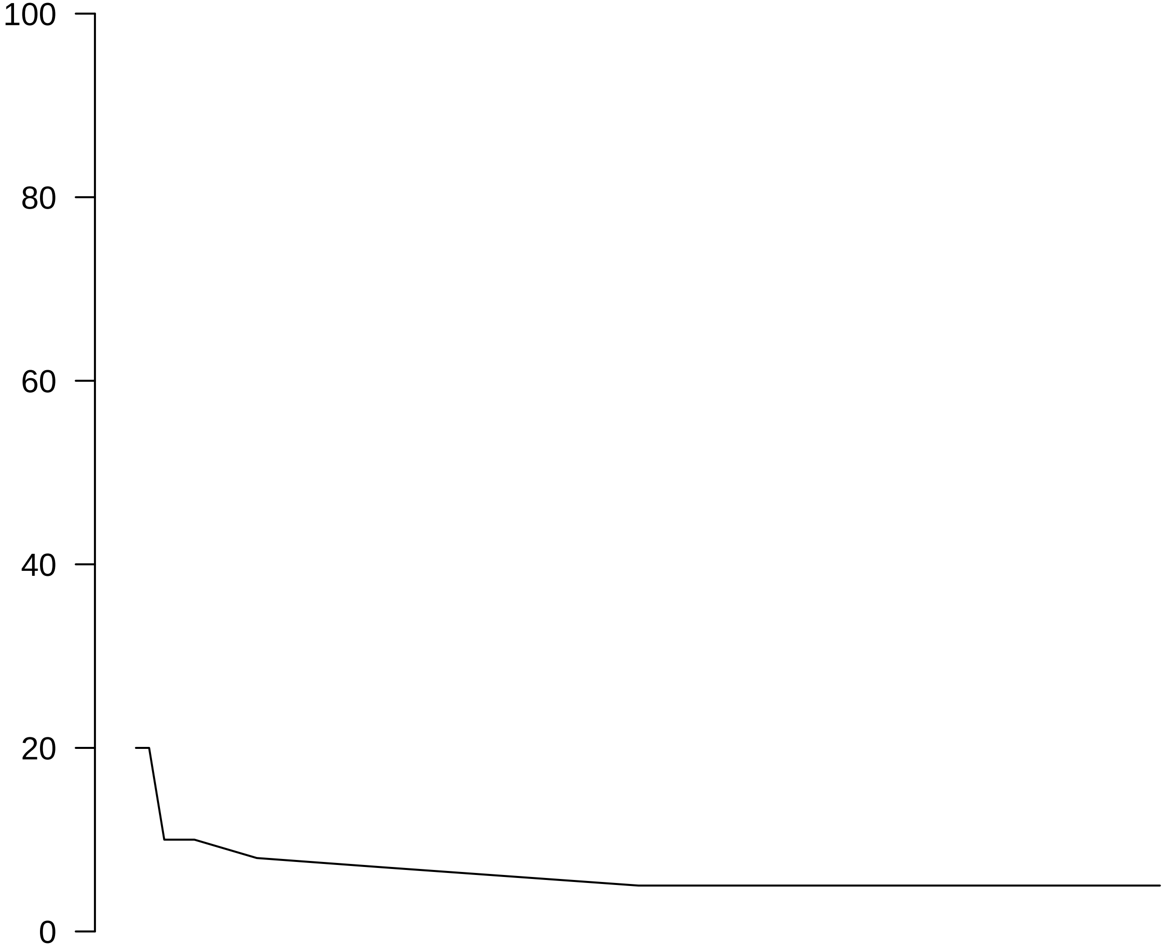

participant was in isolated p−value condition
classified as moderate cliff

23

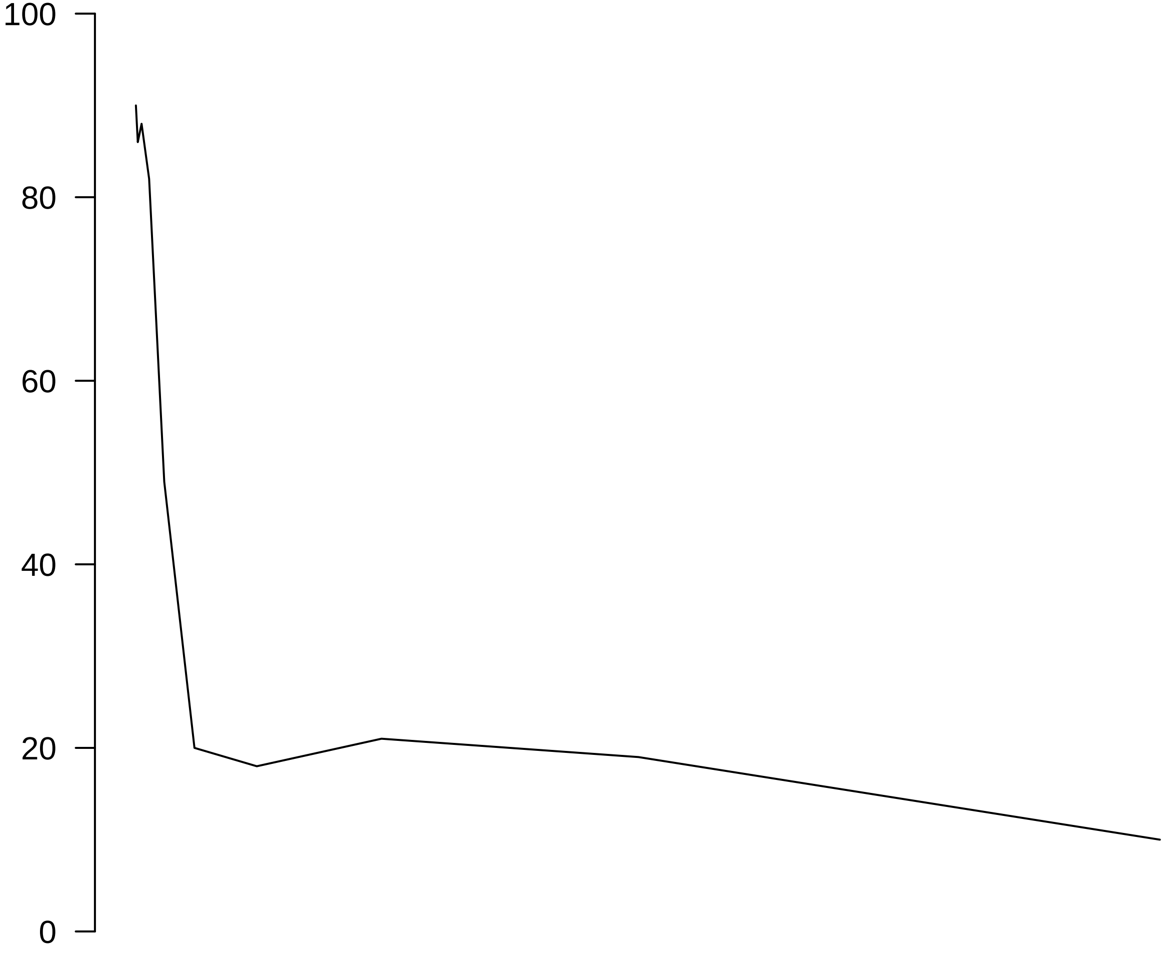

participant was in isolated p−value condition
classified as moderate cliff

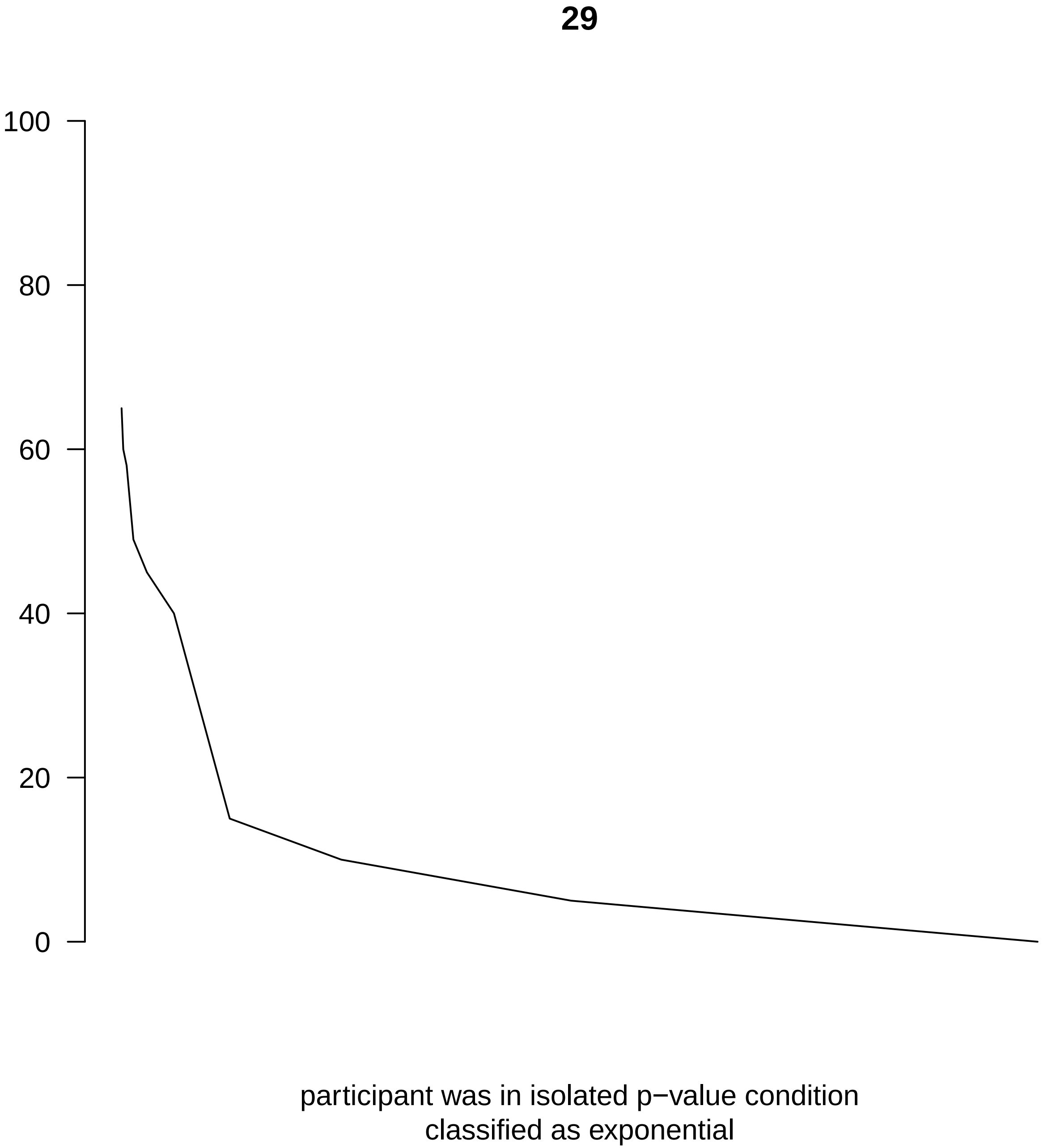
29
100
80
60
40
20
0
participant was in isolated p−value condition
classified as exponential

35

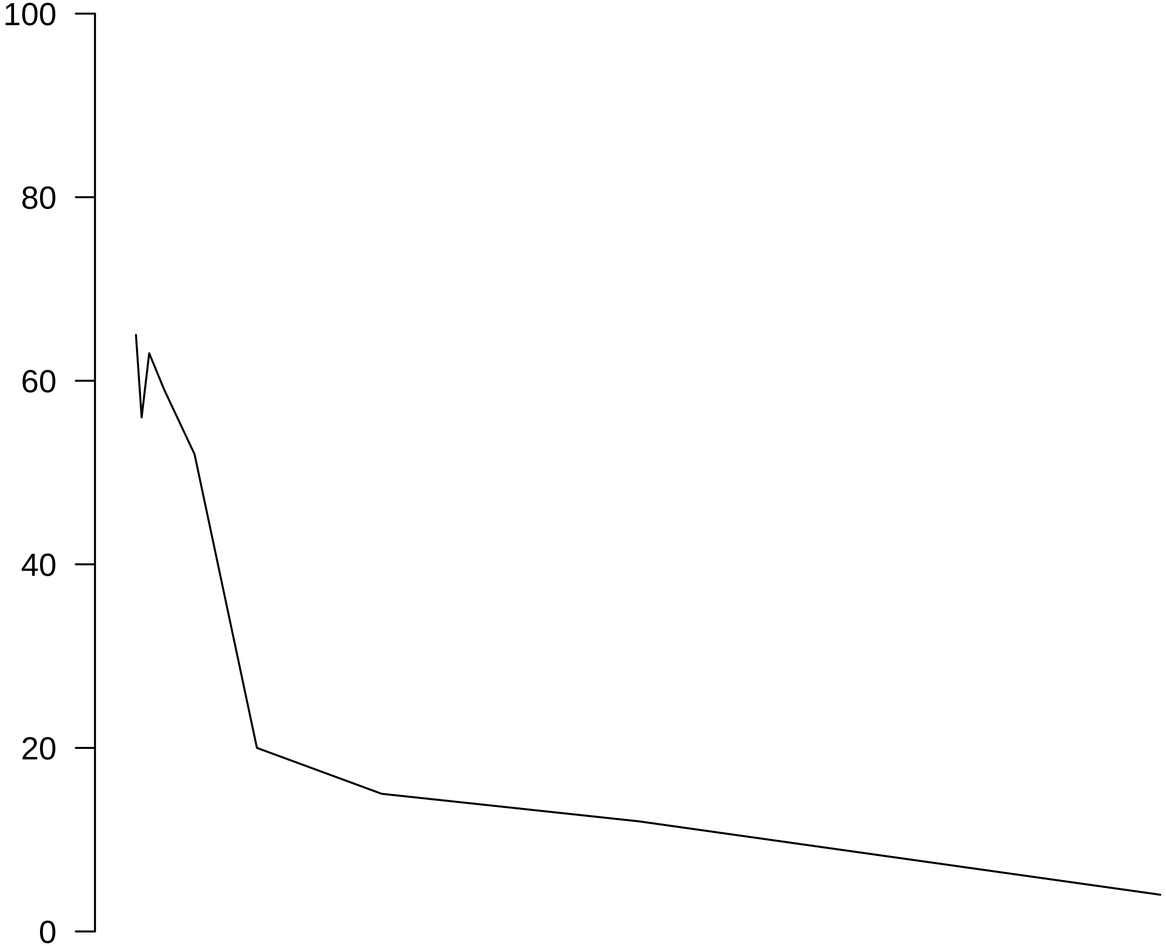

participant was in isolated p−value condition
classified as moderate cliff

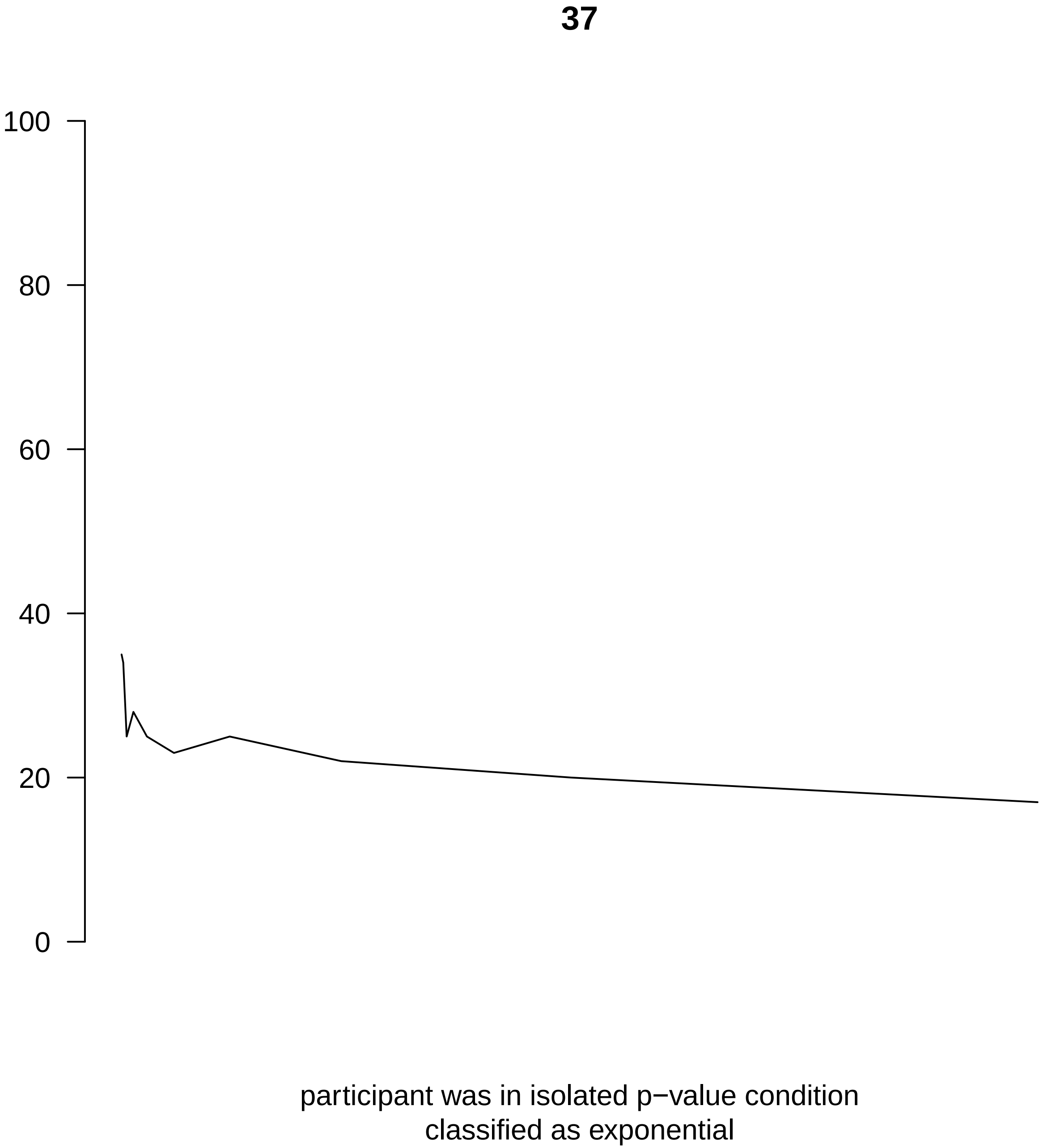
37
100
80
60
40
20
0
participant was in isolated p−value condition
classified as exponential

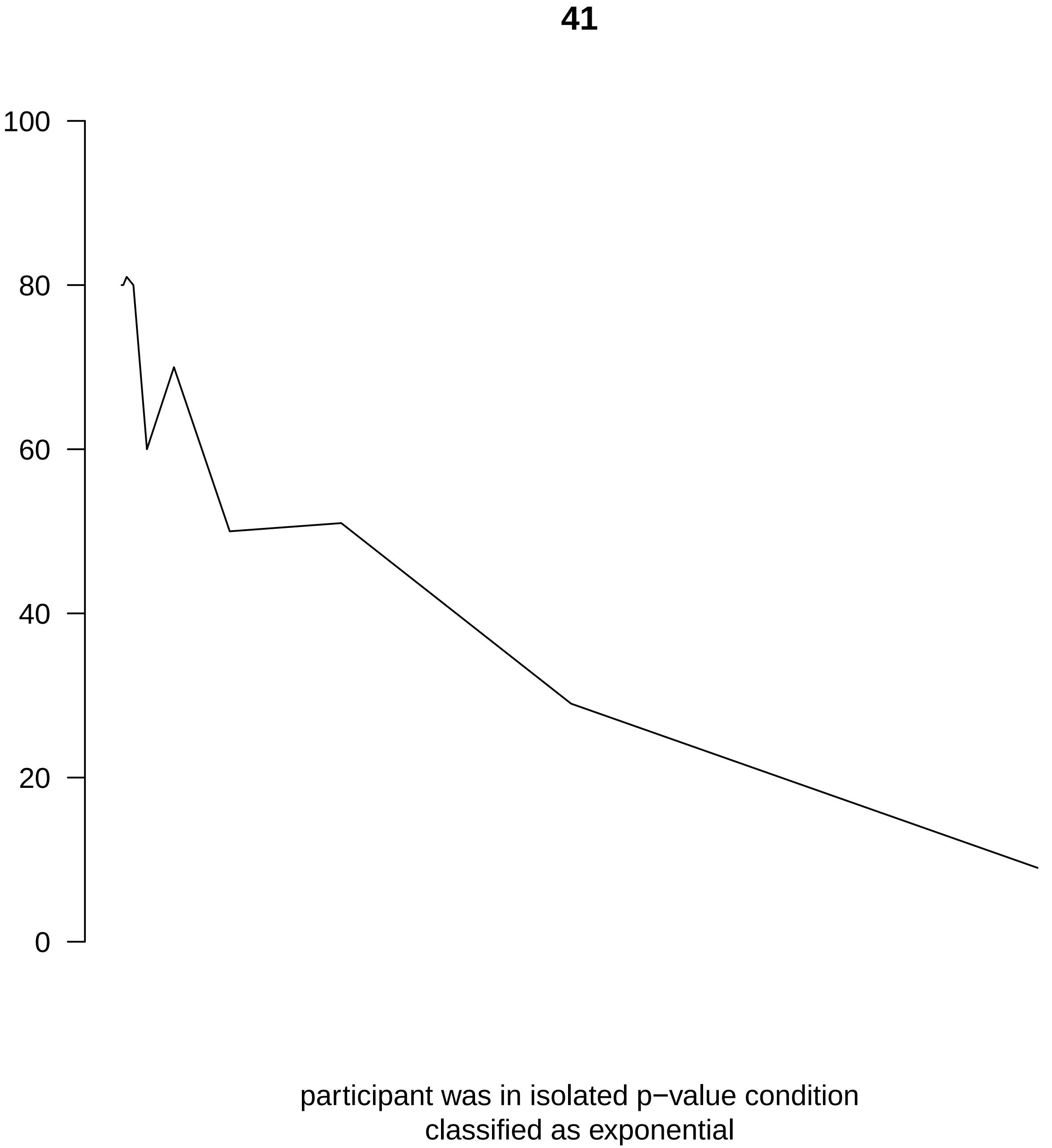
41
100
80
60
40
20
0
participant was in isolated p−value condition
classified as exponential

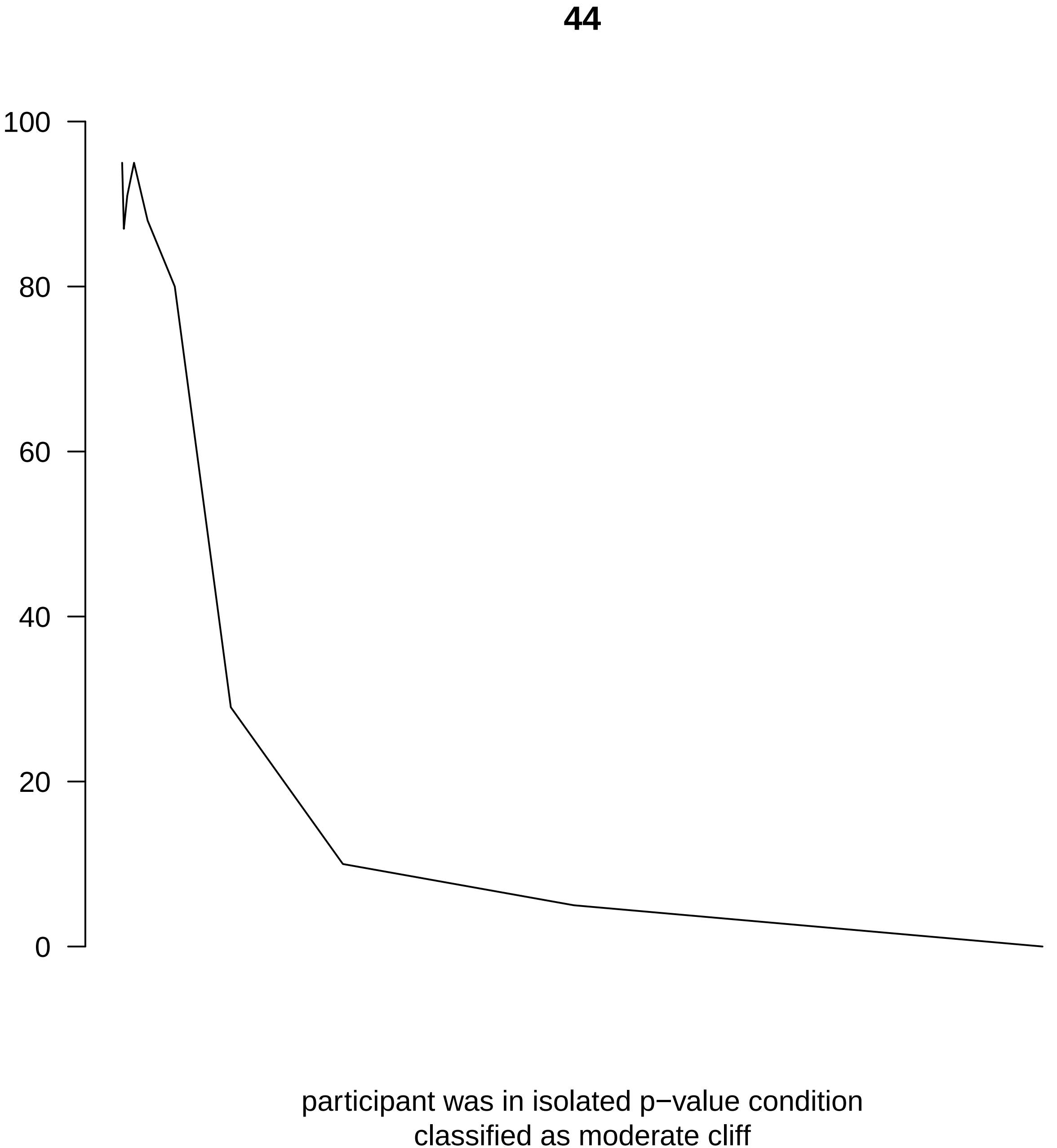
44
100
80
60
40
20
0
participant was in isolated p−value condition
classified as moderate cliff

47

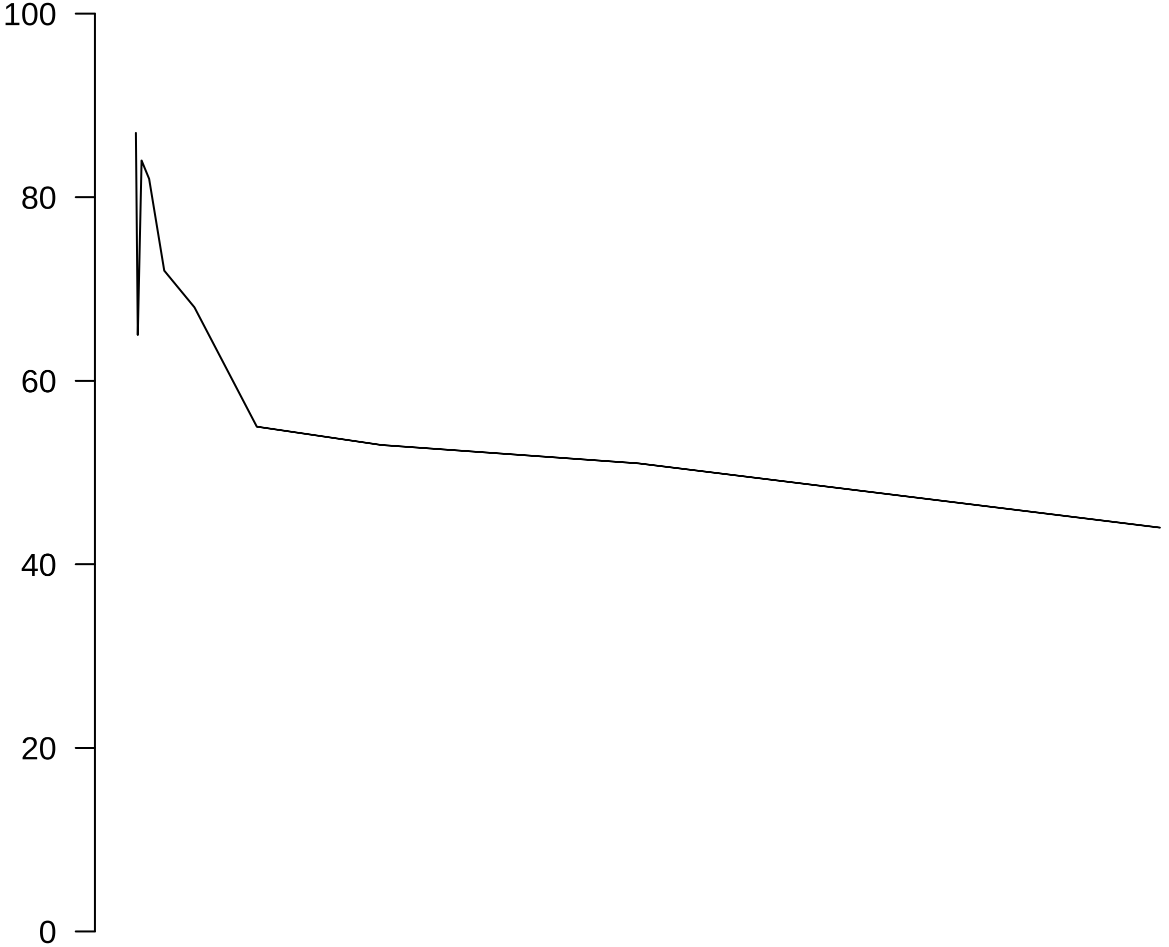

participant was in isolated p−value condition
classified as exponential

58

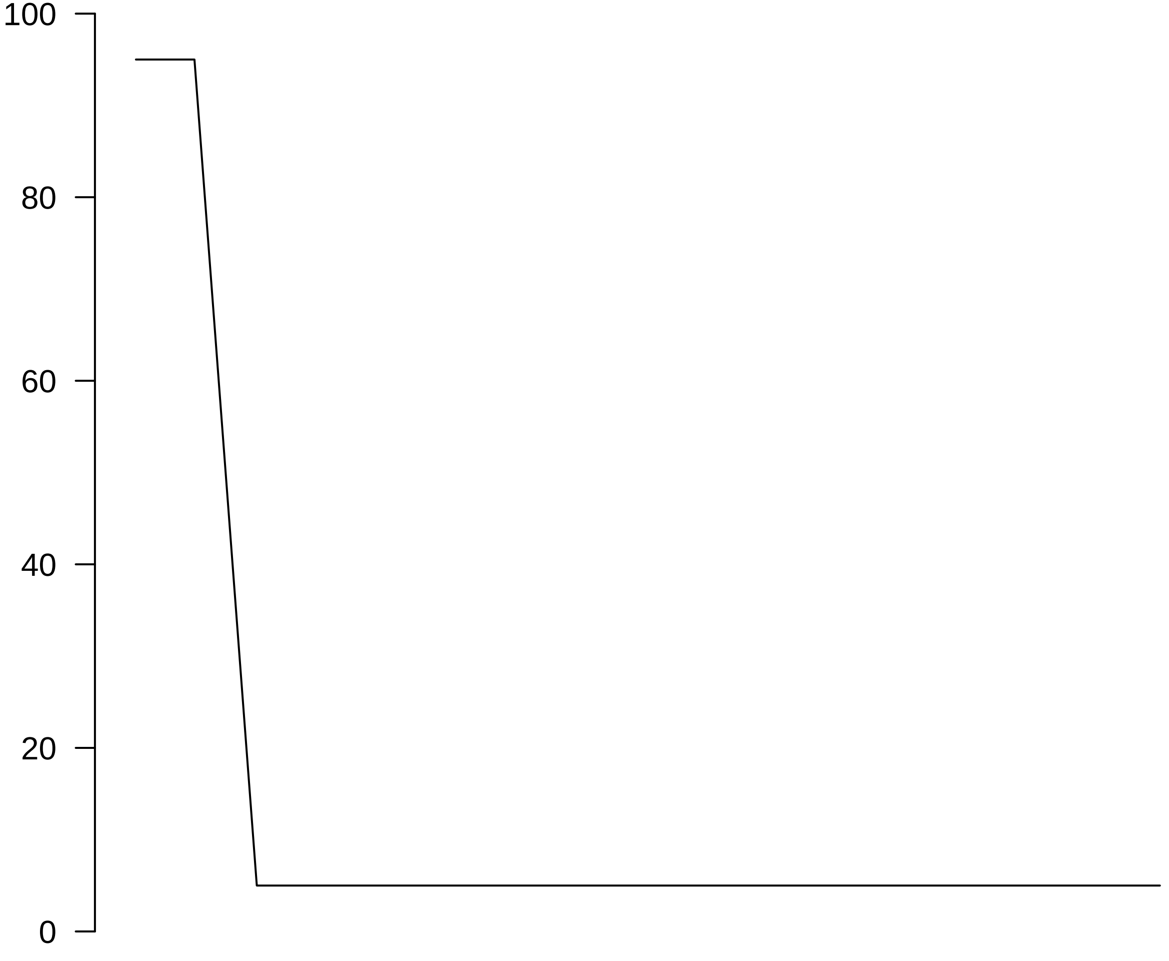

participant was in isolated p−value condition
classified as all or none

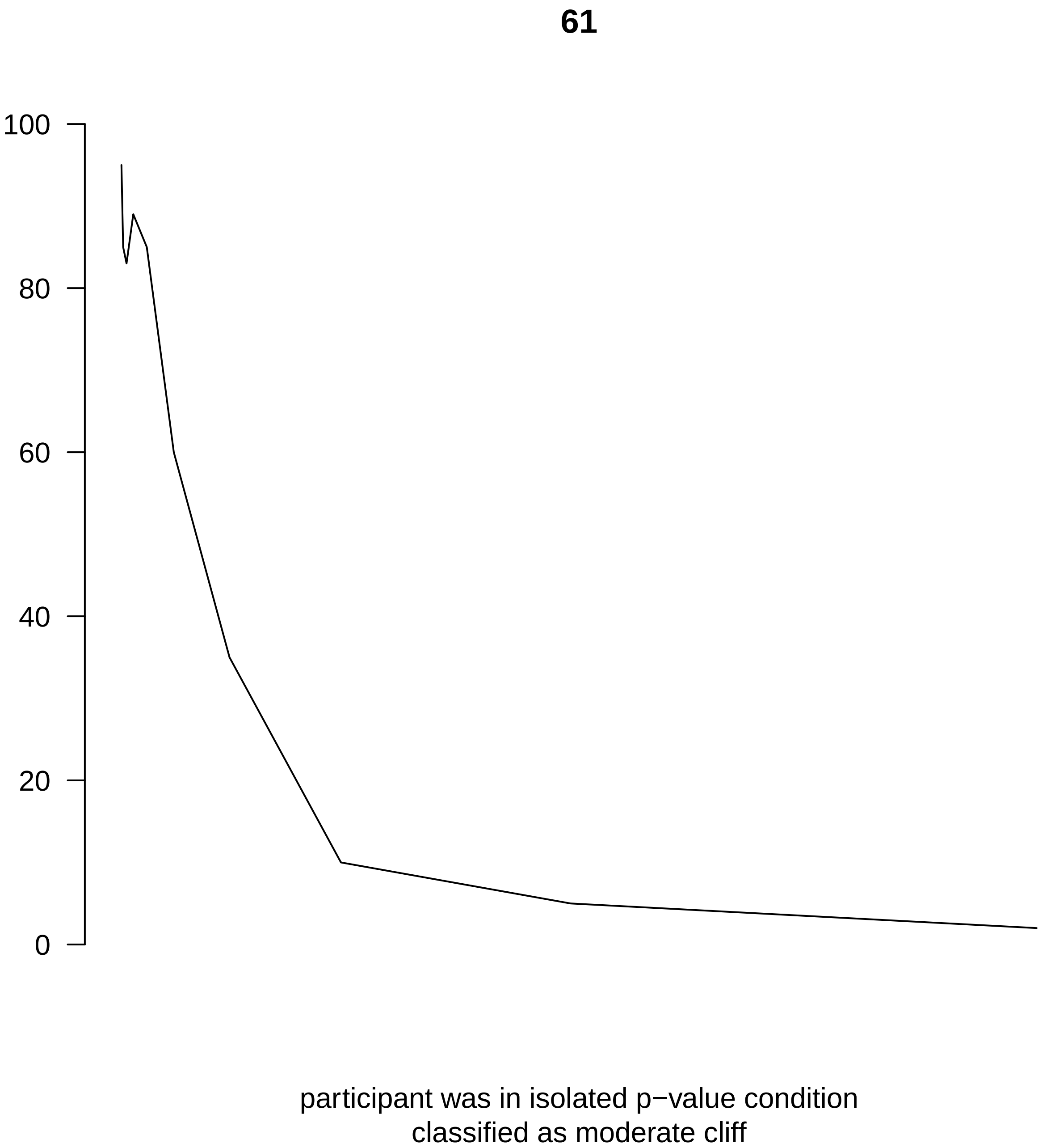
61
100
80
60
40
20
0
participant was in isolated p−value condition
classified as moderate cliff

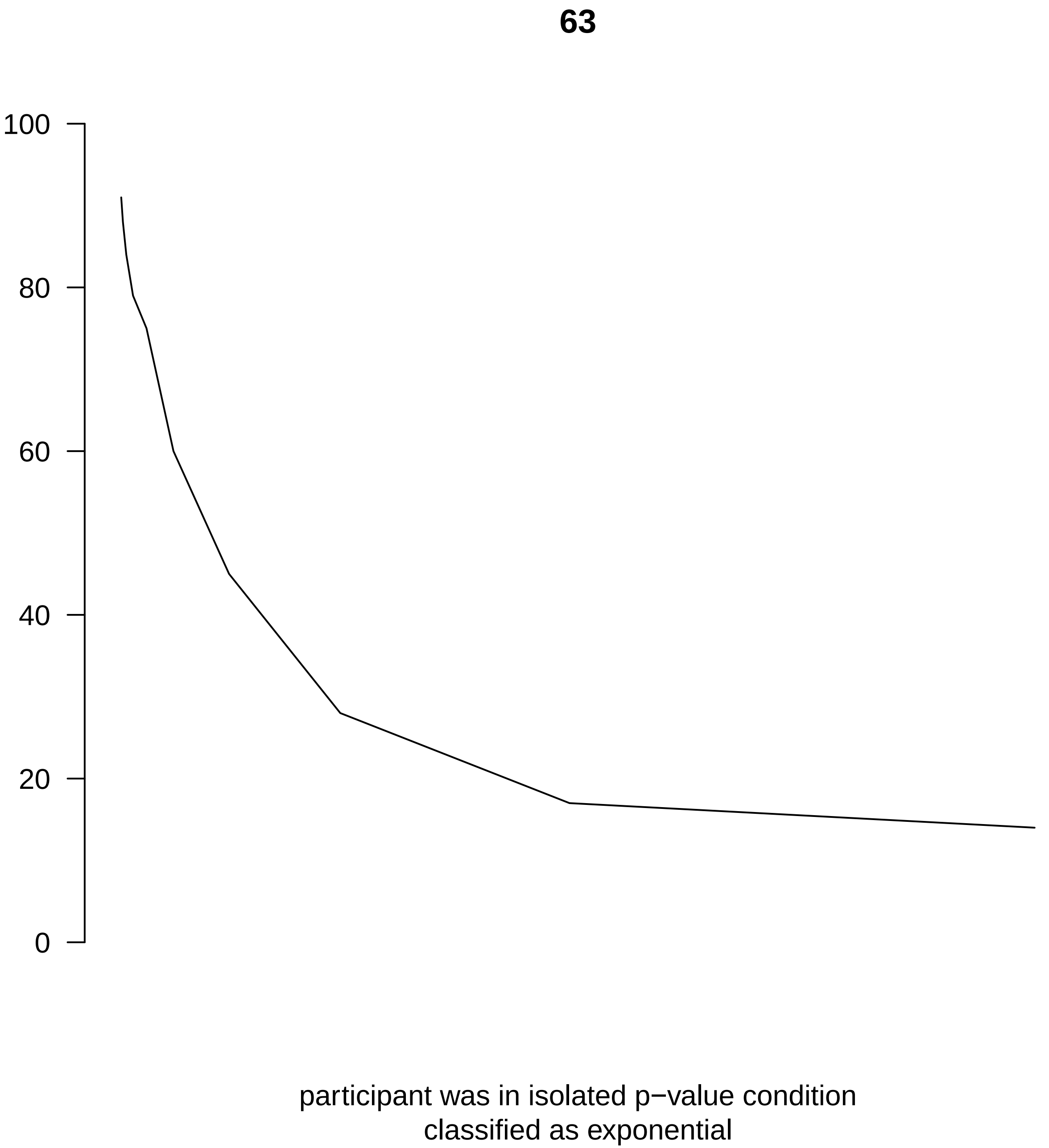
63
100
80
60
40
20
0
participant was in isolated p−value condition
classified as exponential

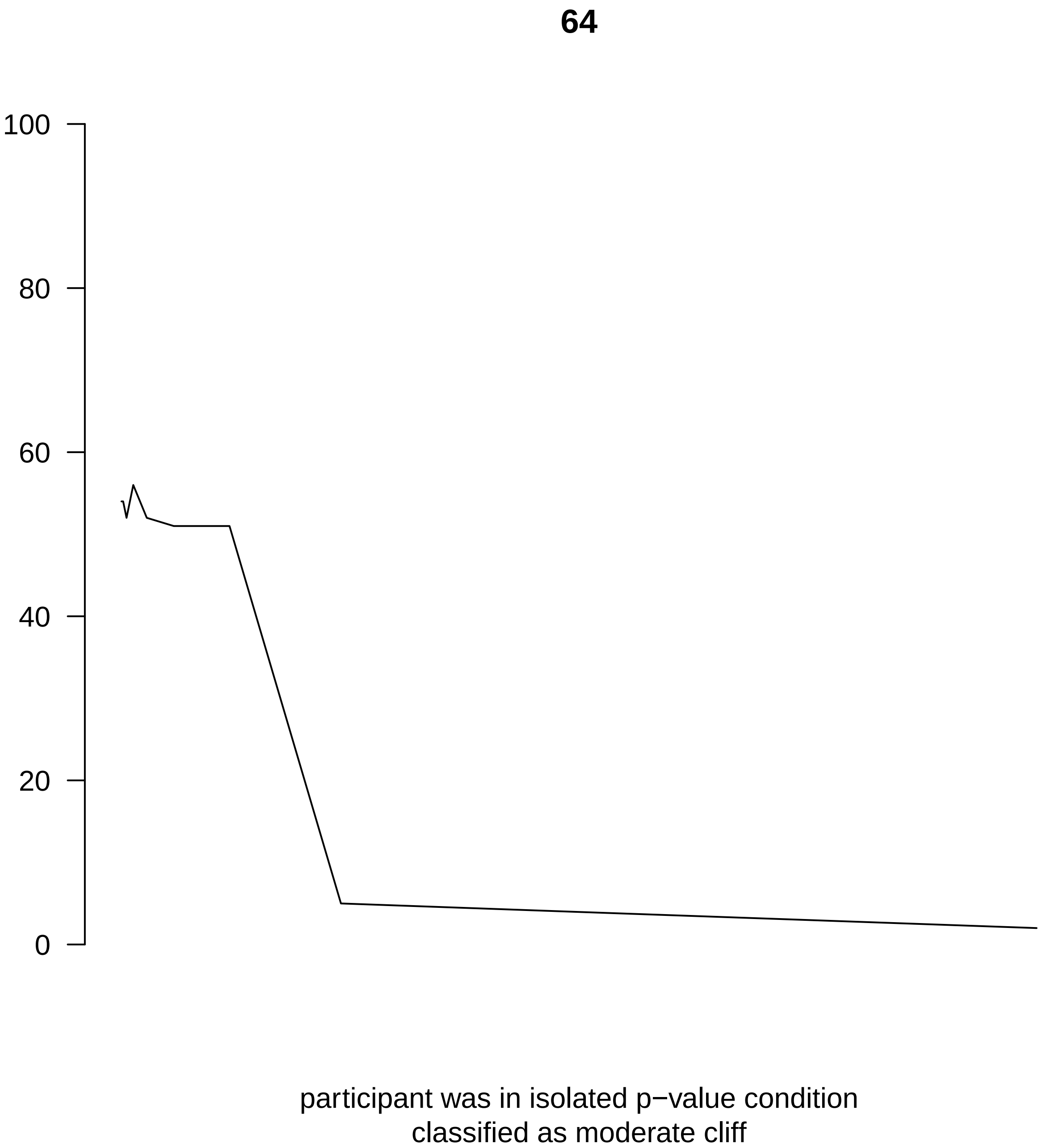
64
100
80
60
40
20
0
participant was in isolated p−value condition
classified as moderate cliff

67

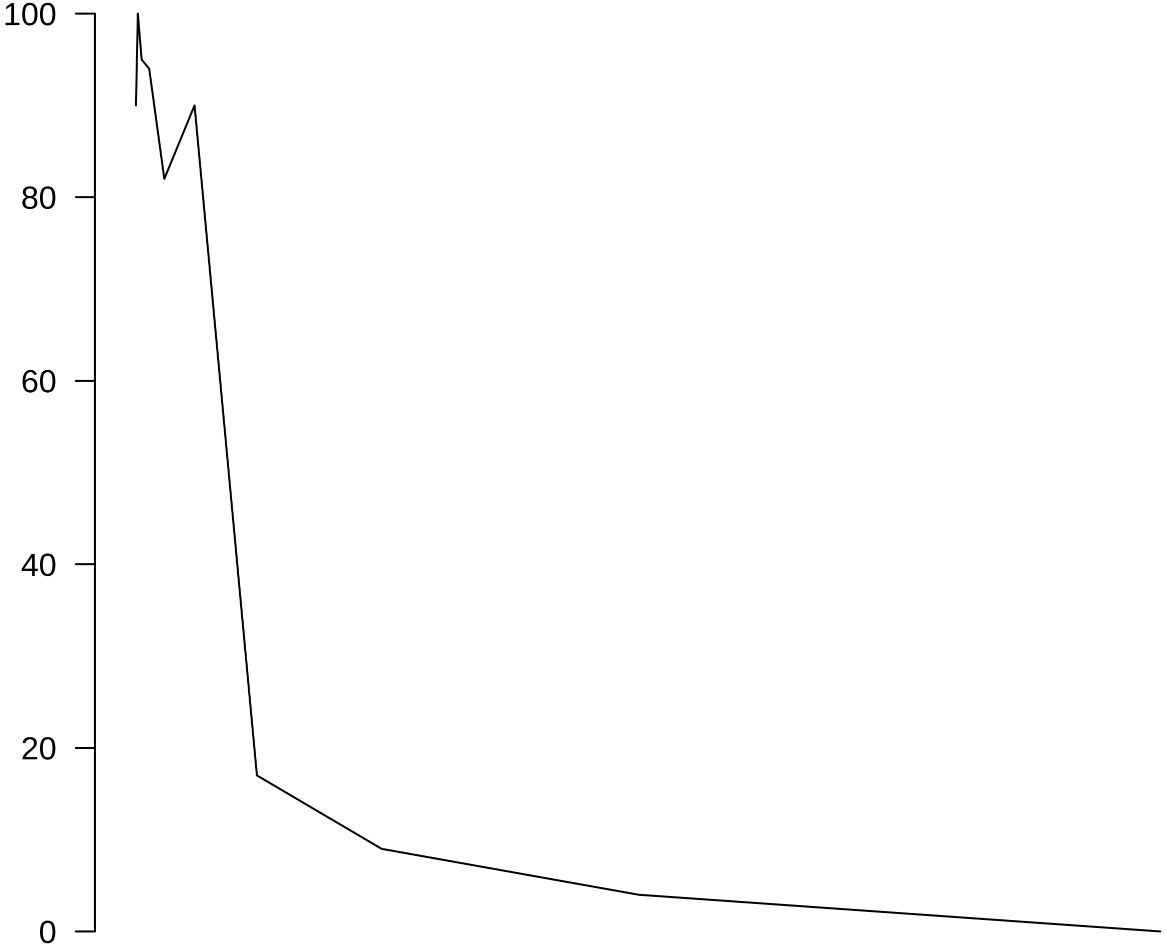

participant was in isolated p−value condition
classified as moderate cliff

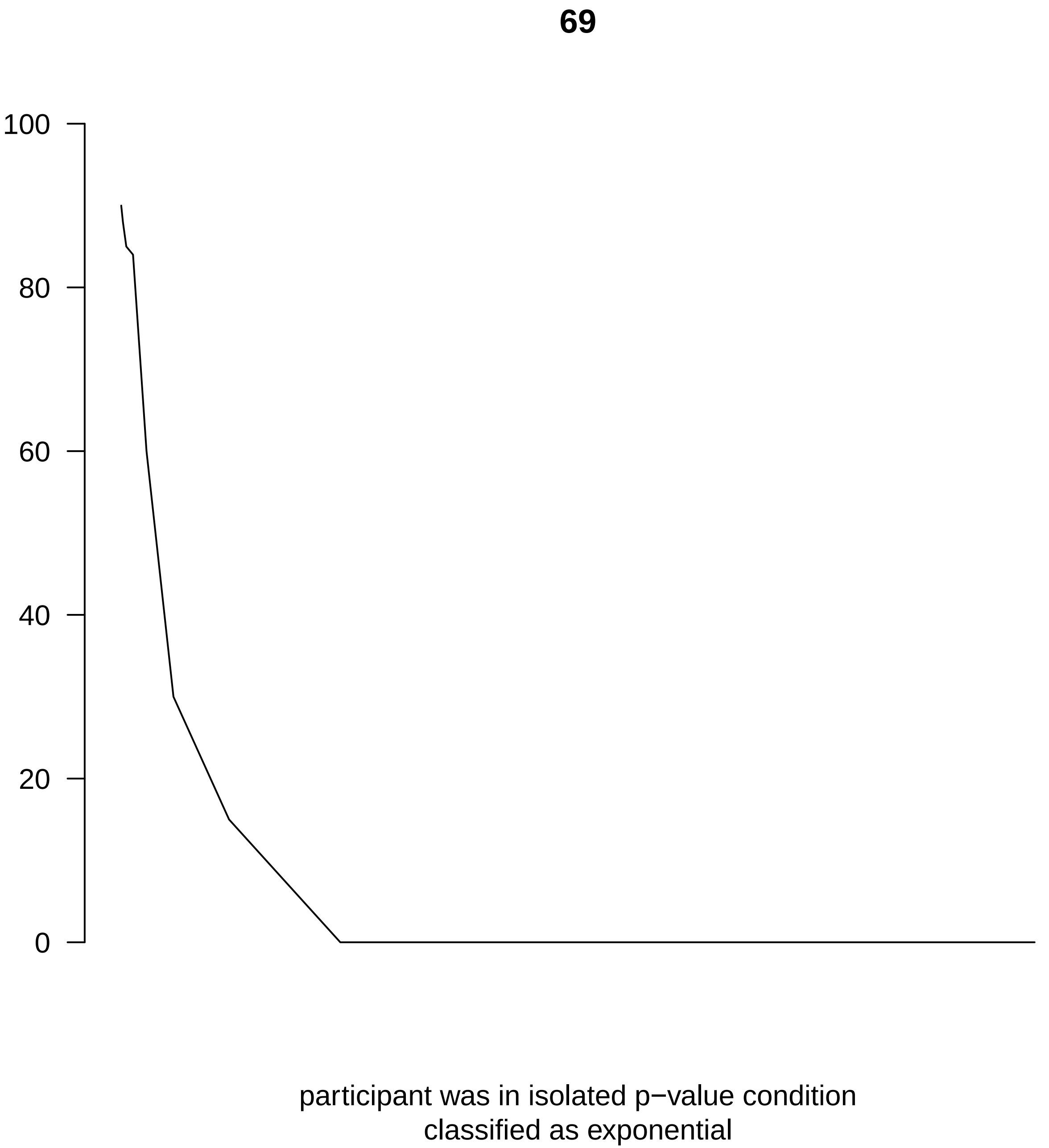
69
100
80
60
40
20
0
participant was in isolated p−value condition
classified as exponential

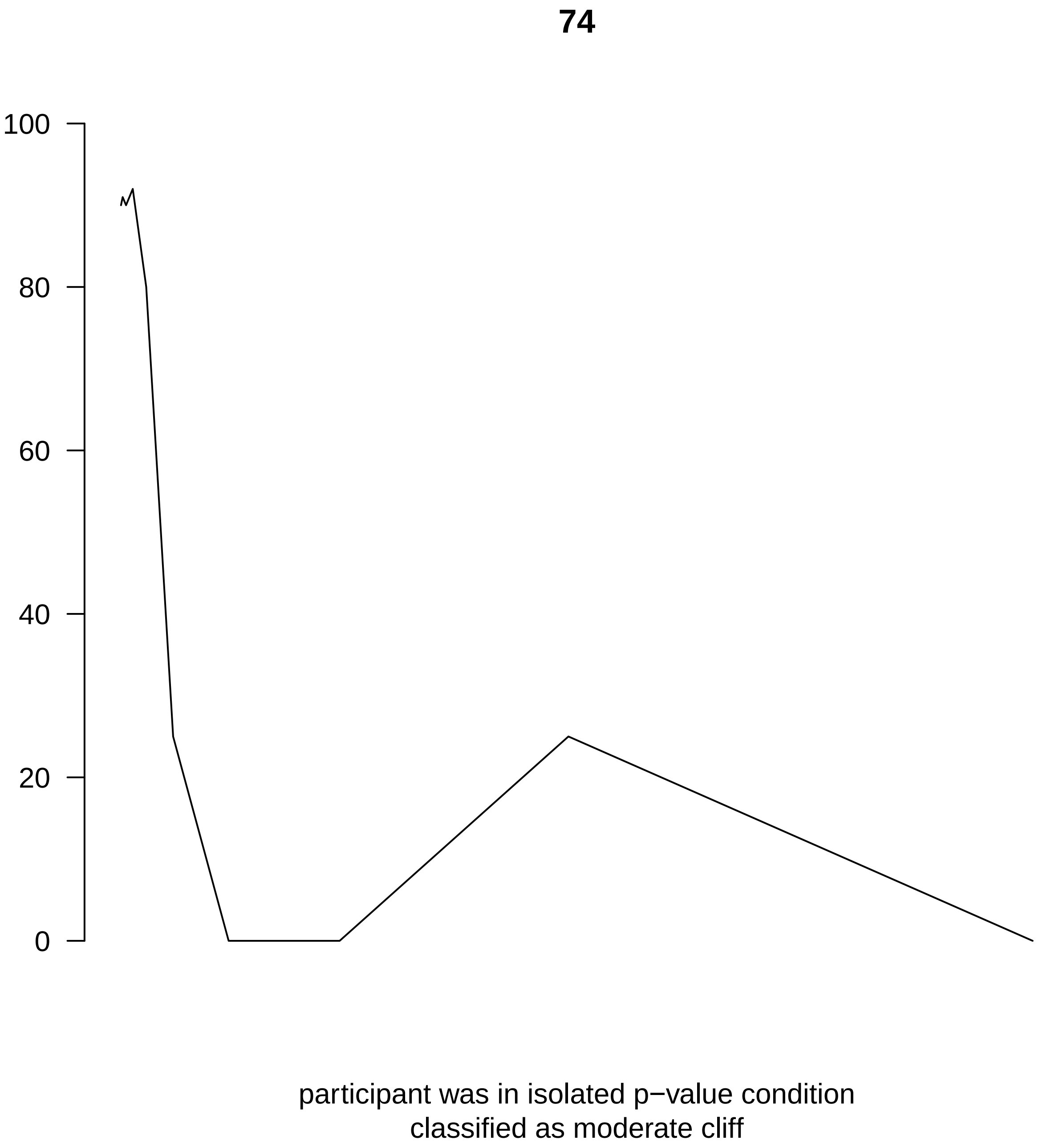
74
100
80
60
40
20
0
participant was in isolated p−value condition
classified as moderate cliff

80

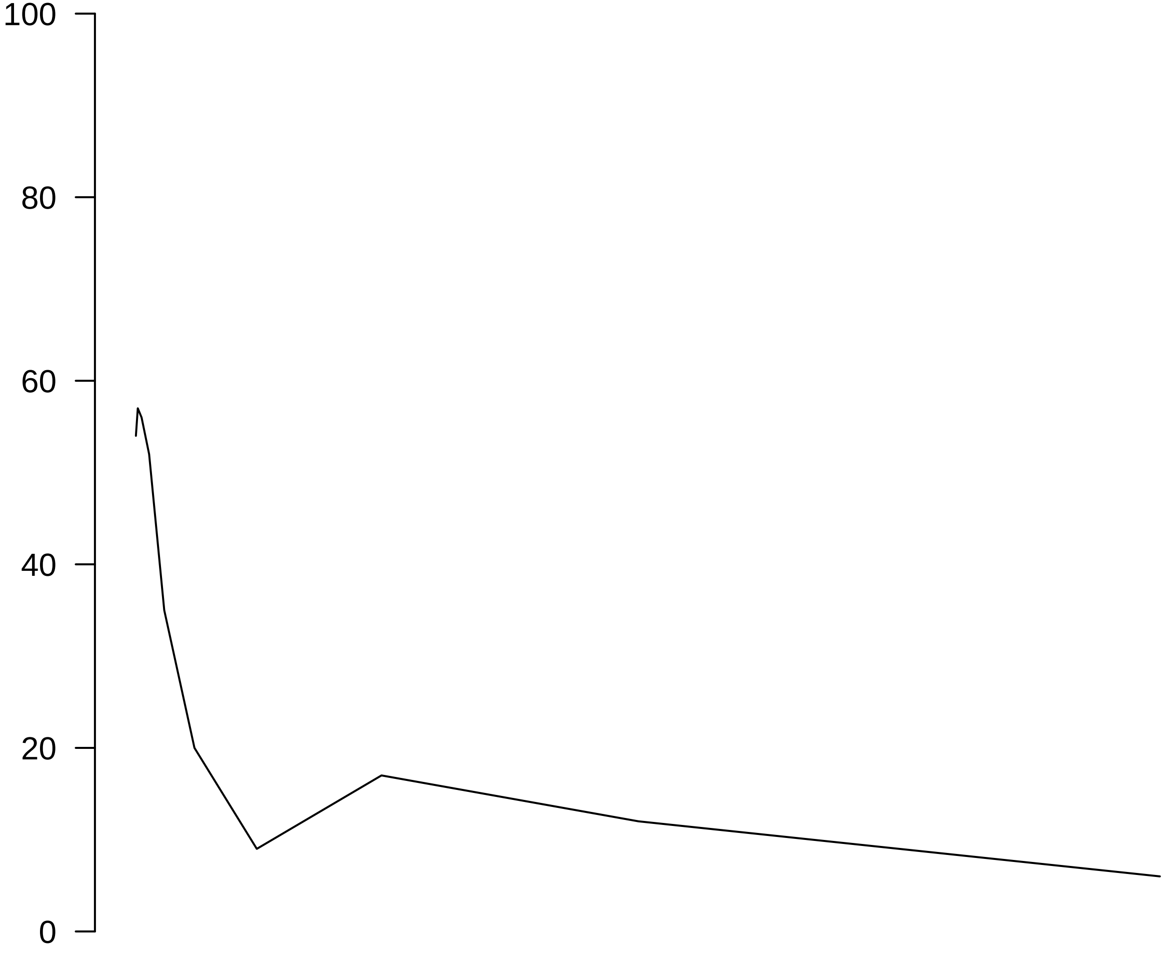

participant was in isolated p−value condition
classified as moderate cliff

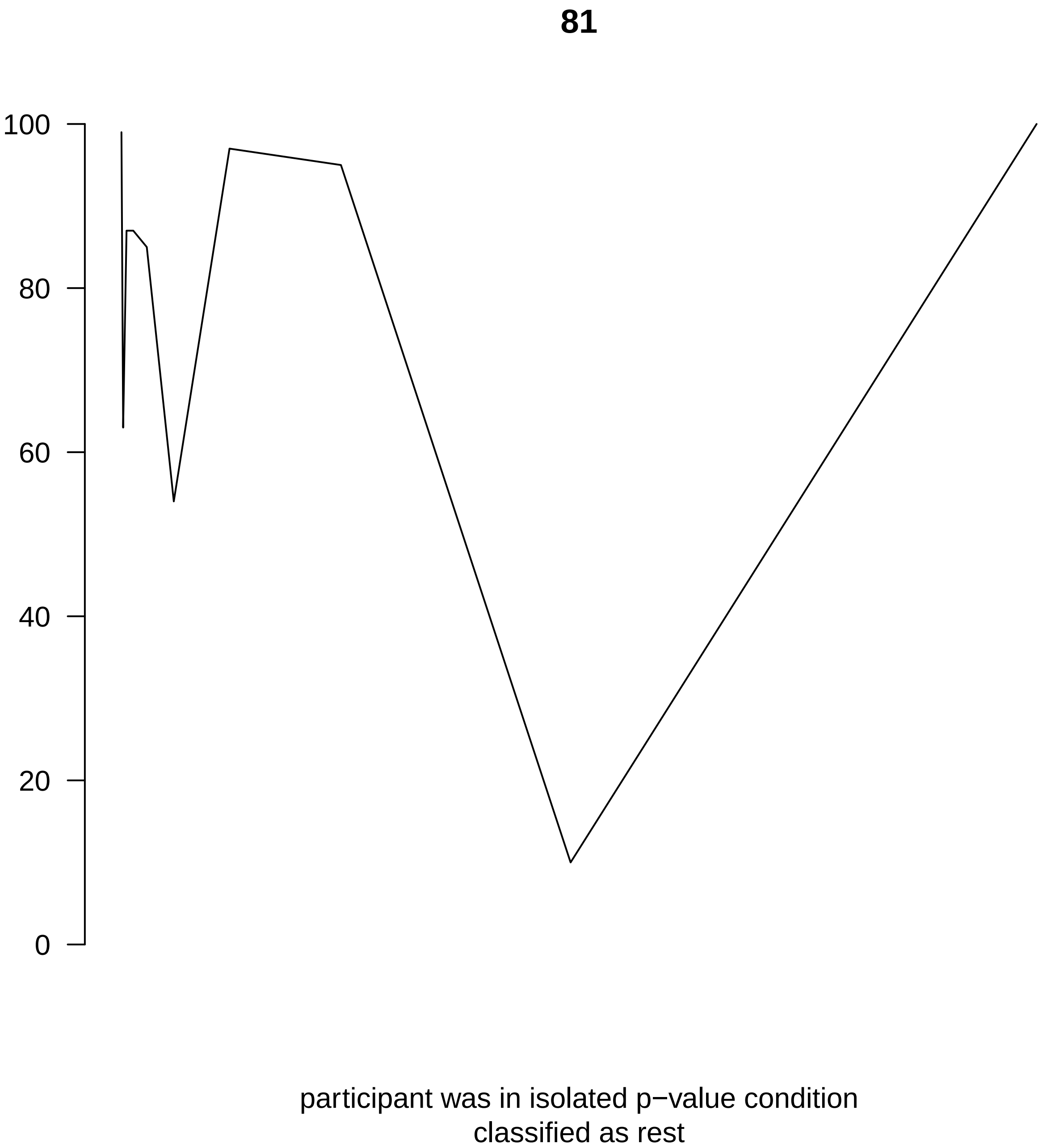
81
100
80
60
40
20
0
participant was in isolated p−value condition
classified as rest

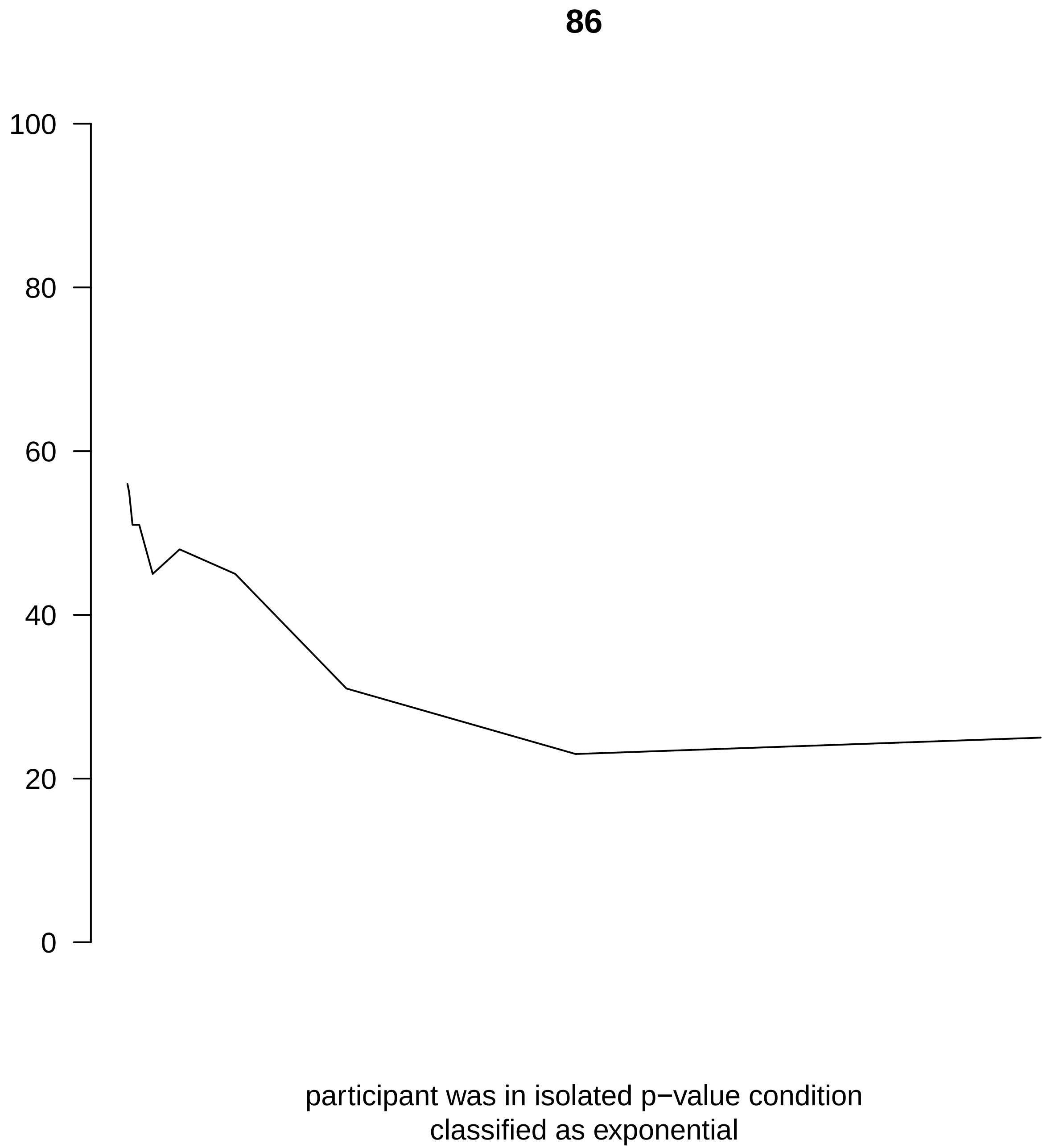
86
100
80
60
40
20
0
participant was in isolated p−value condition
classified as exponential

94

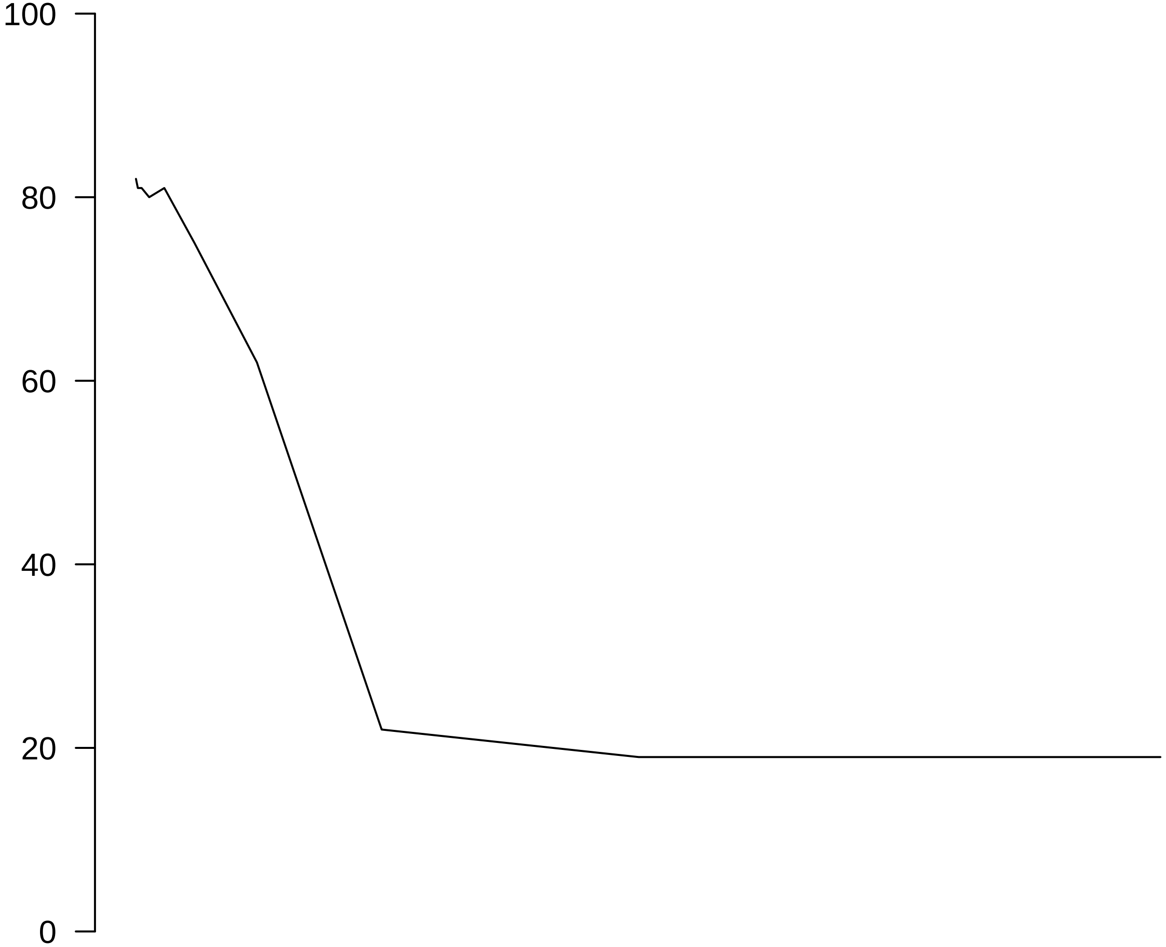

participant was in isolated p−value condition
classified as moderate cliff

96

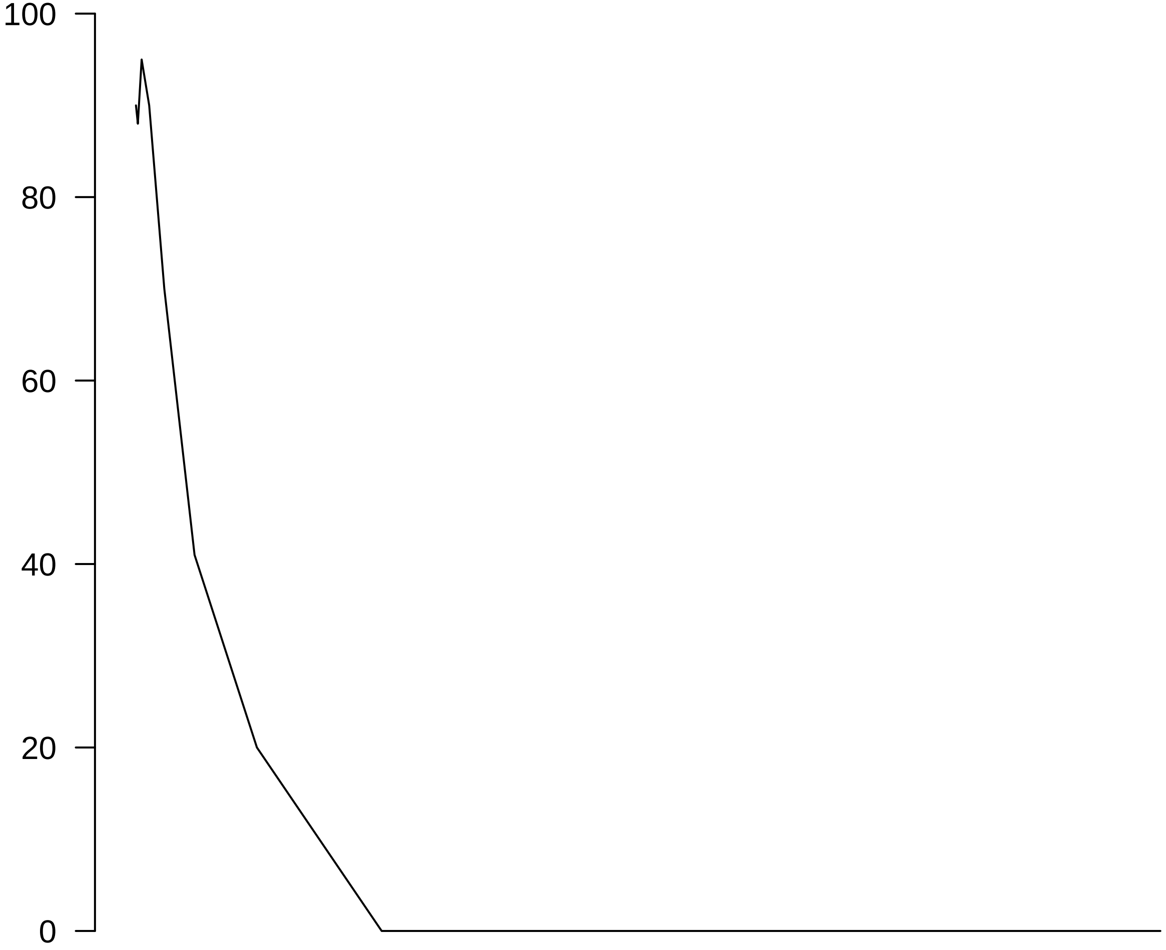

participant was in isolated p−value condition
classified as moderate cliff

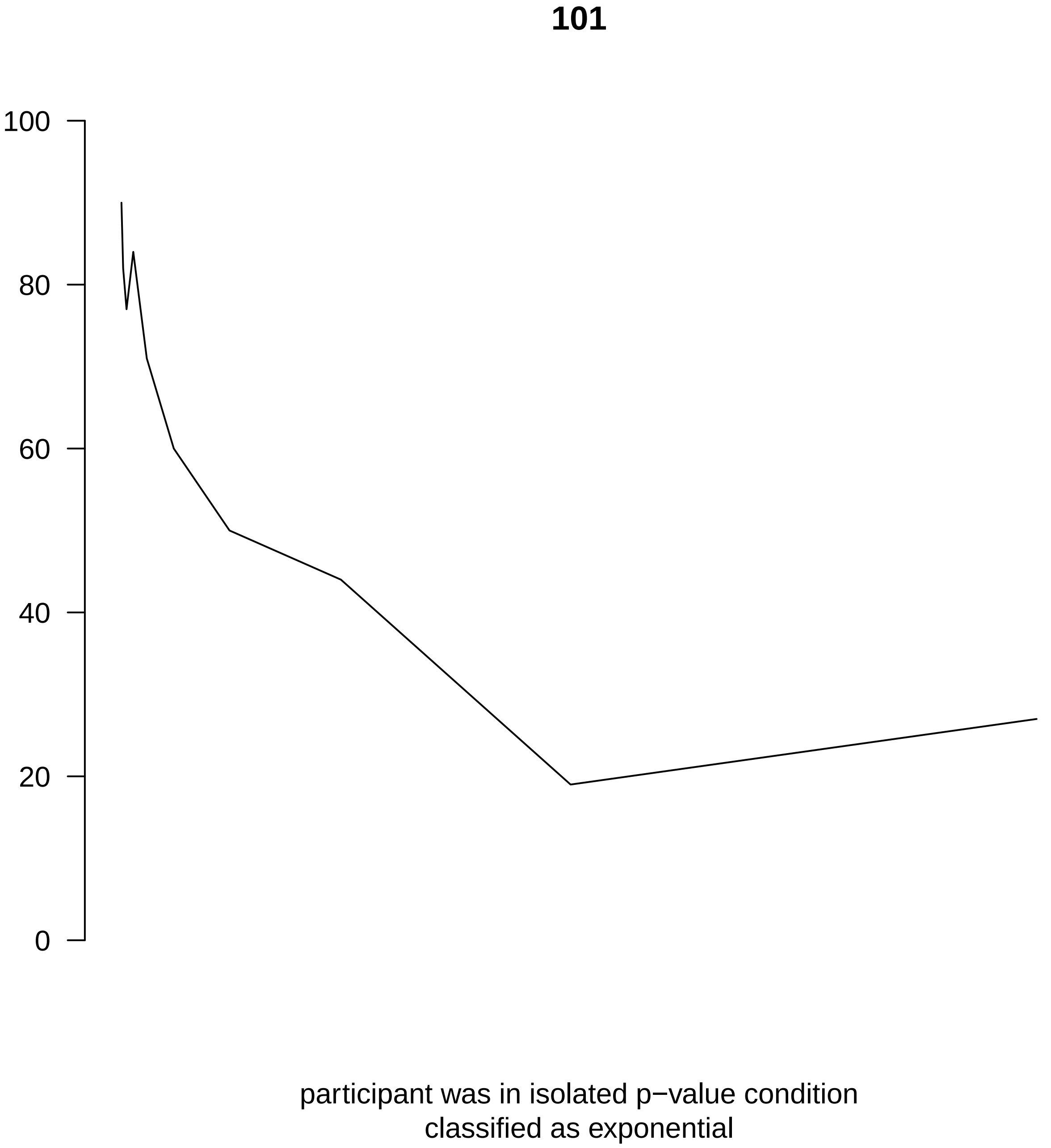
101
100
80
60
40
20
0
participant was in isolated p−value condition
classified as exponential

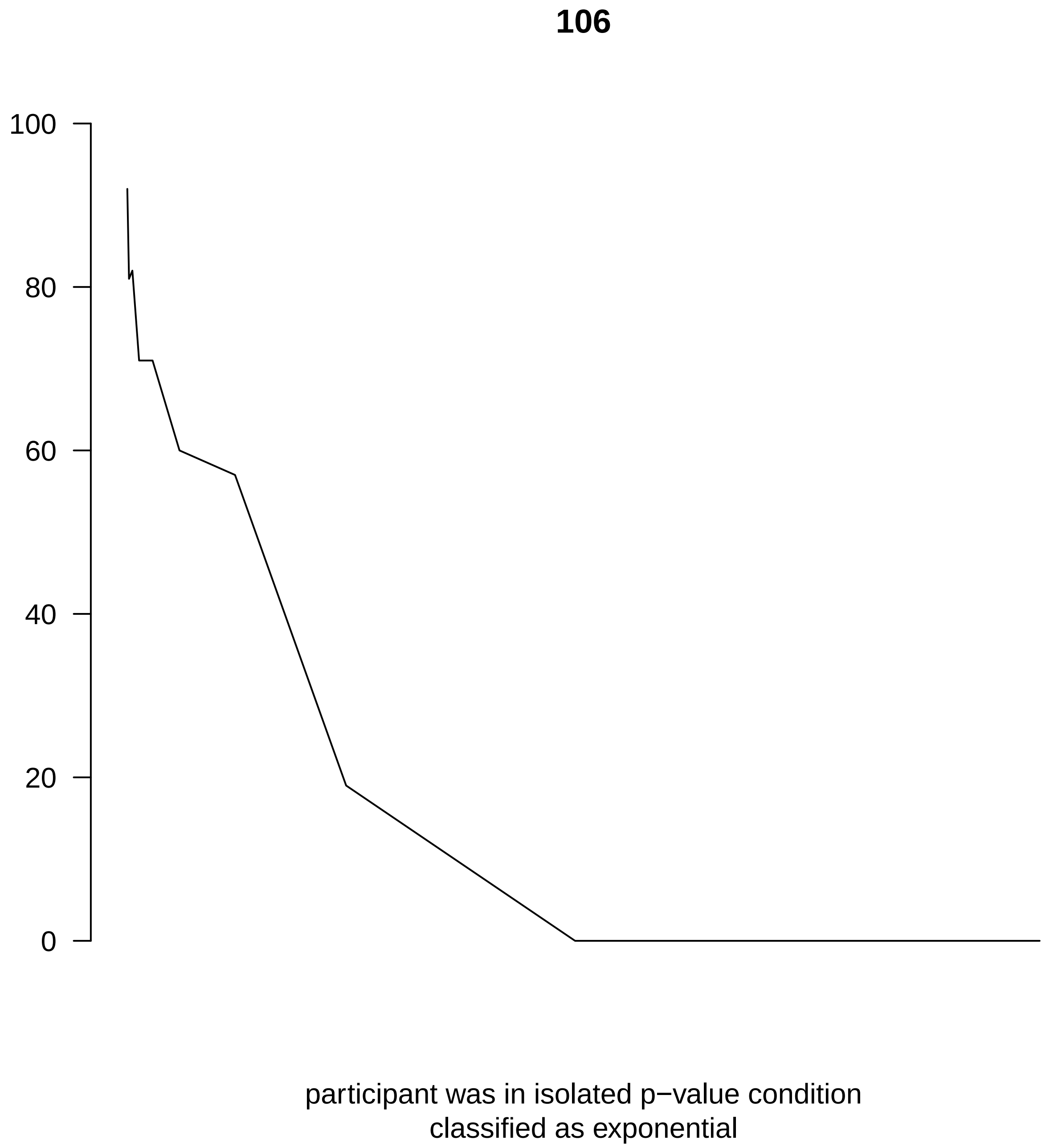
106
100
80
60
40
20
0
participant was in isolated p−value condition
classified as exponential

109

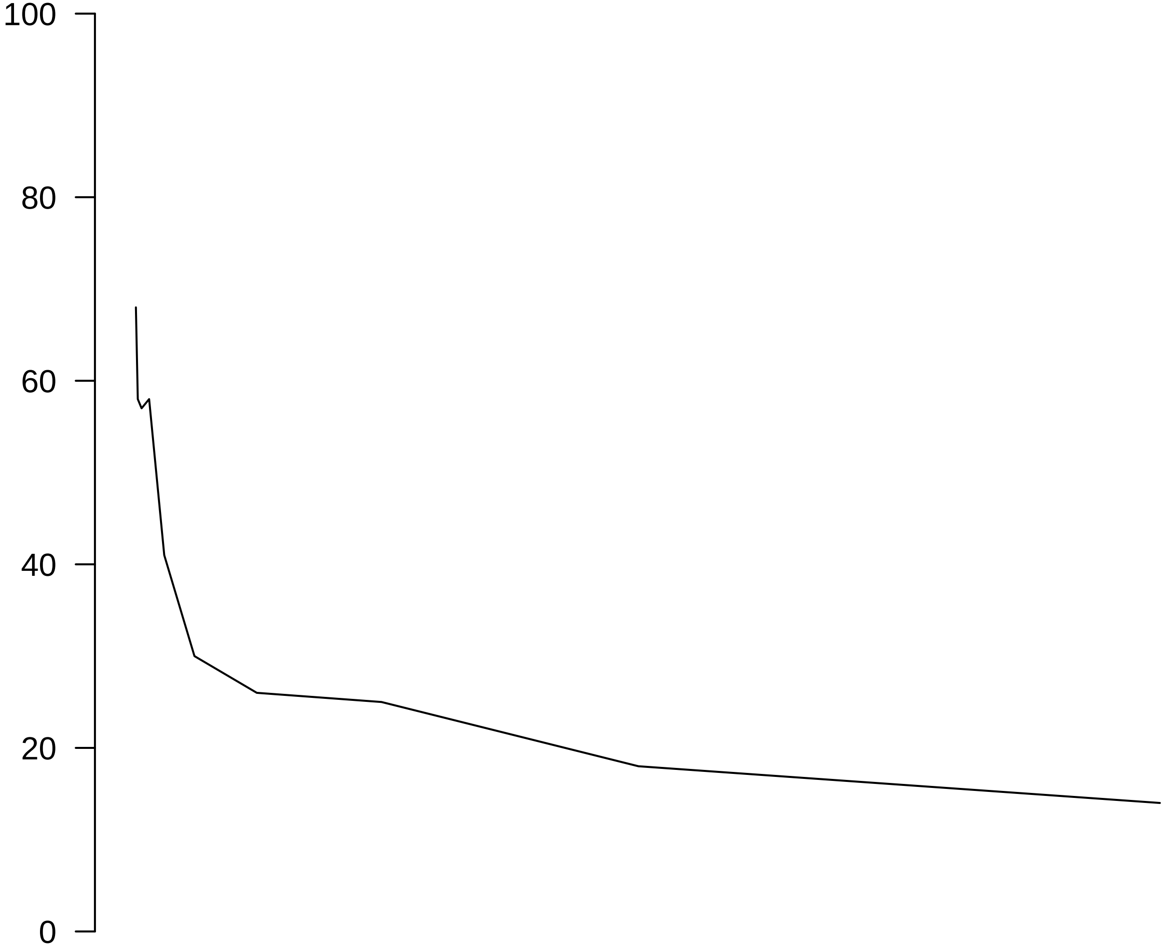

participant was in isolated p−value condition
classified as exponential

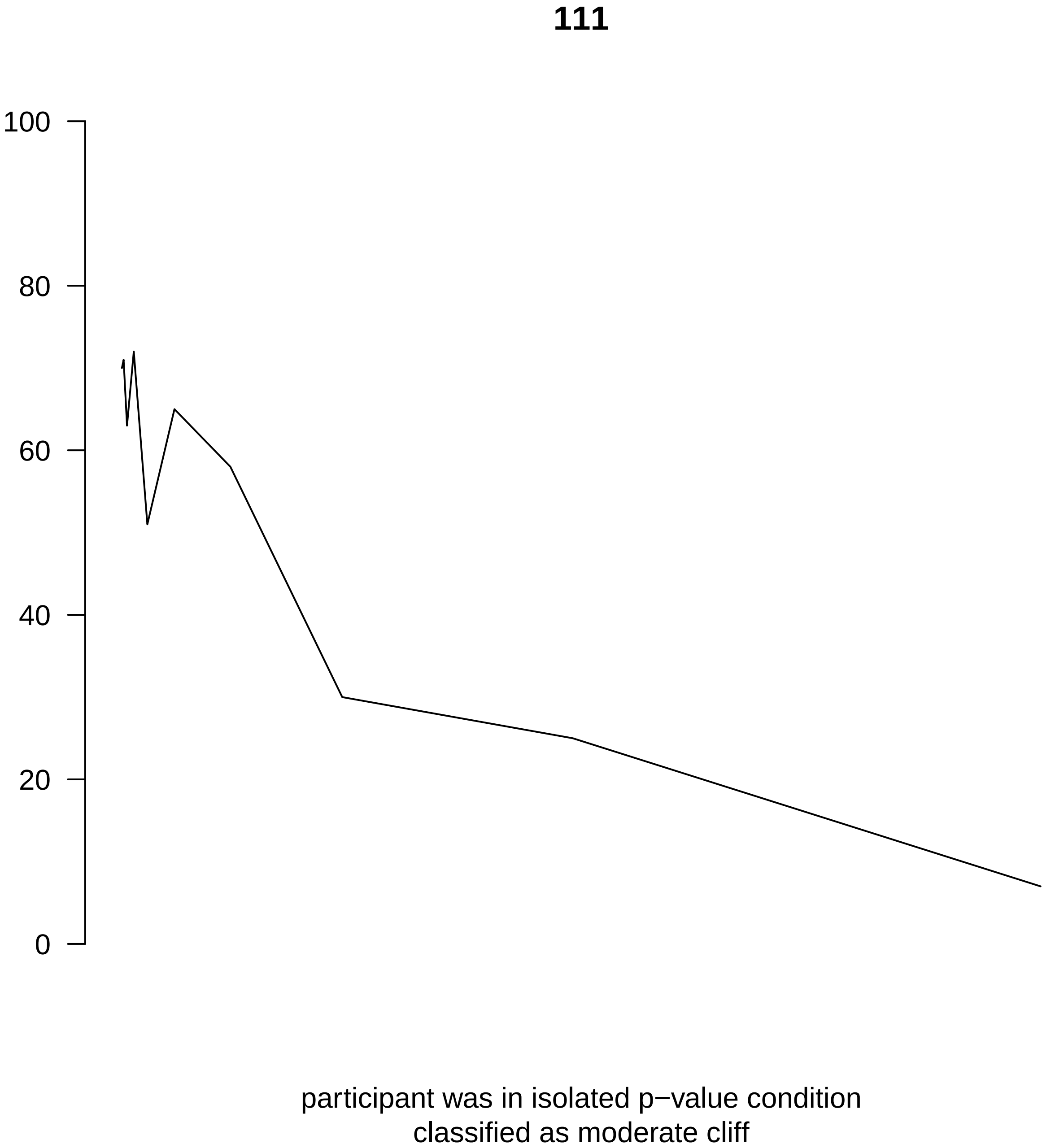
111
100
80
60
40
20
0
participant was in isolated p−value condition
classified as moderate cliff

117

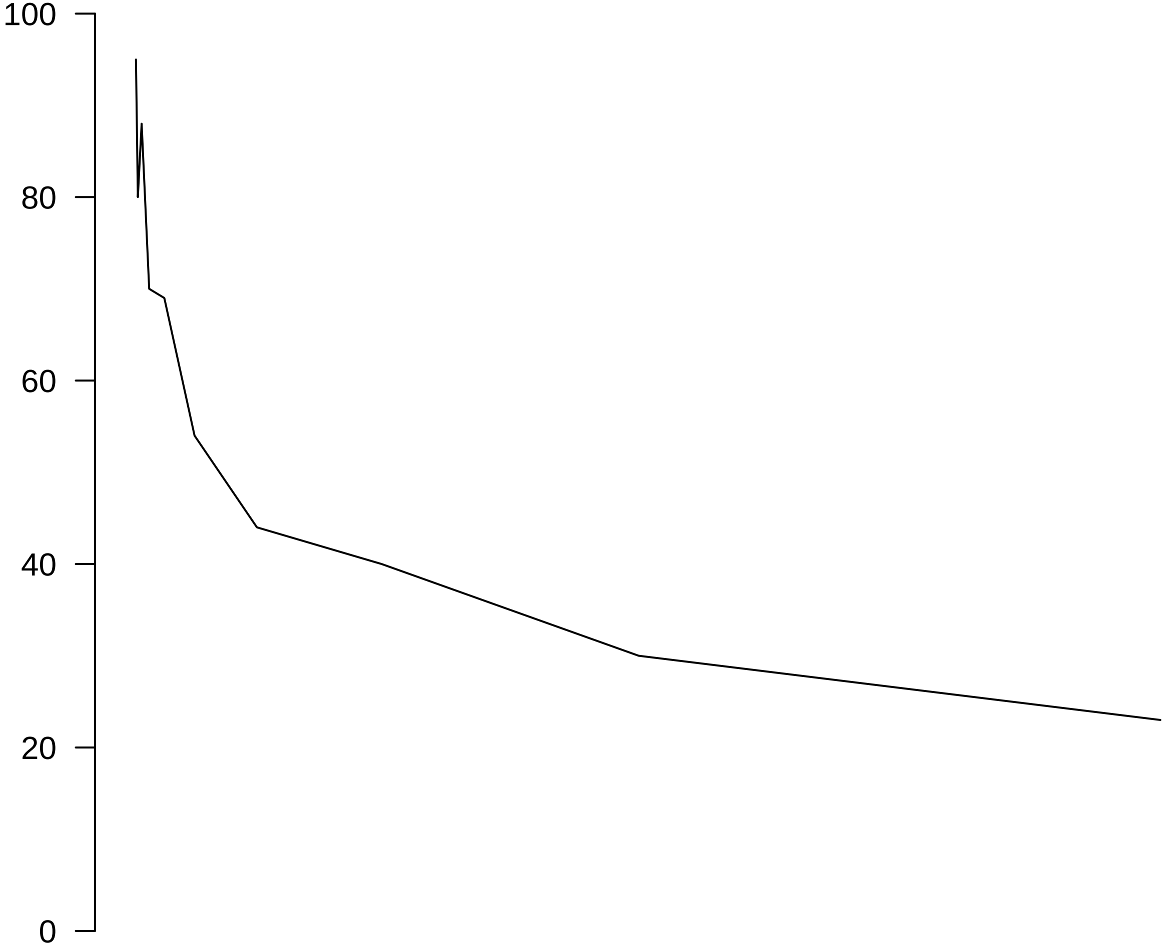

participant was in isolated p−value condition
classified as exponential

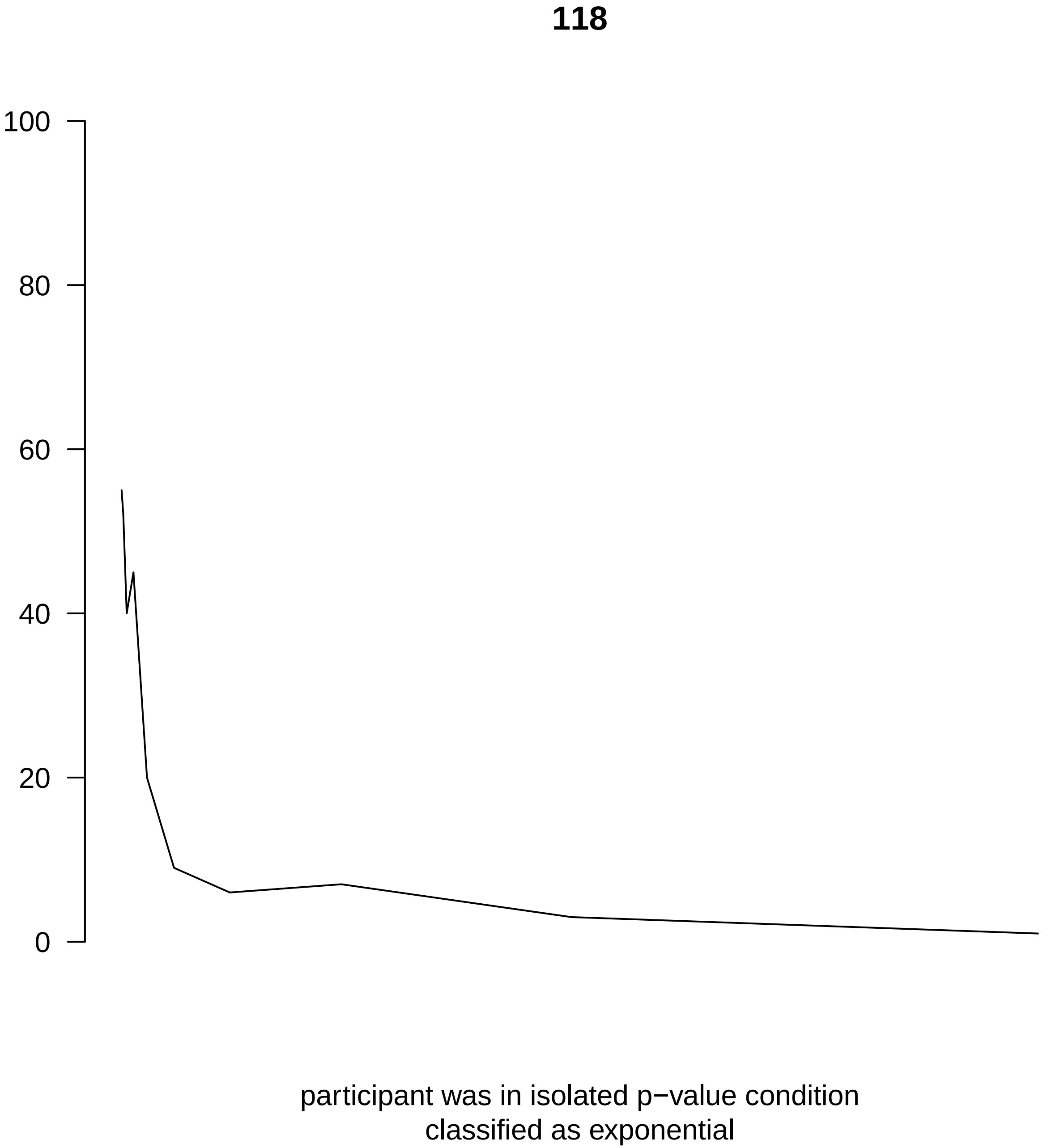
118
100
80
60
40
20
0
participant was in isolated p−value condition
classified as exponential

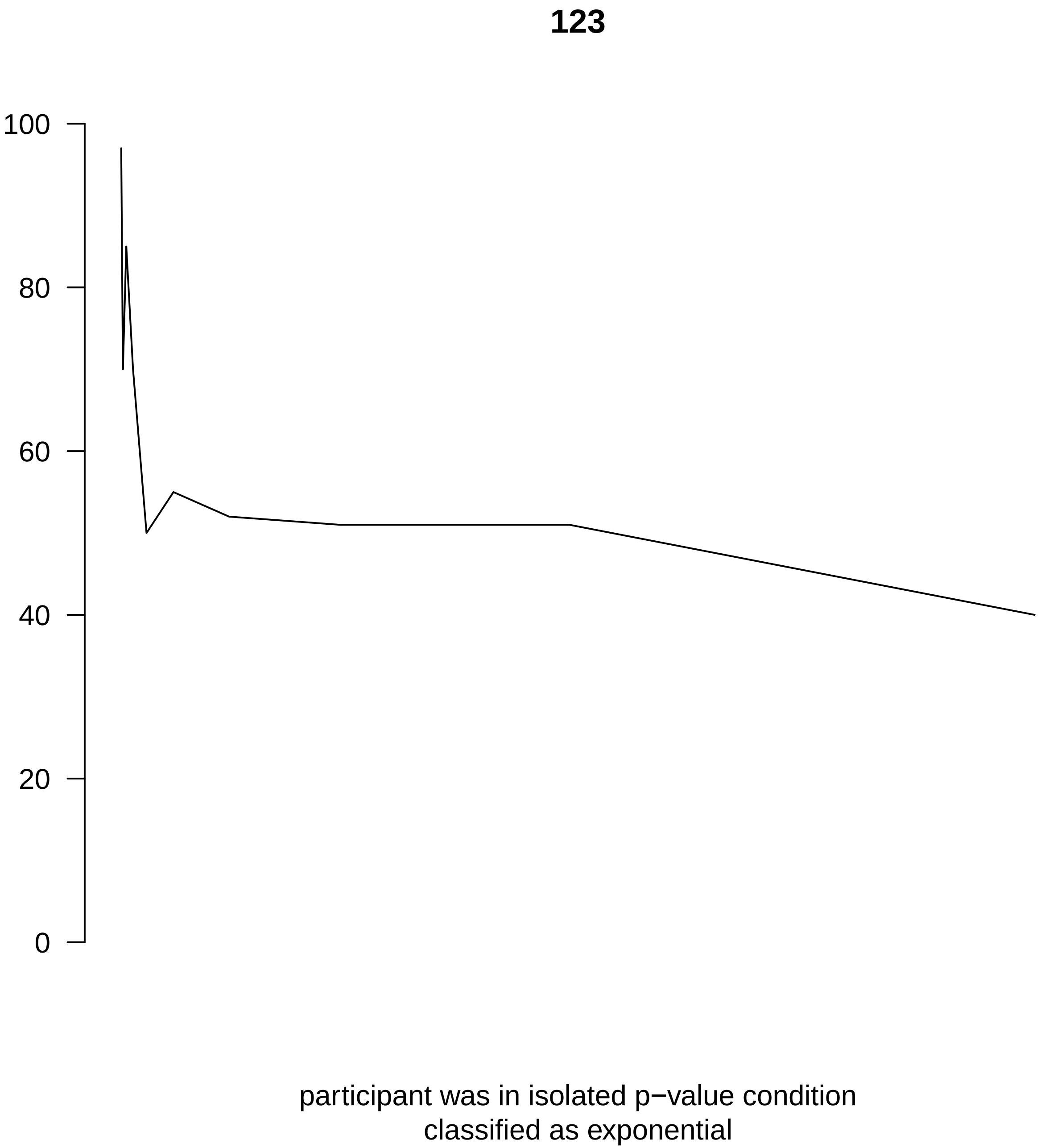
123
100
80
60
40
20
0
participant was in isolated p−value condition
classified as exponential

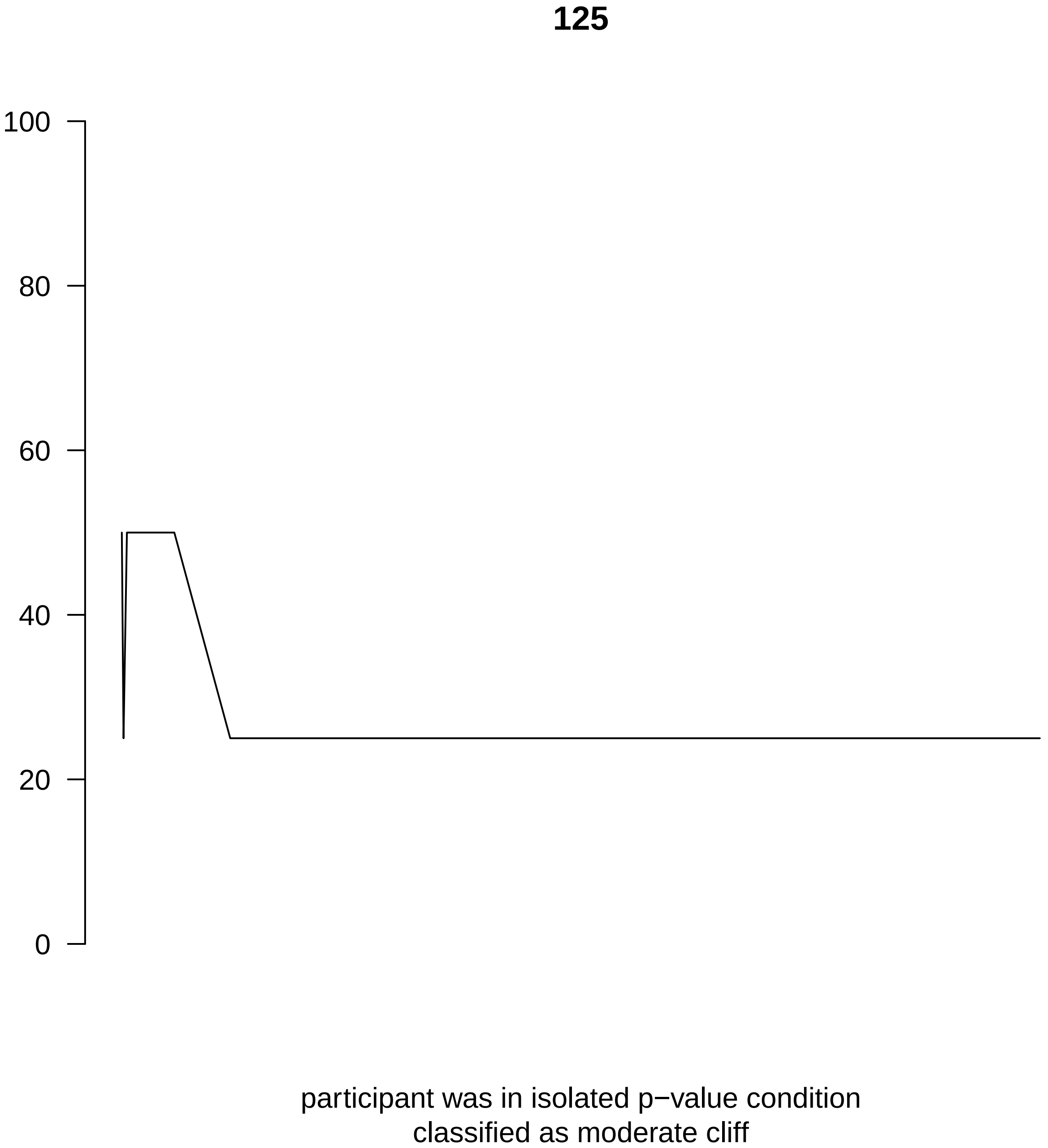
125
100
80
60
40
20
0
participant was in isolated p−value condition
classified as moderate cliff

133

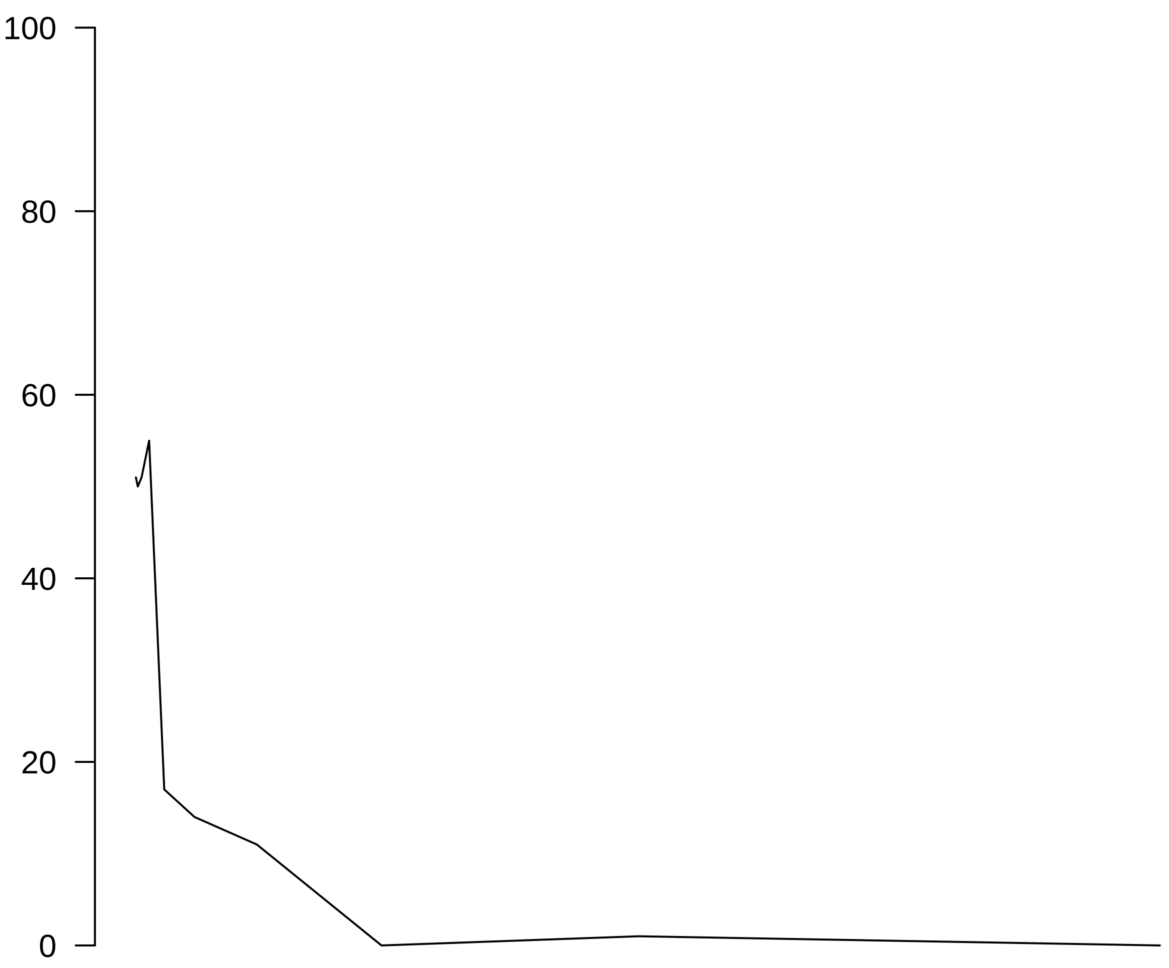

participant was in isolated p−value condition
classified as moderate cliff

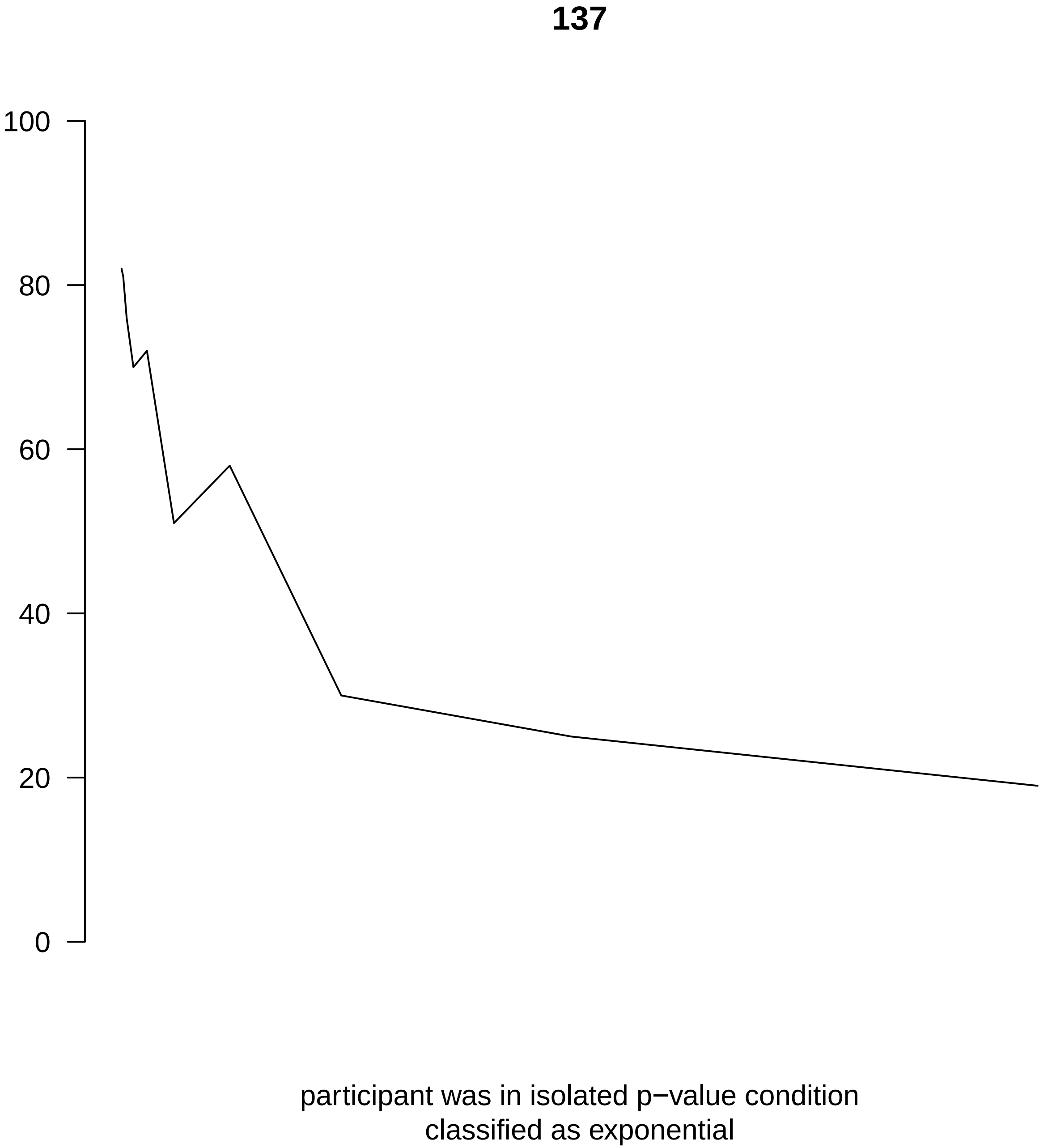
137
100
80
60
40
20
0
participant was in isolated p−value condition
classified as exponential

1

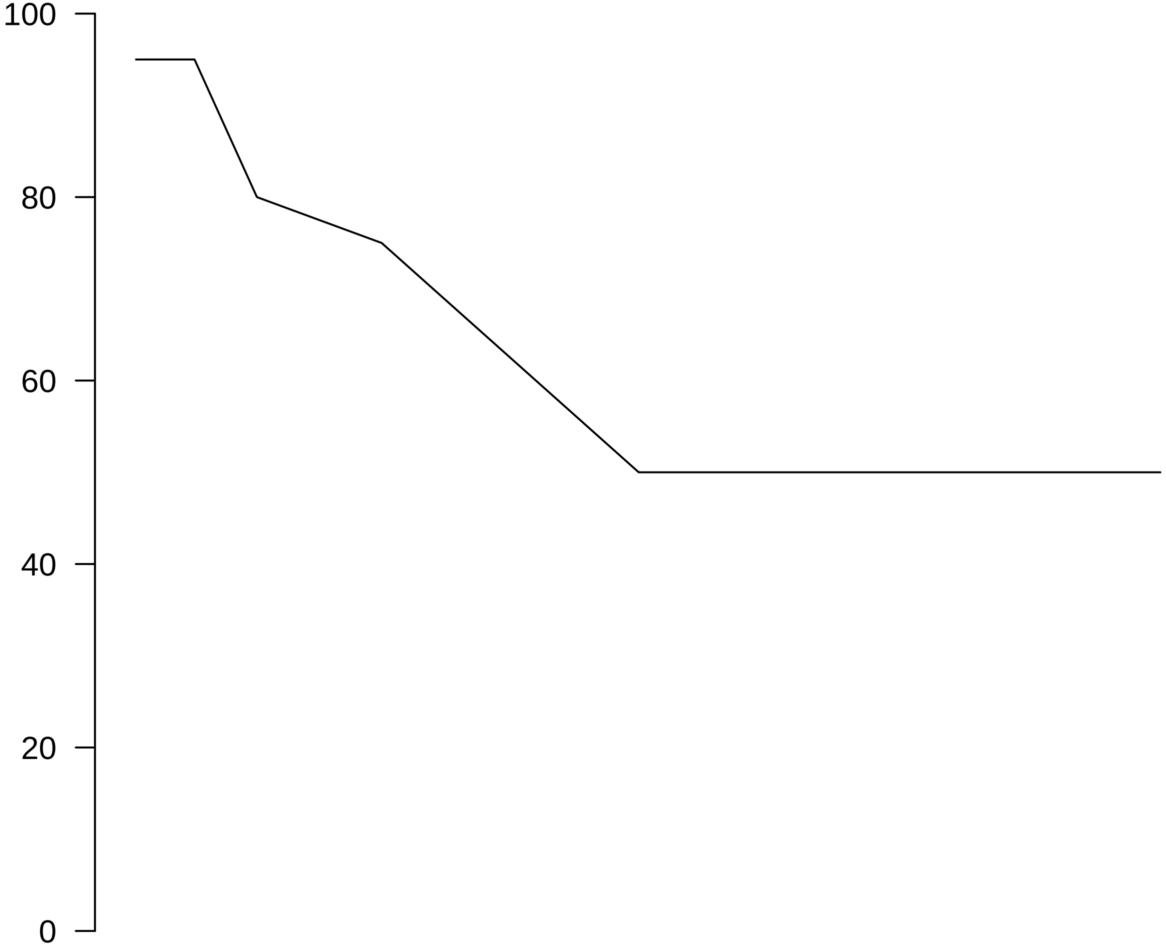

participant was in all at once p−value condition
classified as moderate cliff

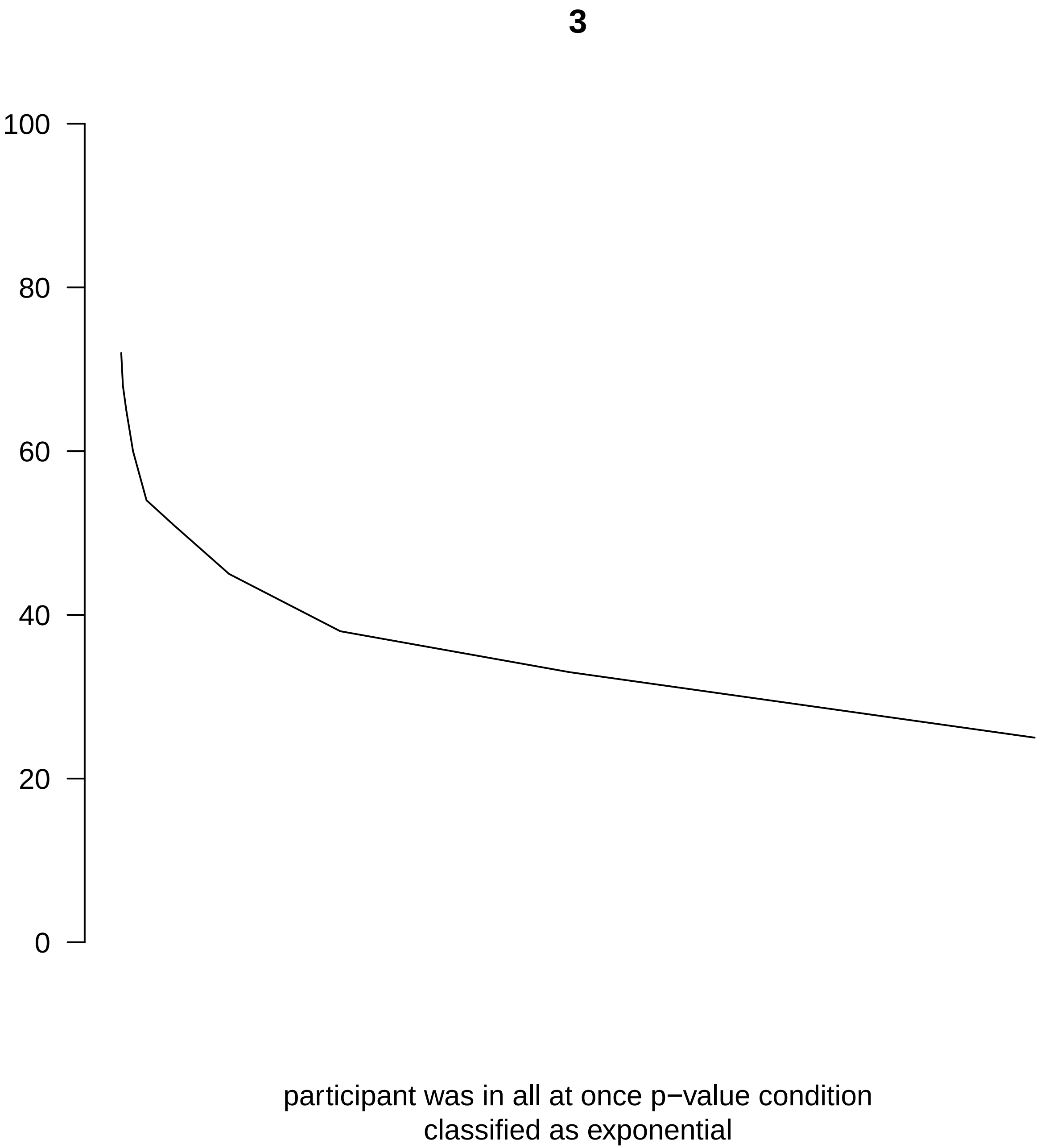
3
100
80
60
40
20
0
participant was in all at once p−value condition
classified as exponential

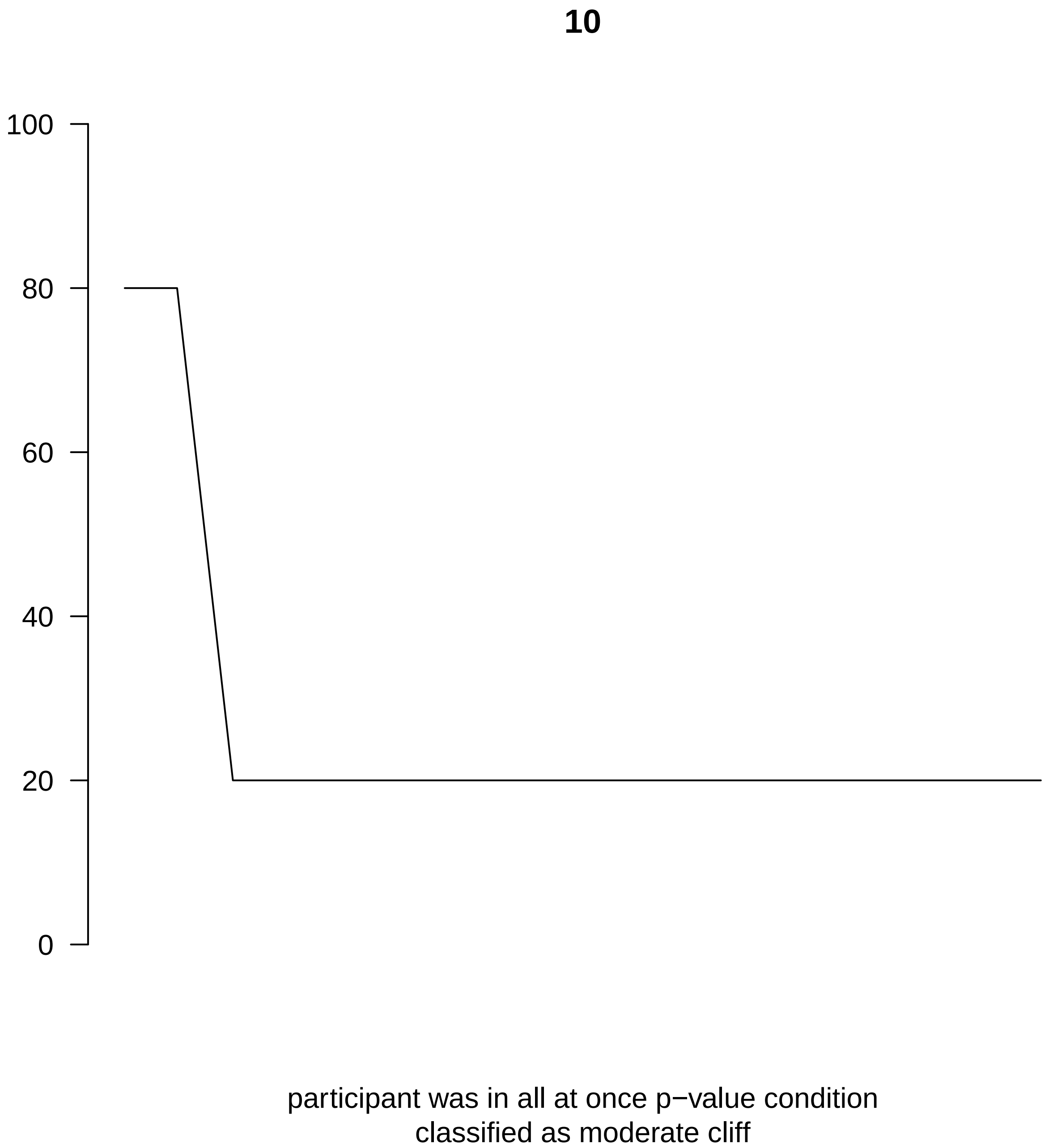
10
100
80
60
40
20
0
participant was in all at once p−value condition
classified as moderate cliff

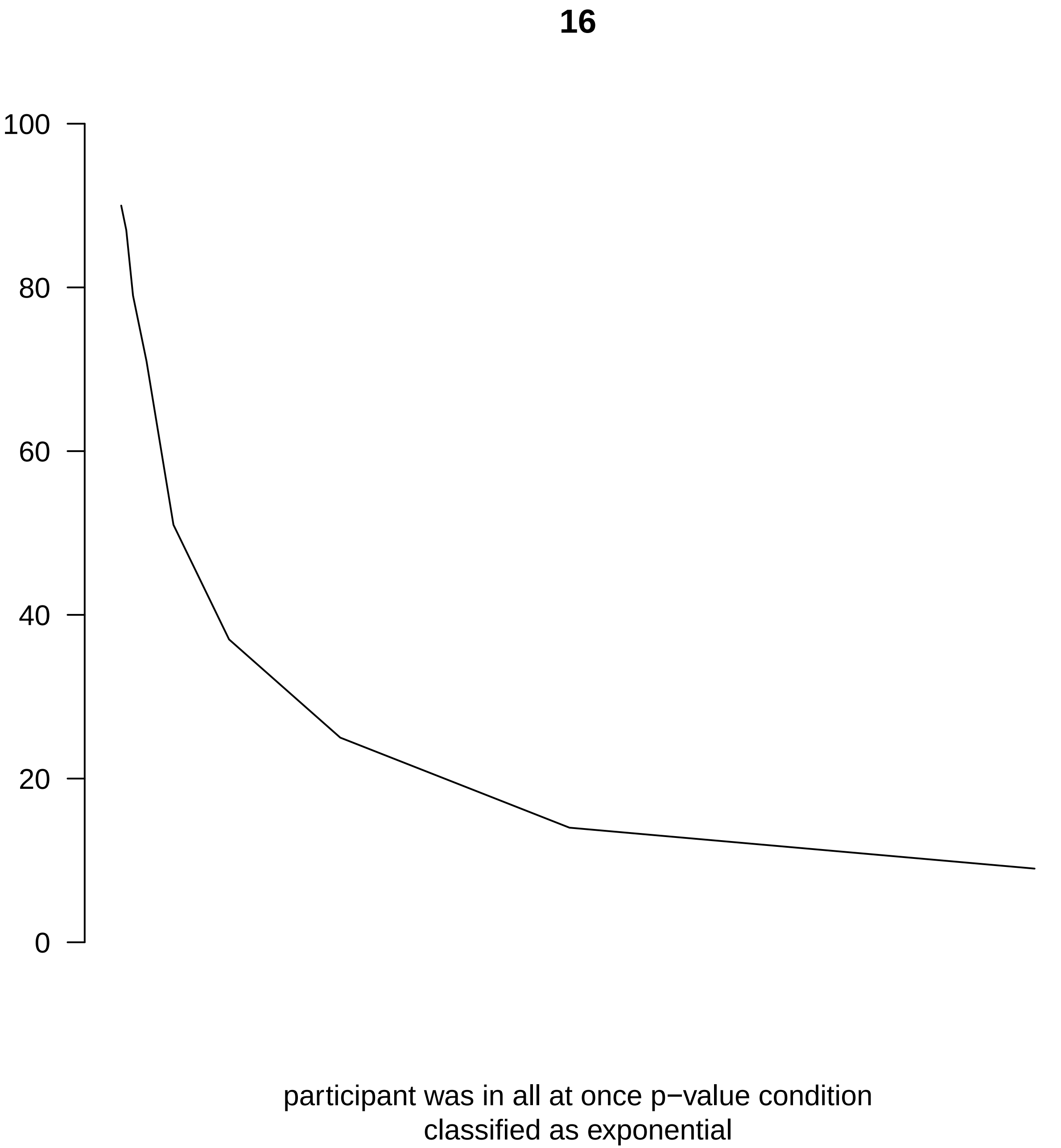
16
100
80
60
40
20
0
participant was in all at once p−value condition
classified as exponential

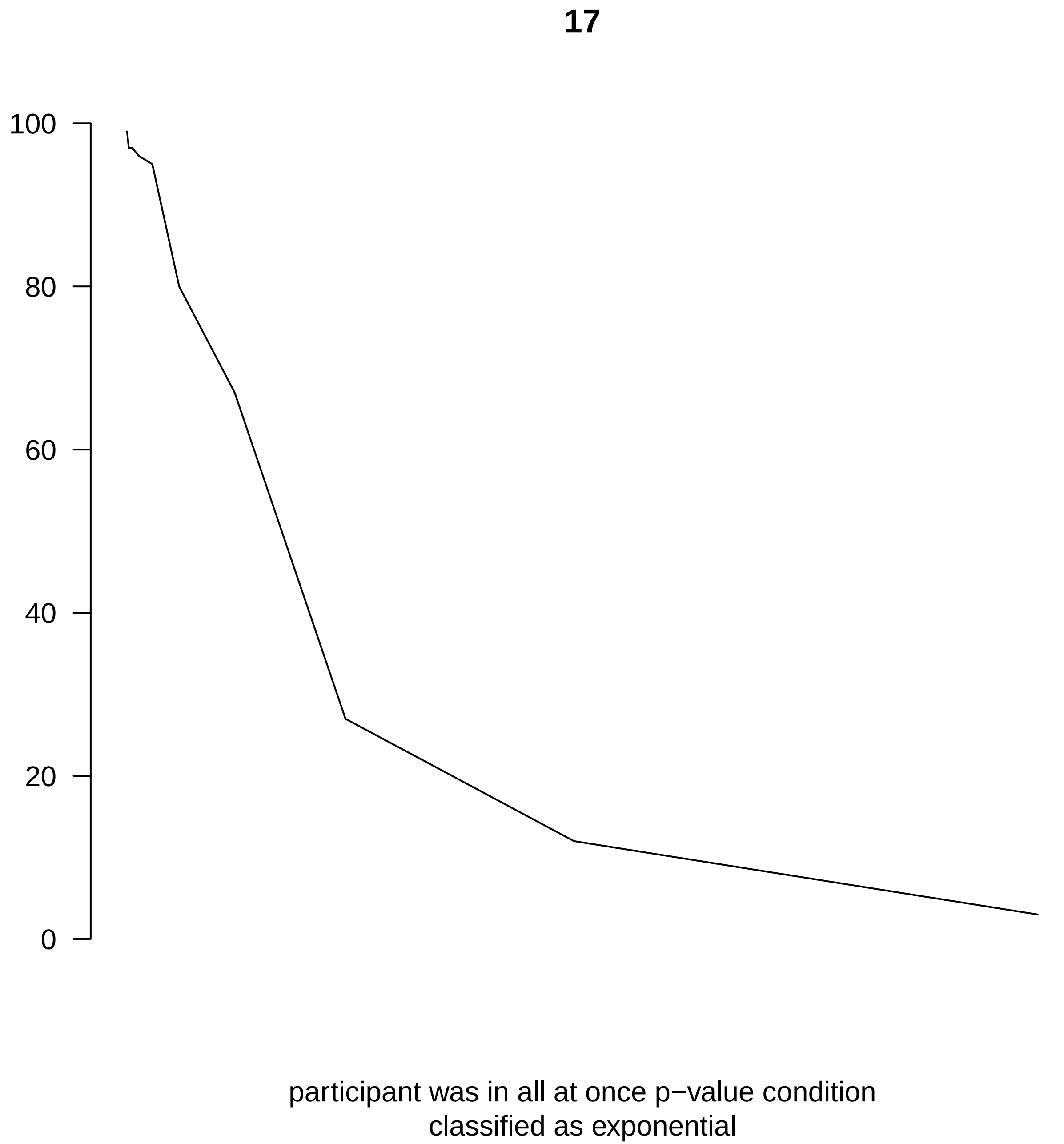
17
100
80
60
40
20
0
participant was in all at once p−value condition
classified as exponential

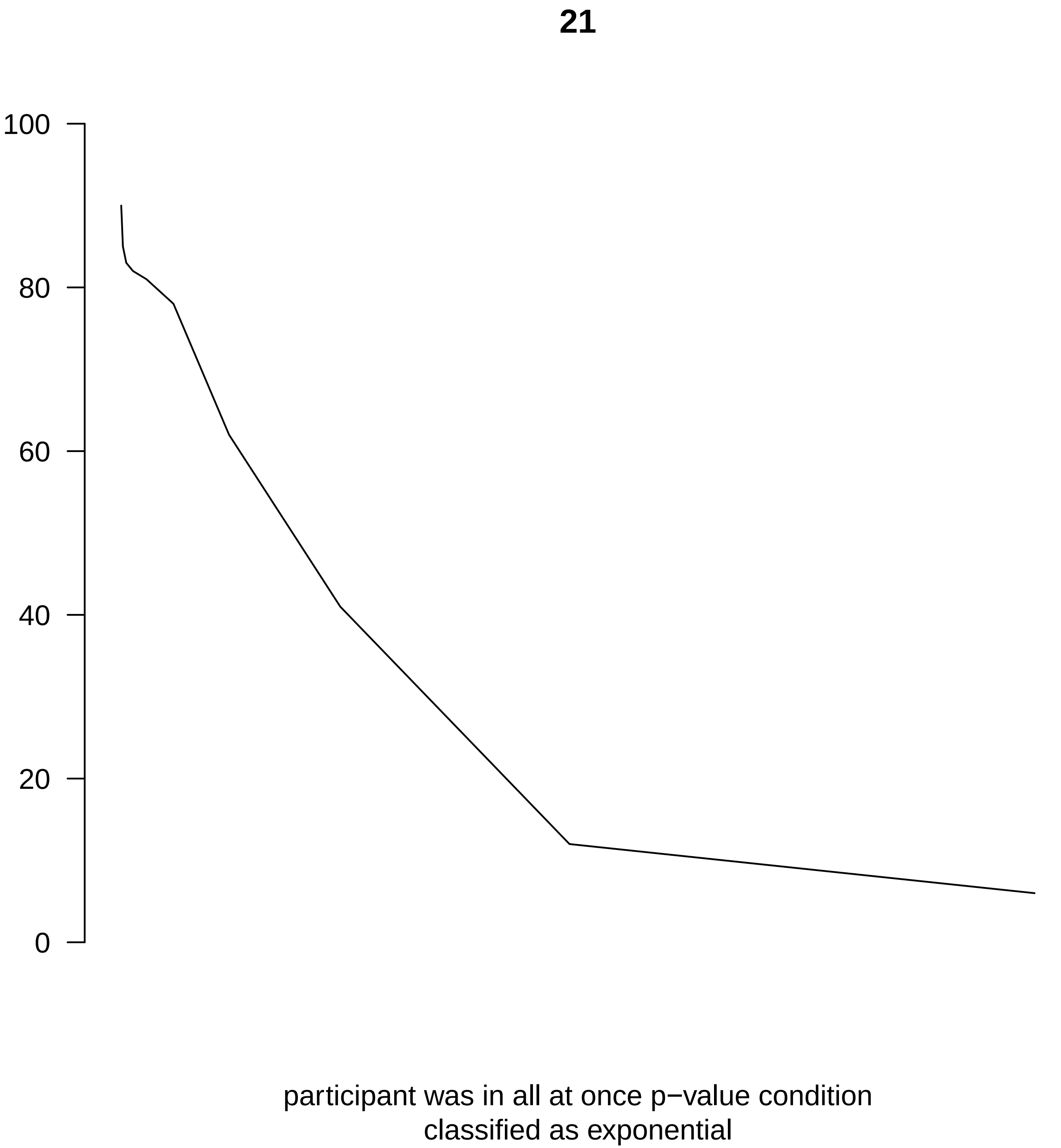
21
100
80
60
40
20
0
participant was in all at once p−value condition
classified as exponential

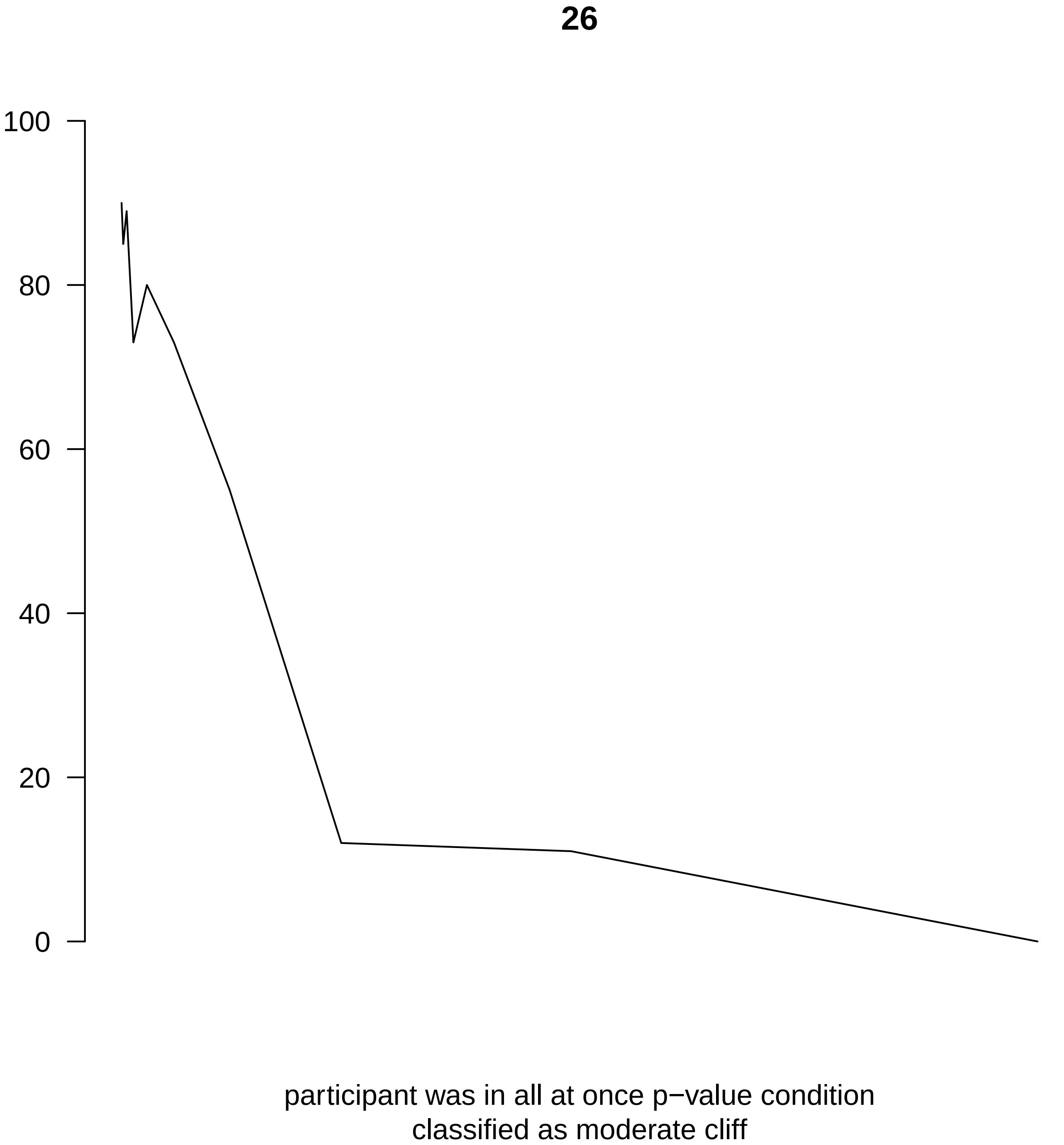
26
100
80
60
40
20
0
participant was in all at once p−value condition
classified as moderate cliff

27

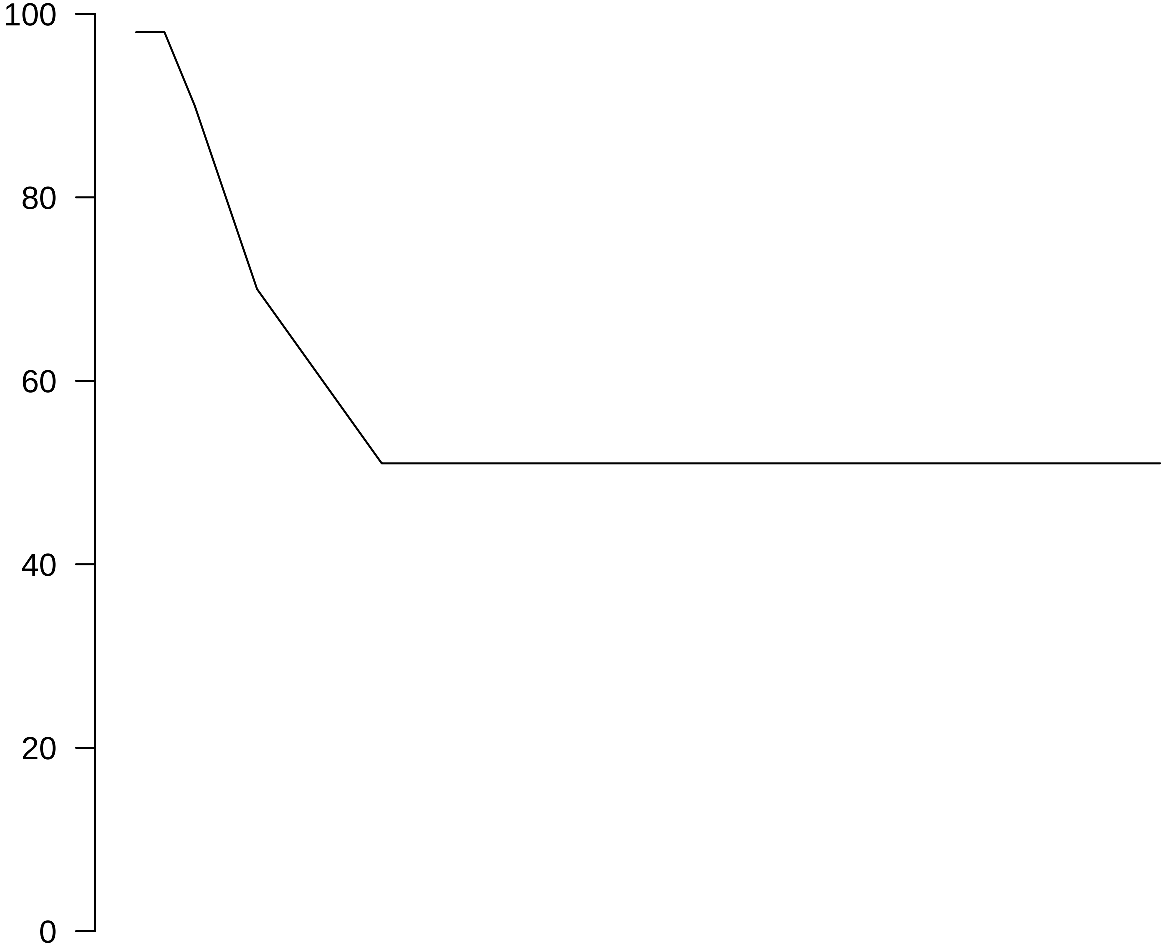

participant was in all at once p−value condition
classified as moderate cliff

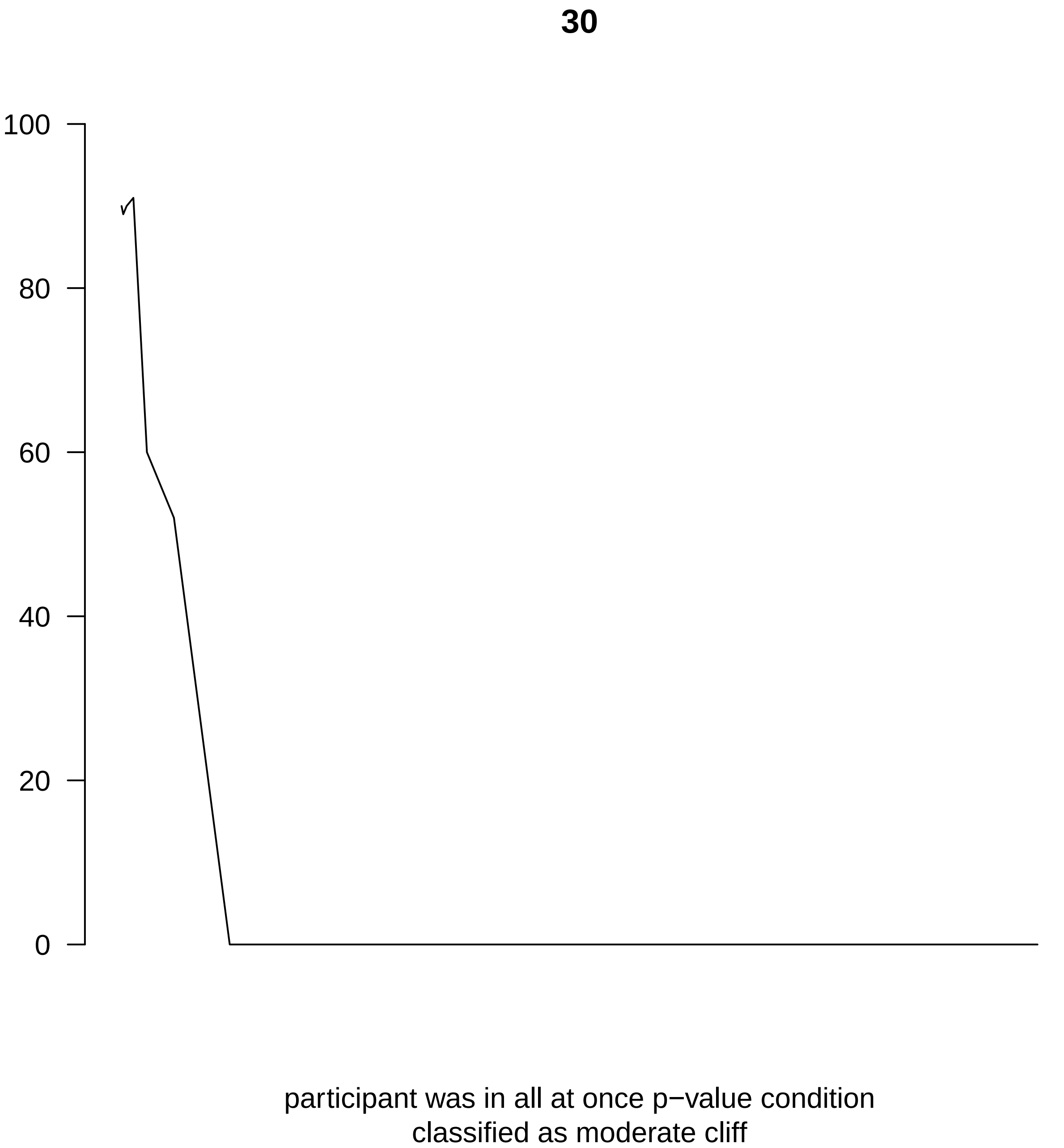
30
100
80
60
40
20
0
participant was in all at once p−value condition
classified as moderate cliff

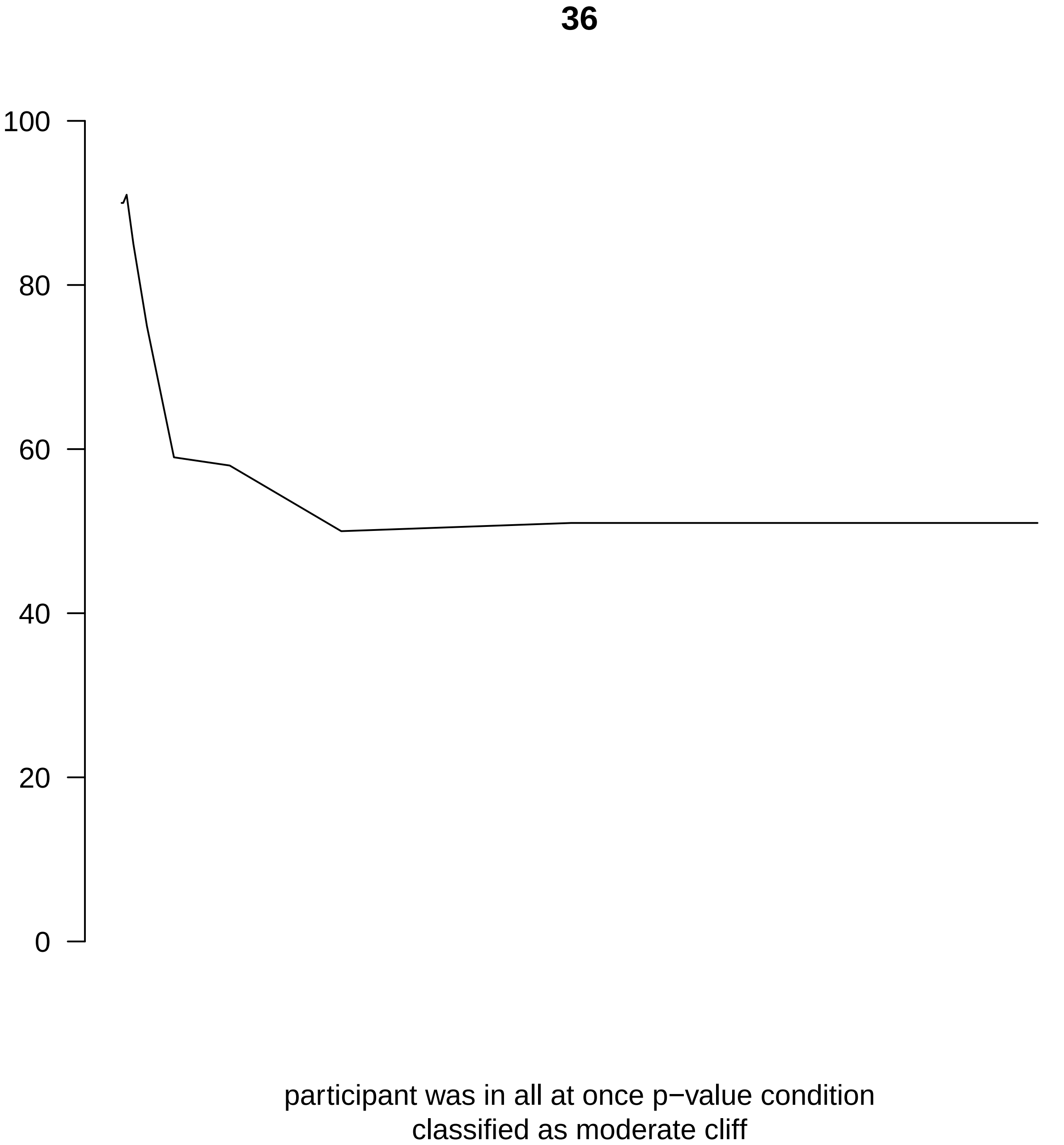
36
100
80
60
40
20
0
participant was in all at once p−value condition
classified as moderate cliff

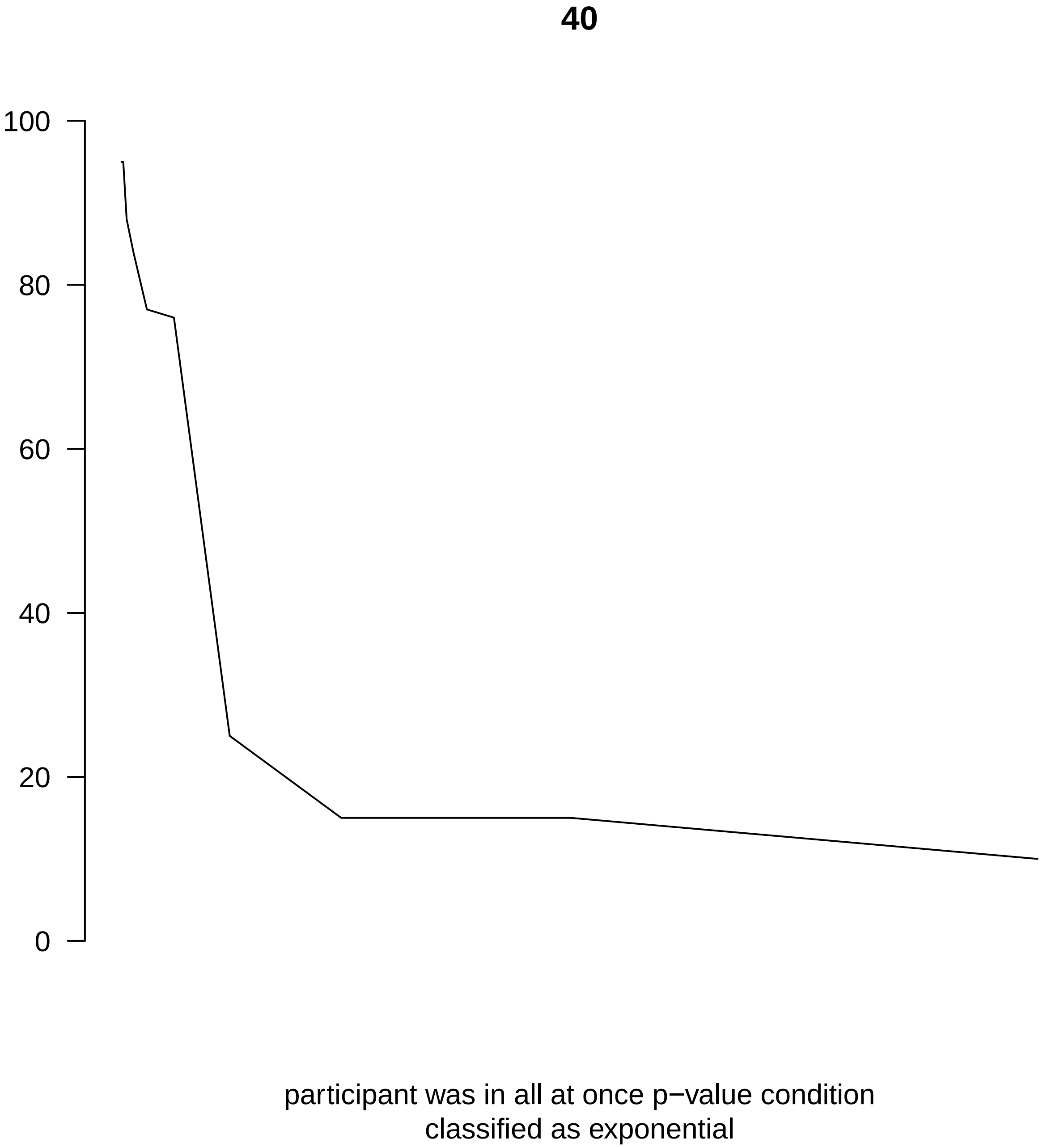
40
100
80
60
40
20
0
participant was in all at once p−value condition
classified as exponential

48

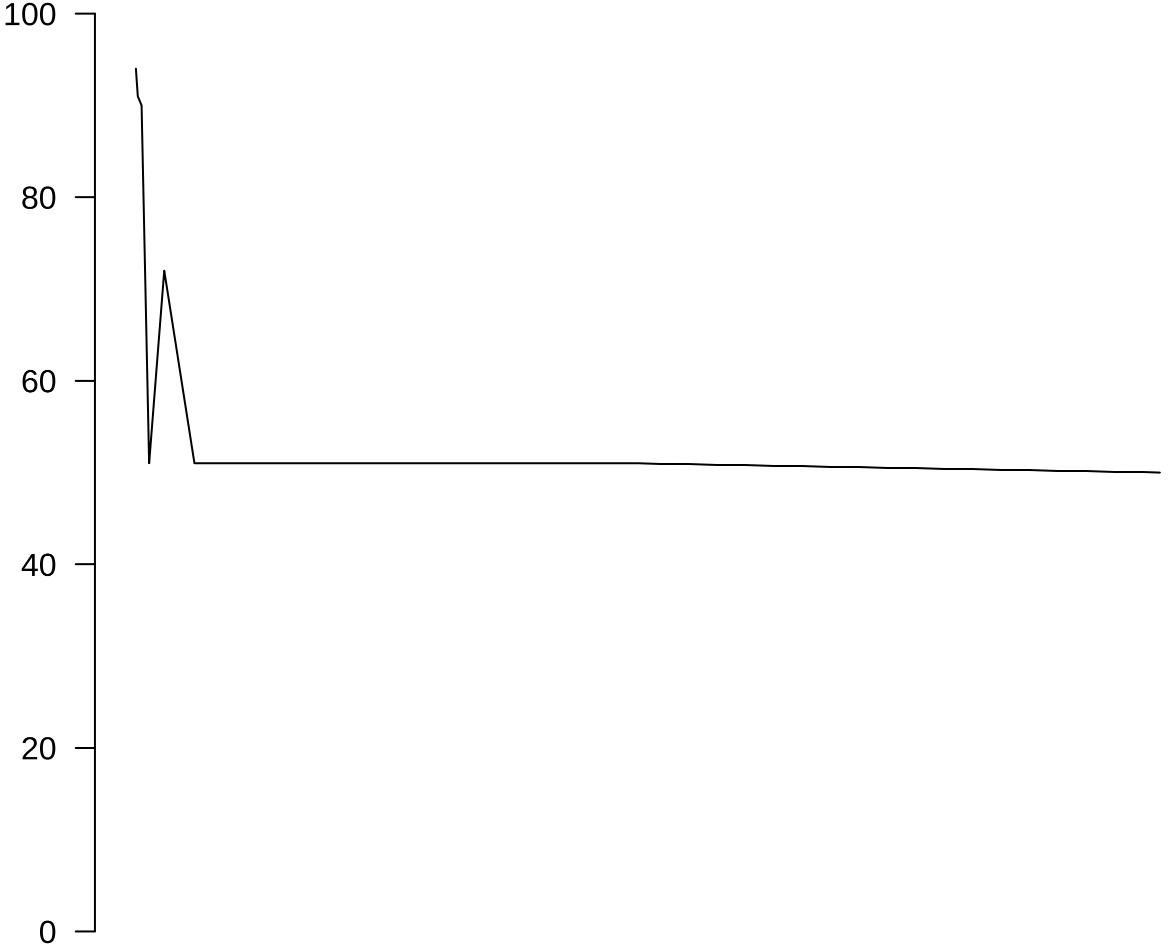

participant was in all at once p−value condition
classified as moderate cliff

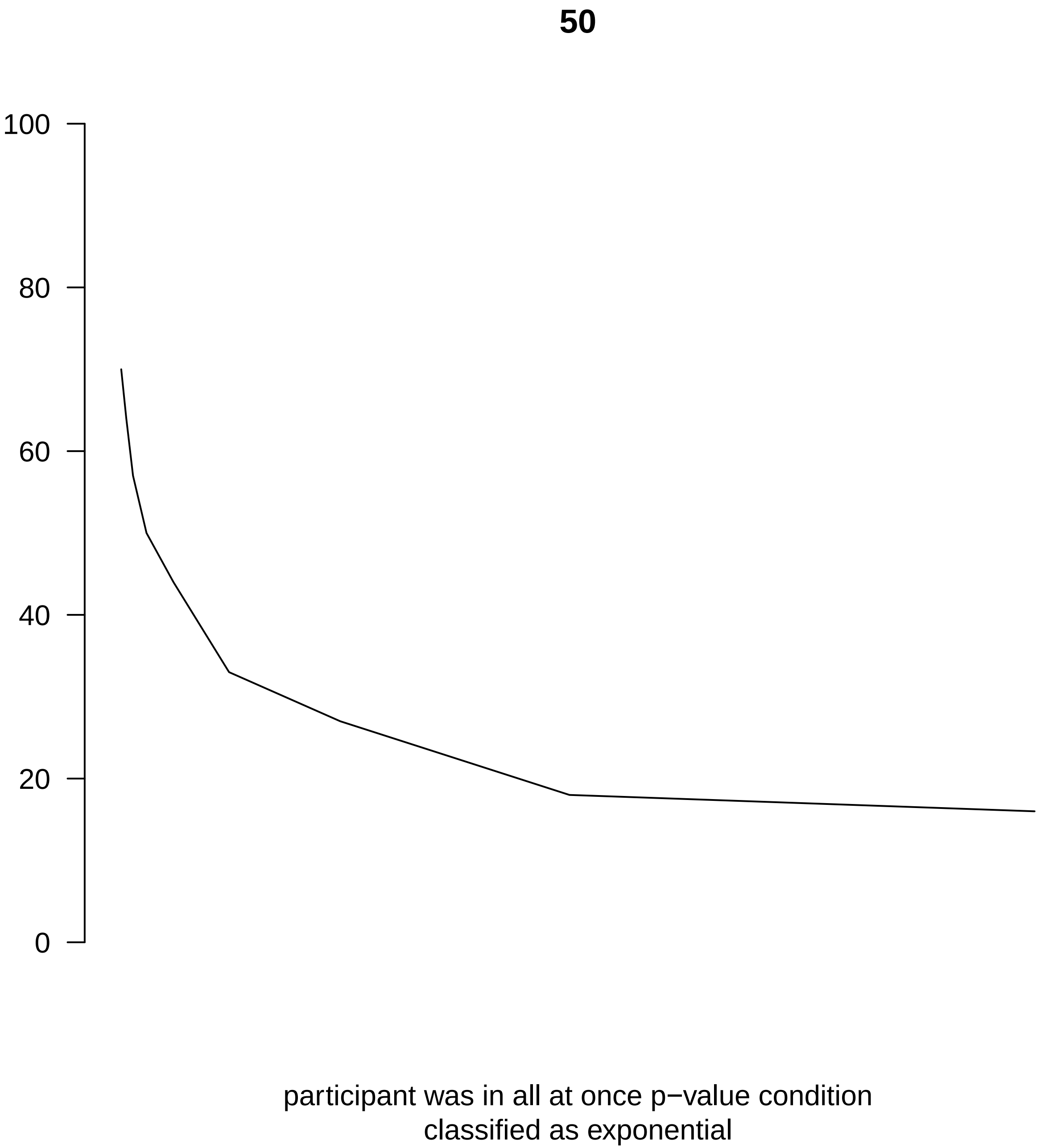
50
100
80
60
40
20
0
participant was in all at once p−value condition
classified as exponential

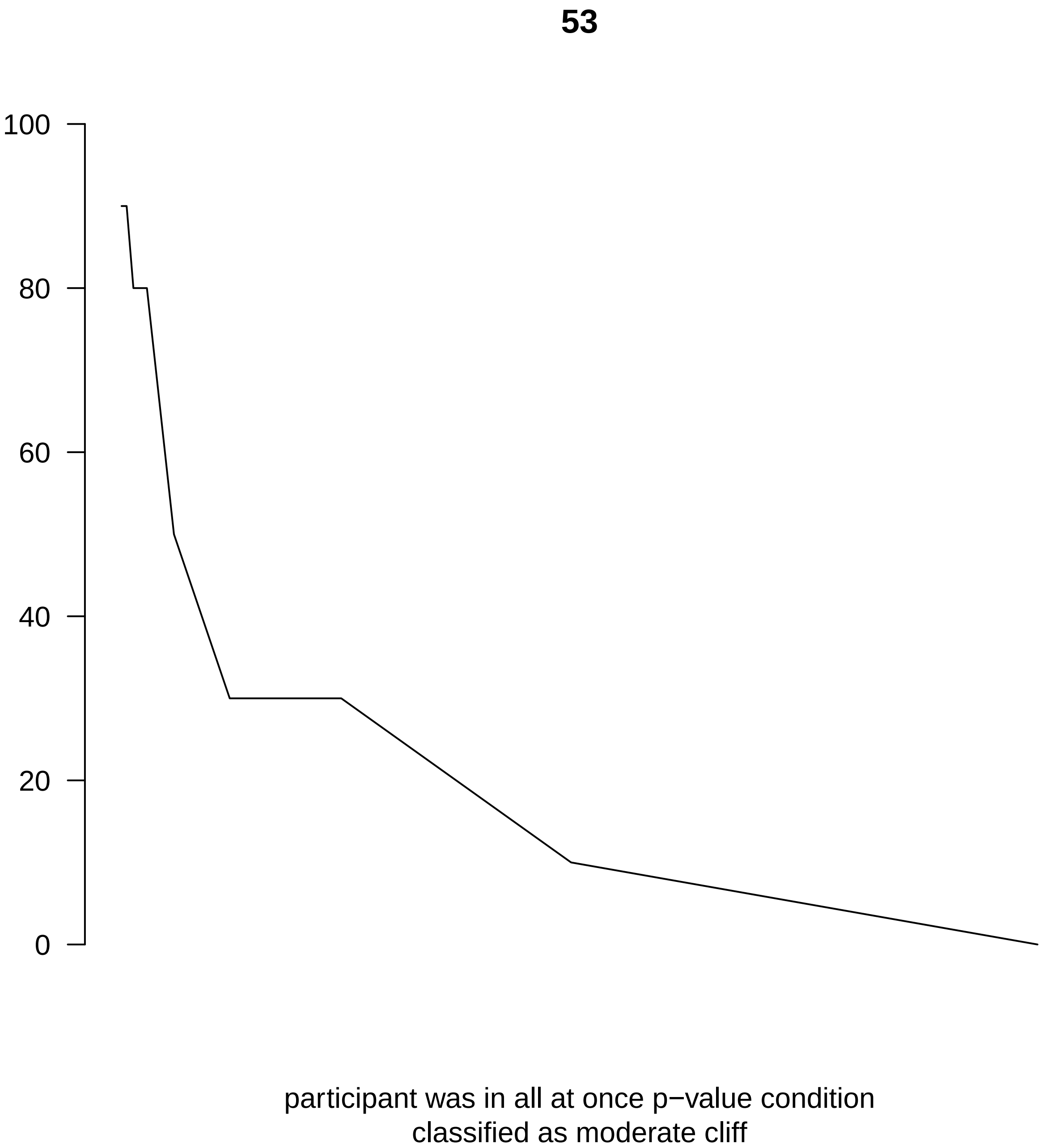
53
100
80
60
40
20
0
participant was in all at once p−value condition
classified as moderate cliff

55

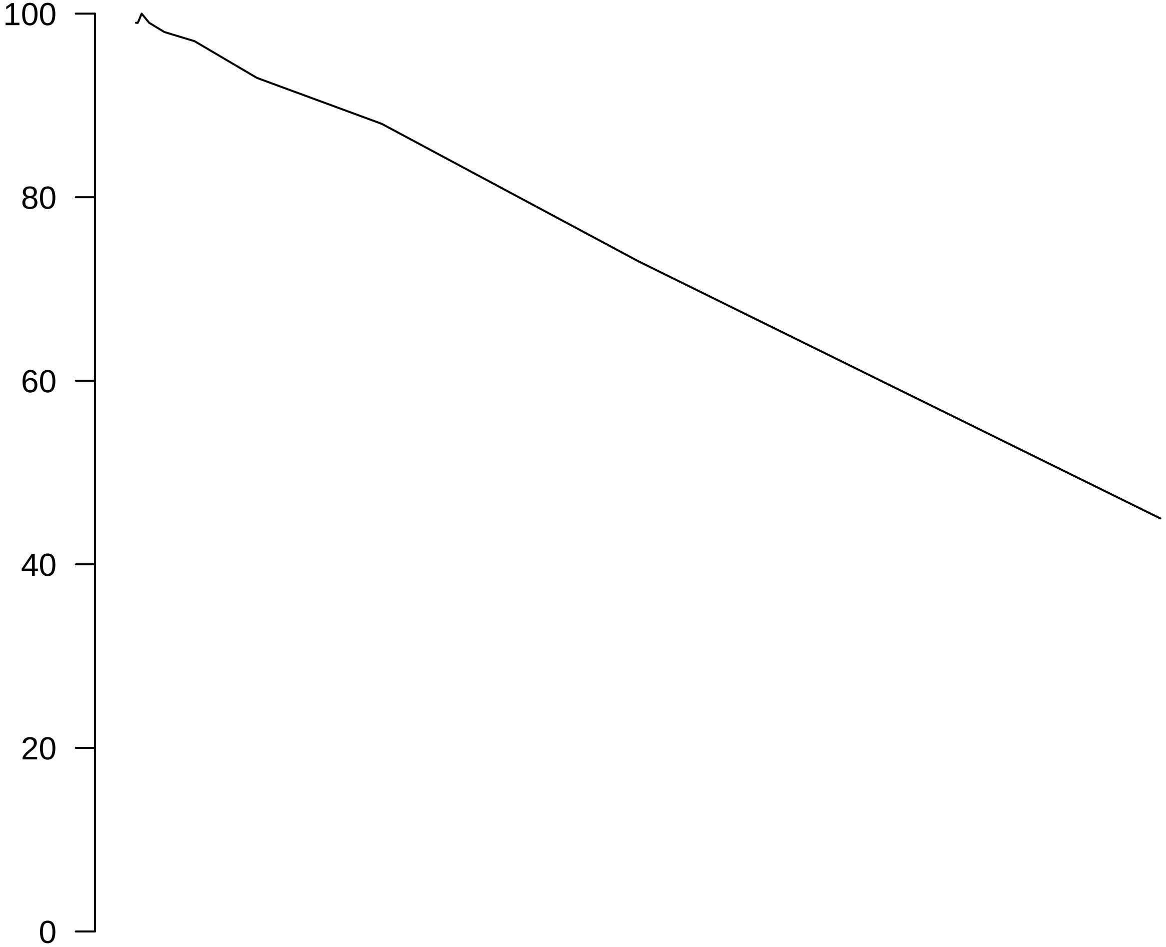

participant was in all at once p−value condition
classified as linear

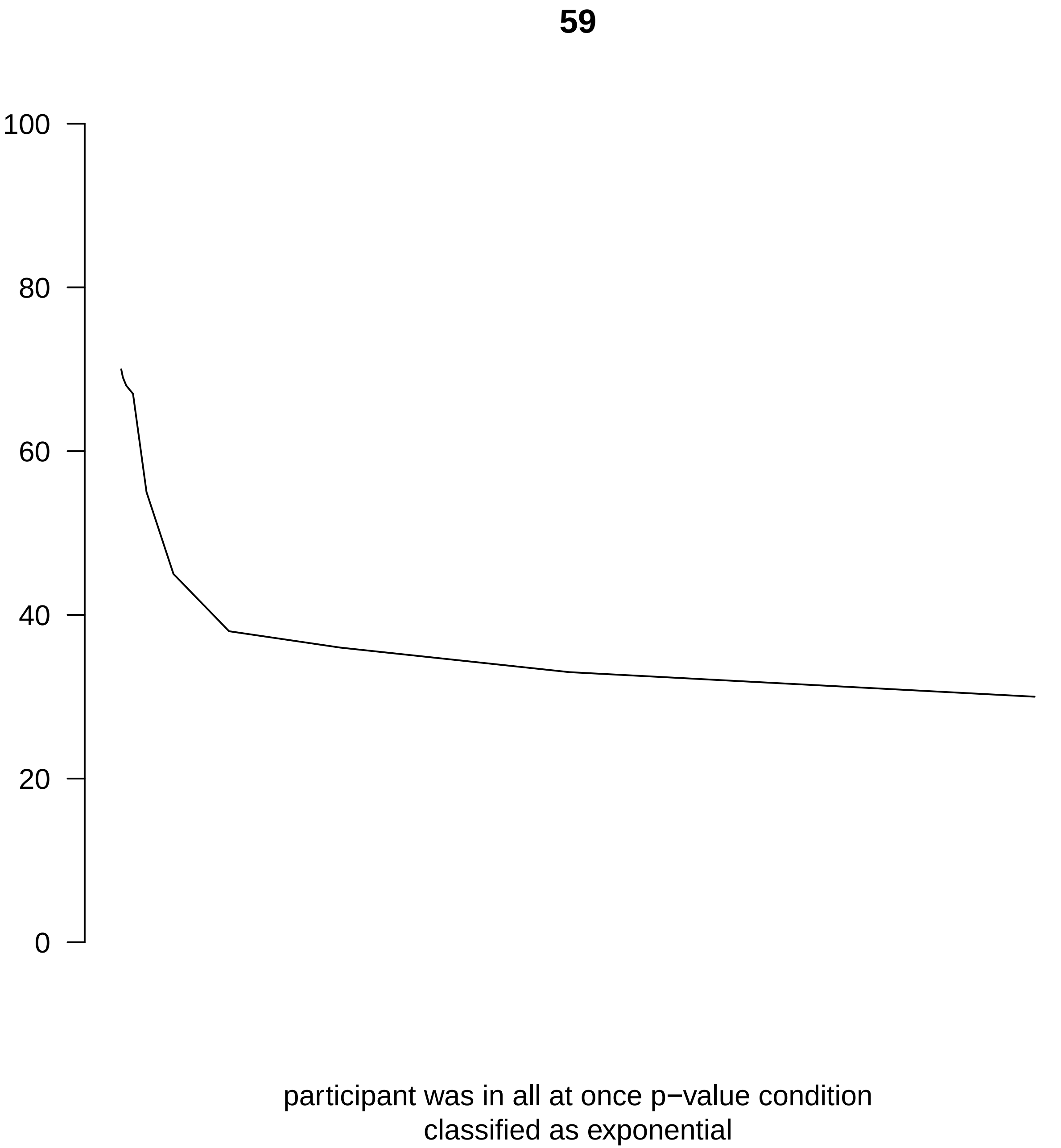
59
100
80
60
40
20
0
participant was in all at once p−value condition
classified as exponential

71

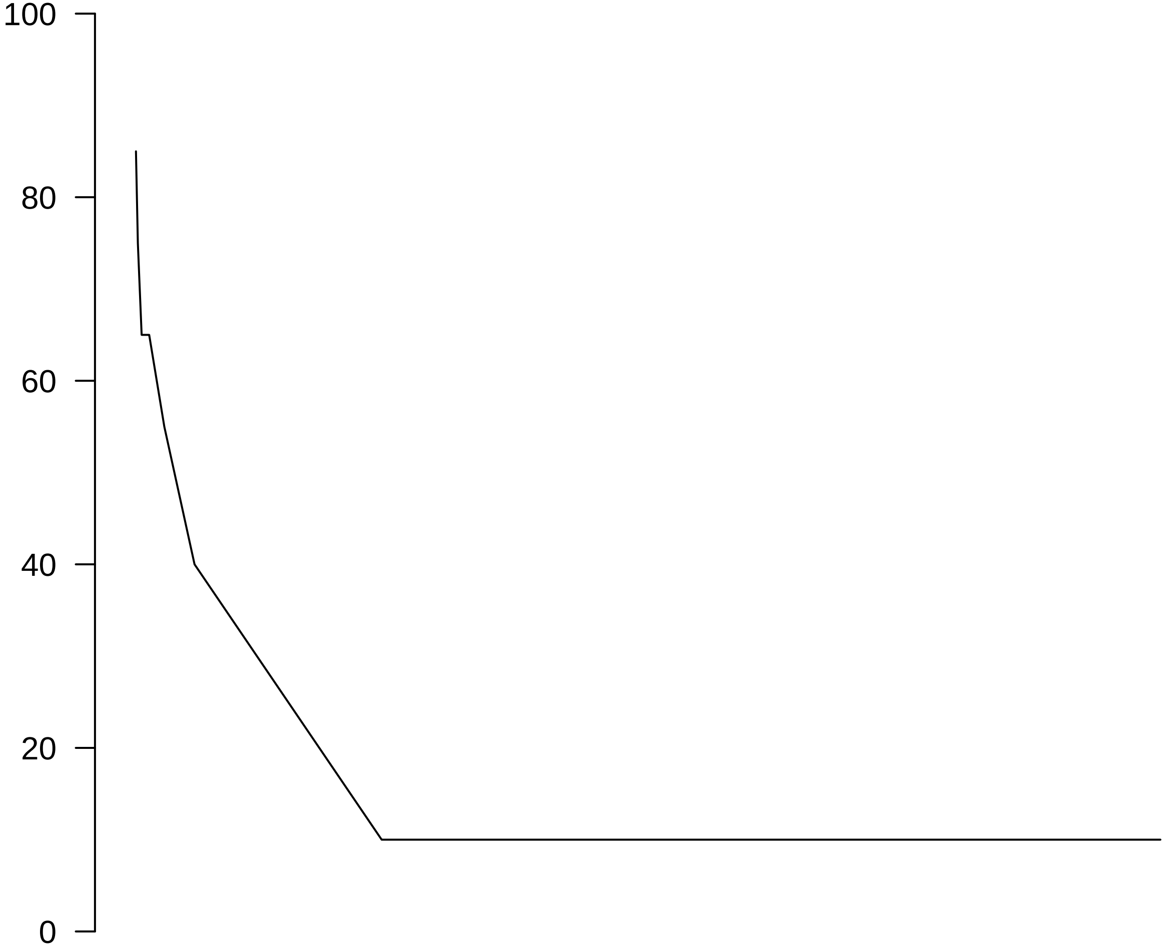

participant was in all at once p−value condition
classified as exponential

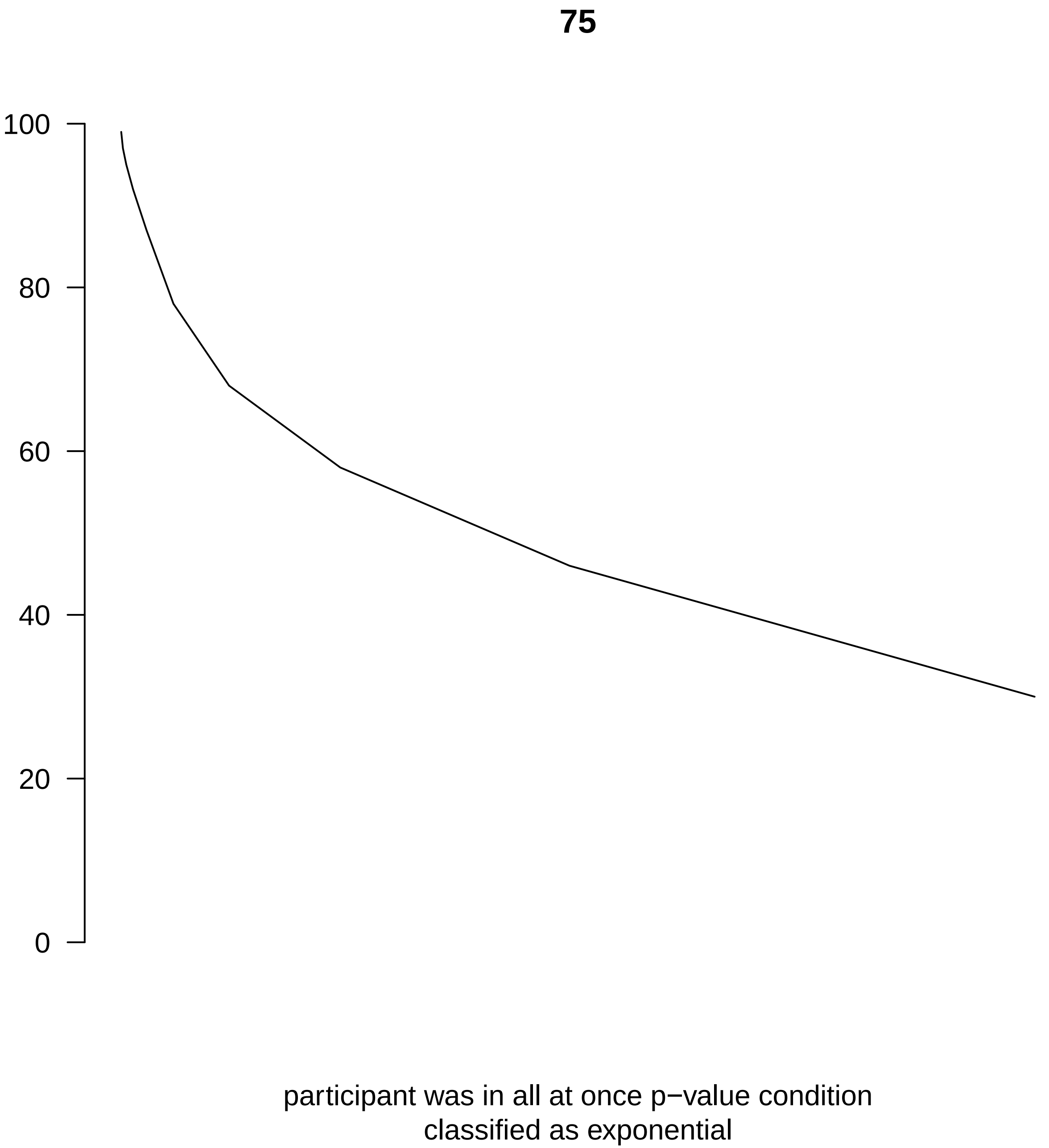
75
100
80
60
40
20
0
participant was in all at once p−value condition
classified as exponential

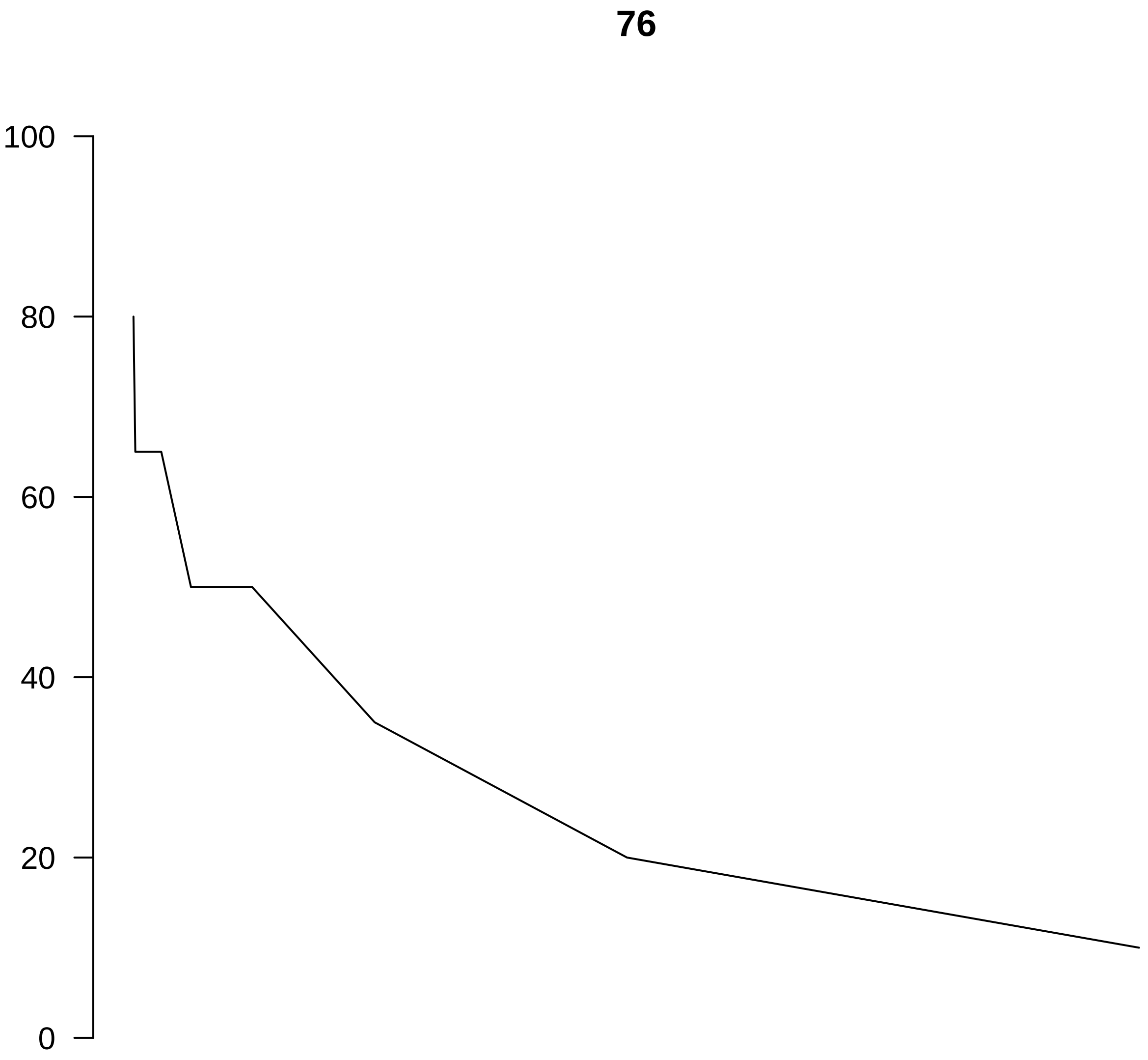

participant was in all at once p−value condition
classified as exponential

85

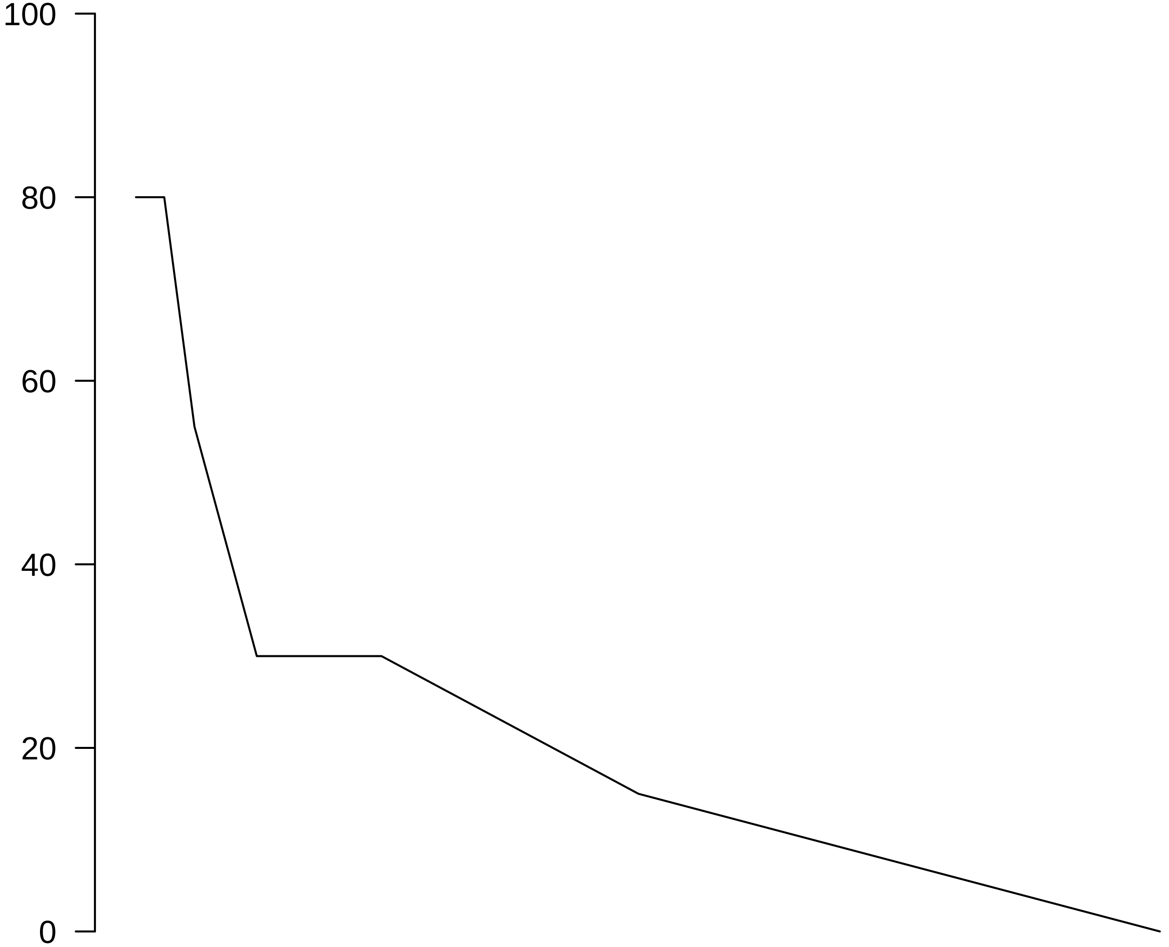

participant was in all at once p−value condition
classified as moderate cliff

88

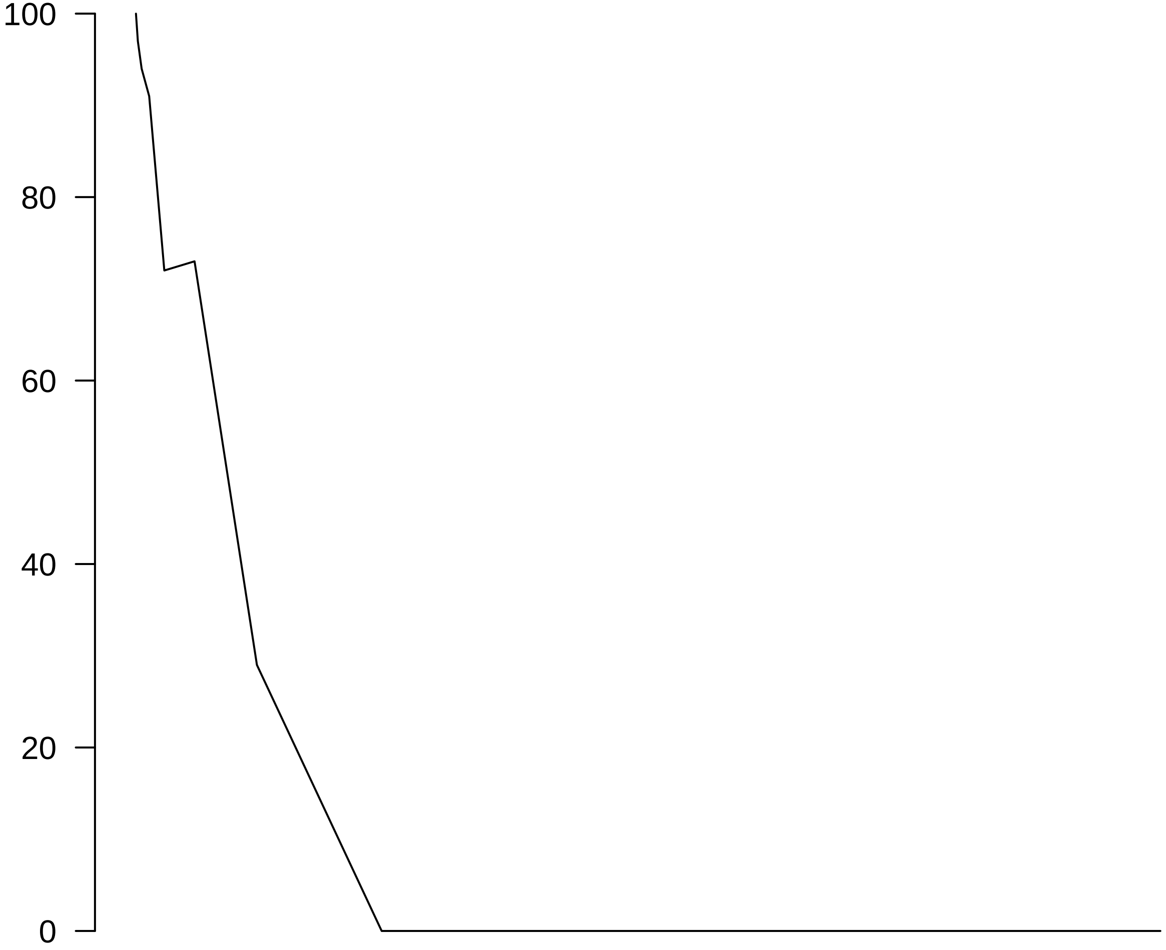

participant was in all at once p−value condition
classified as exponential

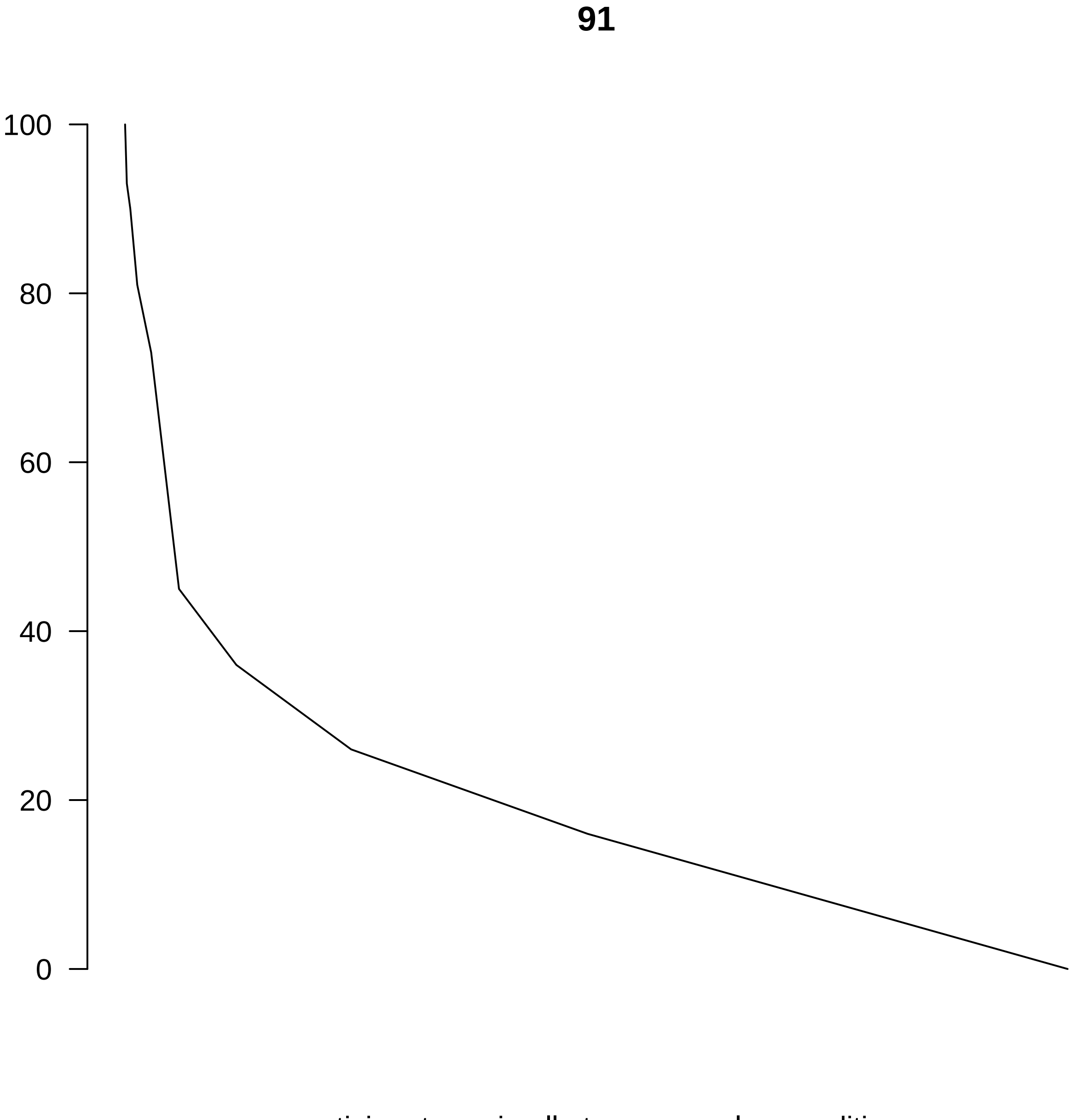

participant was in all at once p−value condition
classified as exponential

98

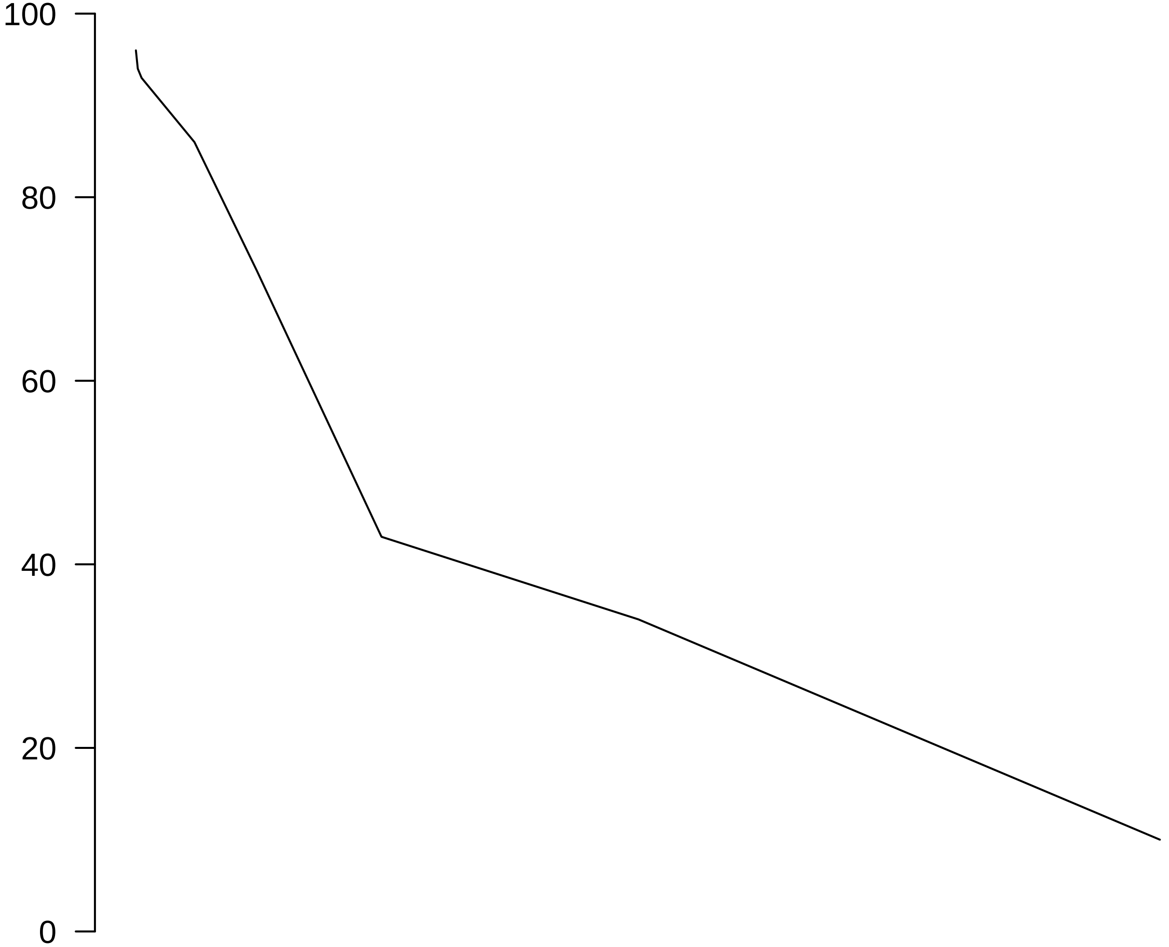

participant was in all at once p−value condition
classified as exponential

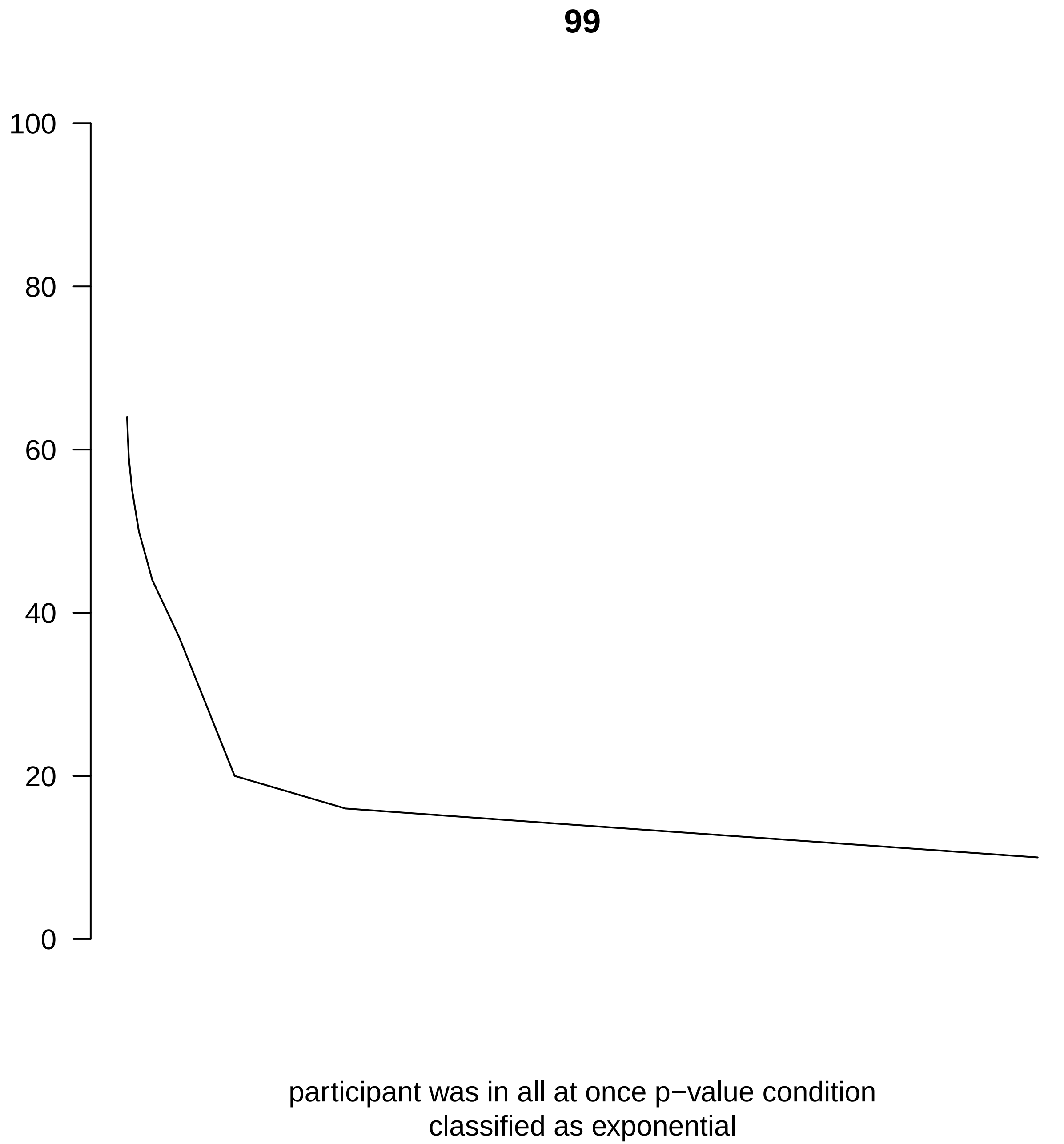
99
100
80
60
40
20
0
participant was in all at once p−value condition
classified as exponential

103

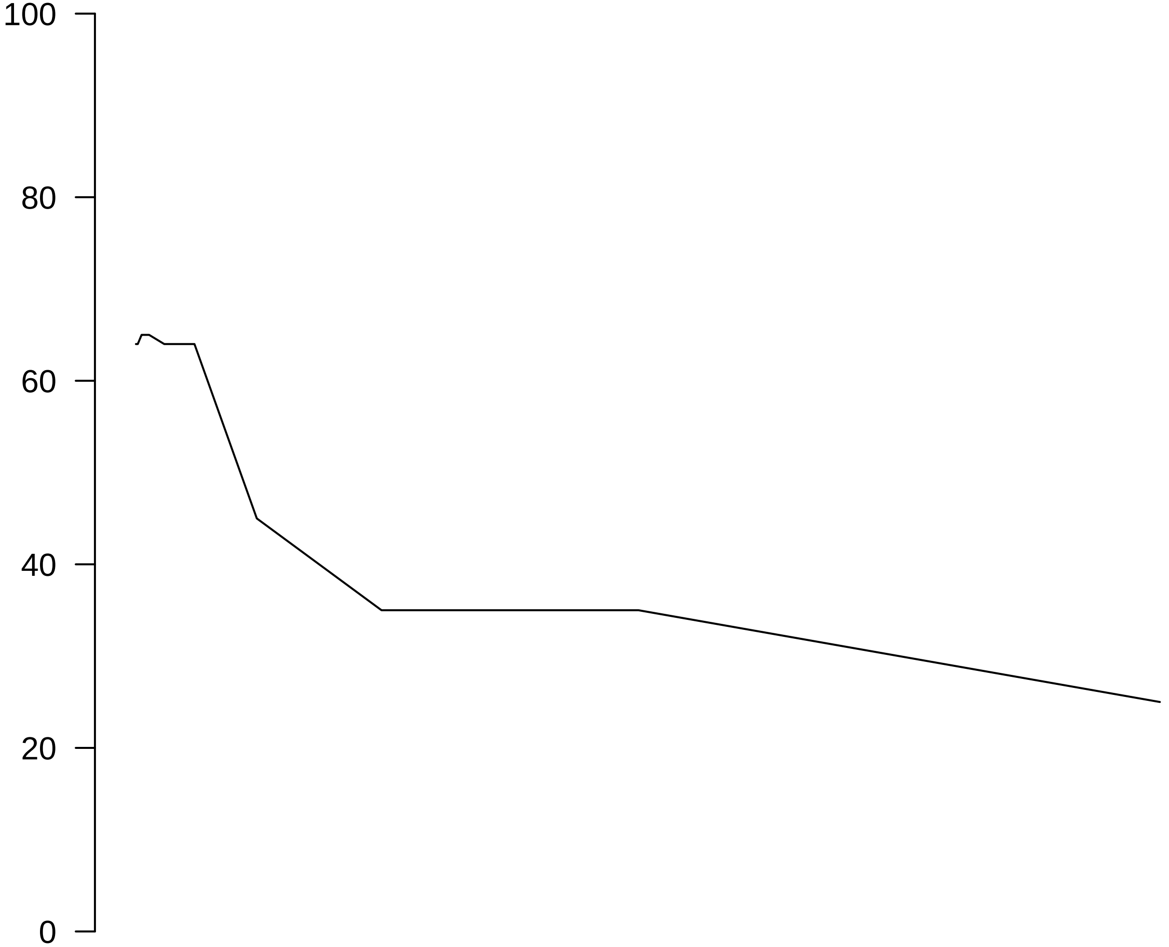

participant was in all at once p−value condition
classified as moderate cliff

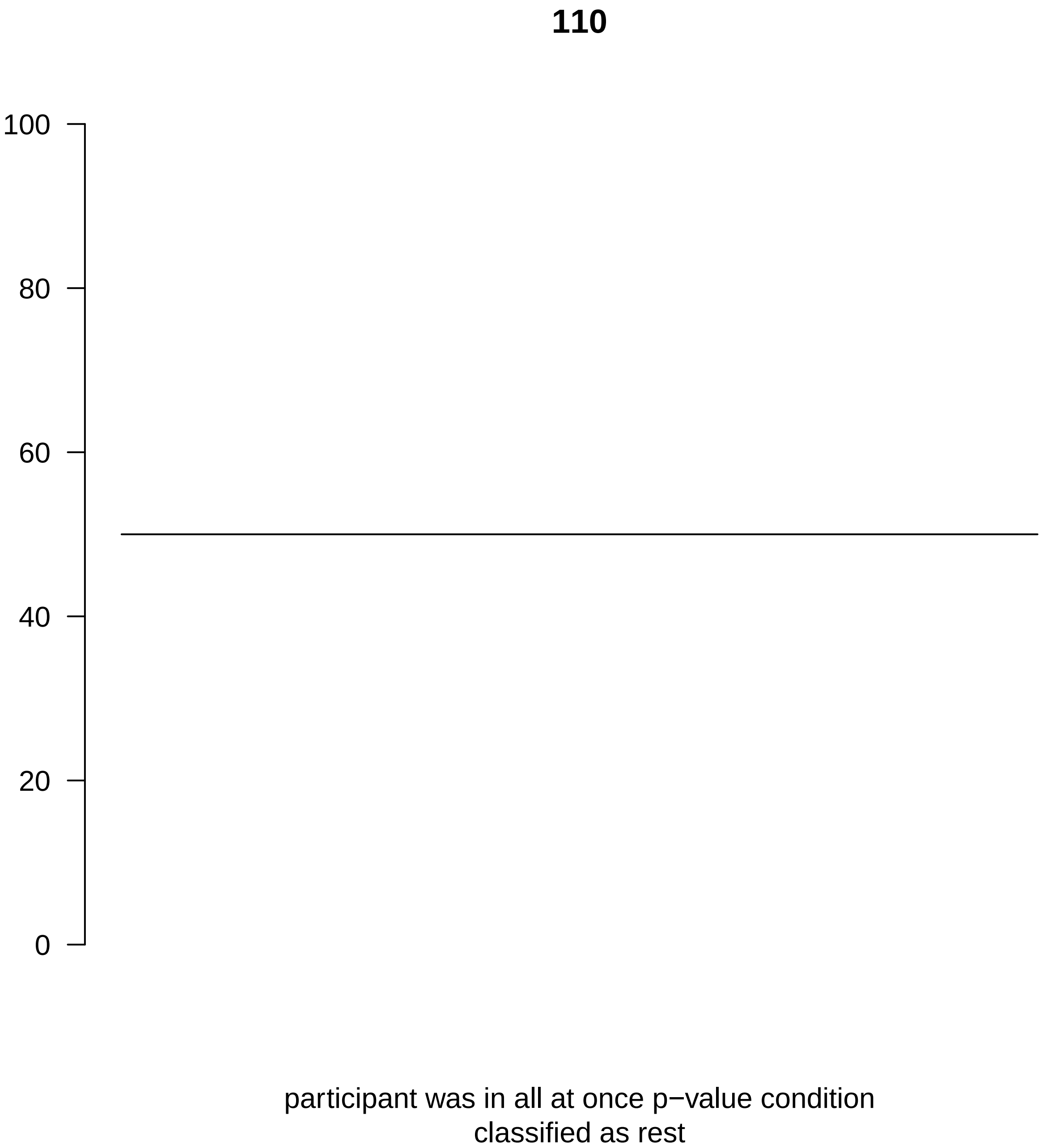
110
100
80
60
40
20
0
participant was in all at once p−value condition
classified as rest

115

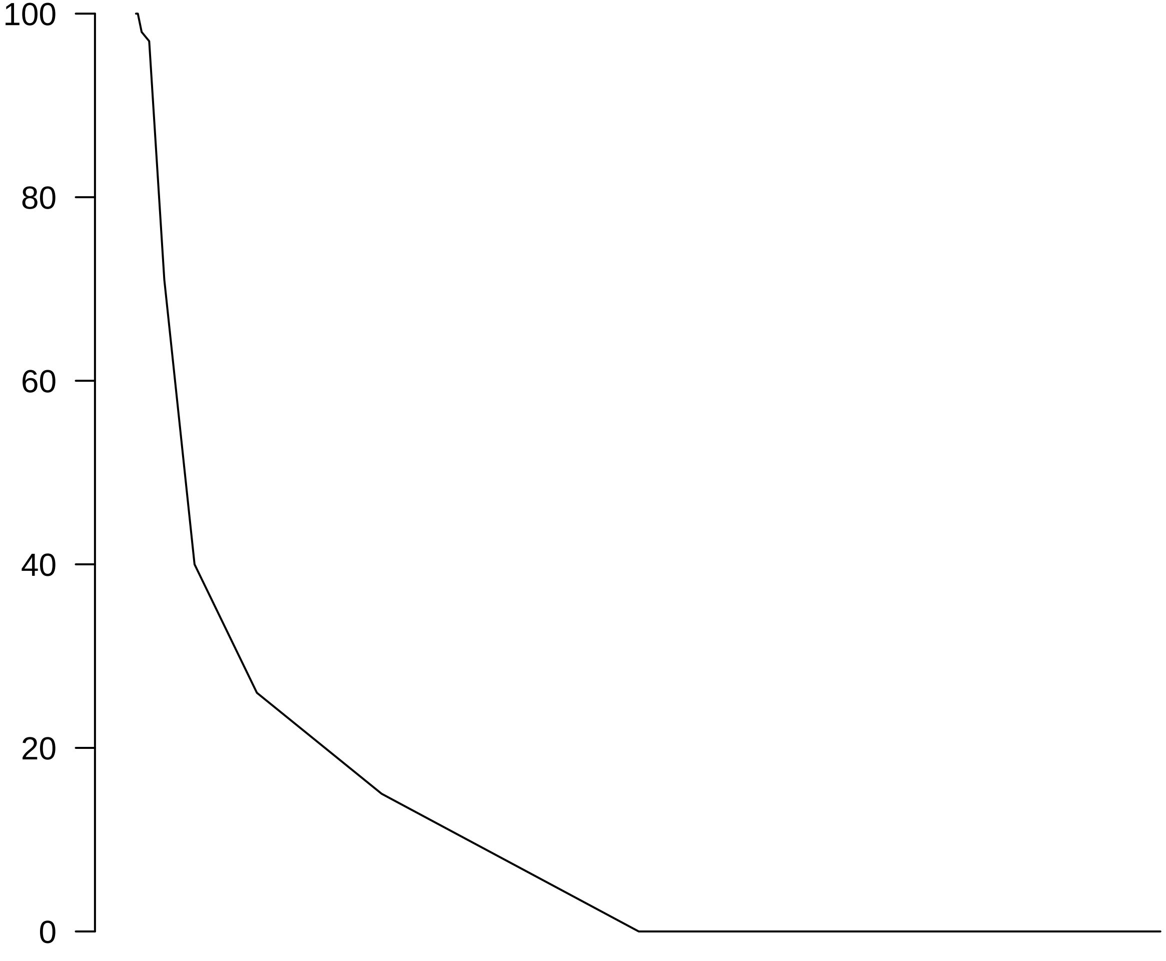

participant was in all at once p−value condition
classified as exponential

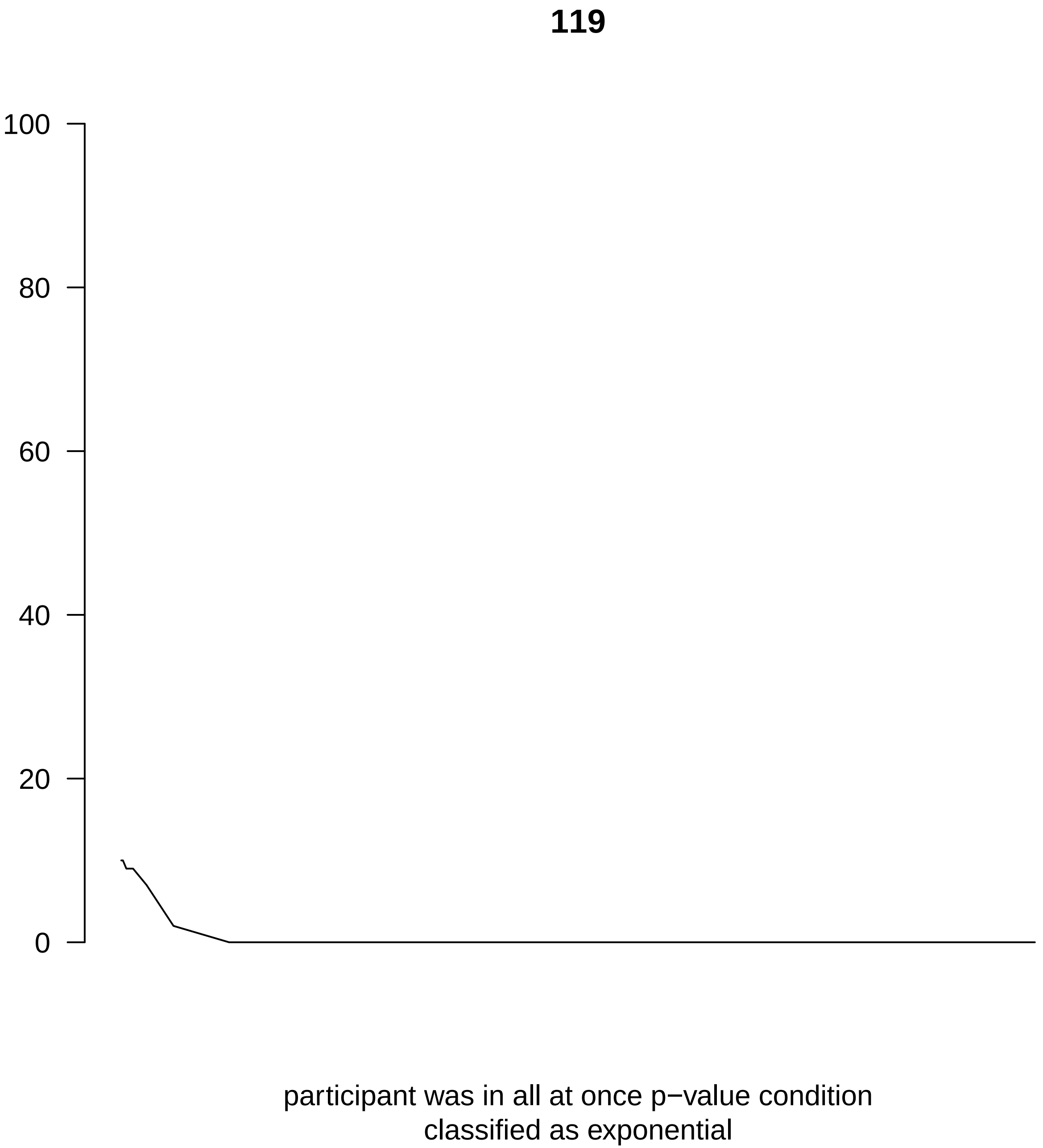
119
100
80
60
40
20
0
participant was in all at once p−value condition
classified as exponential

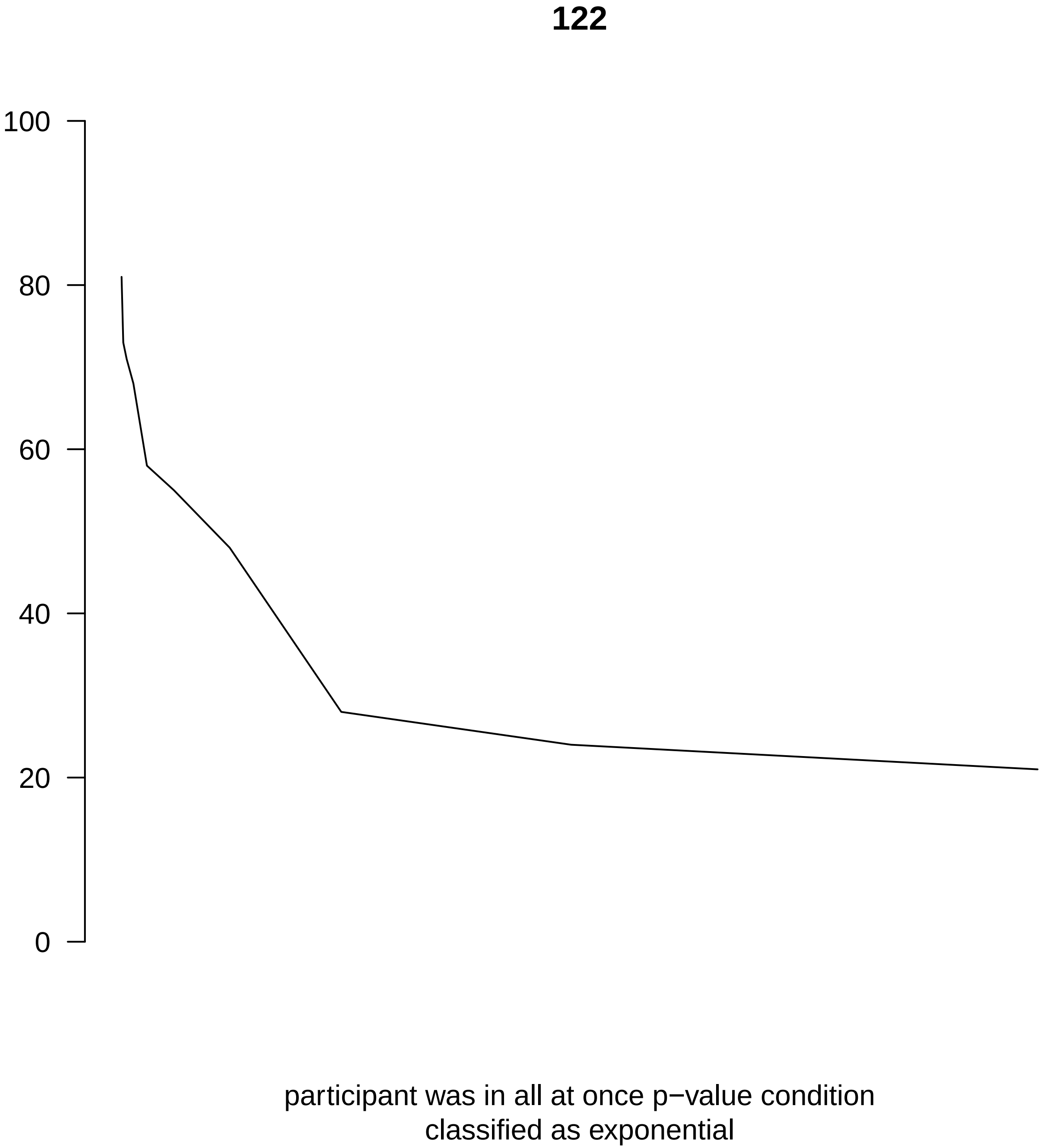
122
100
80
60
40
20
0
participant was in all at once p−value condition
classified as exponential

126

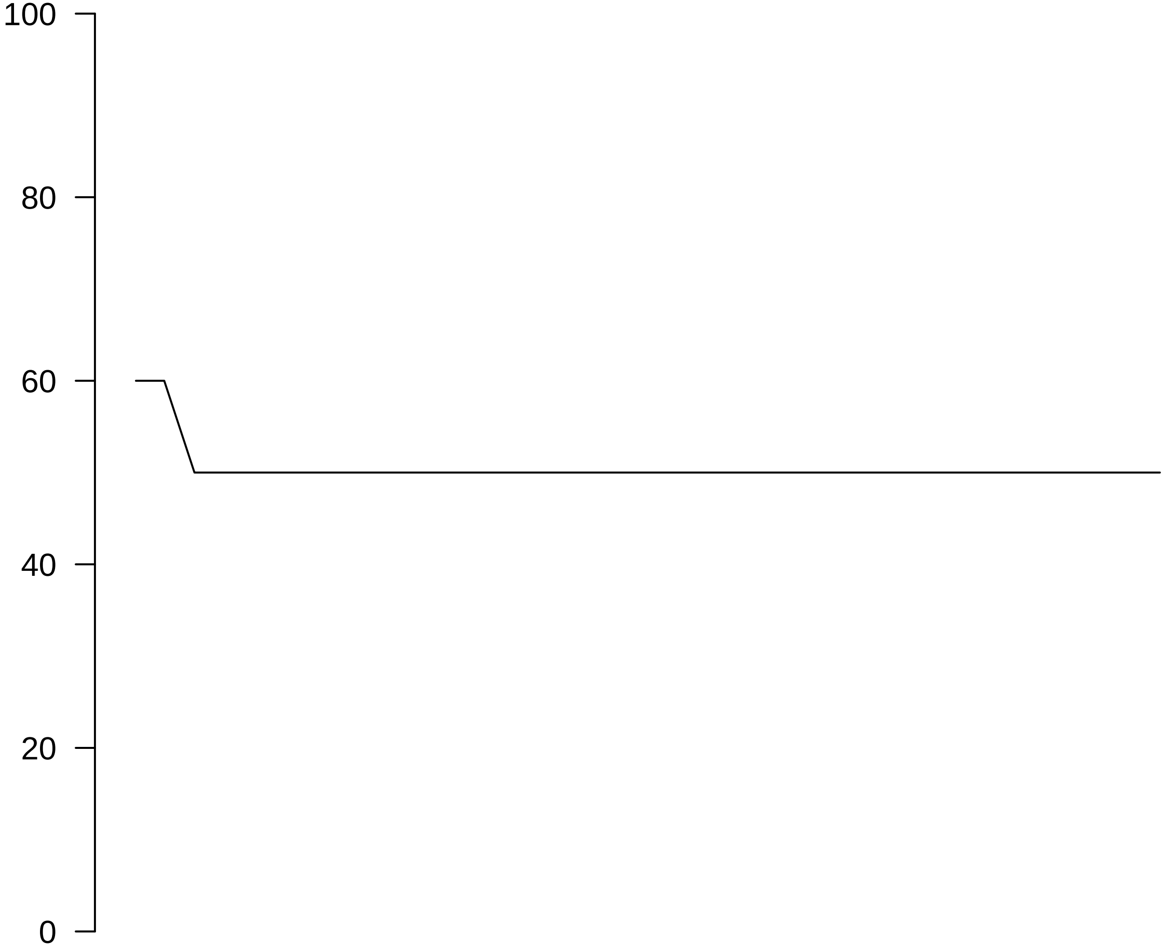

participant was in all at once p−value condition
classified as moderate cliff

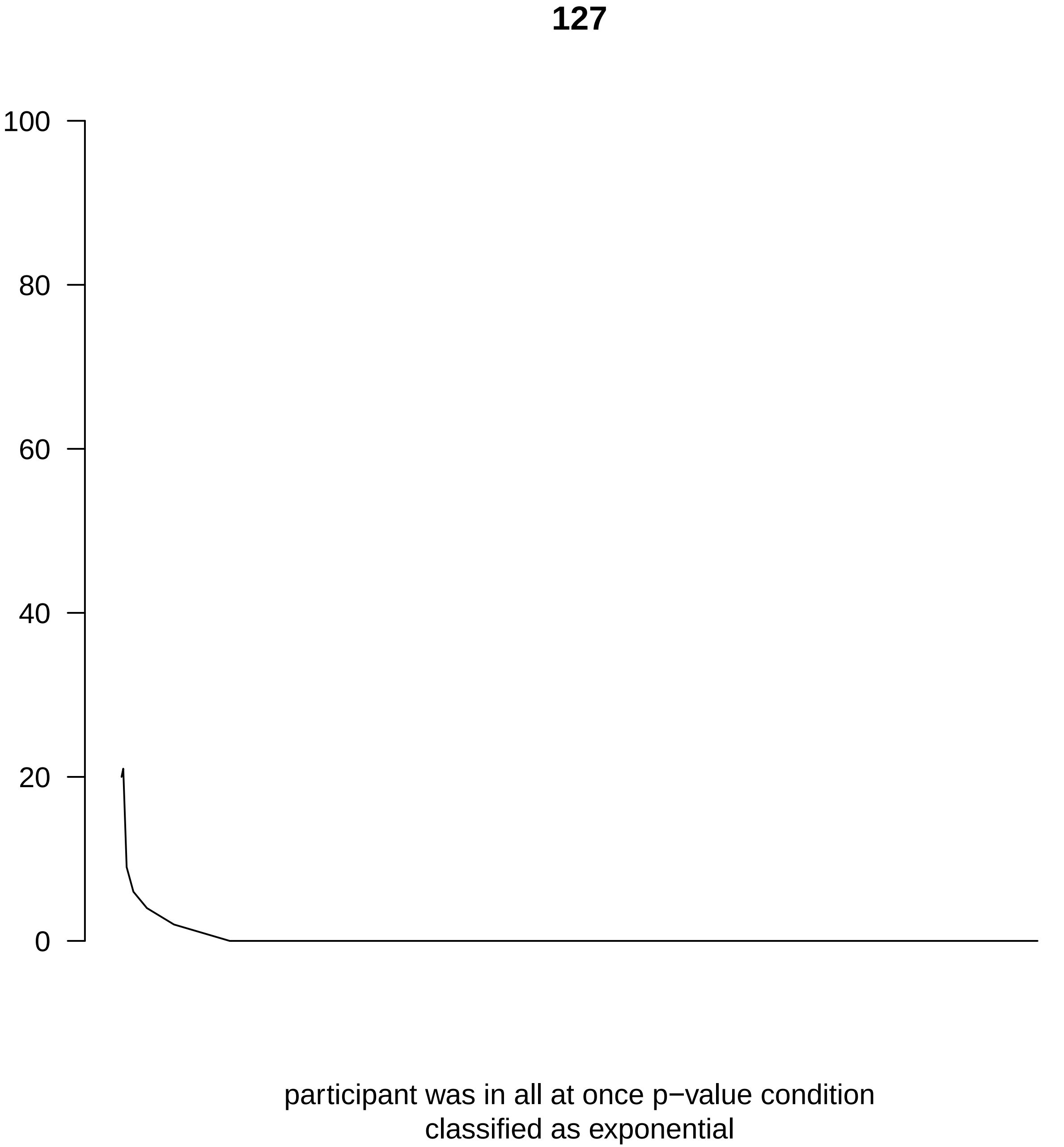
127
100
80
60
40
20
0
participant was in all at once p−value condition
classified as exponential

132

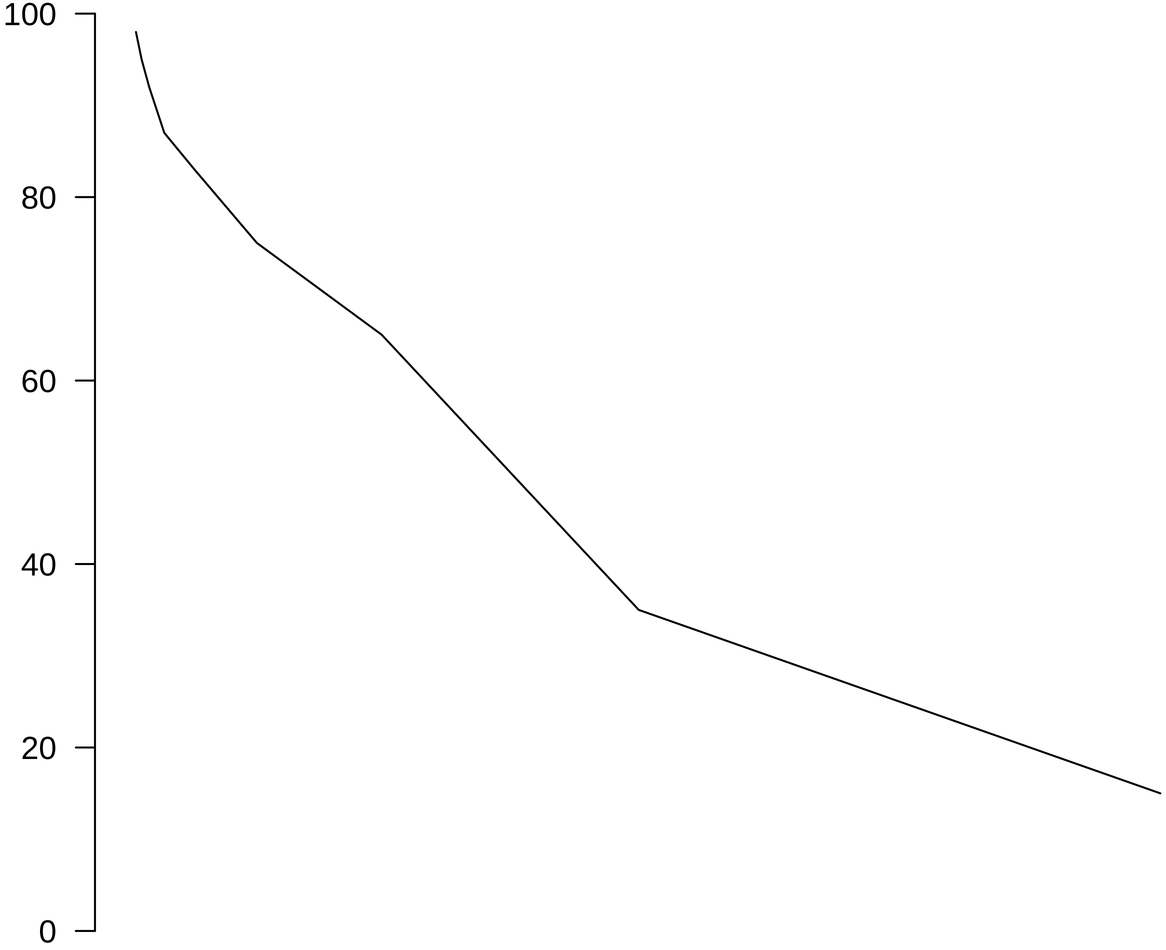

participant was in all at once p−value condition
classified as exponential

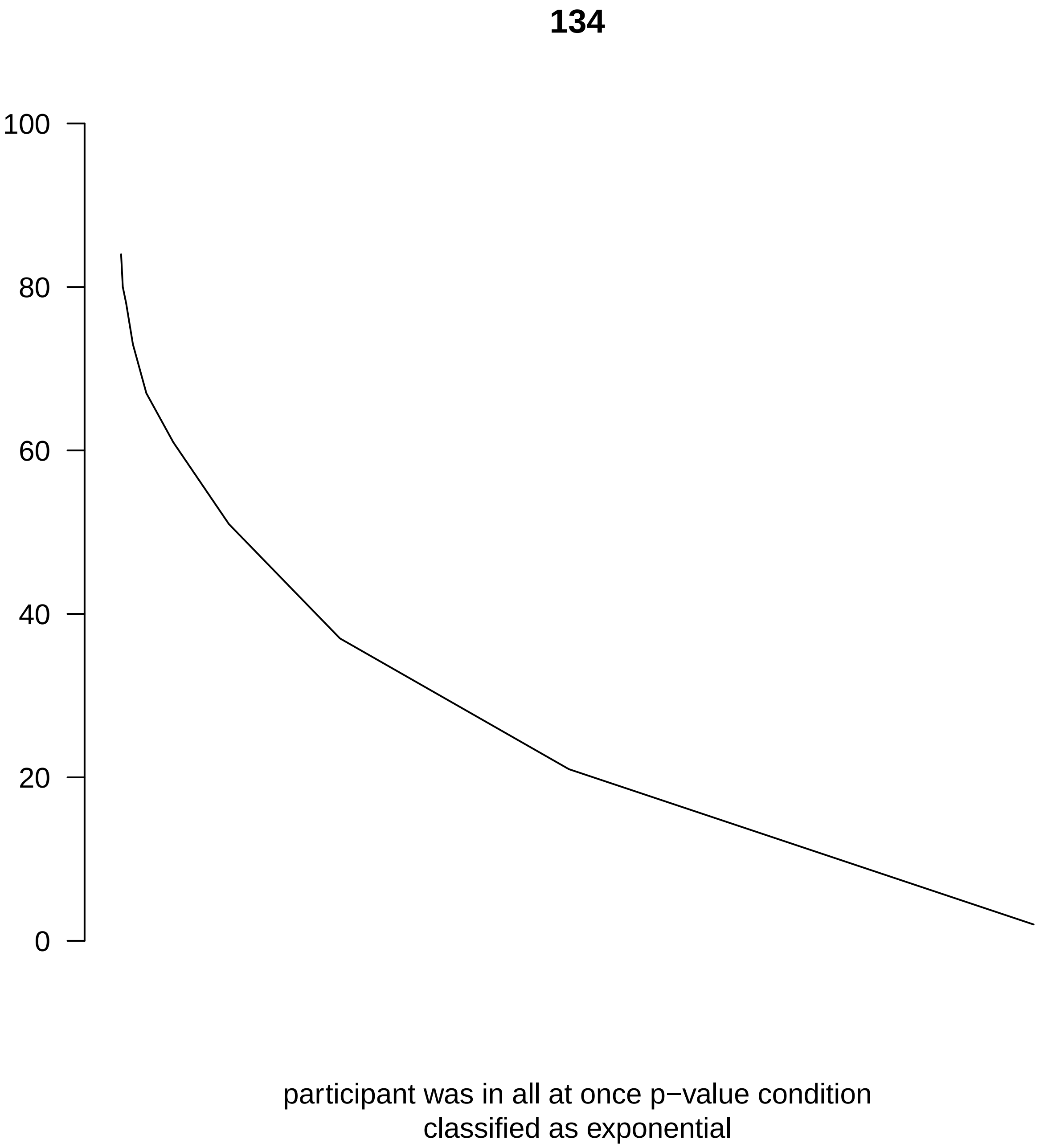
134
100
80
60
40
20
0
participant was in all at once p−value condition
classified as exponential

138

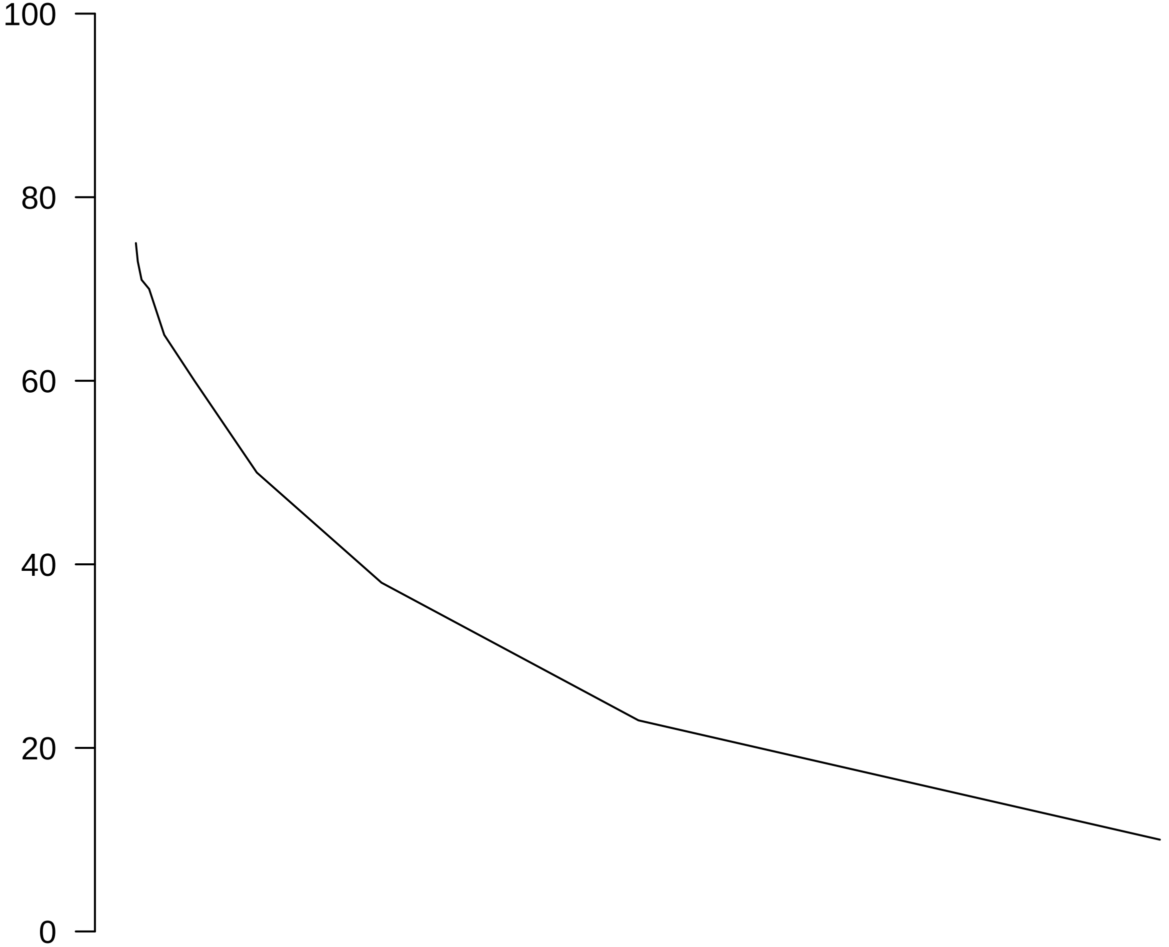

participant was in all at once p−value condition
classified as exponential

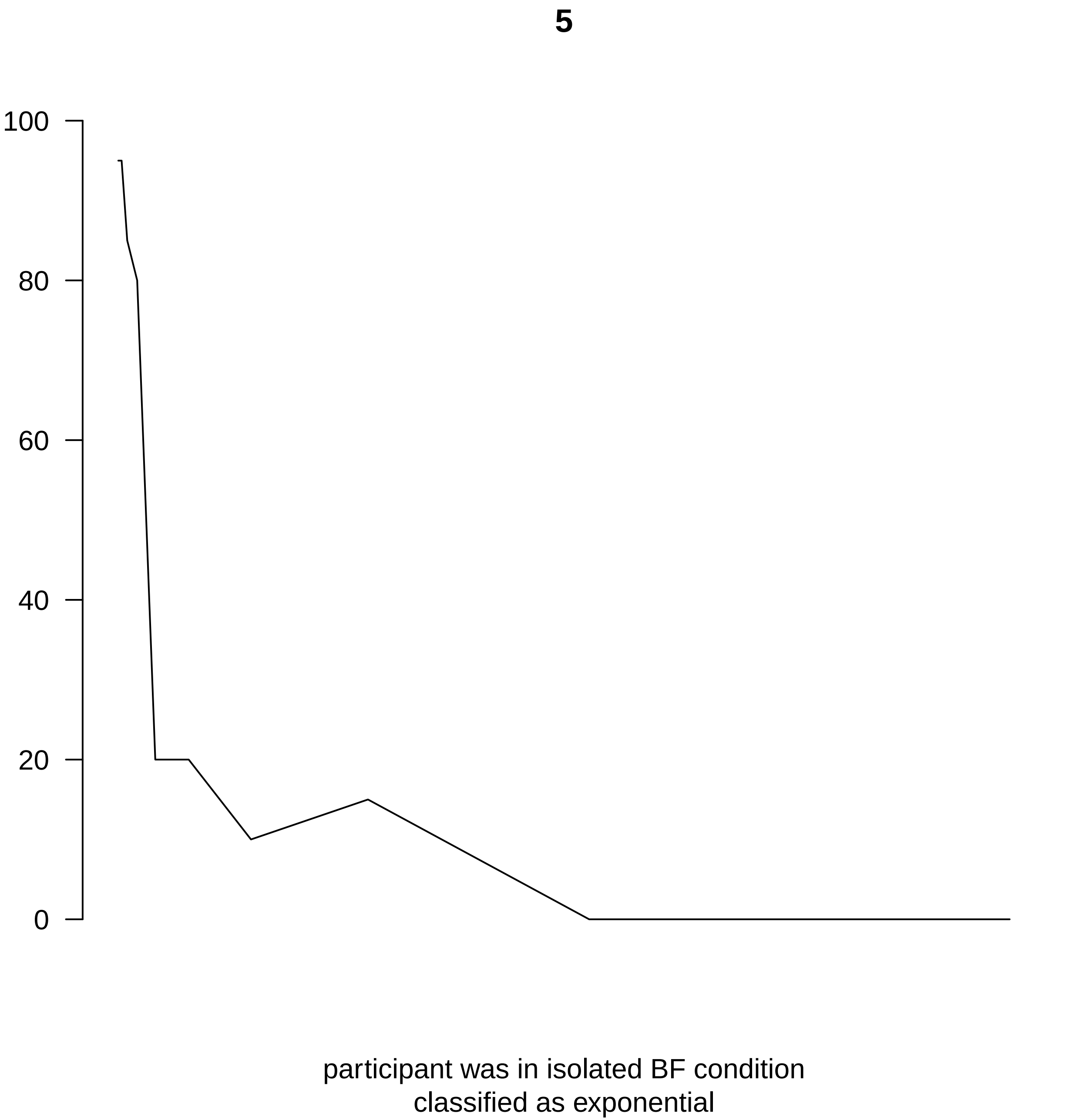

5
100
80
60
40
20
0
participant was in isolated BF condition
classified as exponential

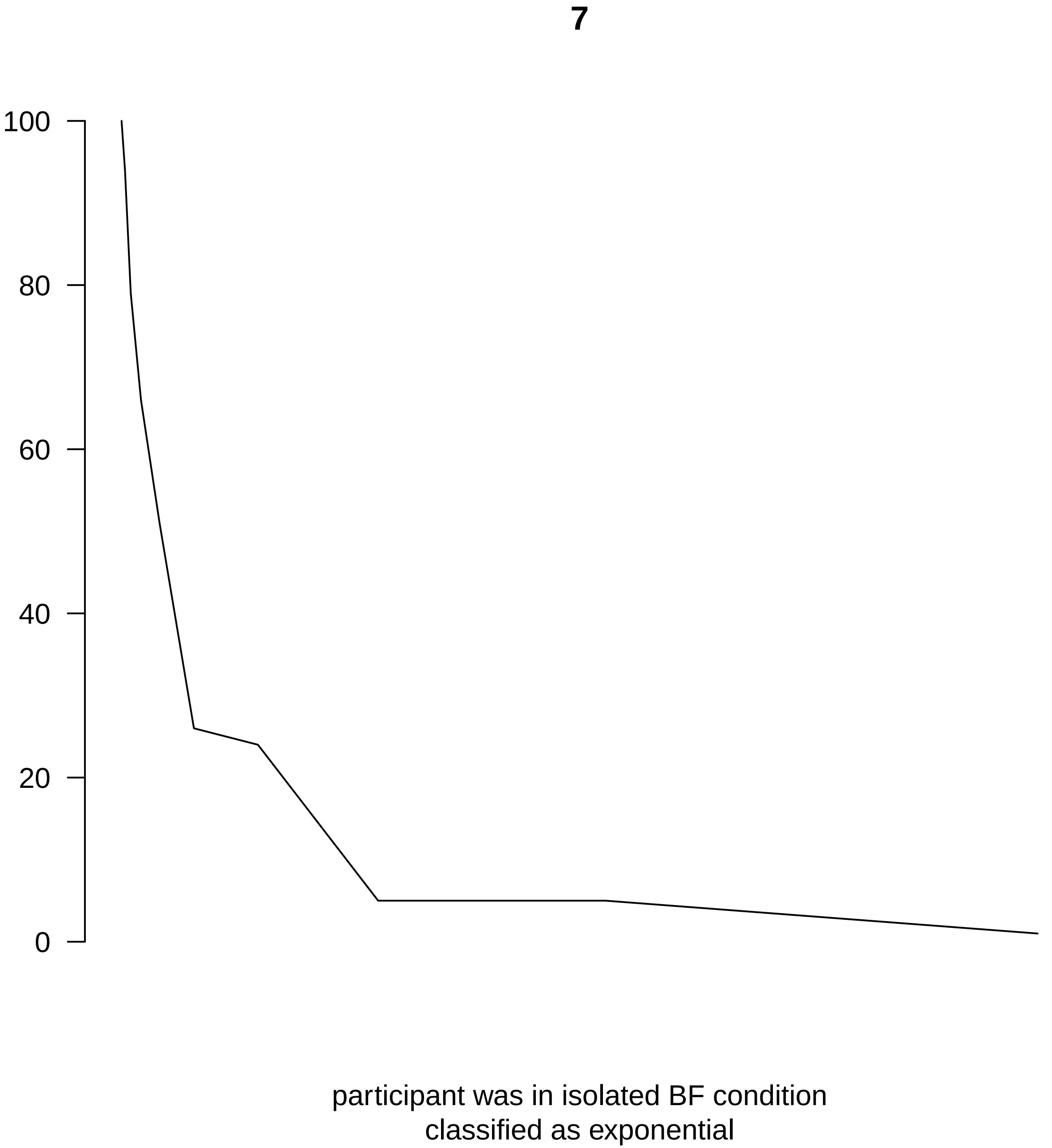
7
100
80
60
40
20
0
participant was in isolated BF condition
classified as exponential

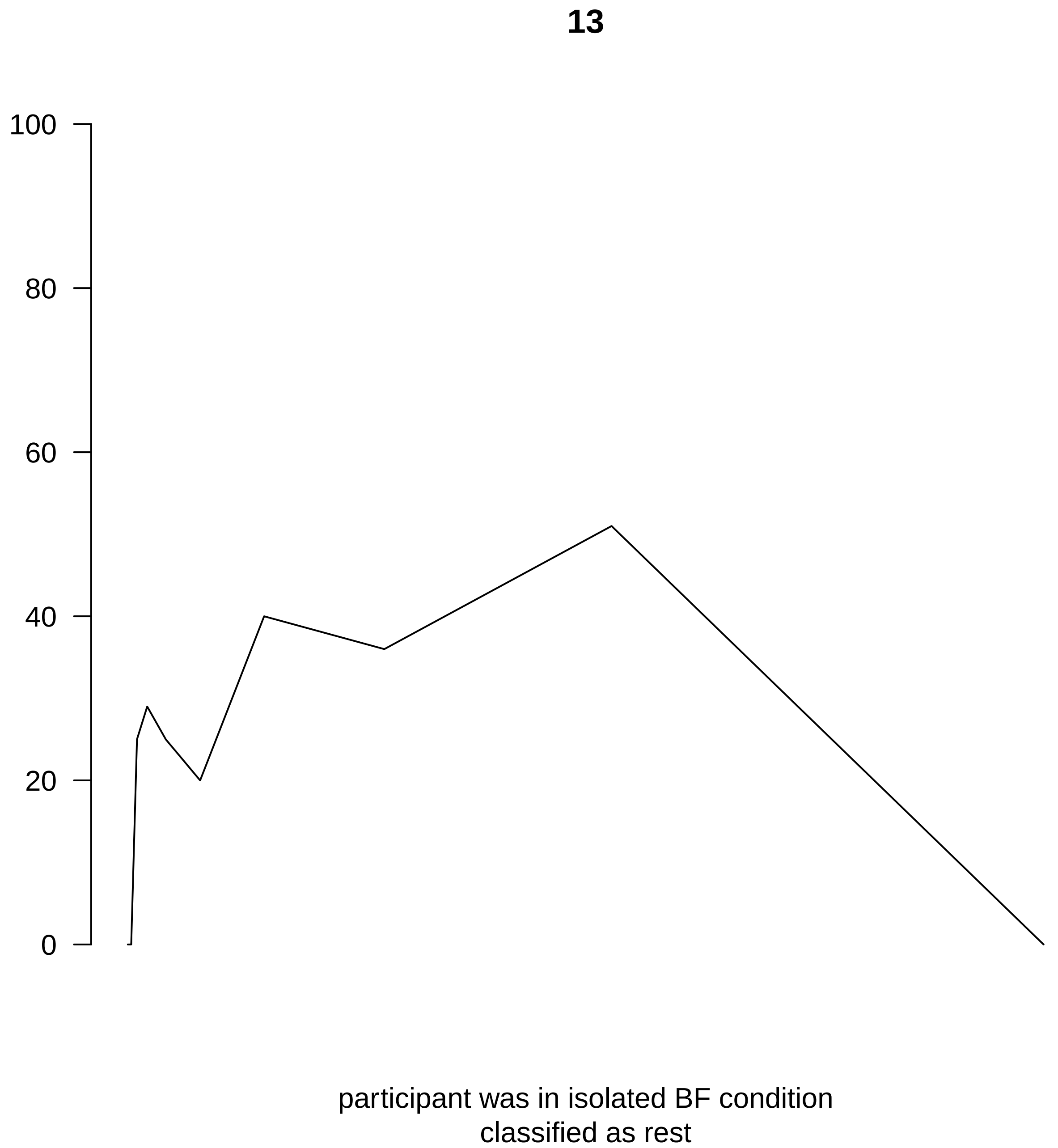
13
100
80
60
40
20
0
participant was in isolated BF condition
classified as rest

19

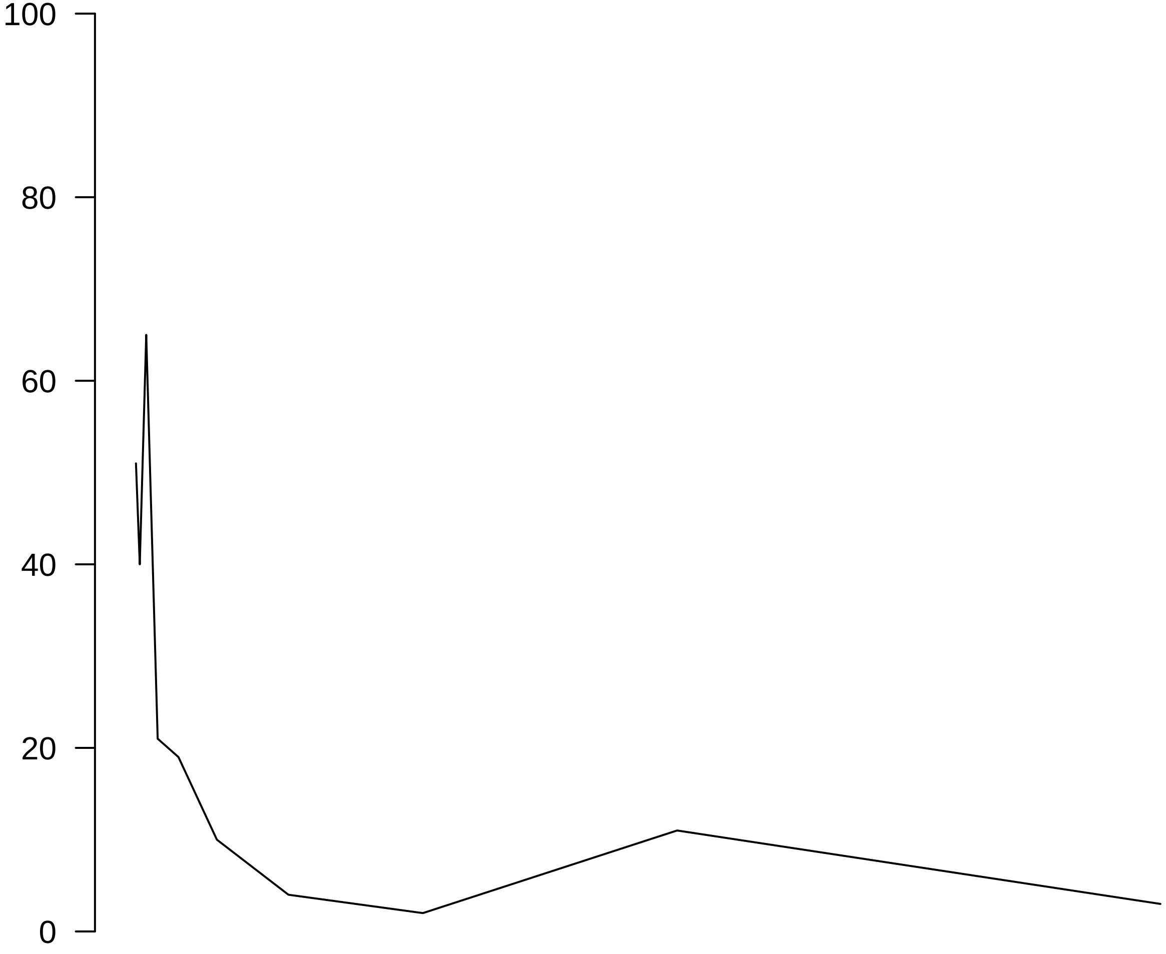

participant was in isolated BF condition
classified as moderate cliff

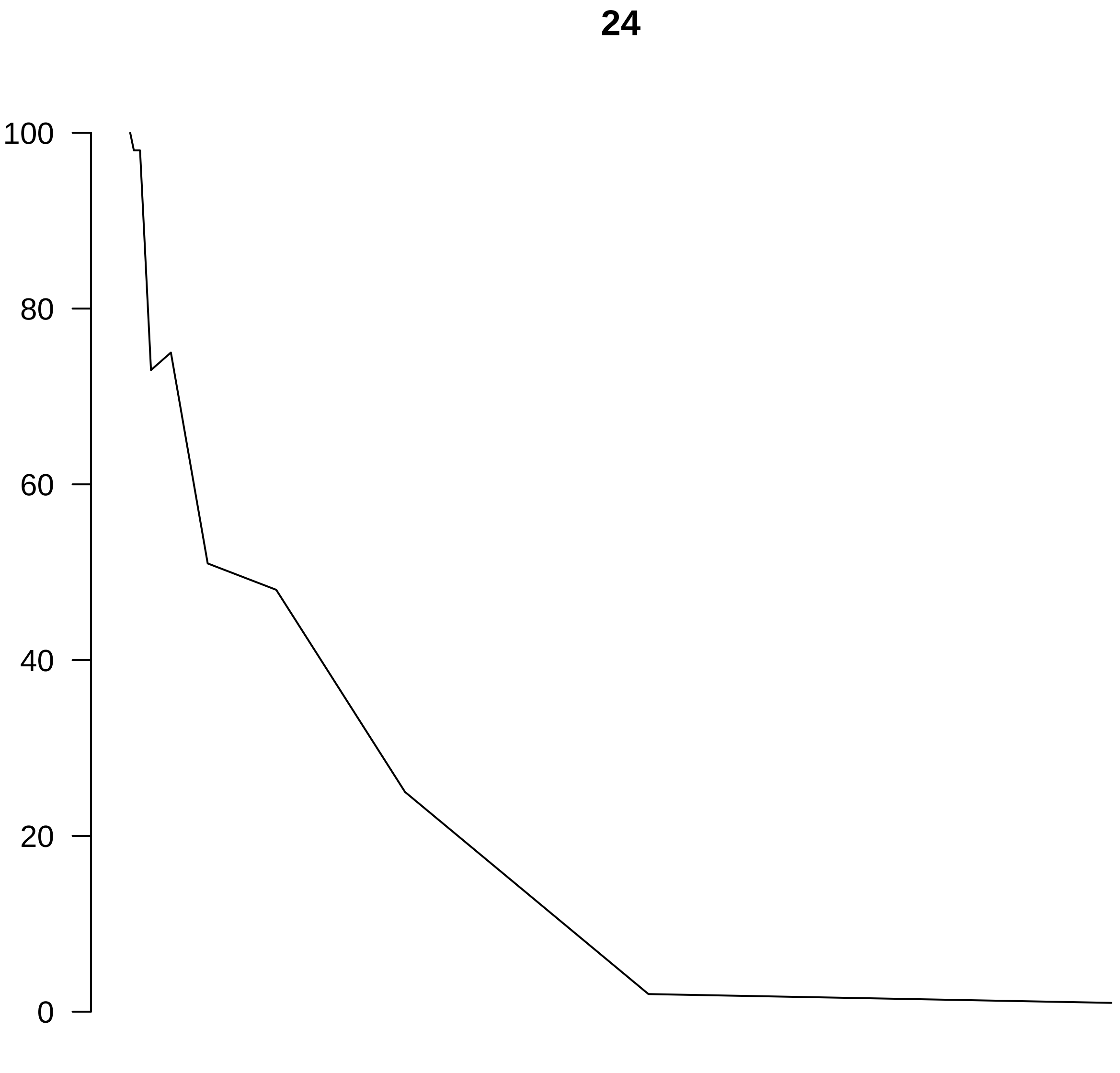

participant was in isolated BF condition
classified as exponential

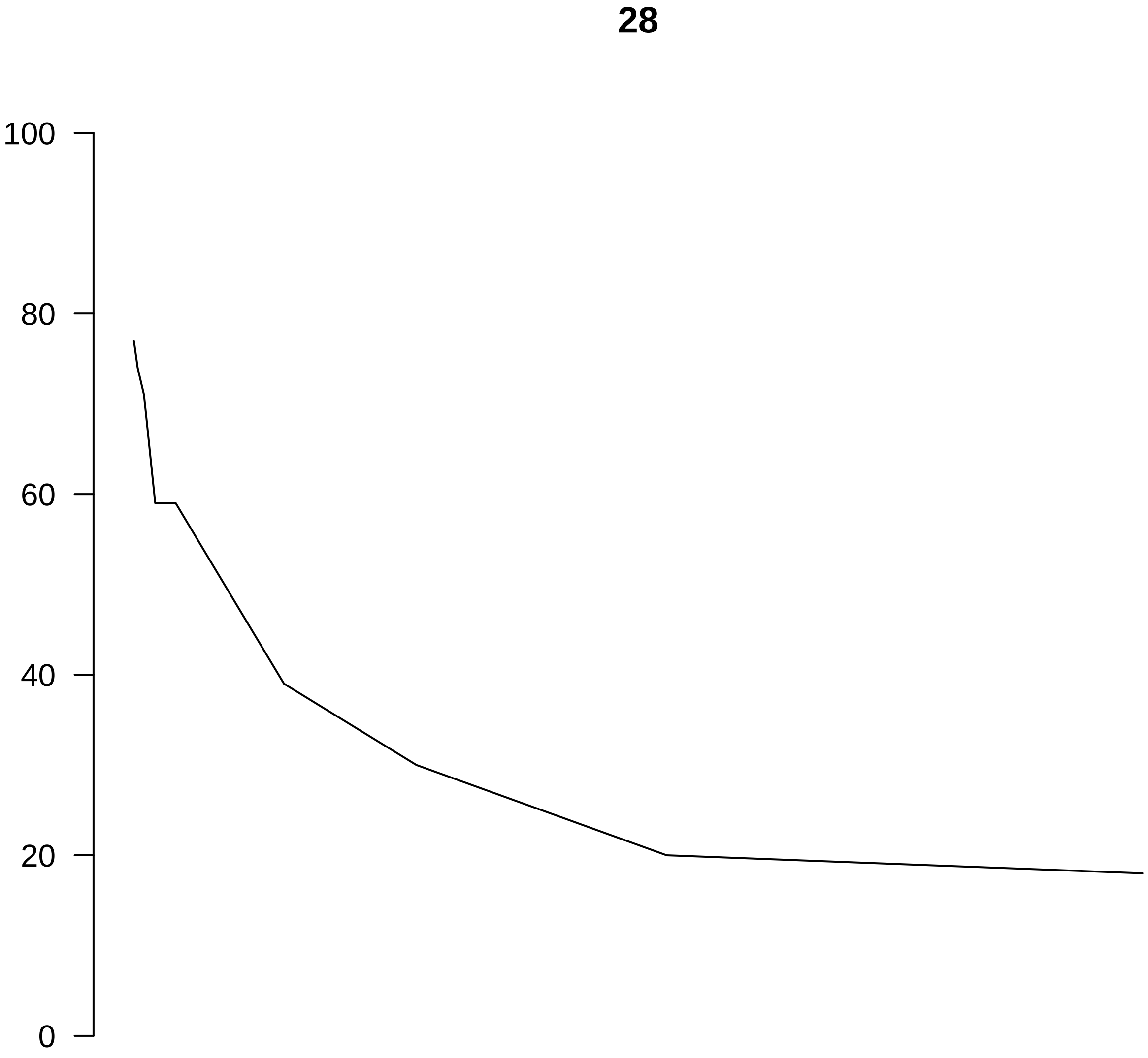

participant was in isolated BF condition
classified as exponential

31

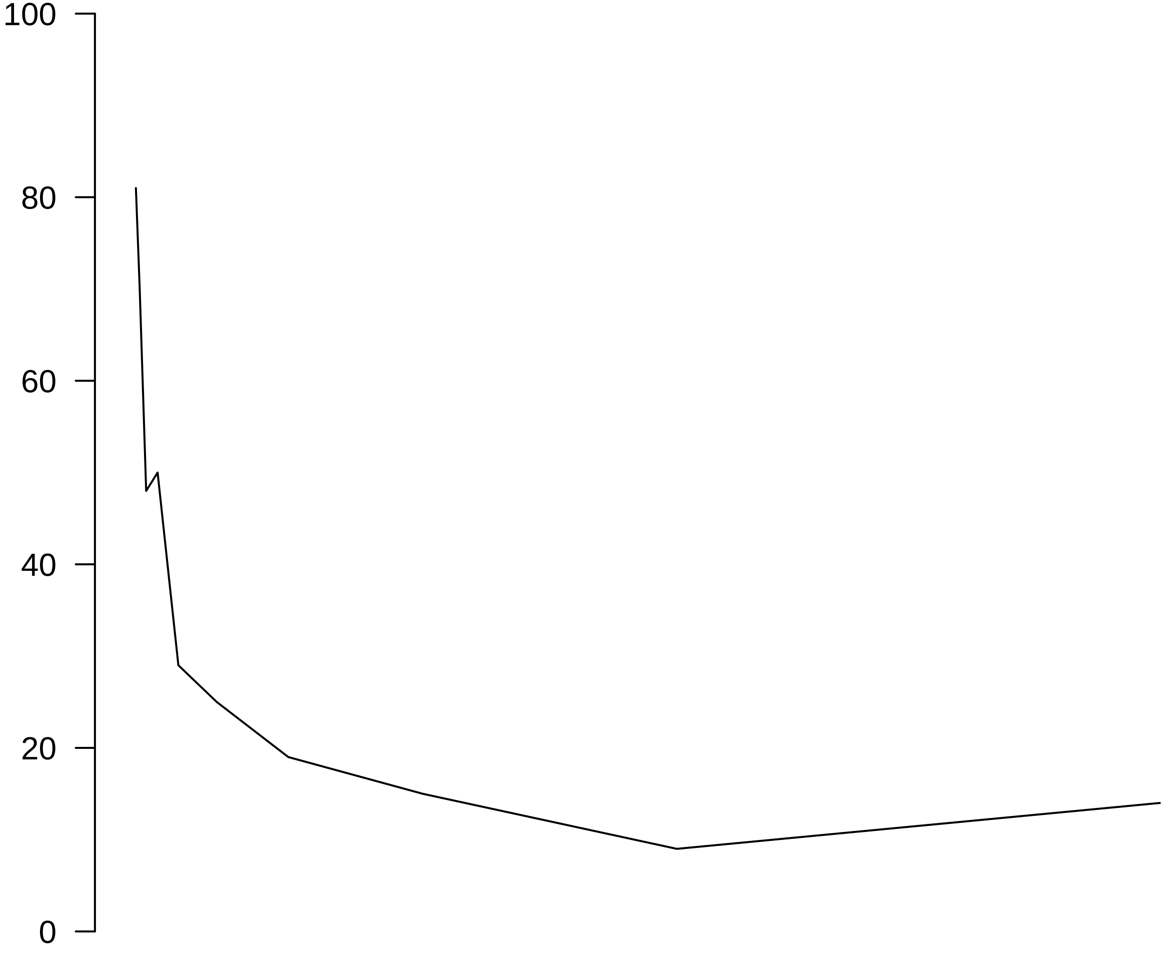

participant was in isolated BF condition
classified as exponential

34

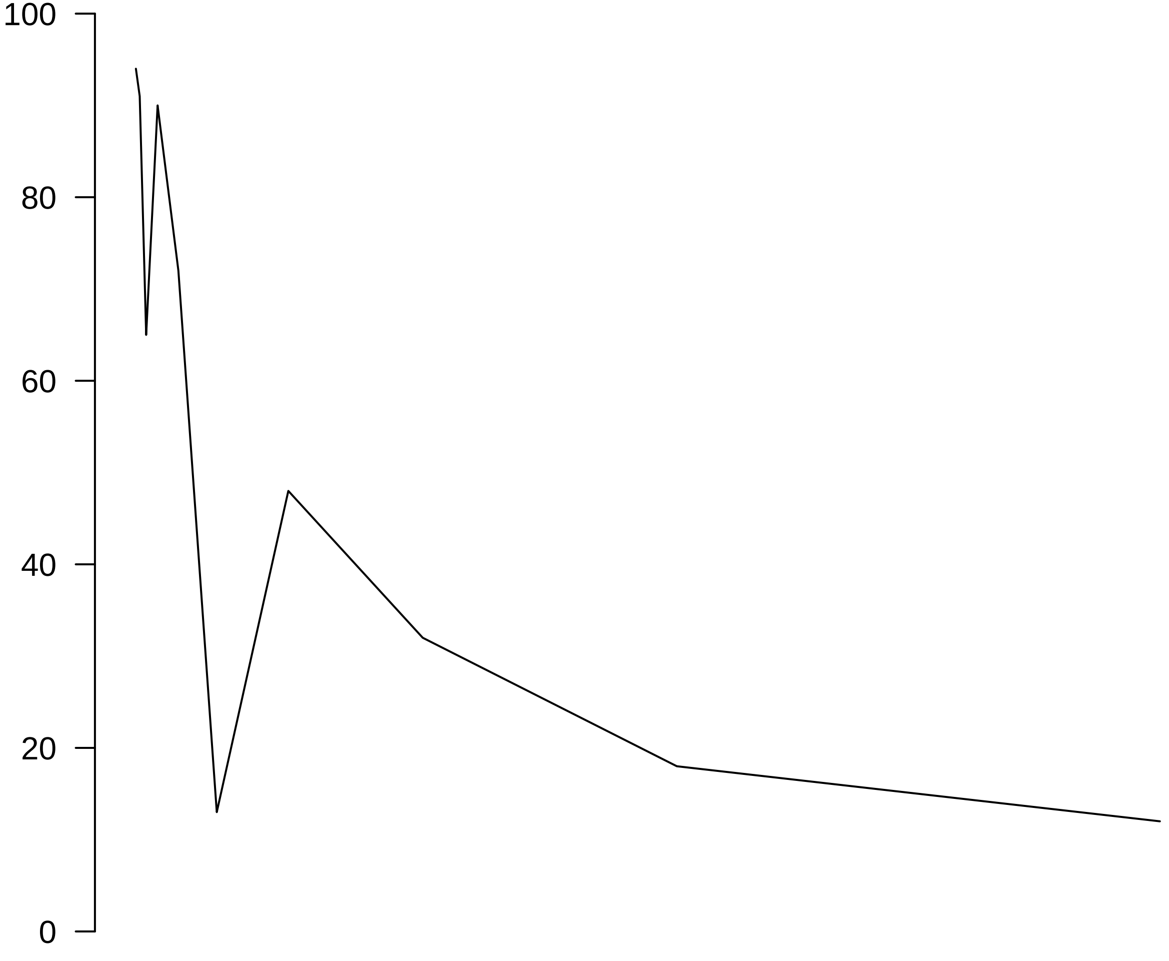

participant was in isolated BF condition
classified as rest

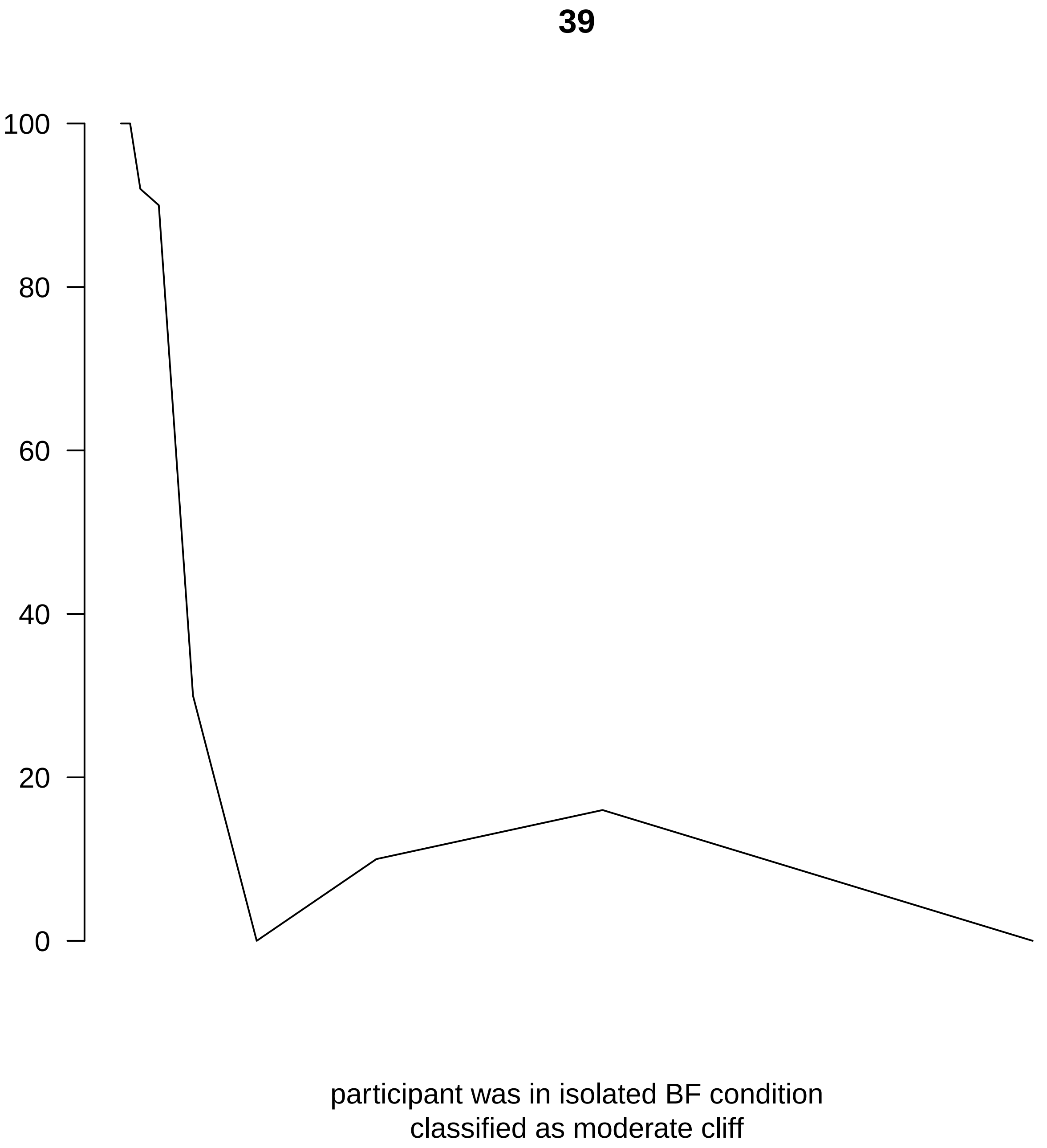

39
100
80
60
40
20
0
participant was in isolated BF condition
classified as moderate cliff

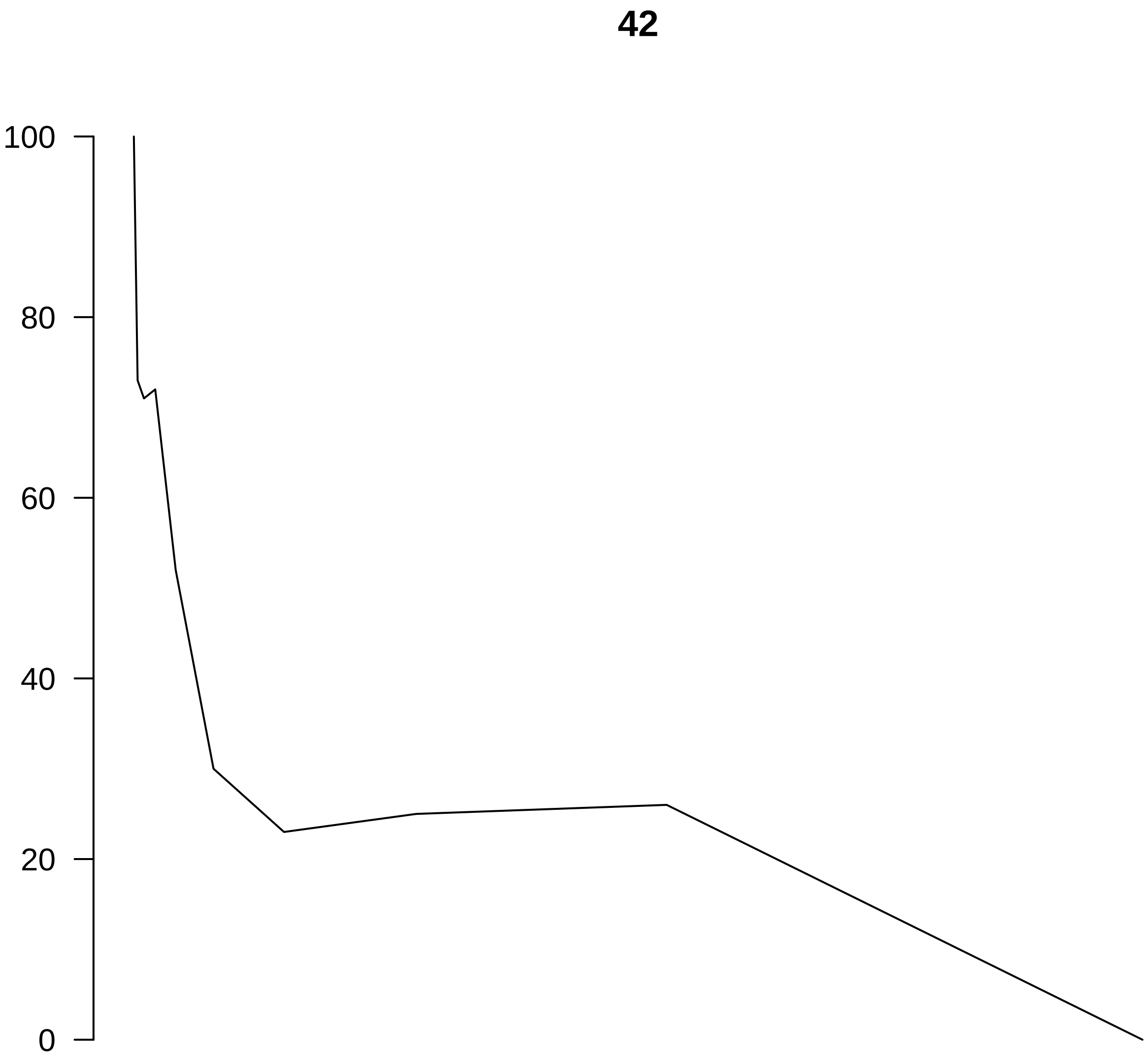

participant was in isolated BF condition
classified as exponential

45

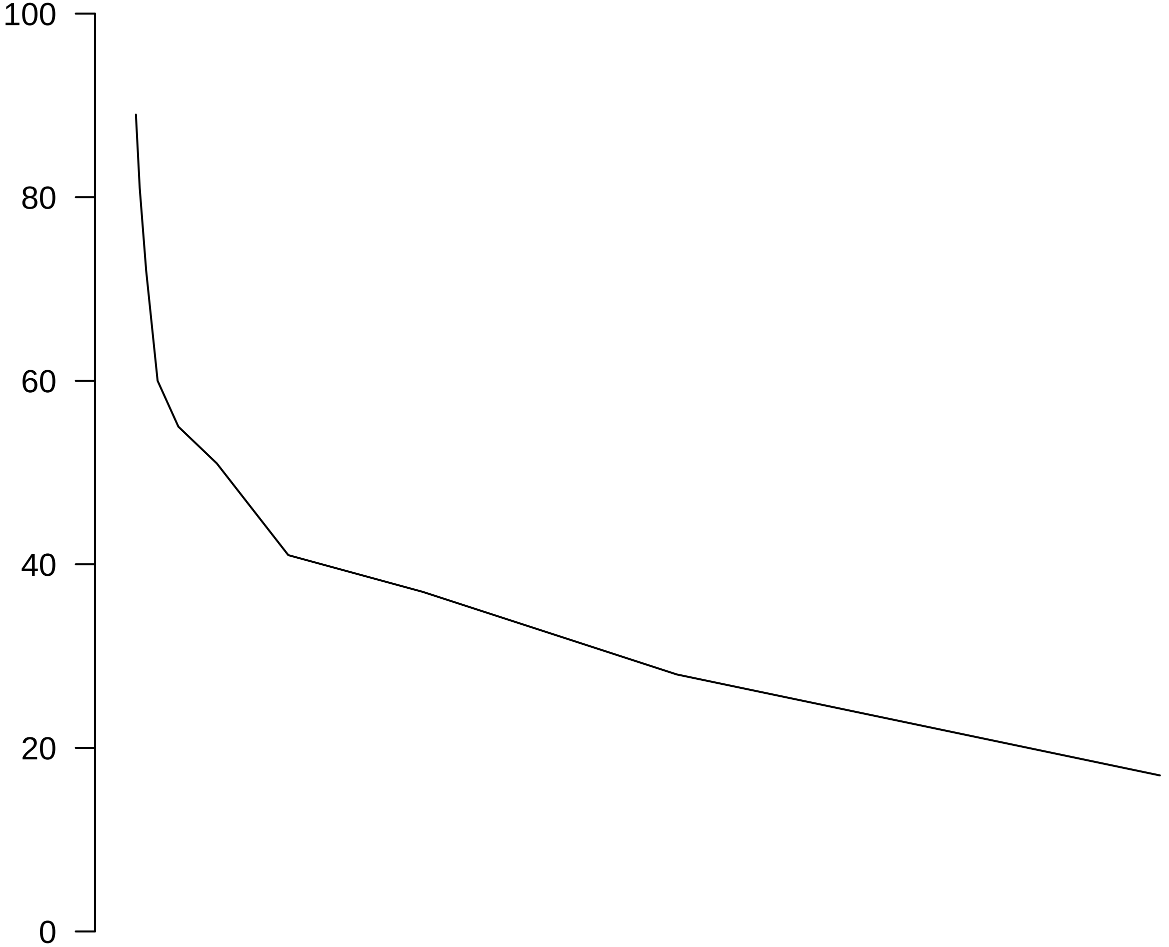

participant was in isolated BF condition
classified as exponential

51

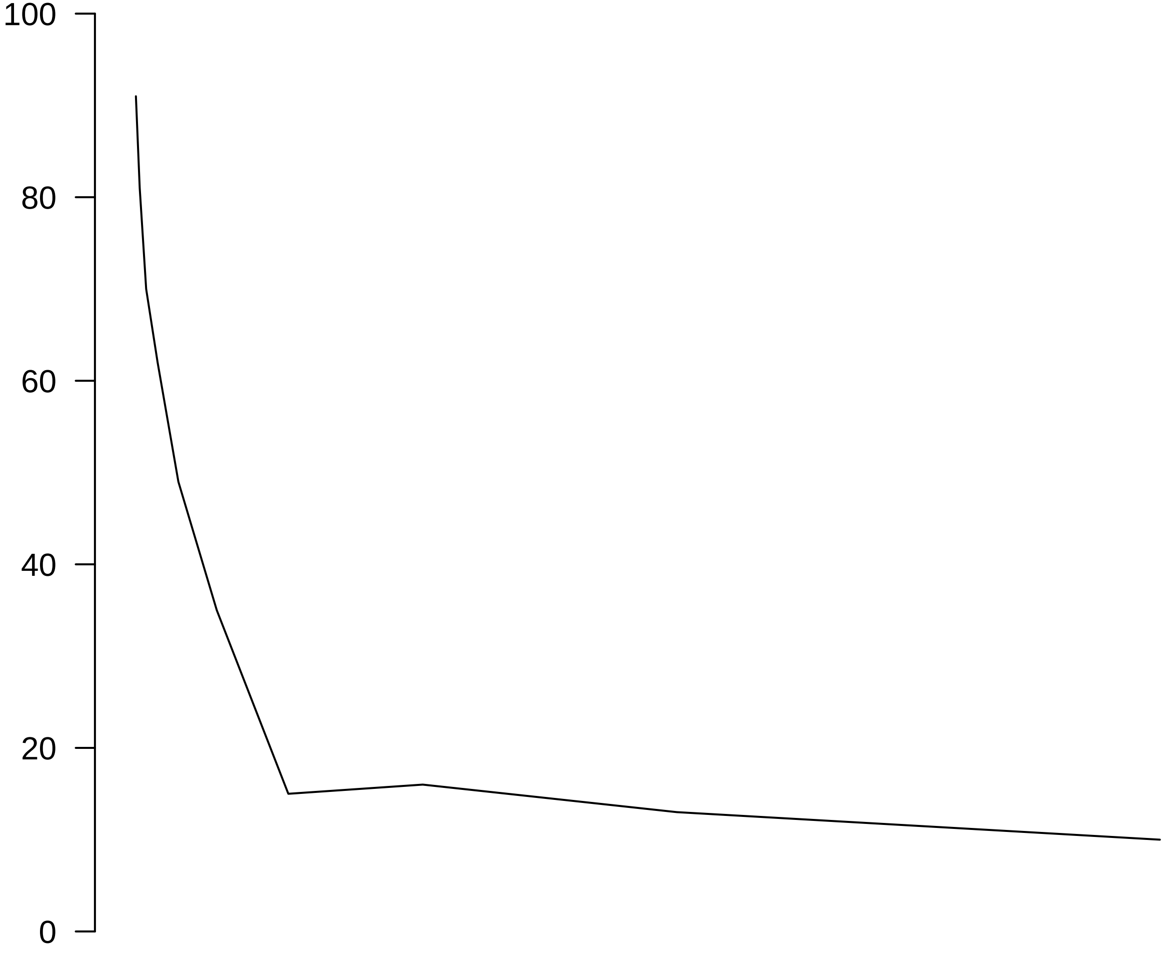

participant was in isolated BF condition
classified as exponential

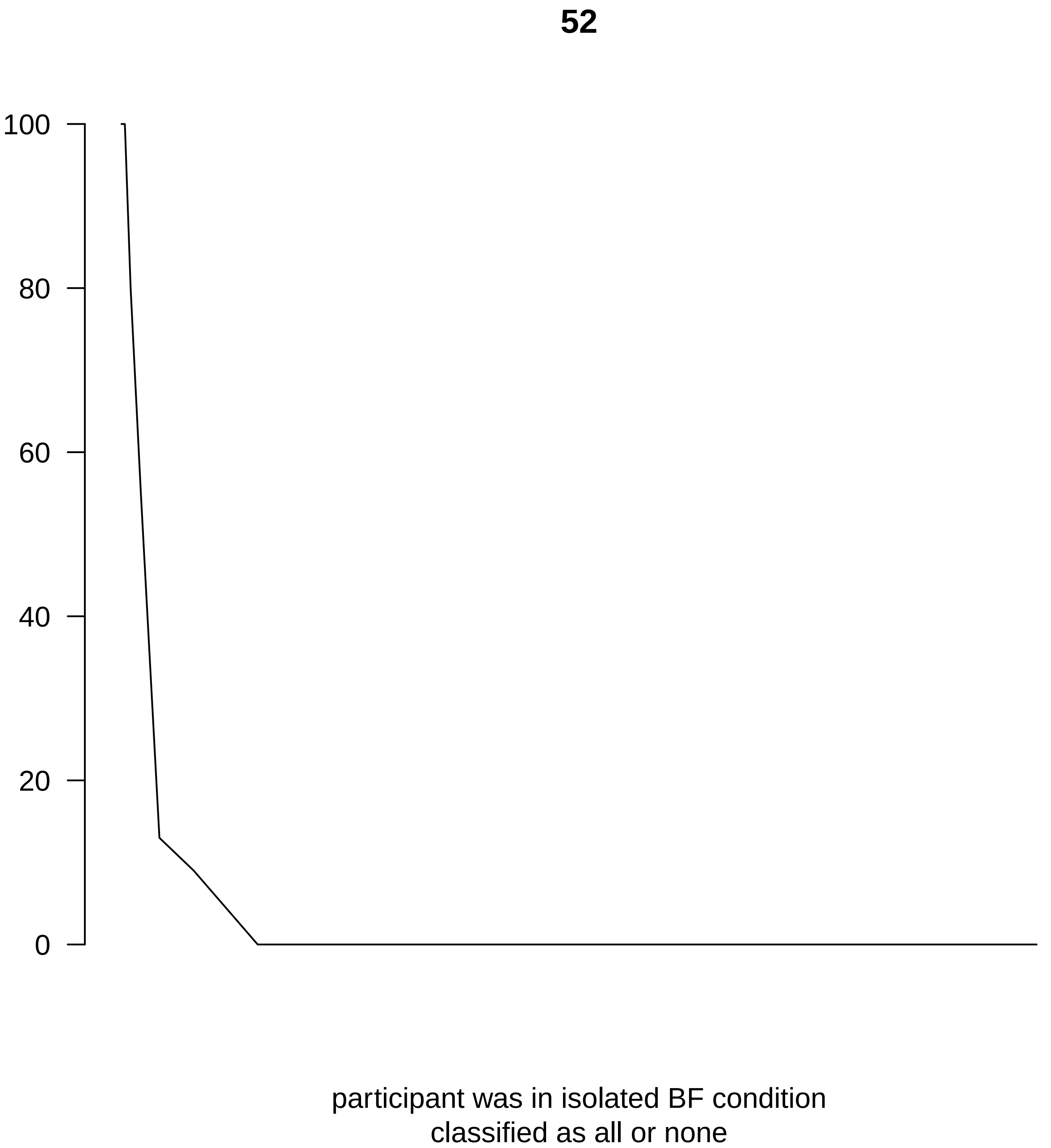
52
100
80
60
40
20
0
participant was in isolated BF condition
classified as all or none

56

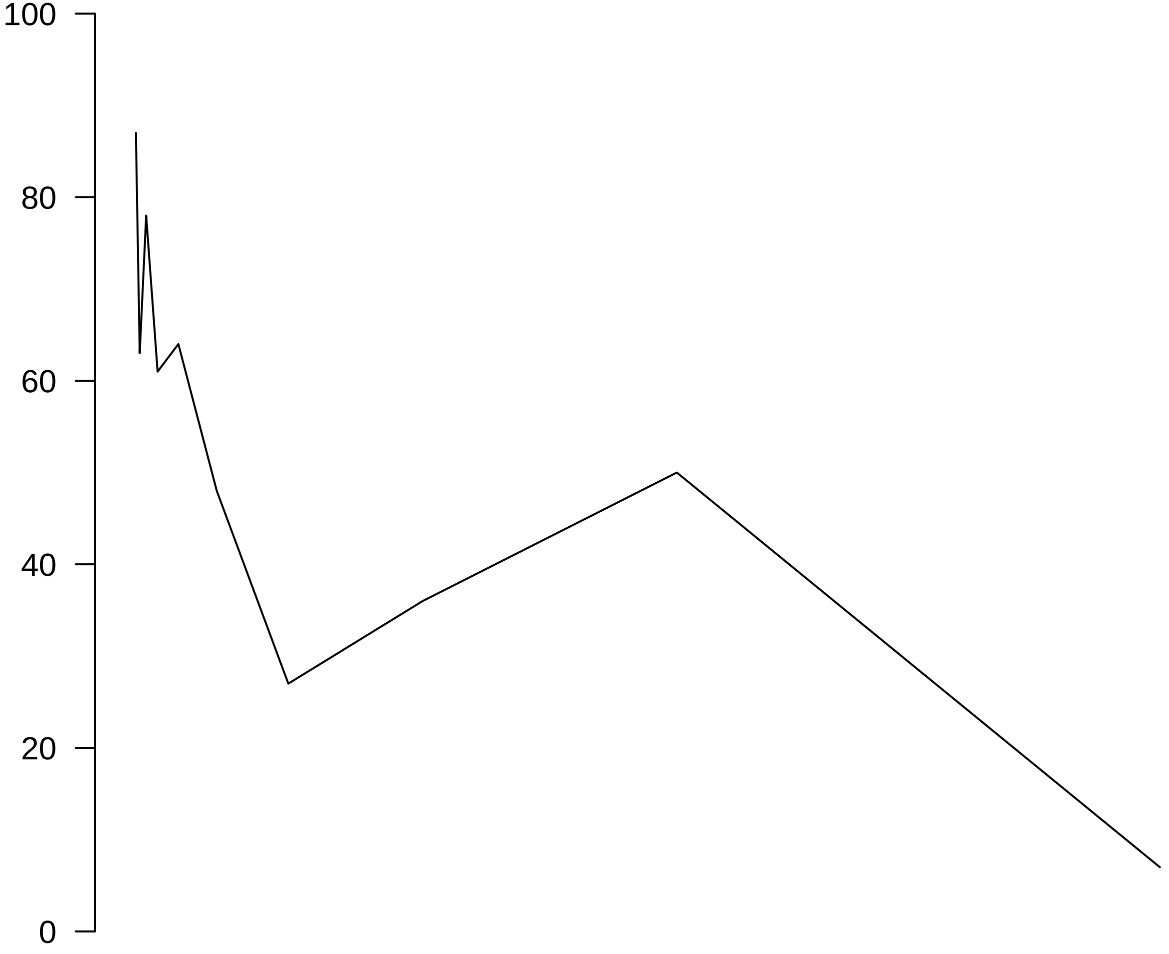

participant was in isolated BF condition
classified as rest

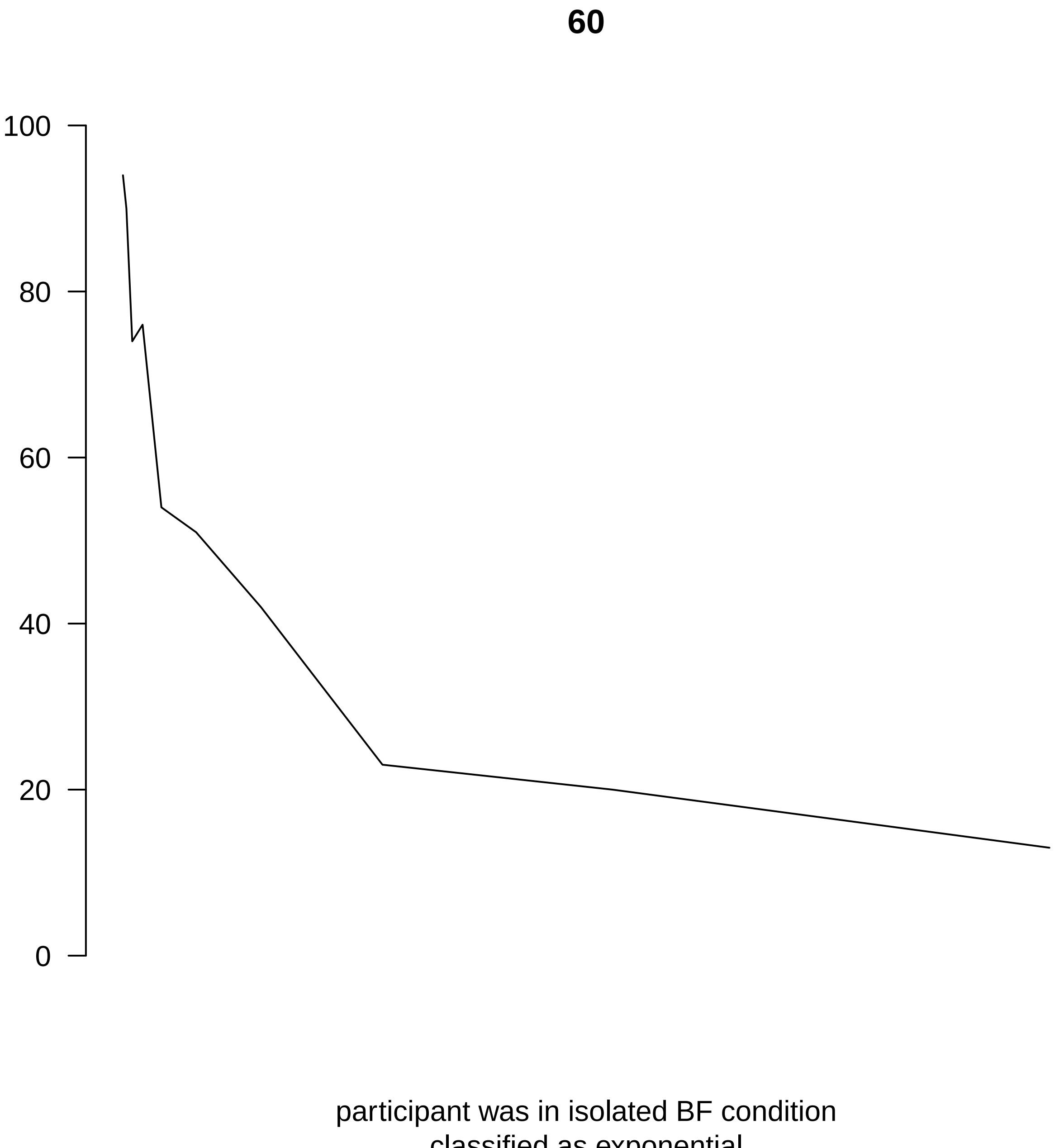
60
100
80
60
40
20
0
participant was in isolated BF condition
classified as exponential

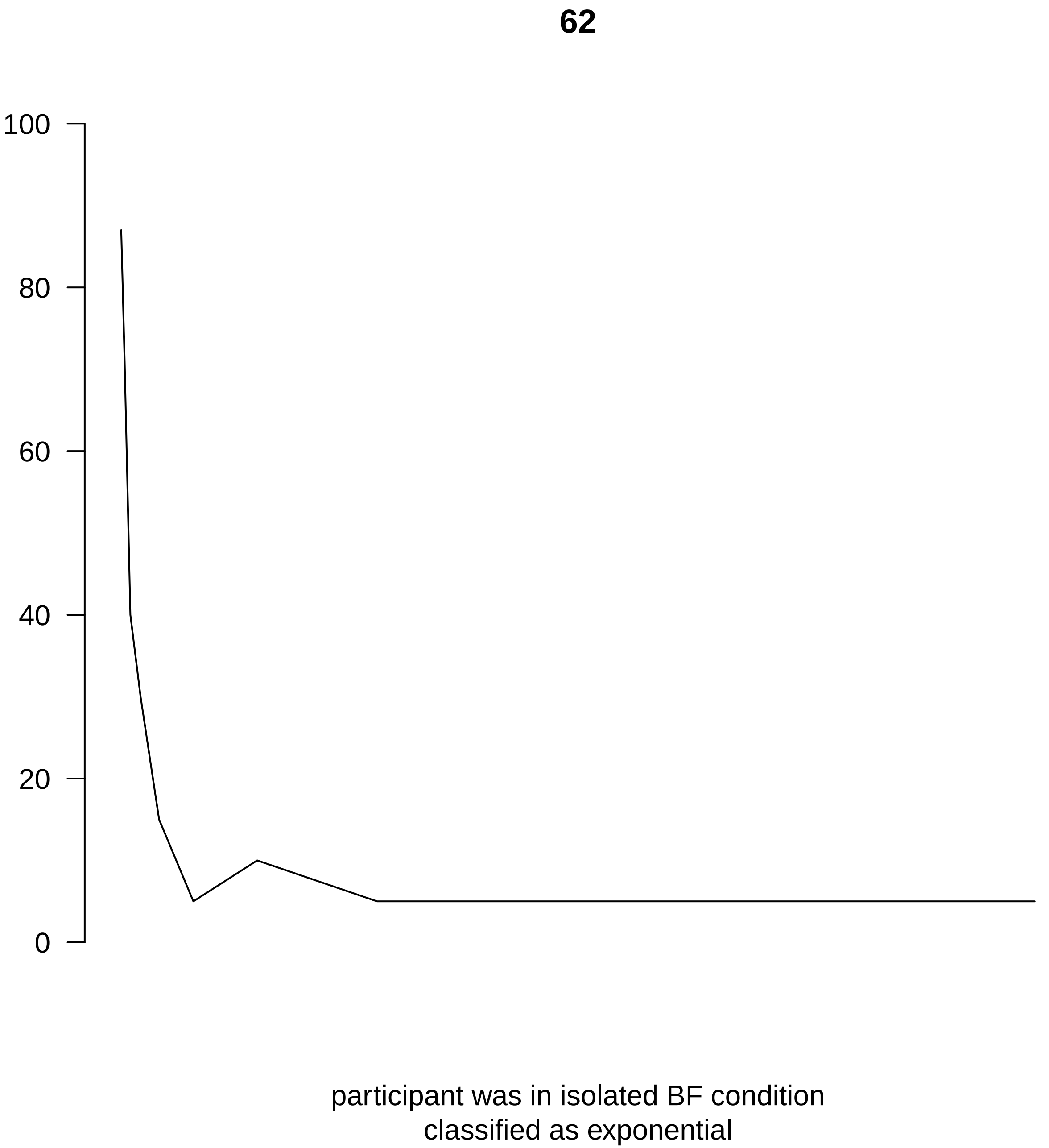
62
100
80
60
40
20
0
participant was in isolated BF condition
classified as exponential

68

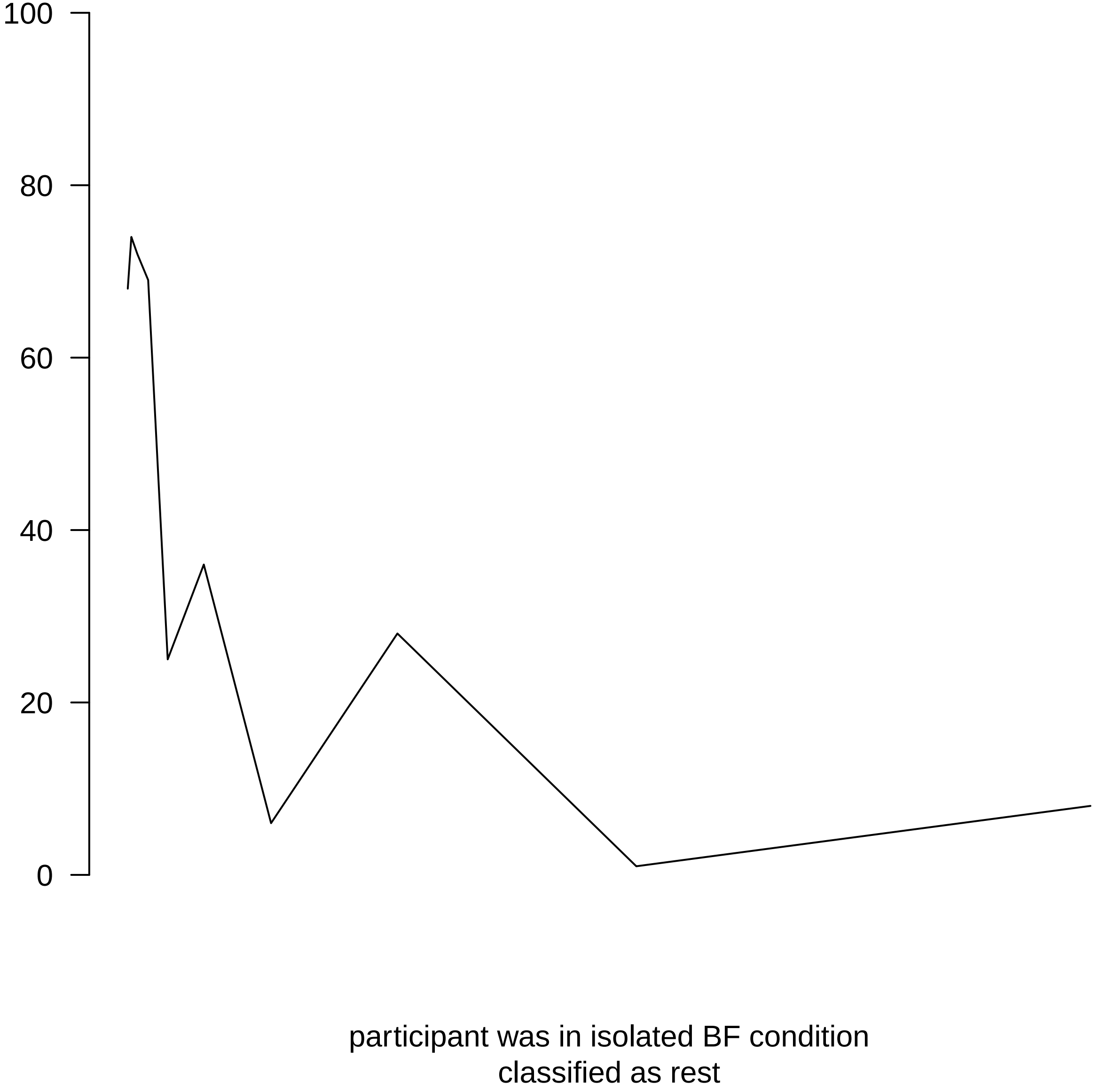
100
80
60
40
20
0
participant was in isolated BF condition
classified as rest

70

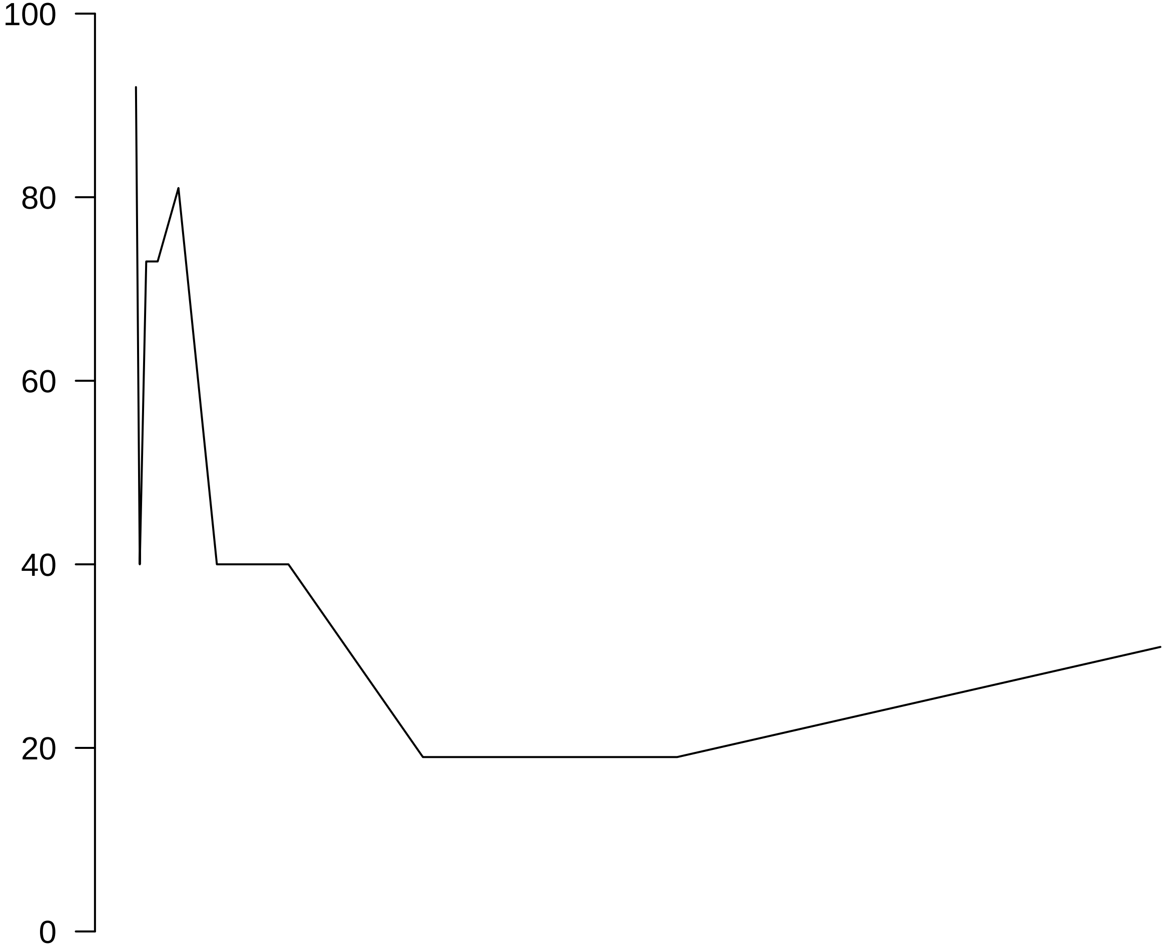

participant was in isolated BF condition
classified as rest

73

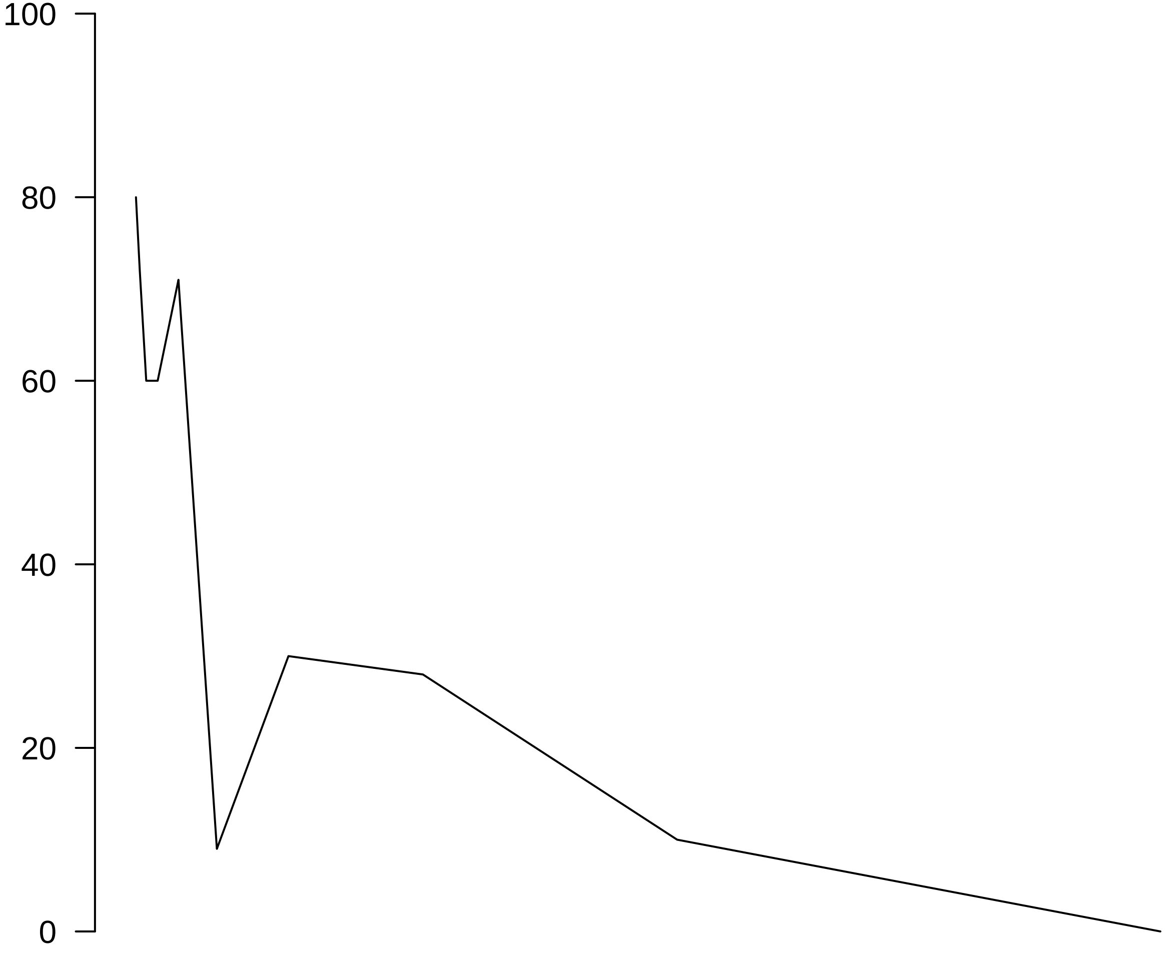

participant was in isolated BF condition
classified as moderate cliff

78

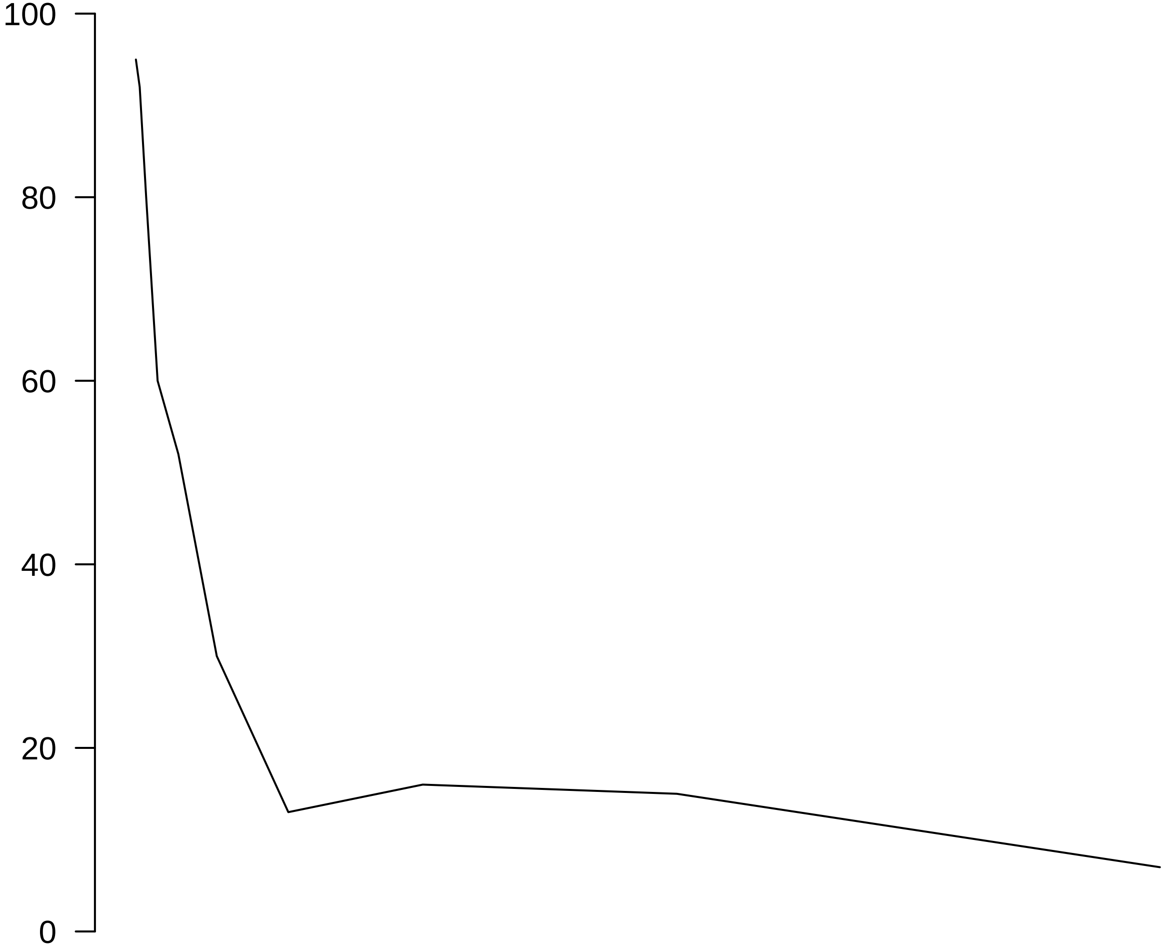

participant was in isolated BF condition
classified as exponential

79

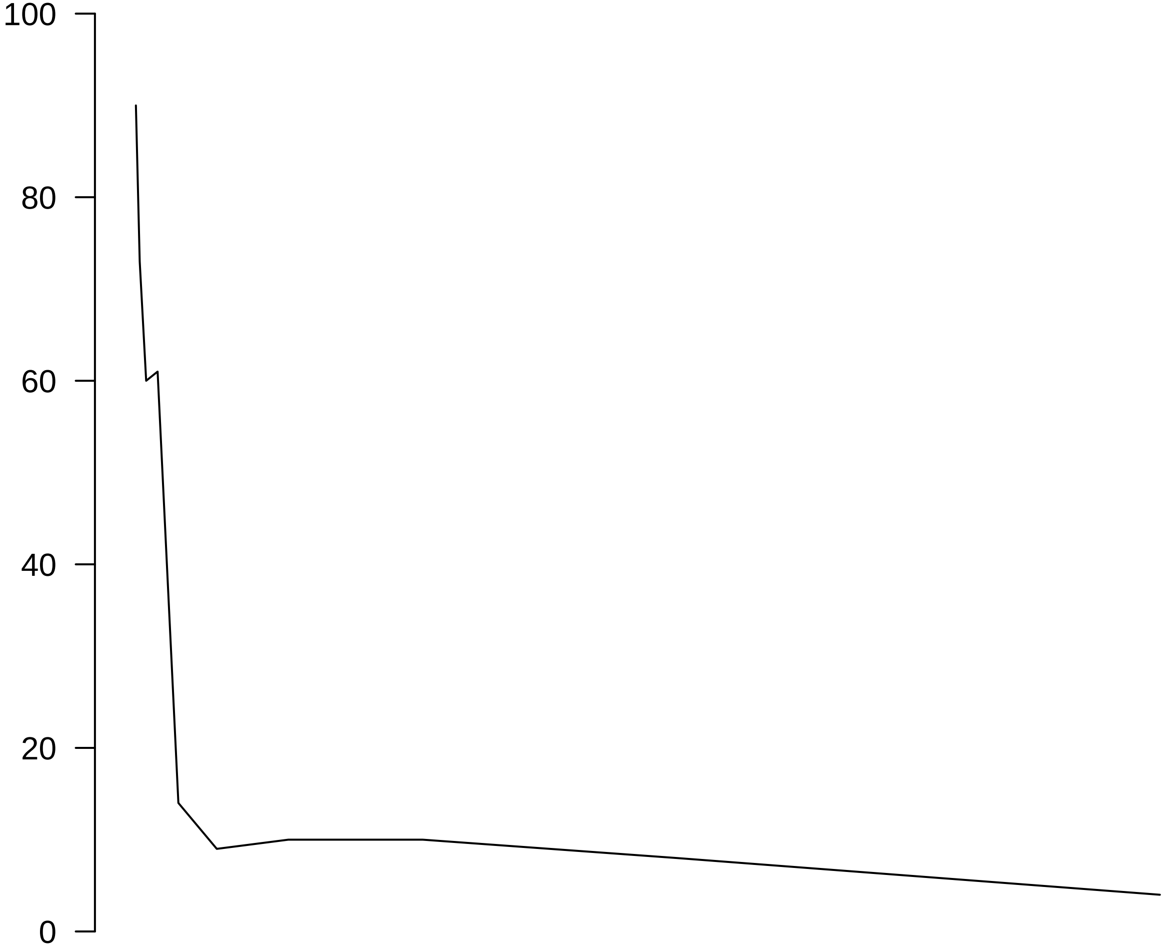

participant was in isolated BF condition
classified as exponential

83

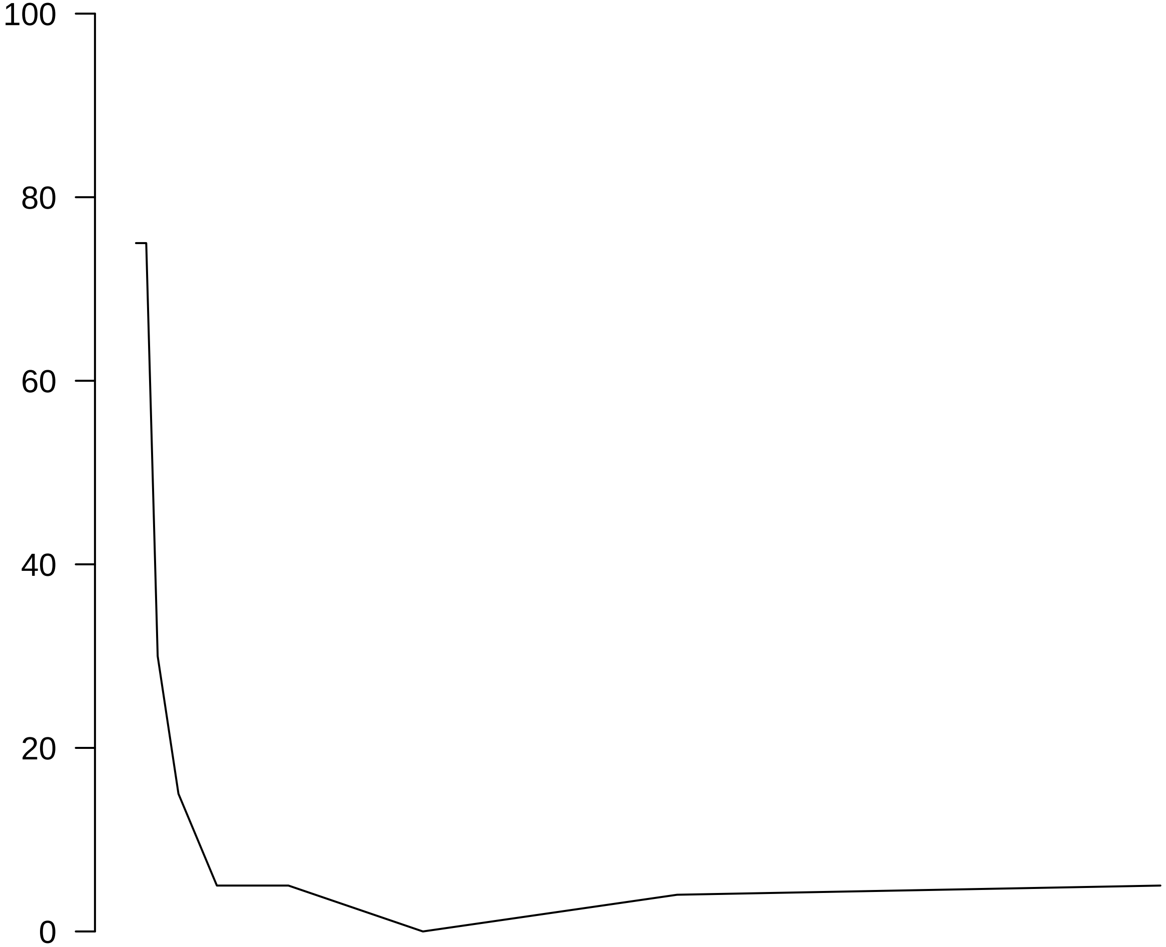

participant was in isolated BF condition
classified as exponential

87

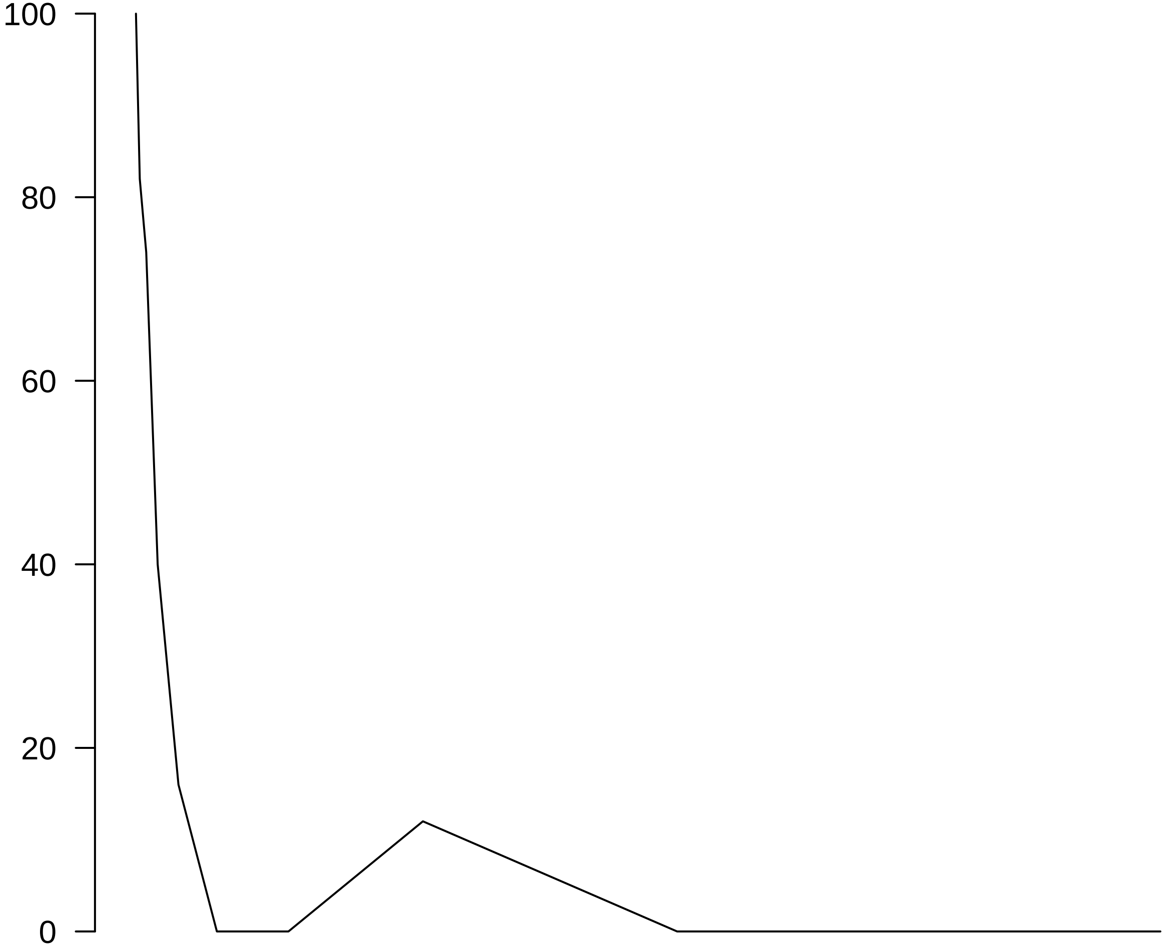

participant was in isolated BF condition
classified as exponential

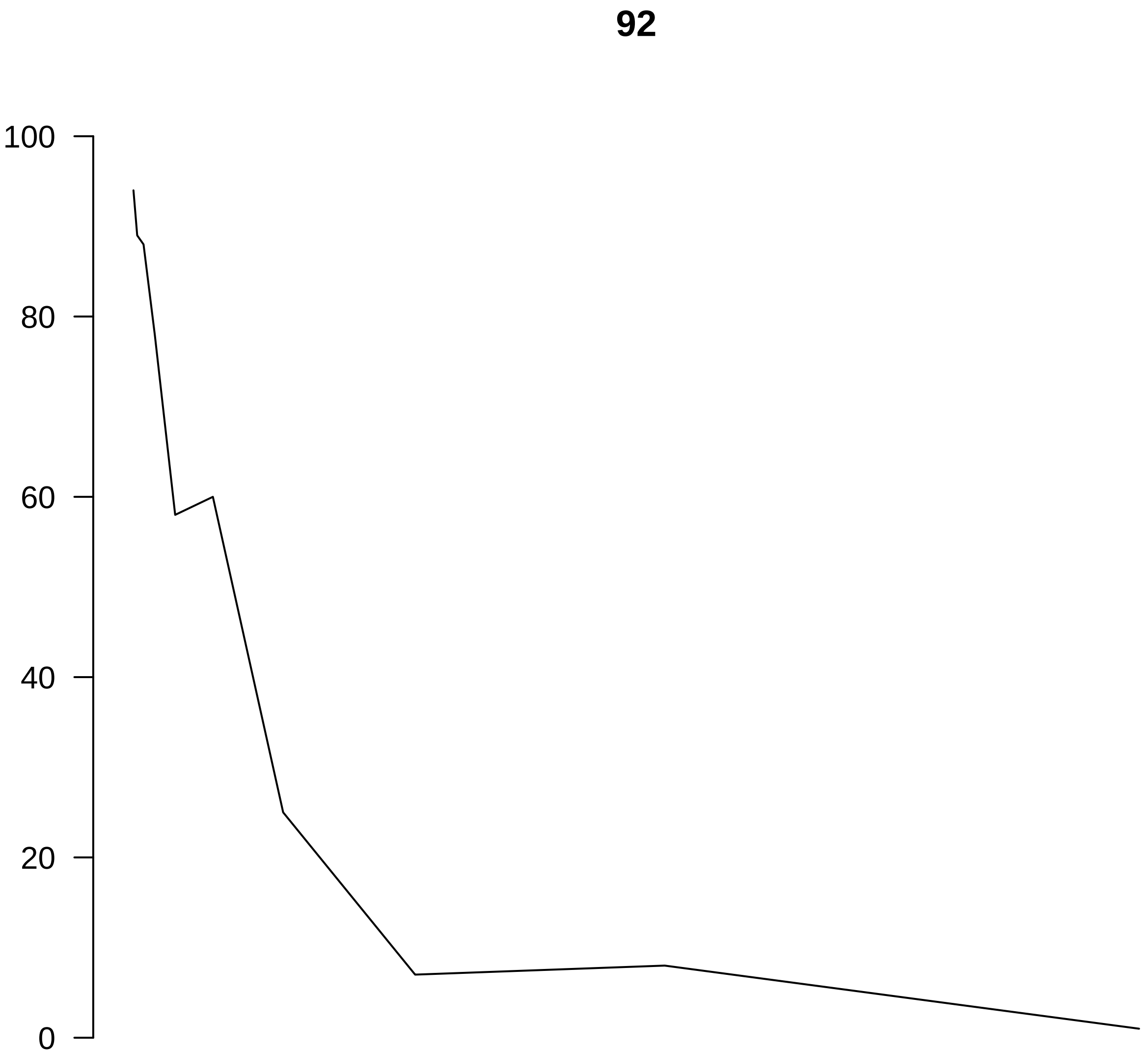

participant was in isolated BF condition
classified as exponential

97

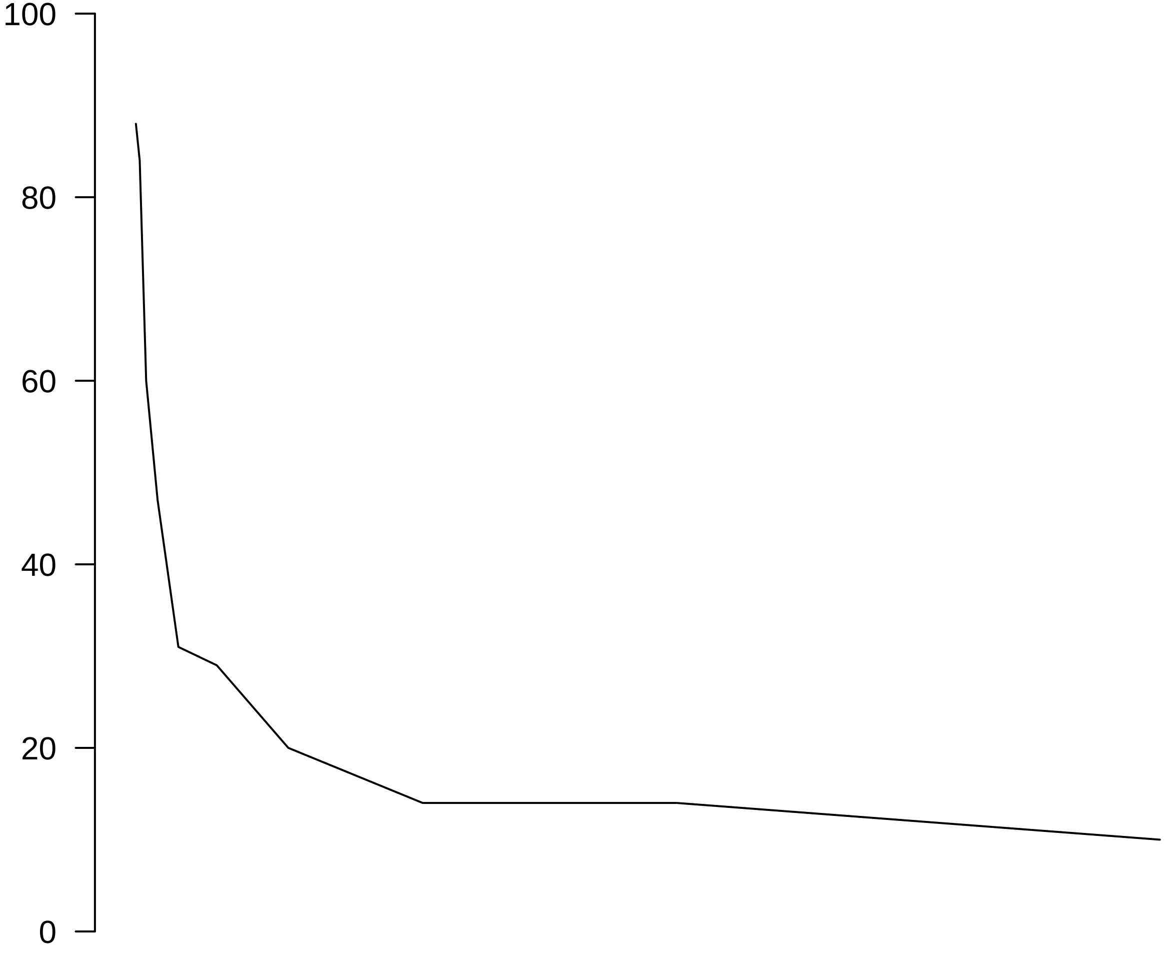

participant was in isolated BF condition
classified as exponential

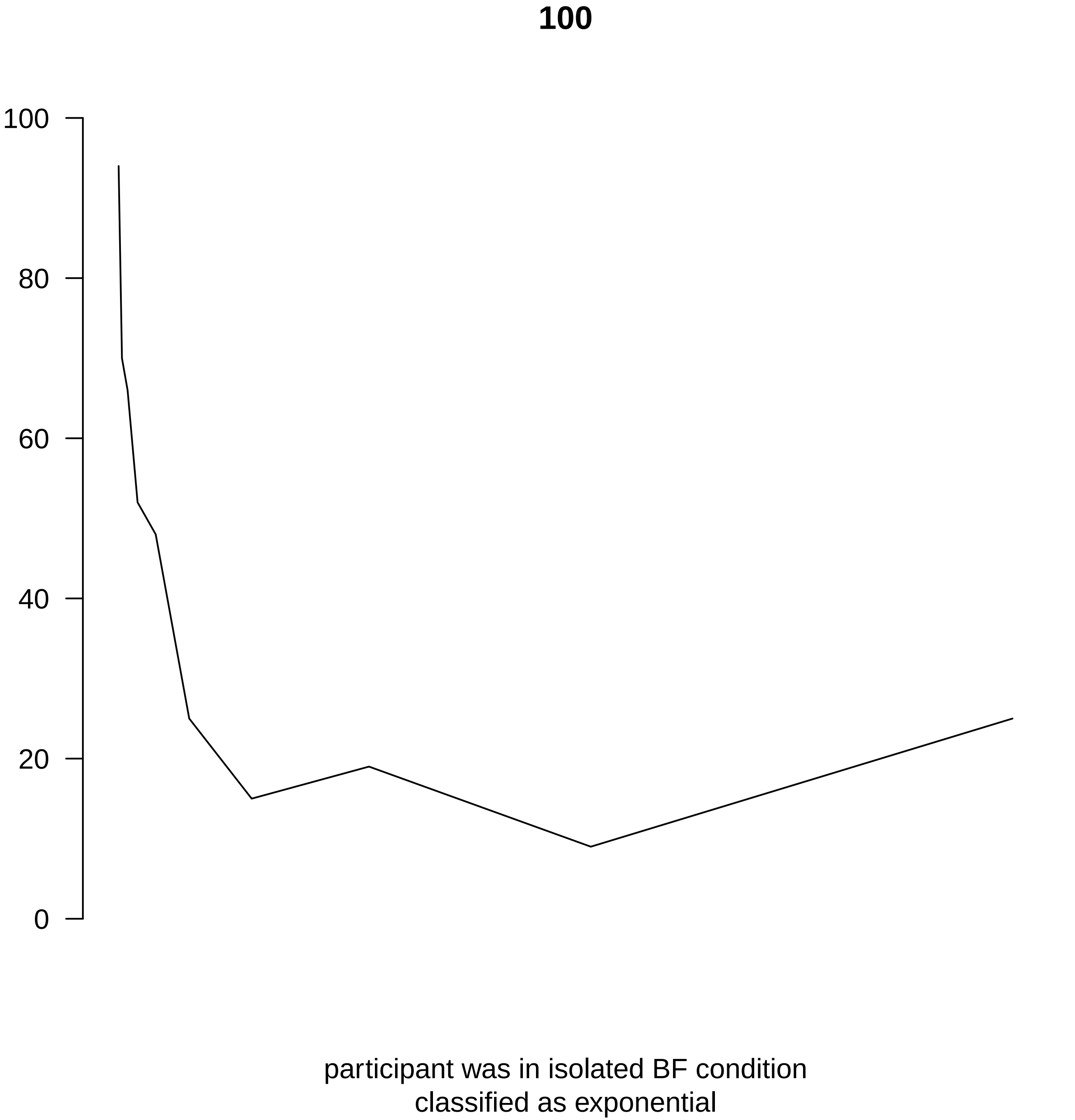
100
100
80
60
40
20
0
participant was in isolated BF condition
classified as exponential

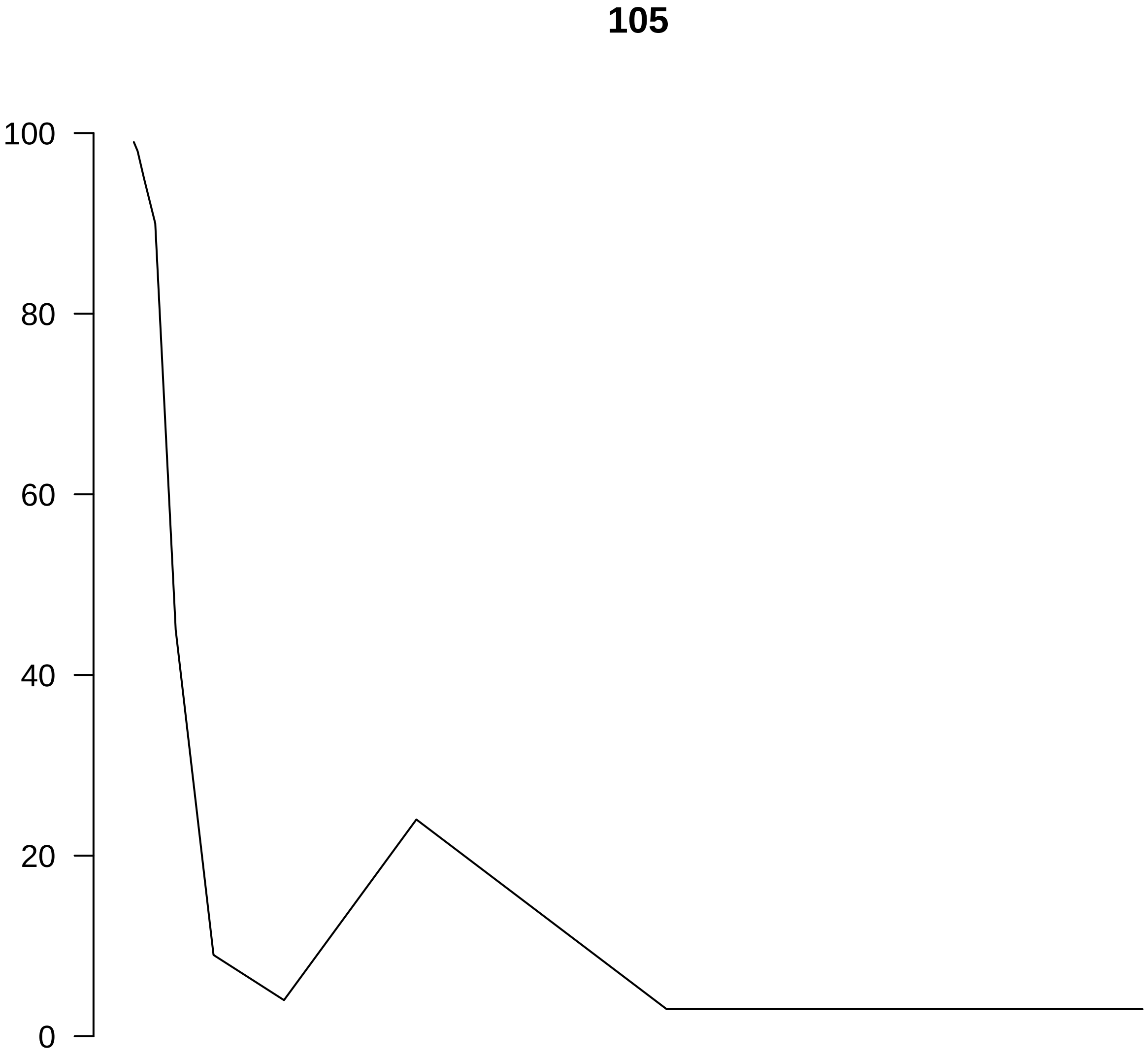

participant was in isolated BF condition
classified as exponential

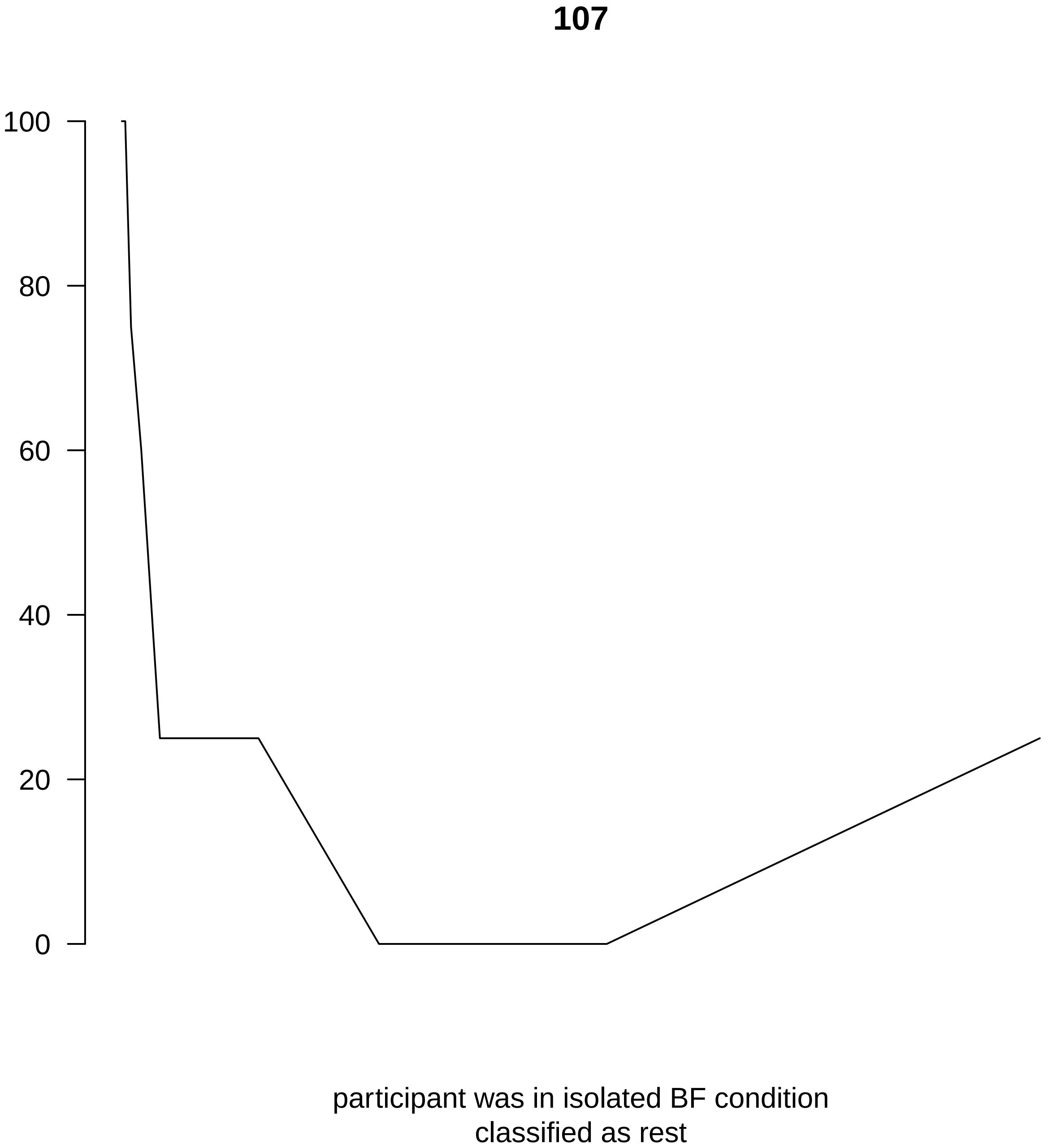
107
100
80
60
40
20
0
participant was in isolated BF condition
classified as rest

113

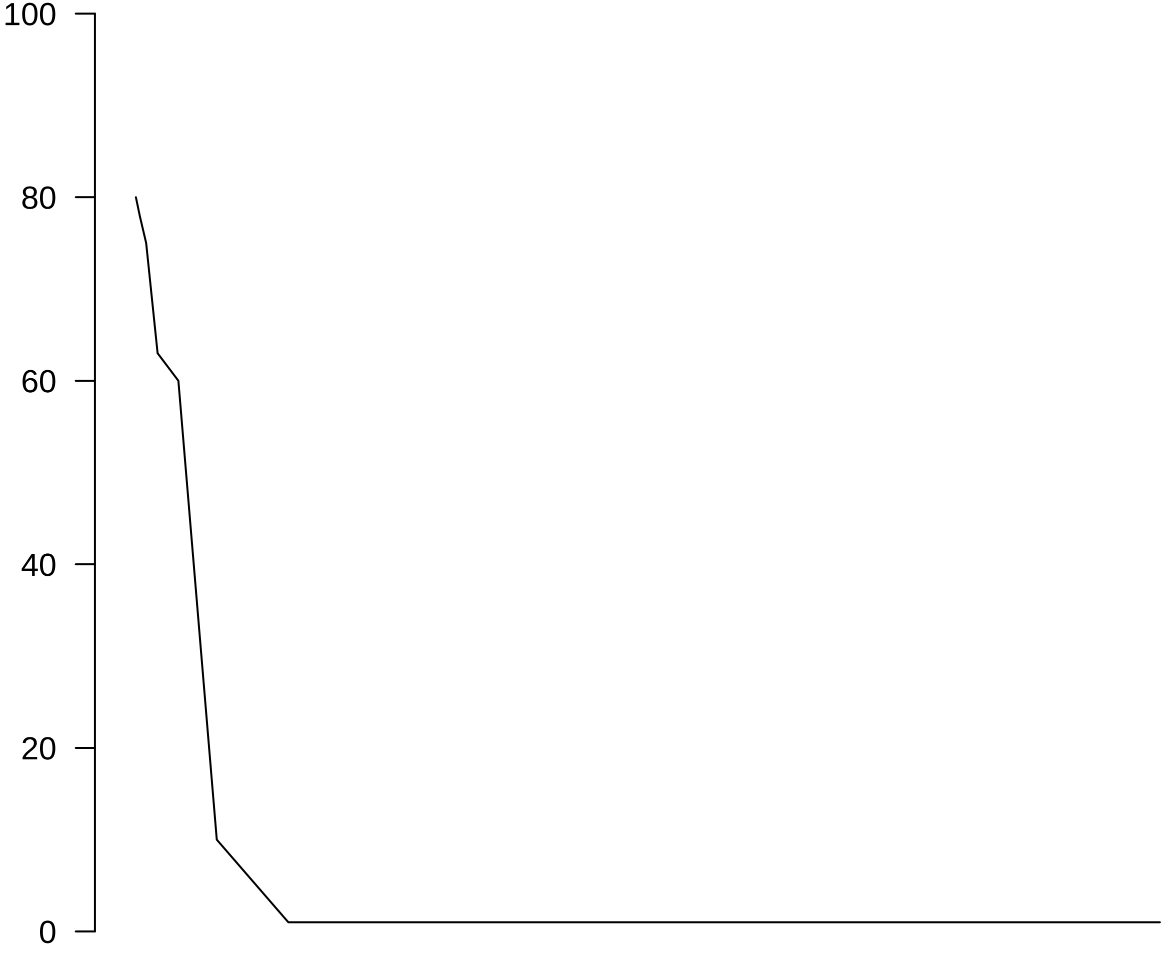

participant was in isolated BF condition
classified as exponential

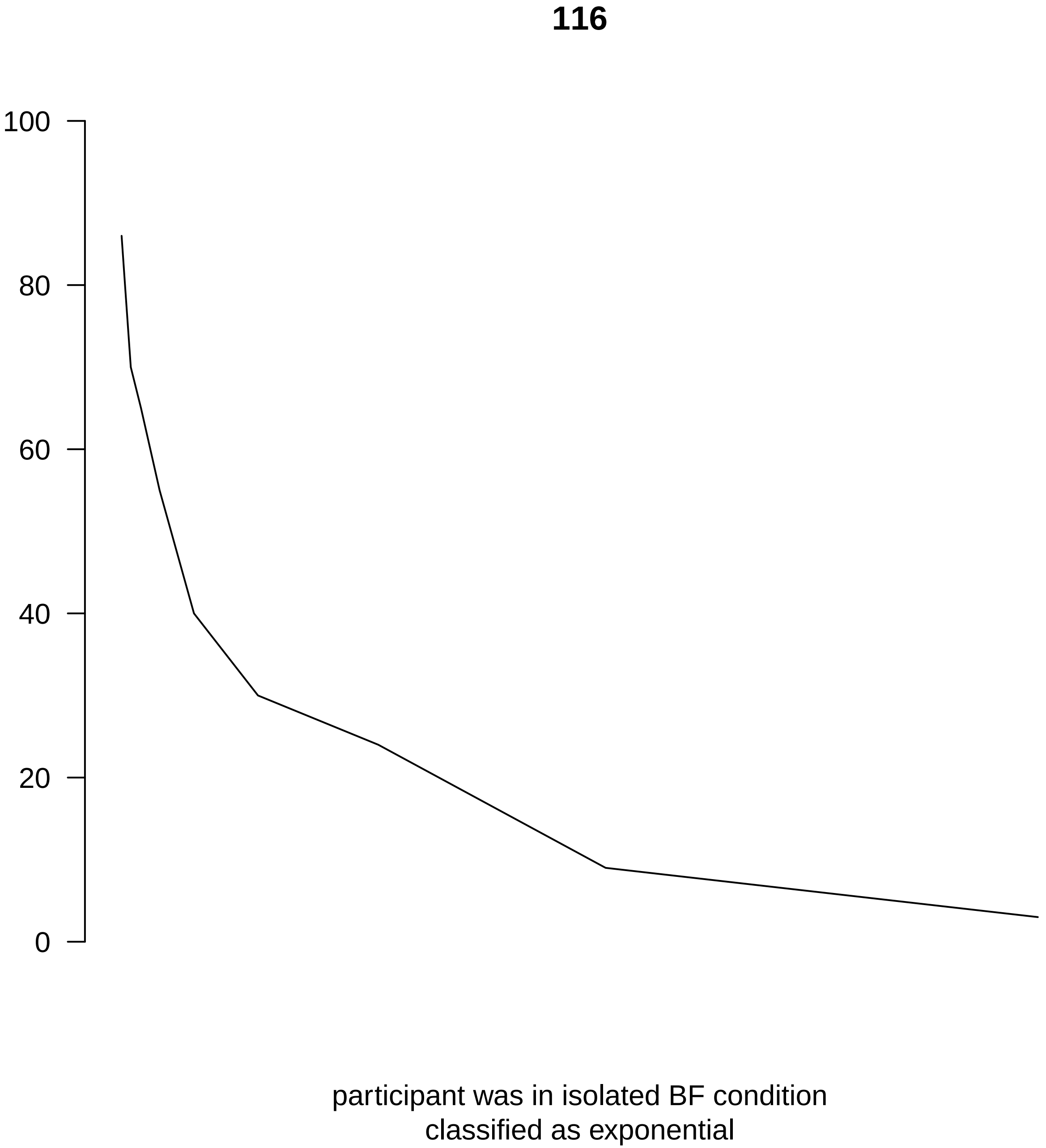
116
100
80
60
40
20
0
participant was in isolated BF condition
classified as exponential

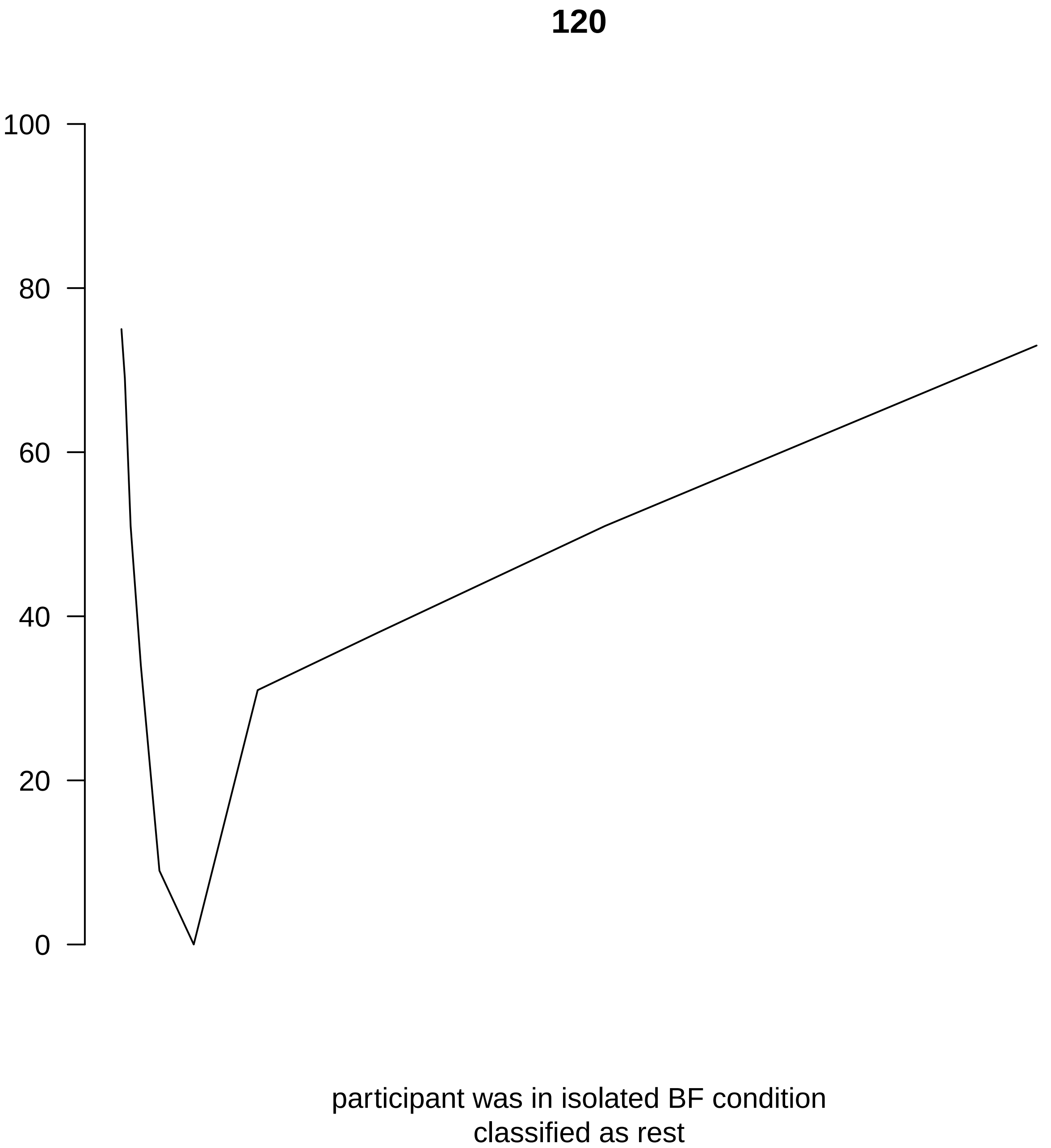
120
100
80
60
40
20
0
participant was in isolated BF condition
classified as rest

124

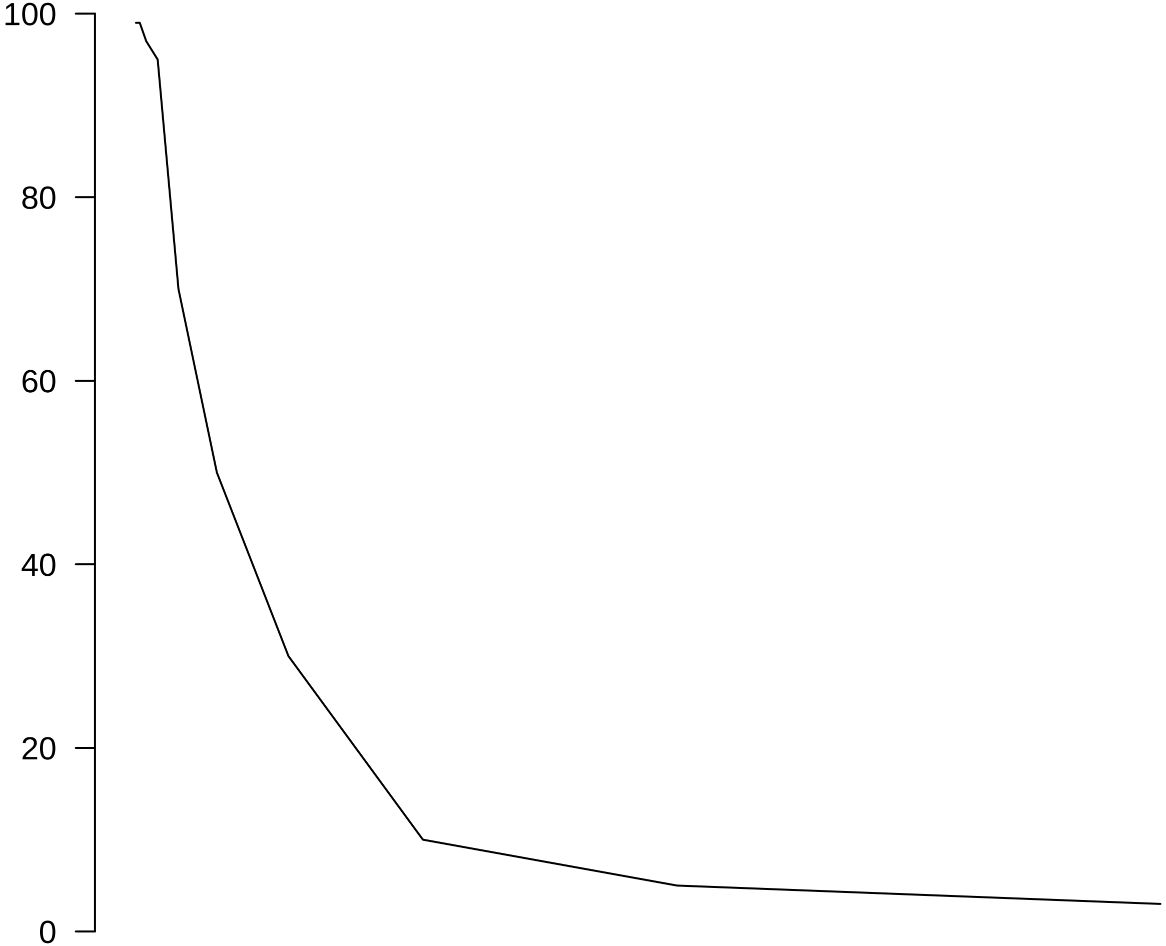

participant was in isolated BF condition
classified as exponential

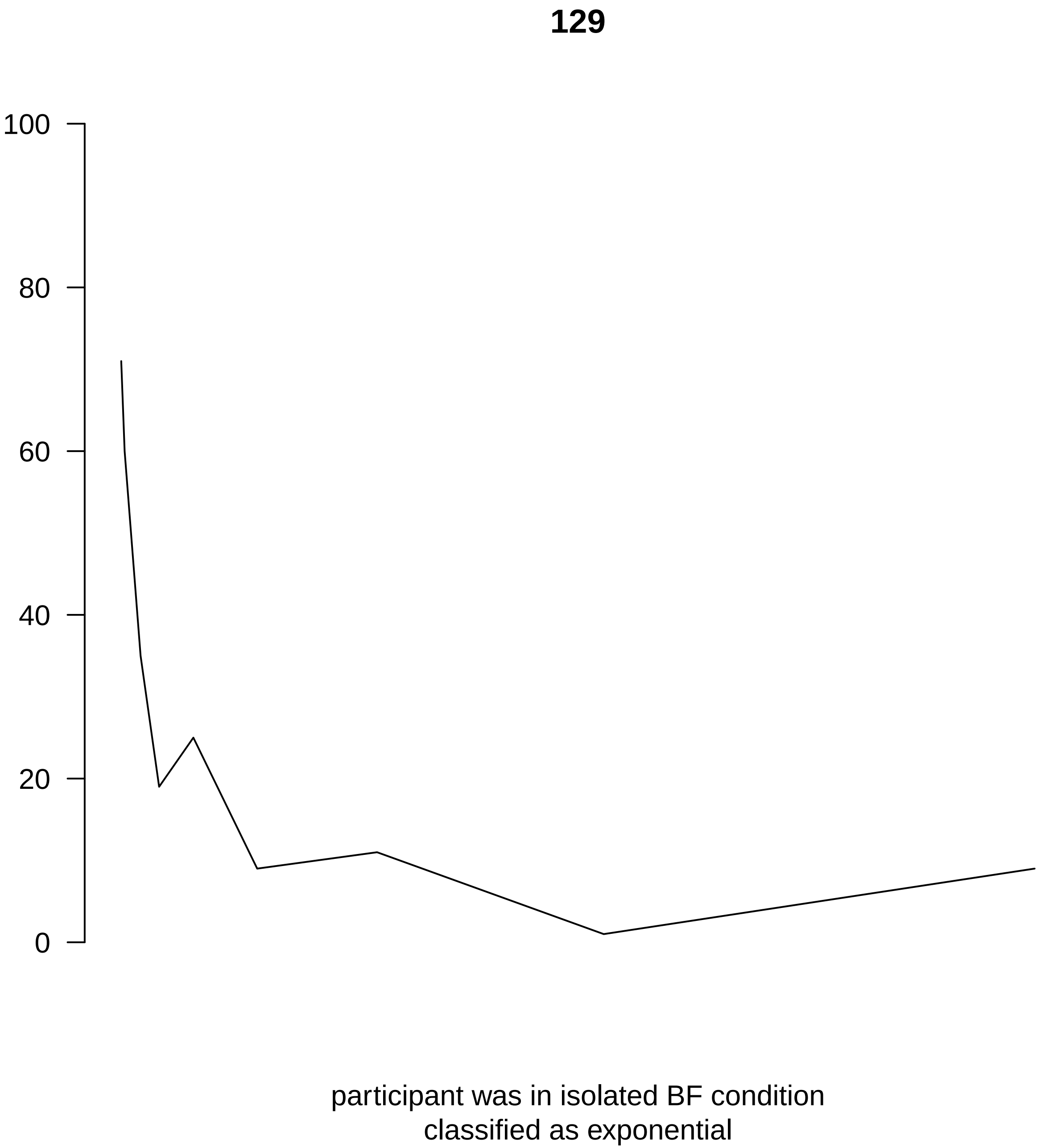
129
100
80
60
40
20
0
participant was in isolated BF condition
classified as exponential

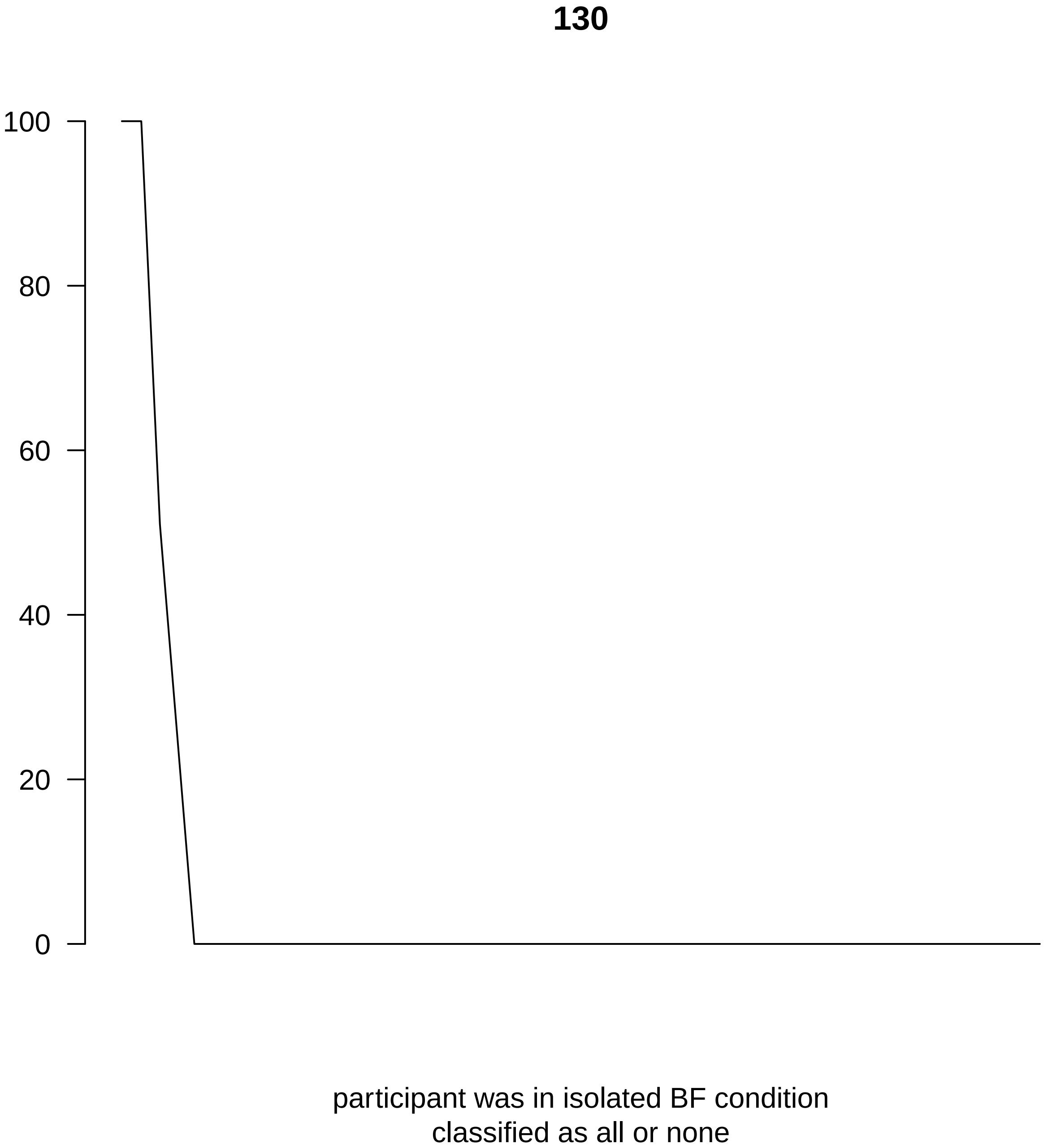
130
100
80
60
40
20
0
participant was in isolated BF condition
classified as all or none

136

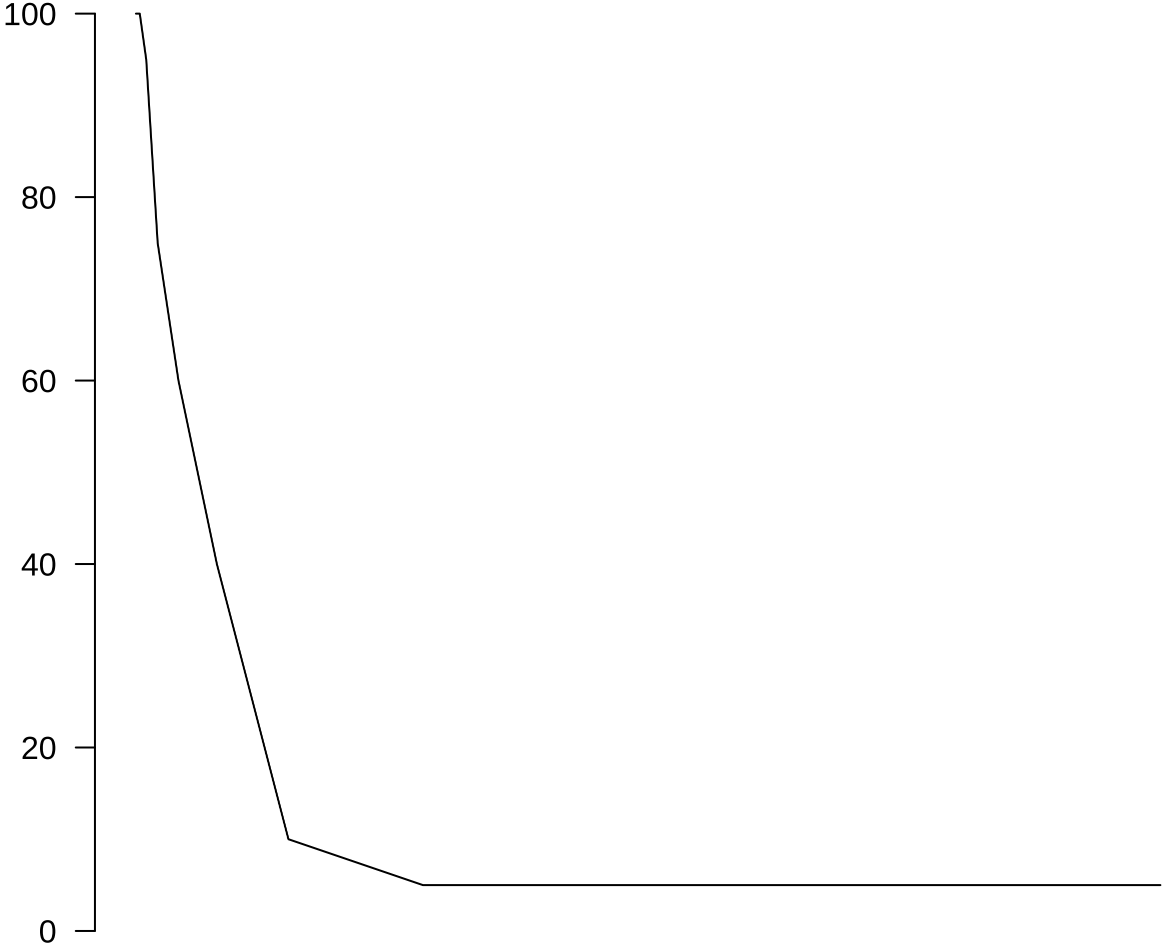

participant was in isolated BF condition
classified as exponential

139

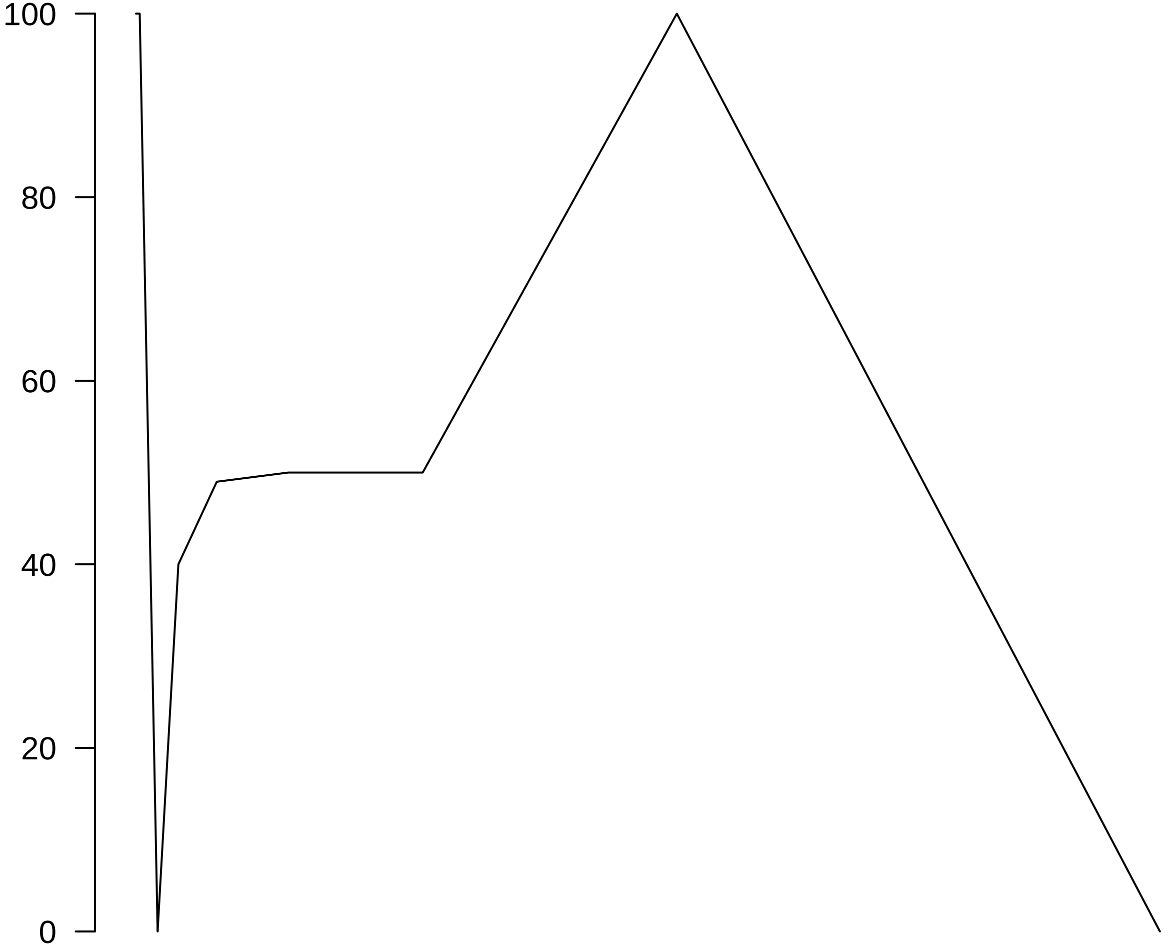

participant was in isolated BF condition
classified as rest

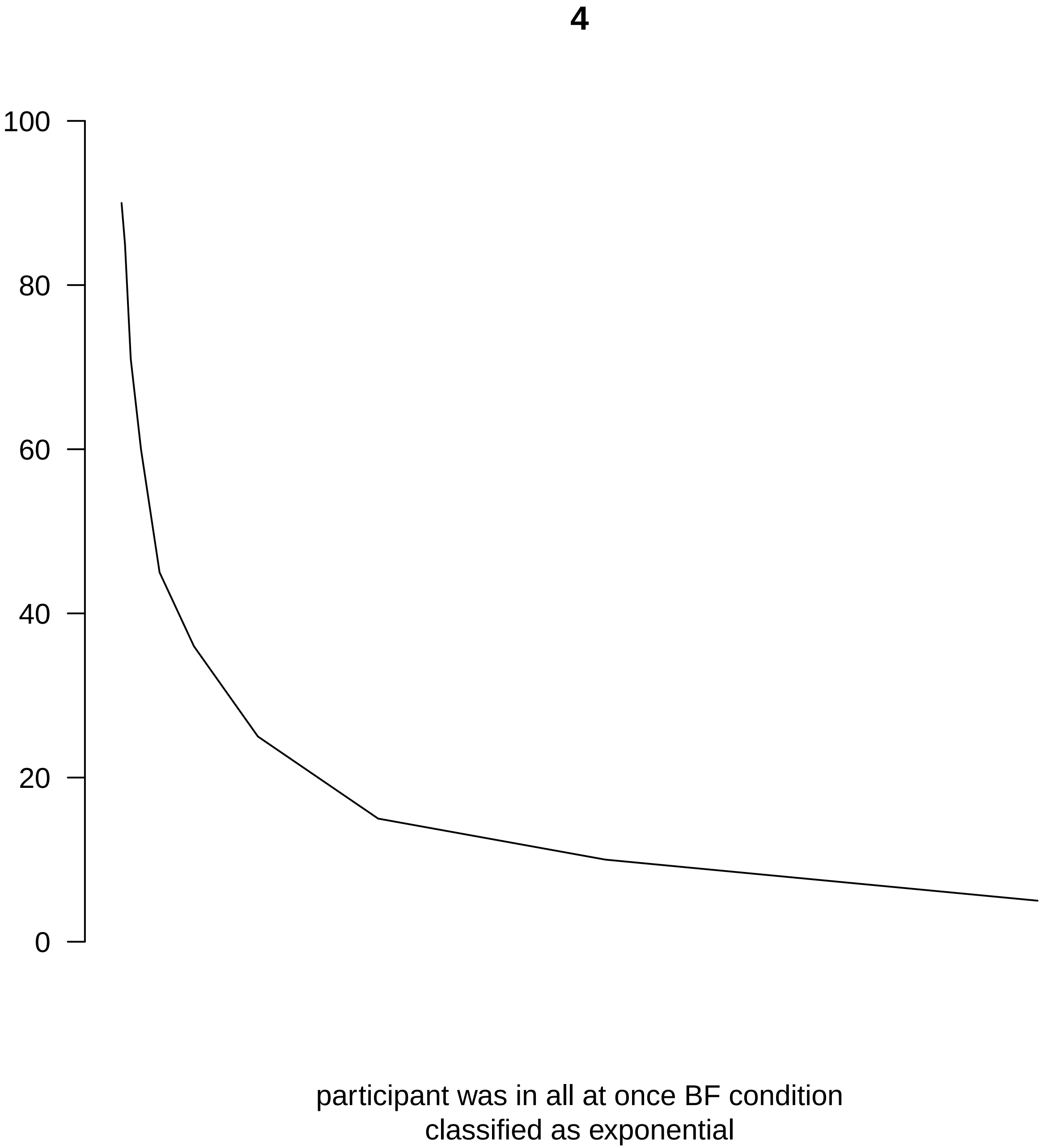
4
100
80
60
40
20
0
participant was in all at once BF condition
classified as exponential

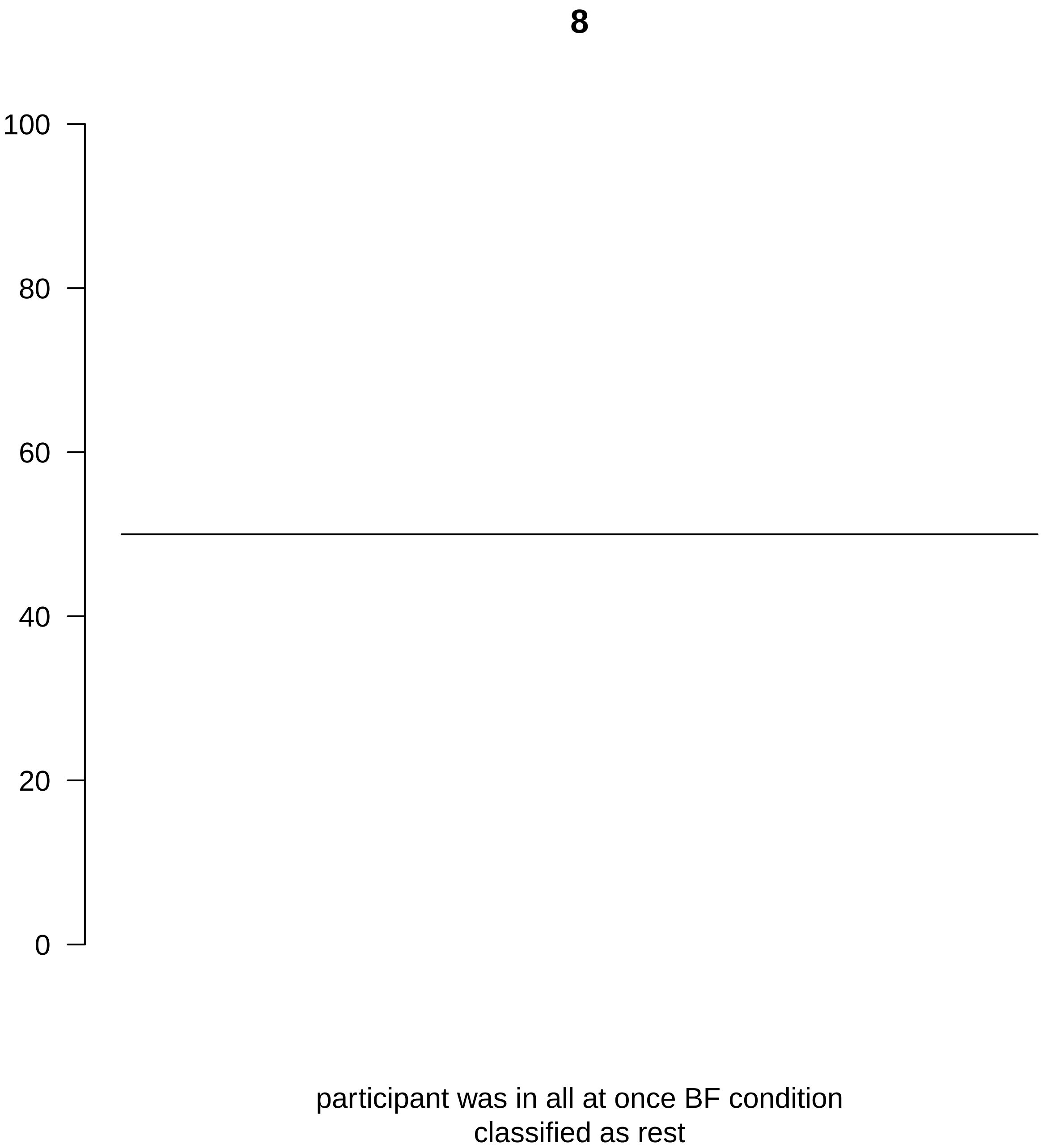
8
100
80
60
40
20
0
participant was in all at once BF condition
classified as rest

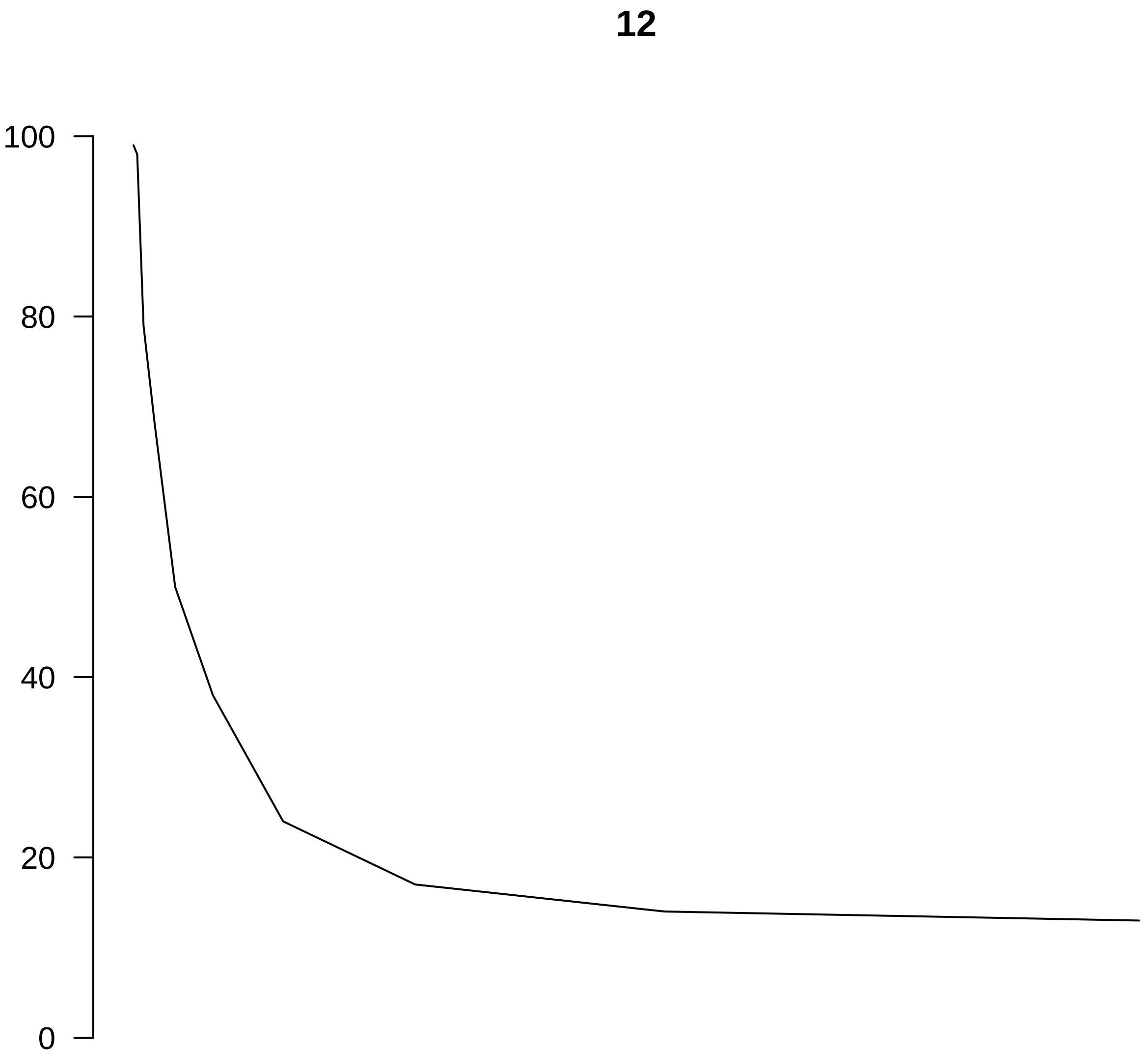

participant was in all at once BF condition
classified as exponential

15

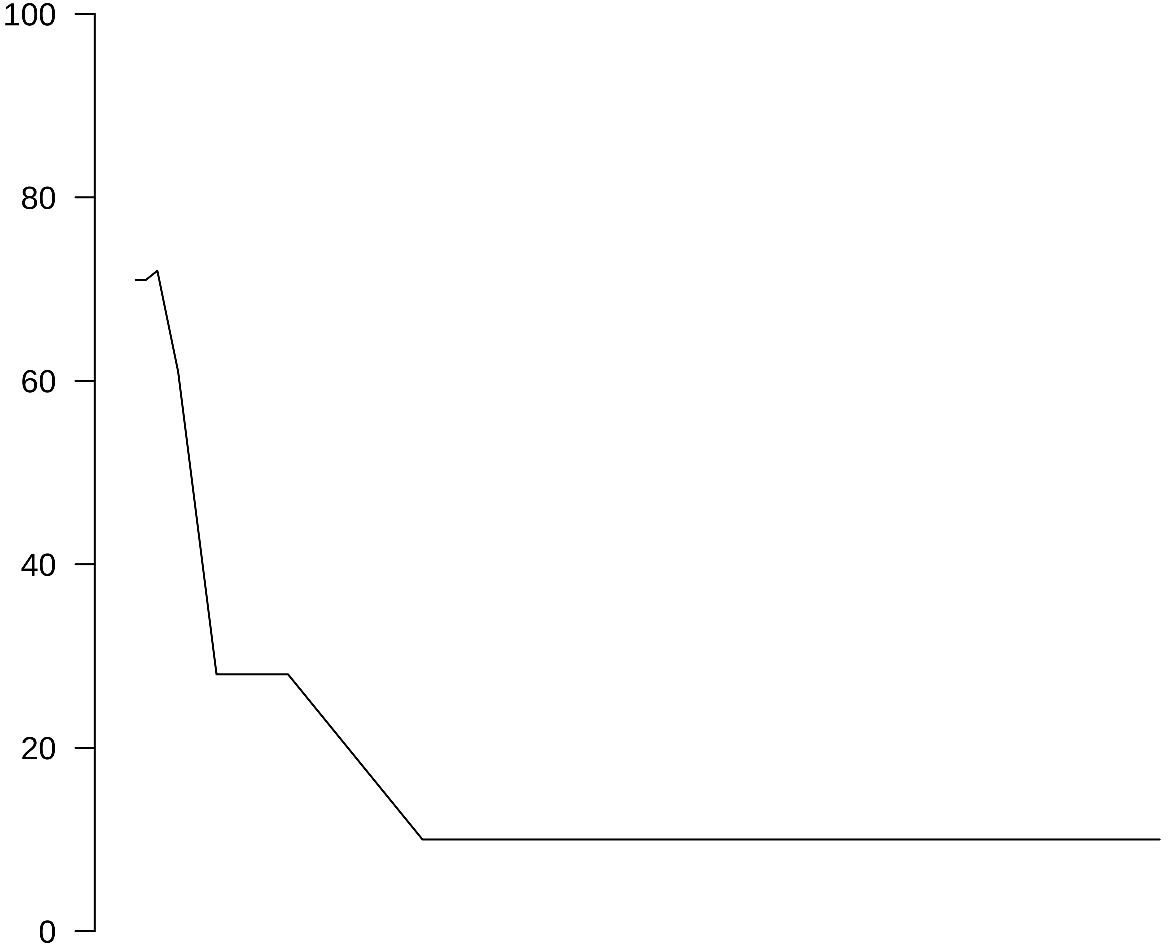

participant was in all at once BF condition
classified as exponential

22

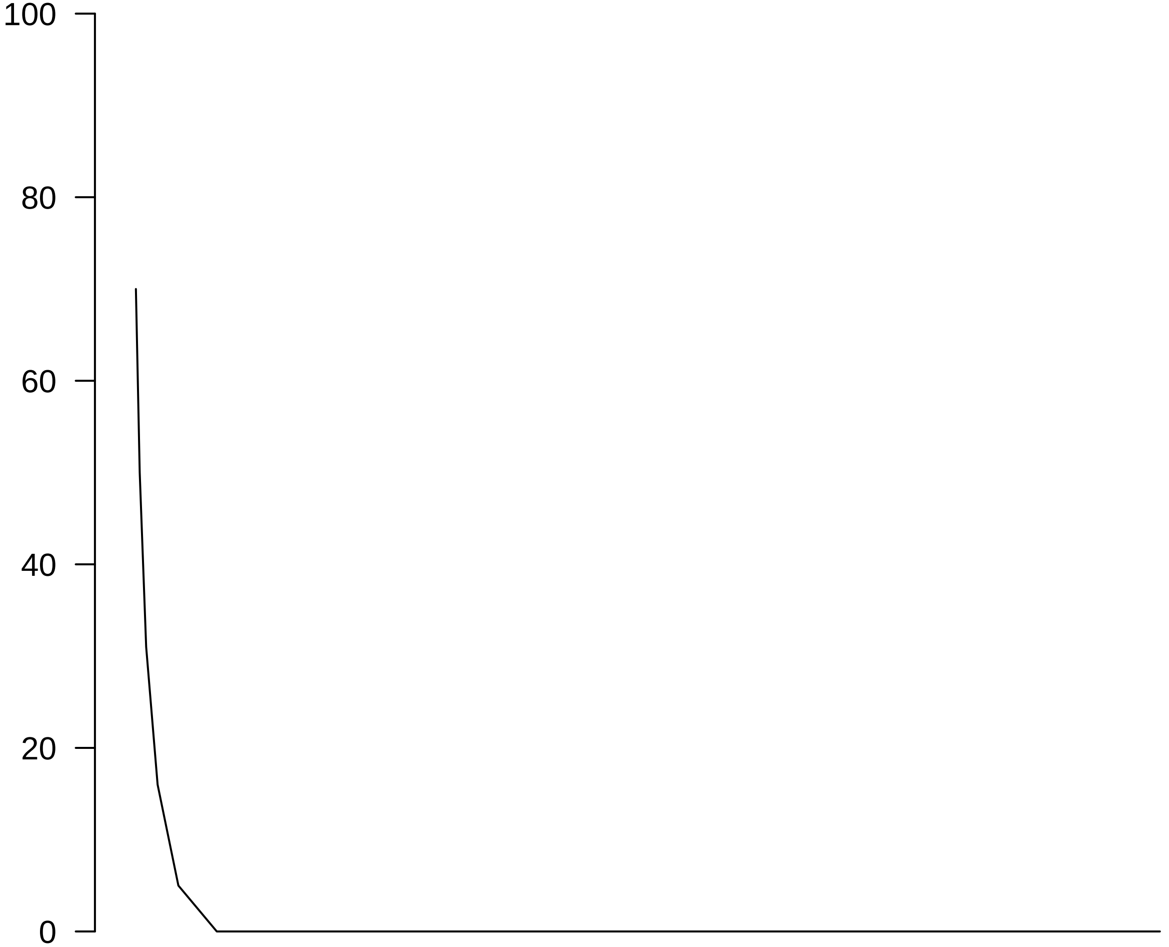

participant was in all at once BF condition
classified as exponential

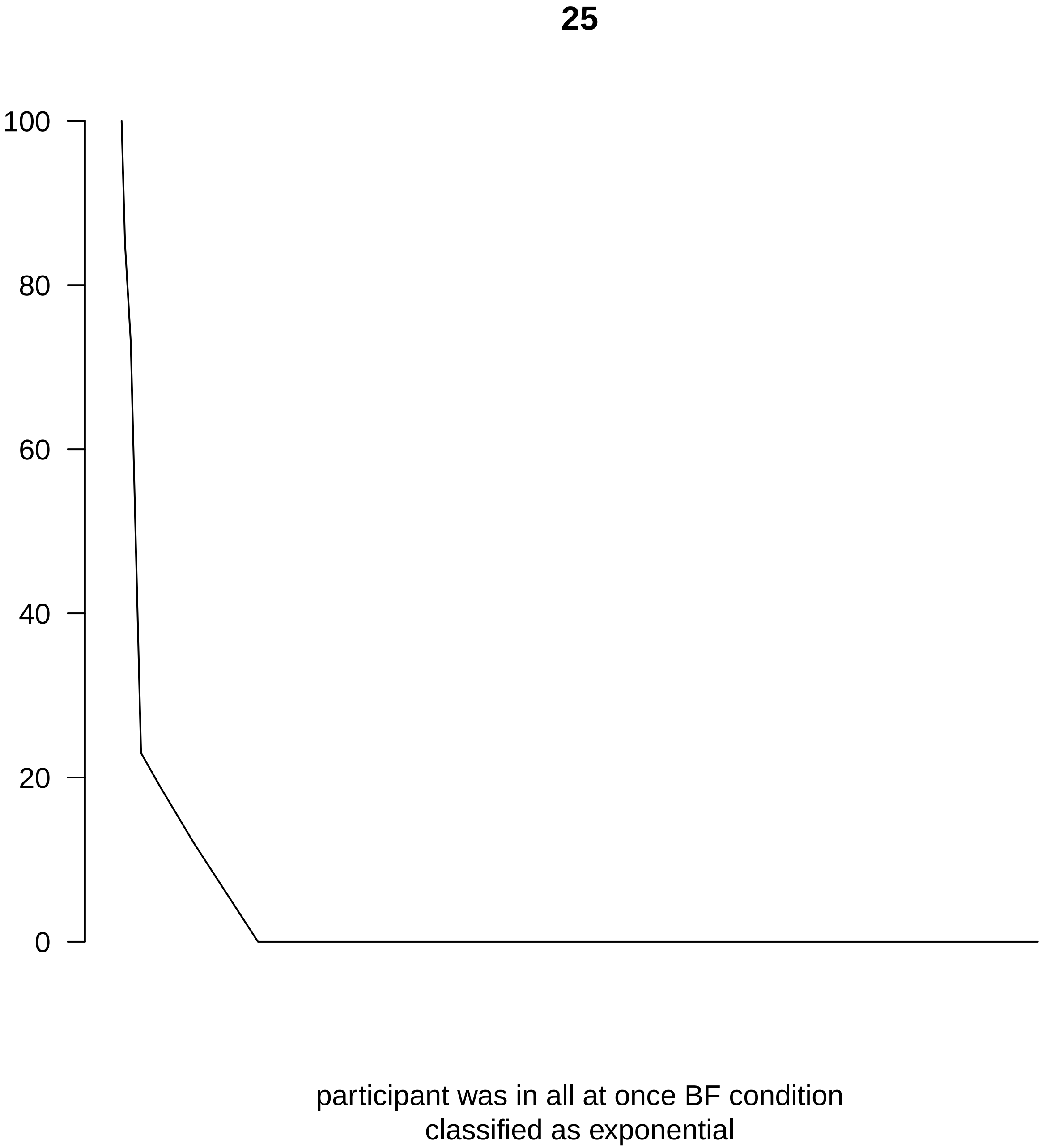
25
100
80
60
40
20
0
participant was in all at once BF condition
classified as exponential

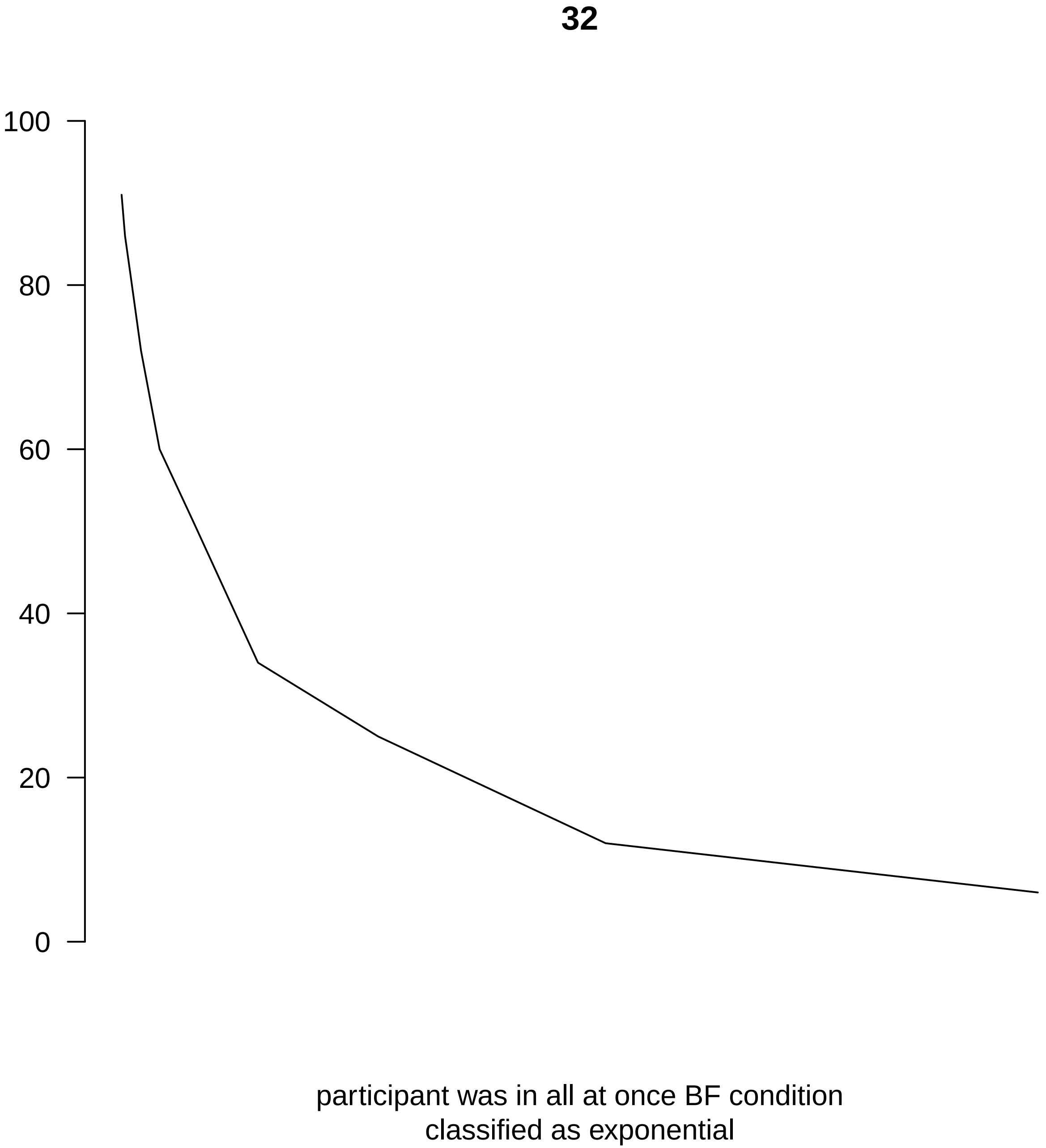
32
100
80
60
40
20
0
participant was in all at once BF condition
classified as exponential

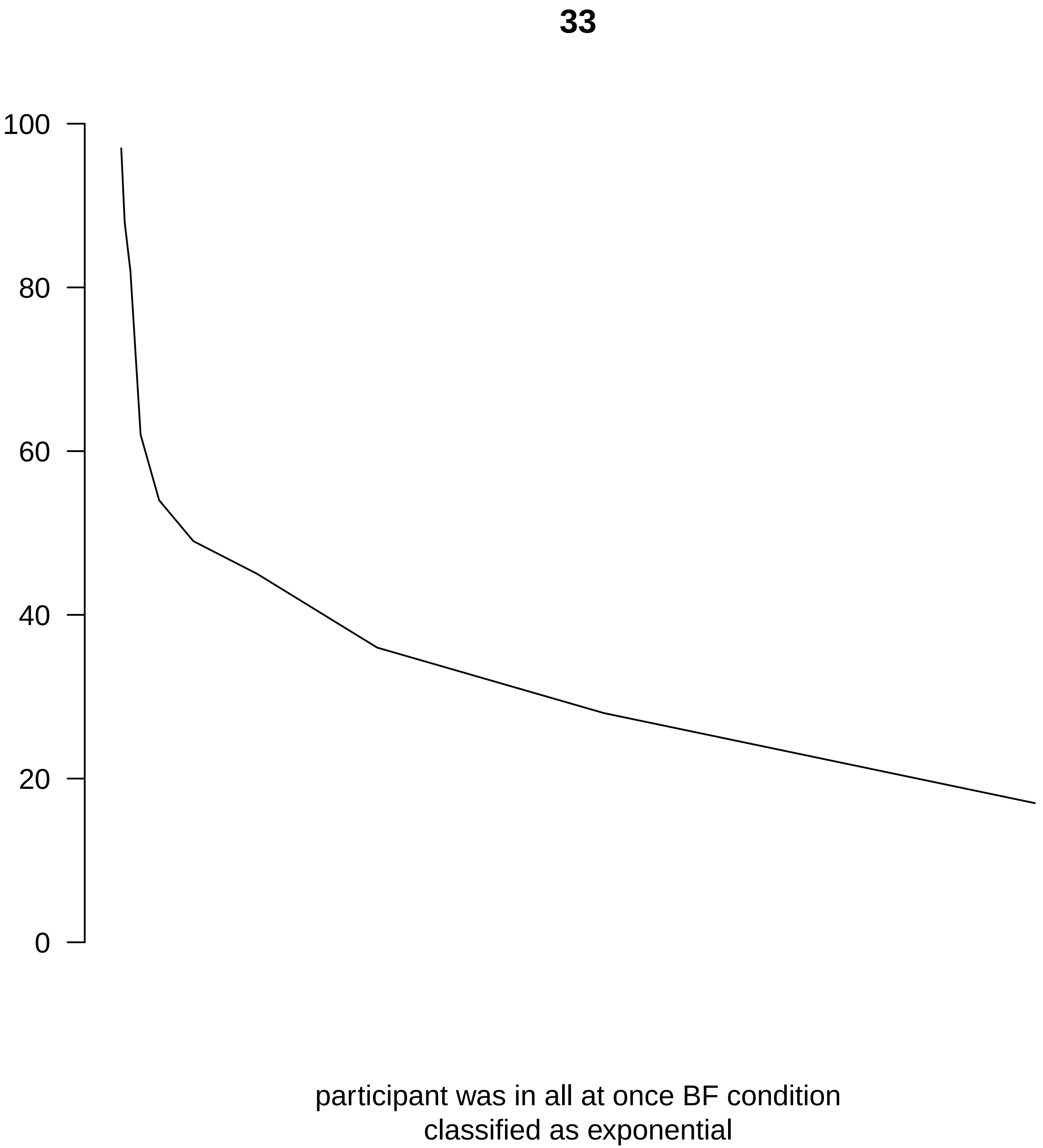
33
100
80
60
40
20
0
participant was in all at once BF condition
classified as exponential

38

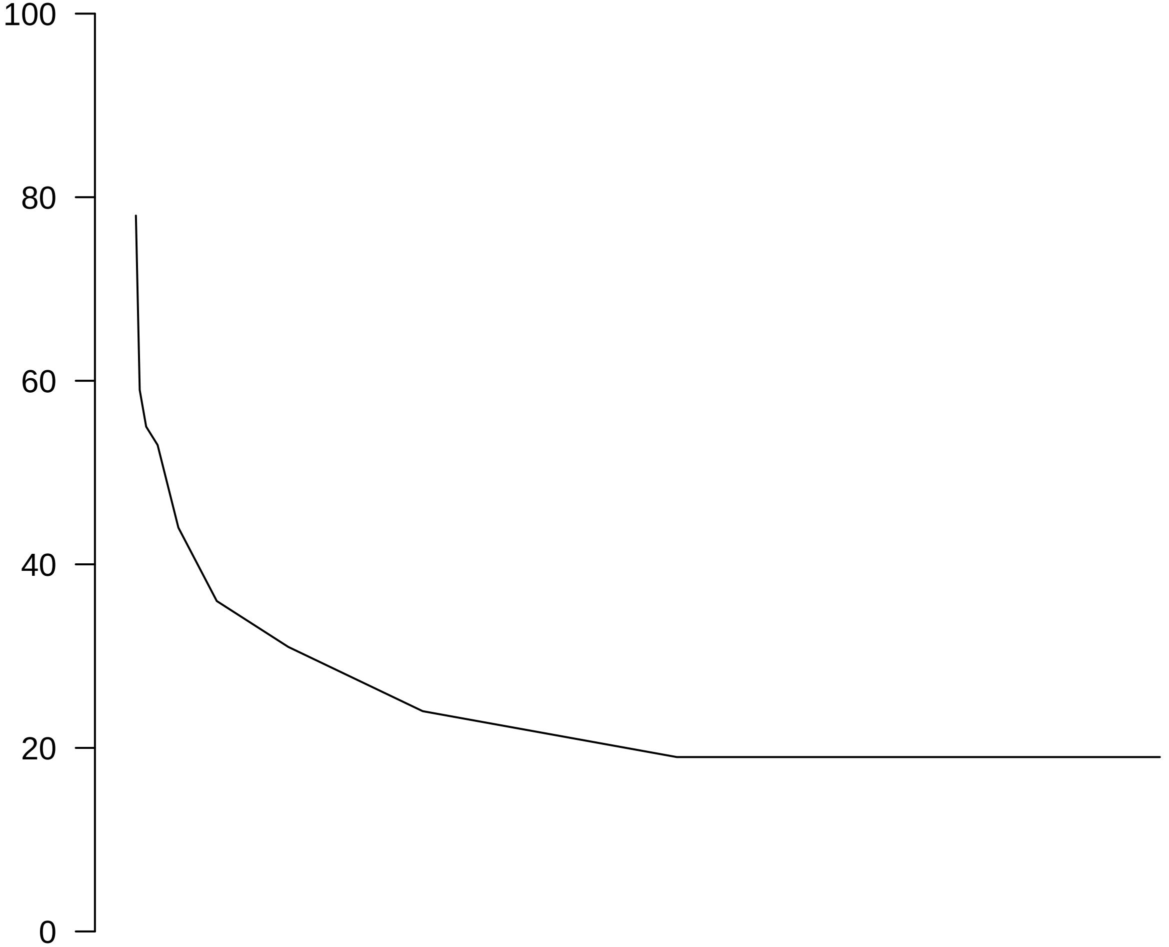

participant was in all at once BF condition
classified as exponential

43

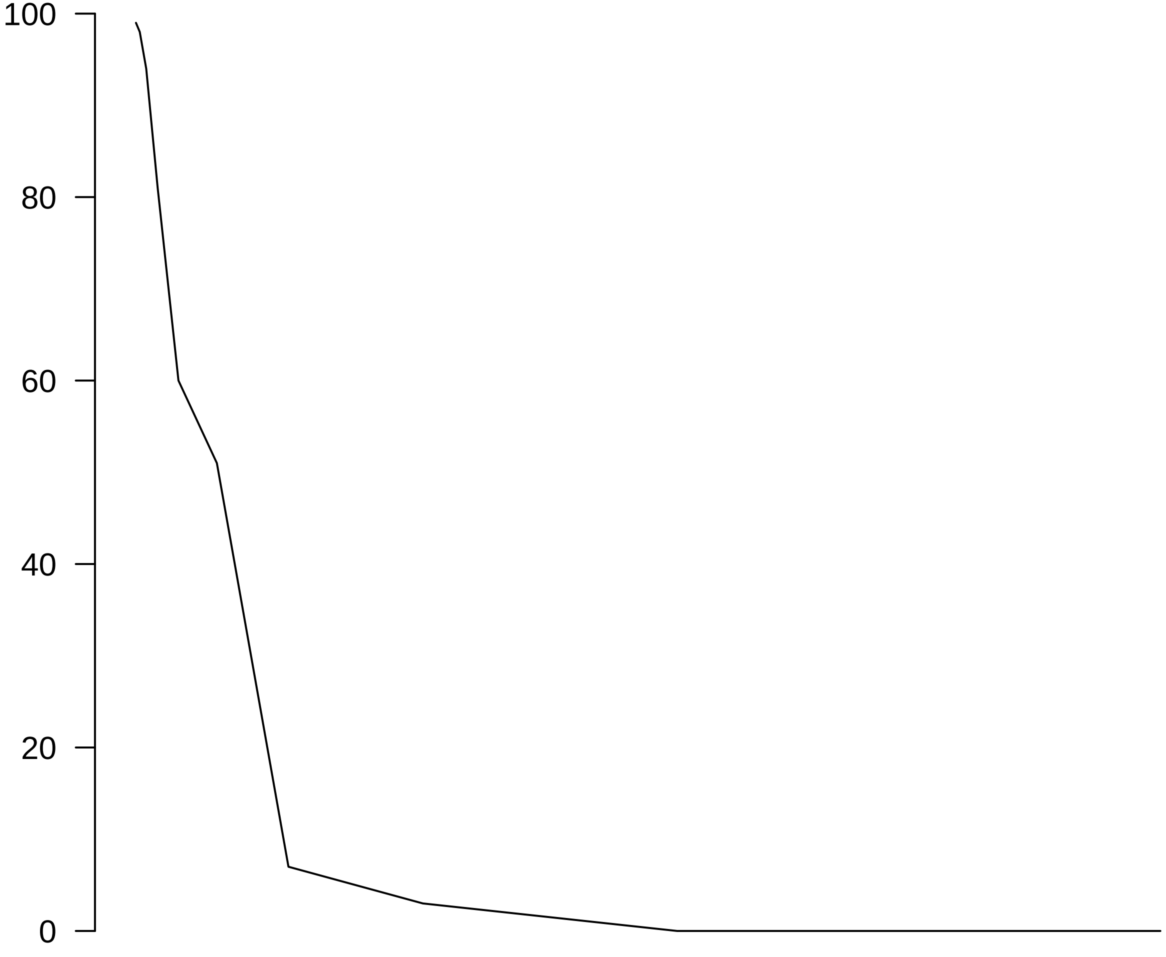

participant was in all at once BF condition
classified as exponential

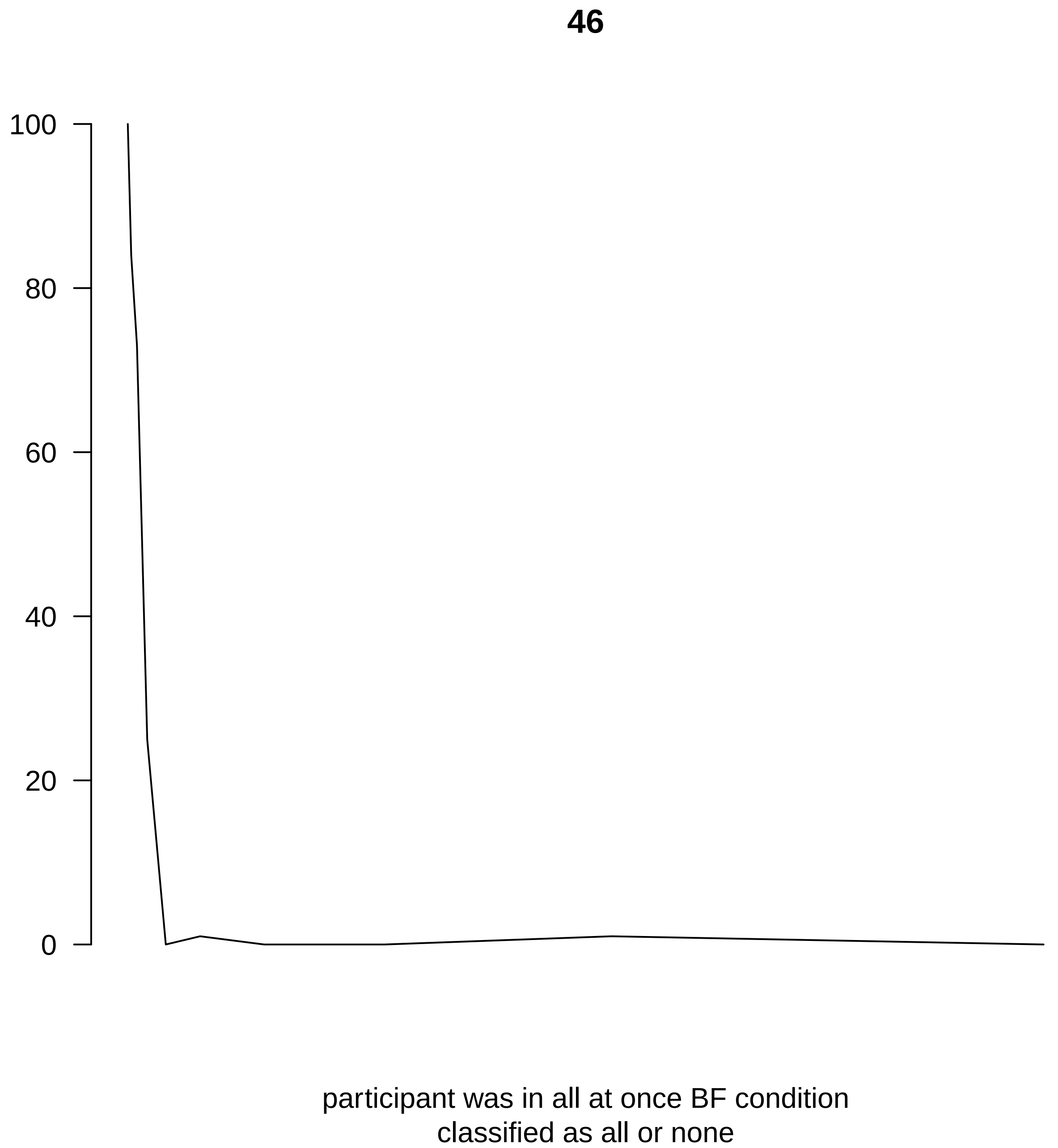
46
100
80
60
40
20
0
participant was in all at once BF condition
classified as all or none

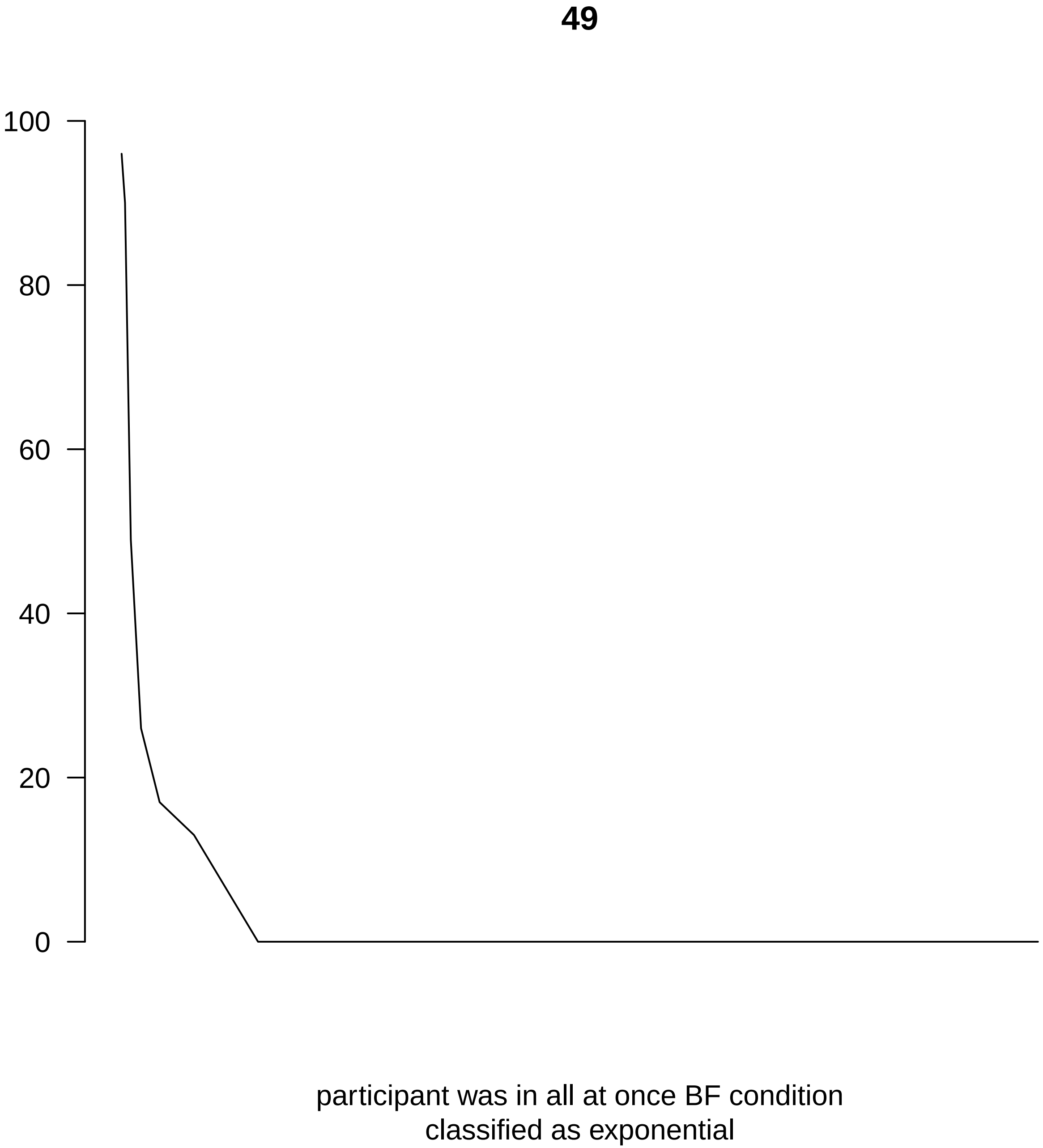
49
100
80
60
40
20
0
participant was in all at once BF condition
classified as exponential

54

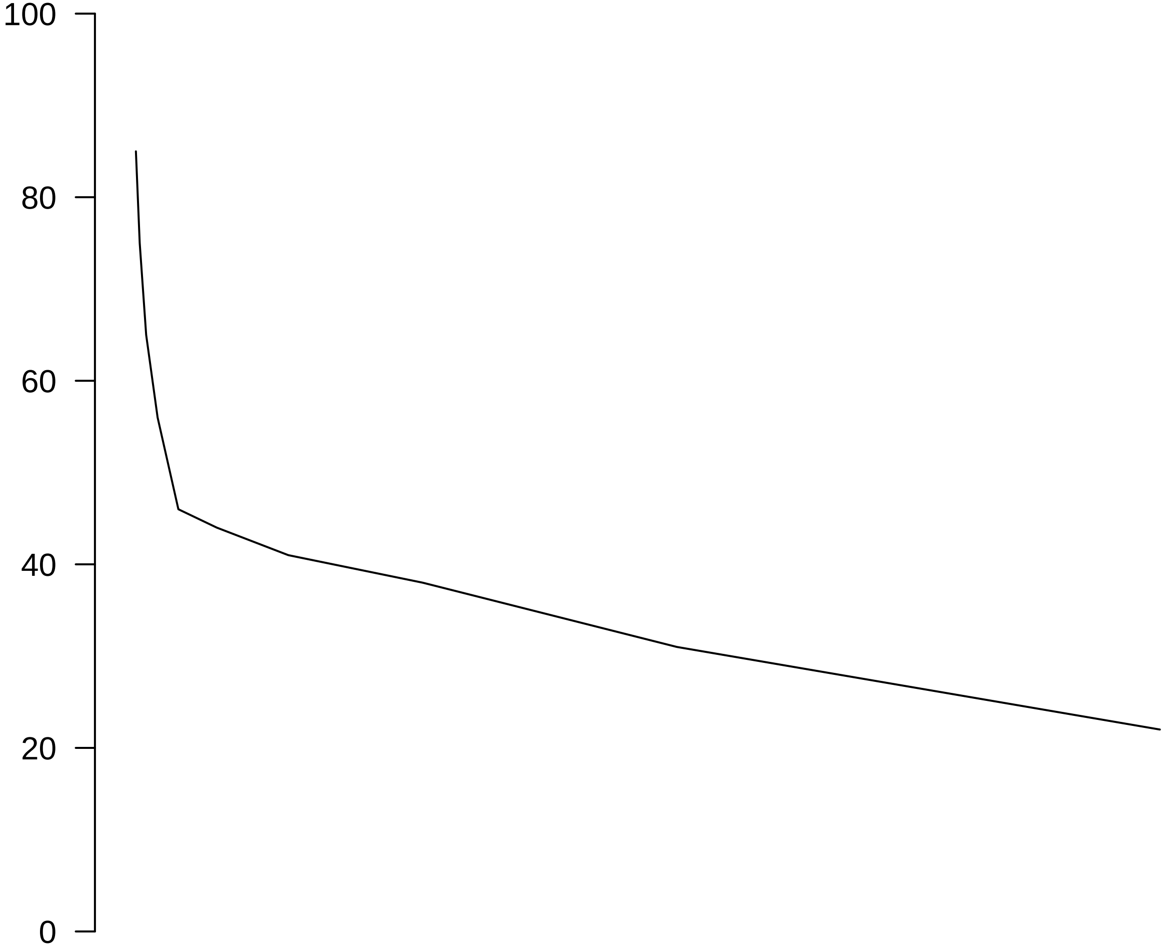

participant was in all at once BF condition
classified as exponential

57

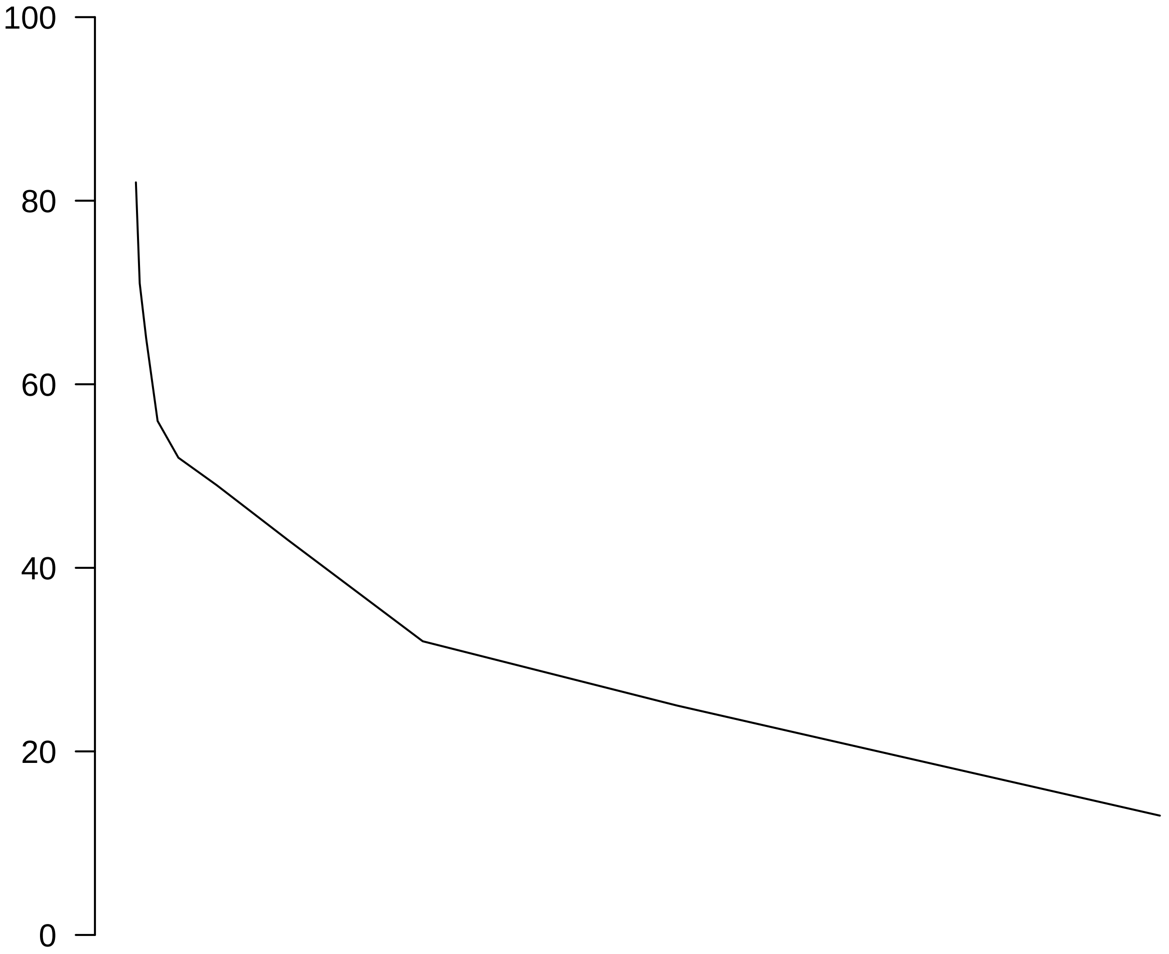

participant was in all at once BF condition
classified as exponential

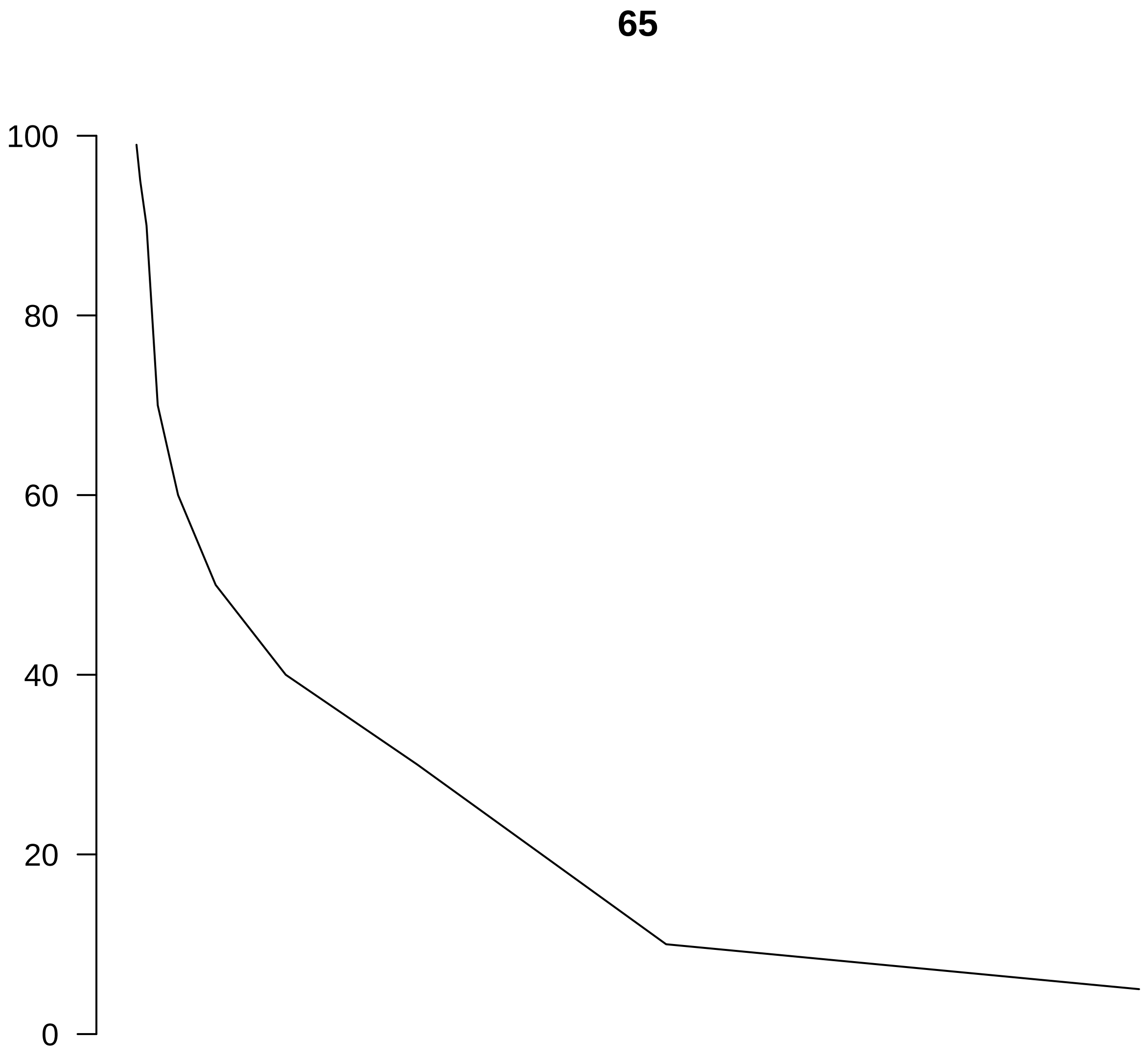

participant was in all at once BF condition
classified as exponential

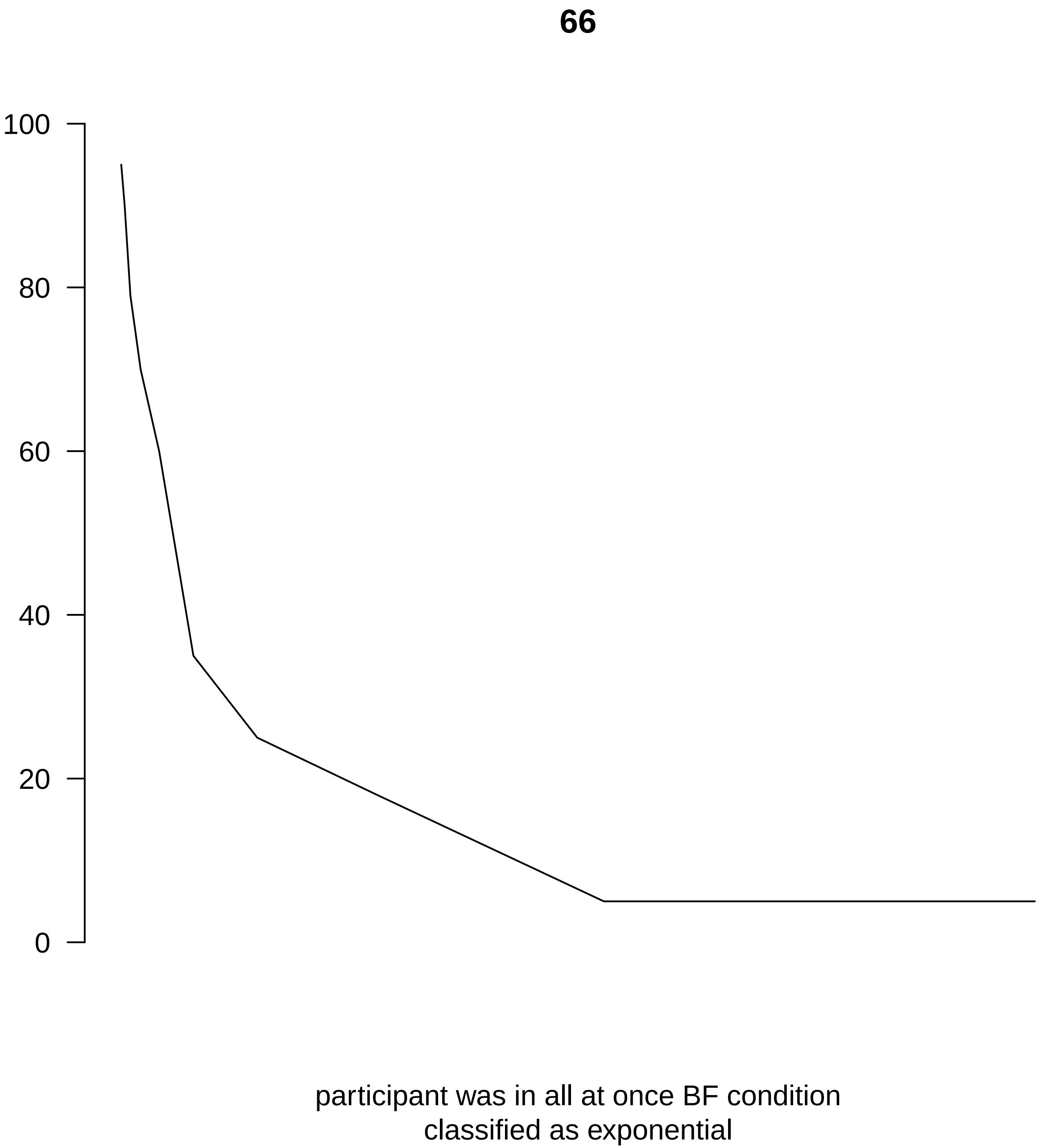
66
100
80
60
40
20
0
participant was in all at once BF condition
classified as exponential

72

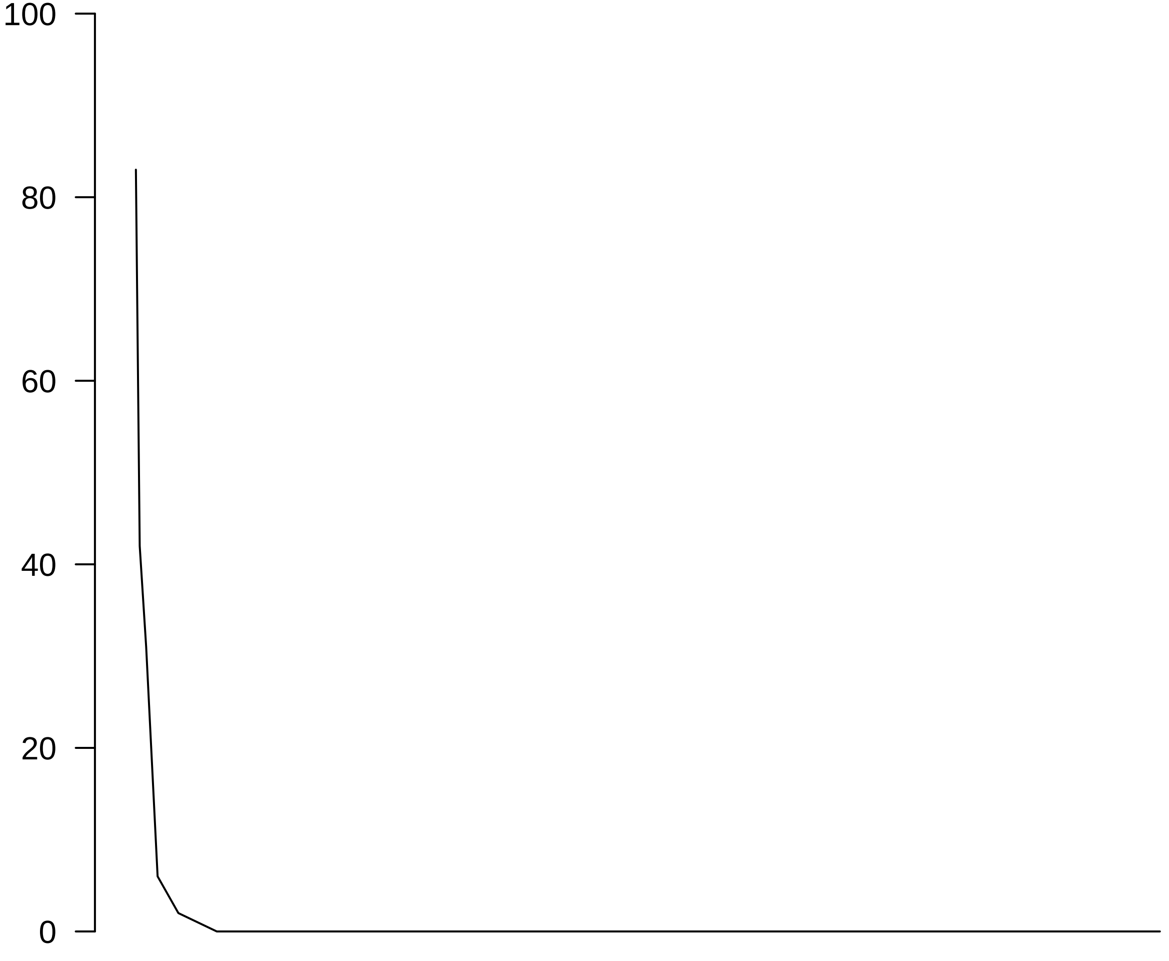

participant was in all at once BF condition
classified as exponential

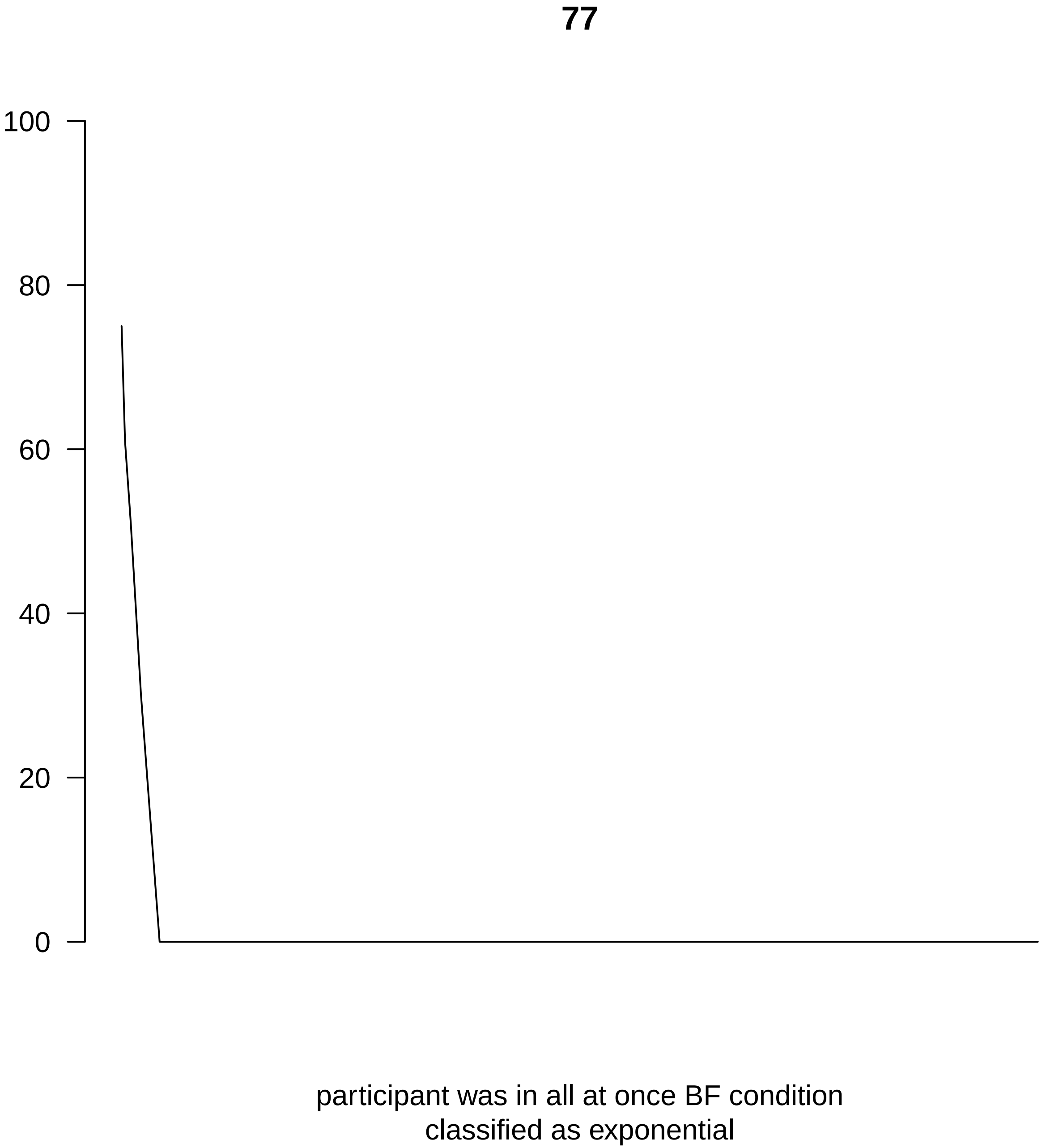
77
100
80
60
40
20
0
participant was in all at once BF condition
classified as exponential

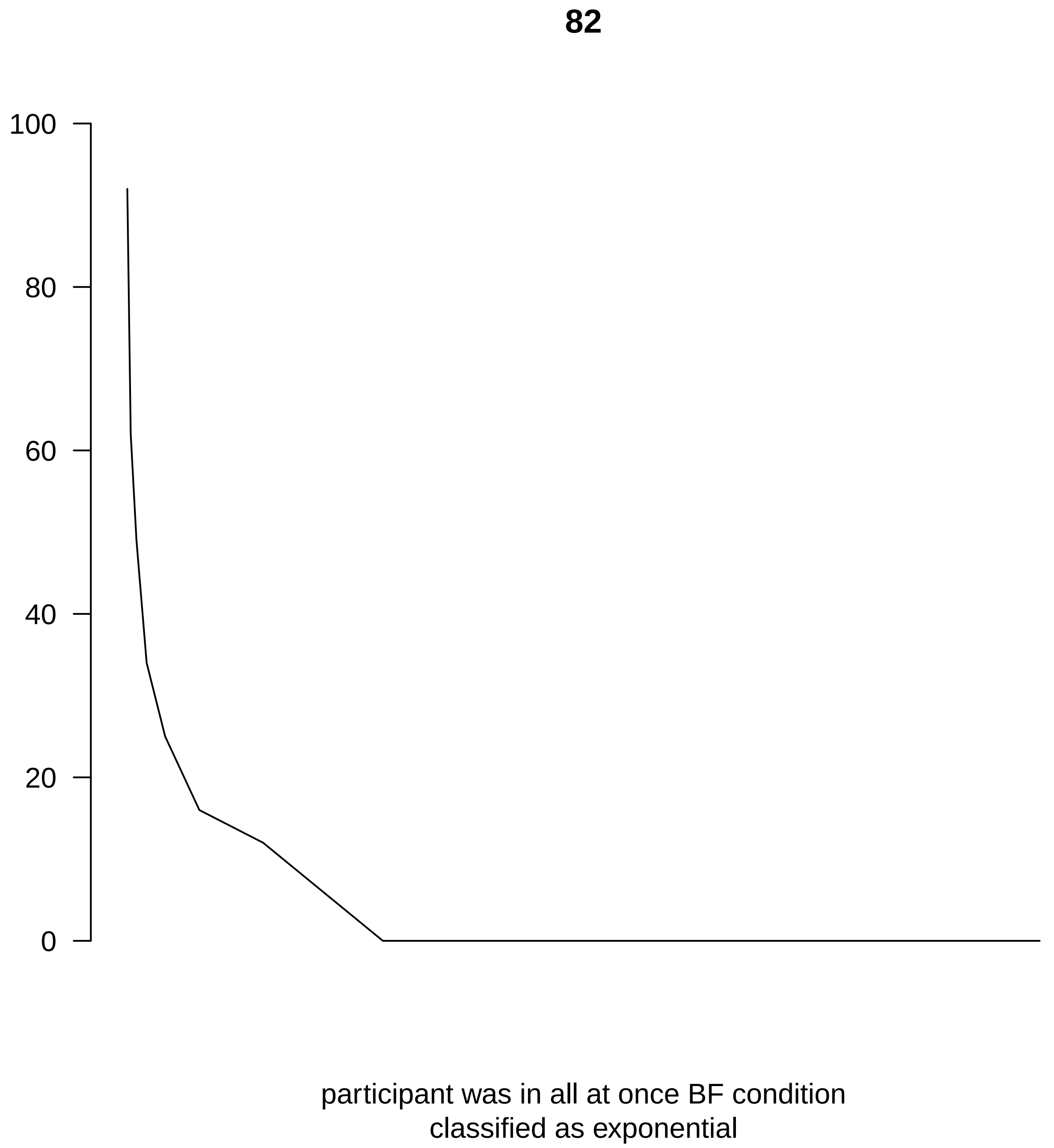
82
100
80
60
40
20
0
participant was in all at once BF condition
classified as exponential

84

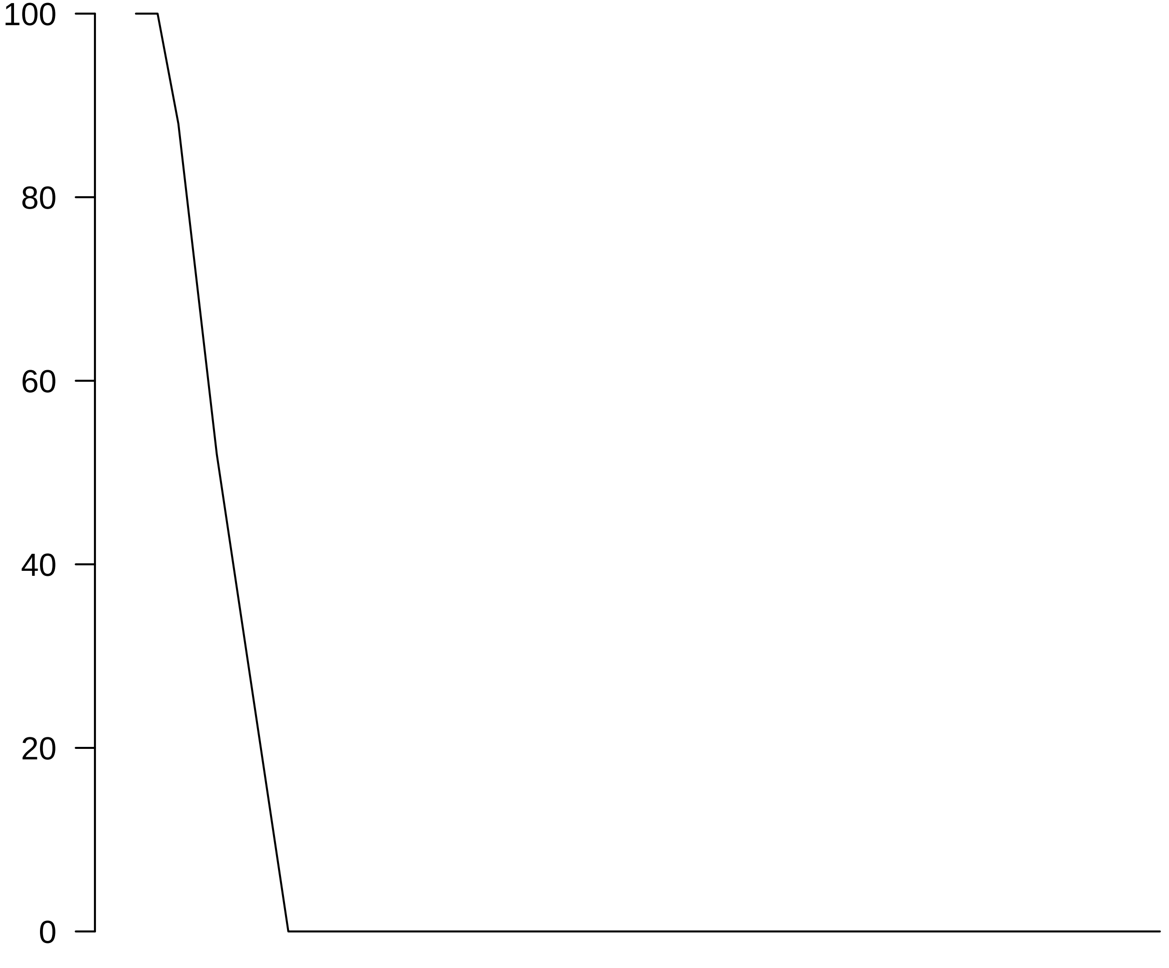

participant was in all at once BF condition
classified as all or none

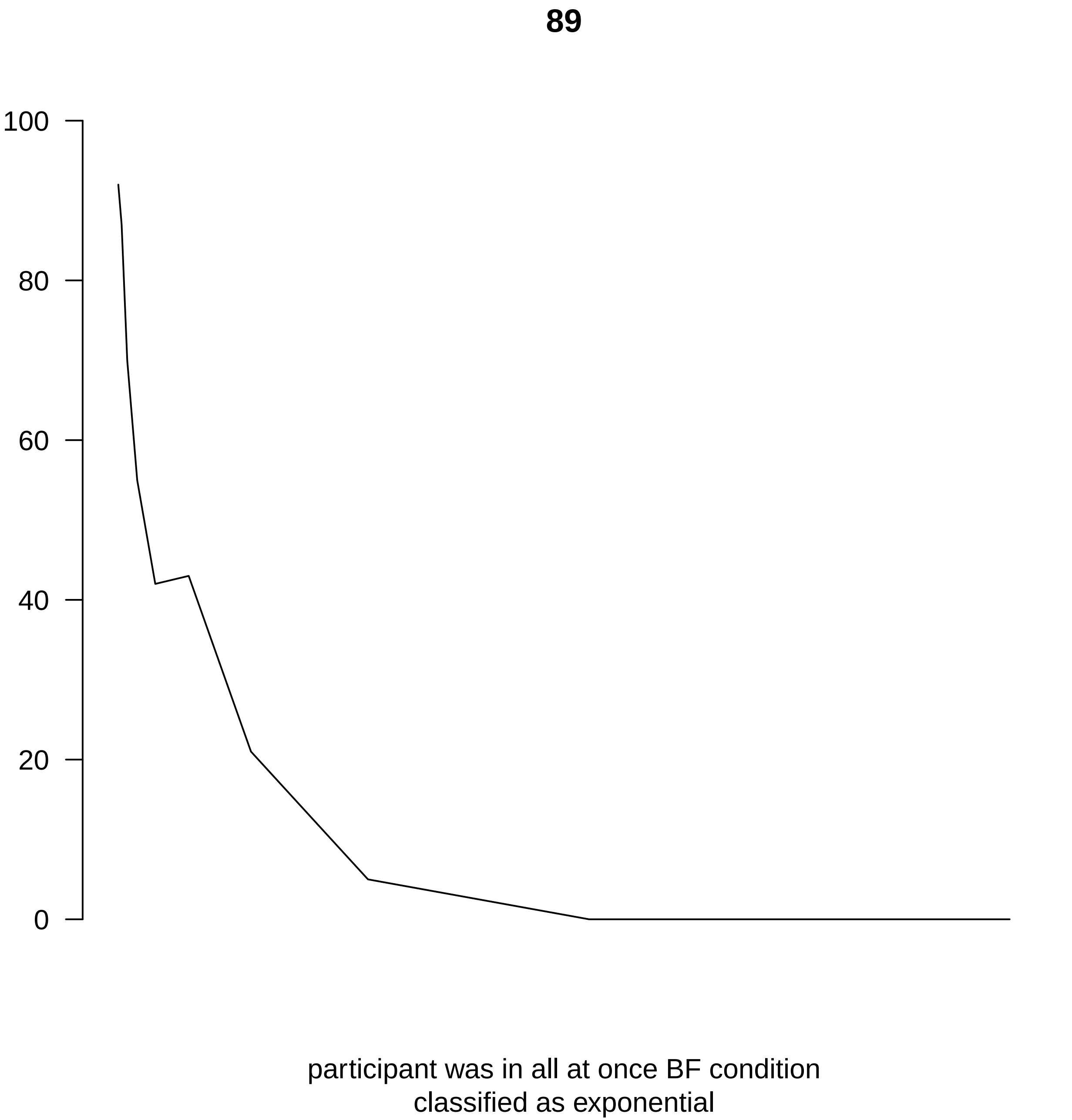

89
100
80
60
40
20
0
participant was in all at once BF condition
classified as exponential

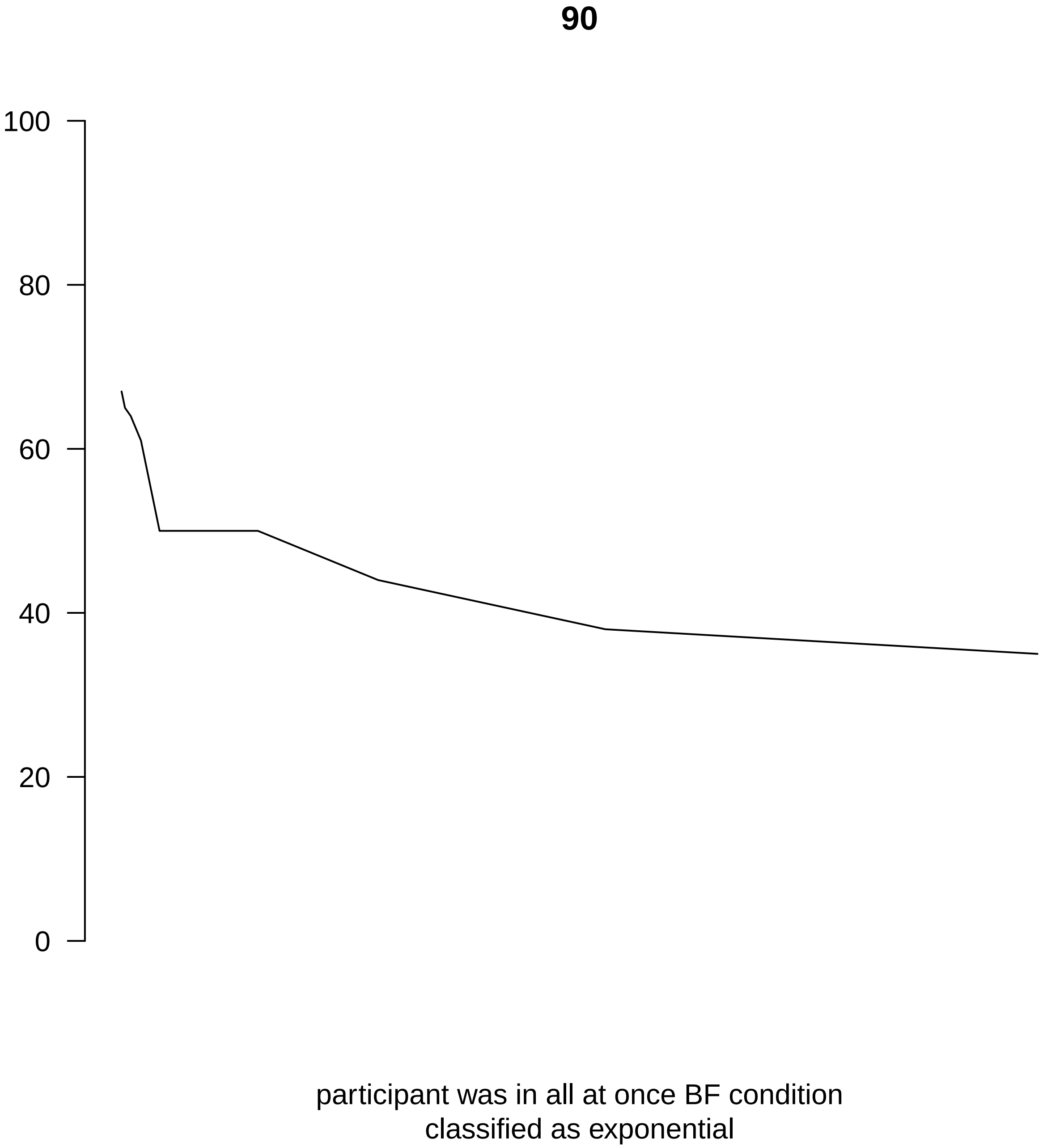
90
100
80
60
40
20
0
participant was in all at once BF condition
classified as exponential

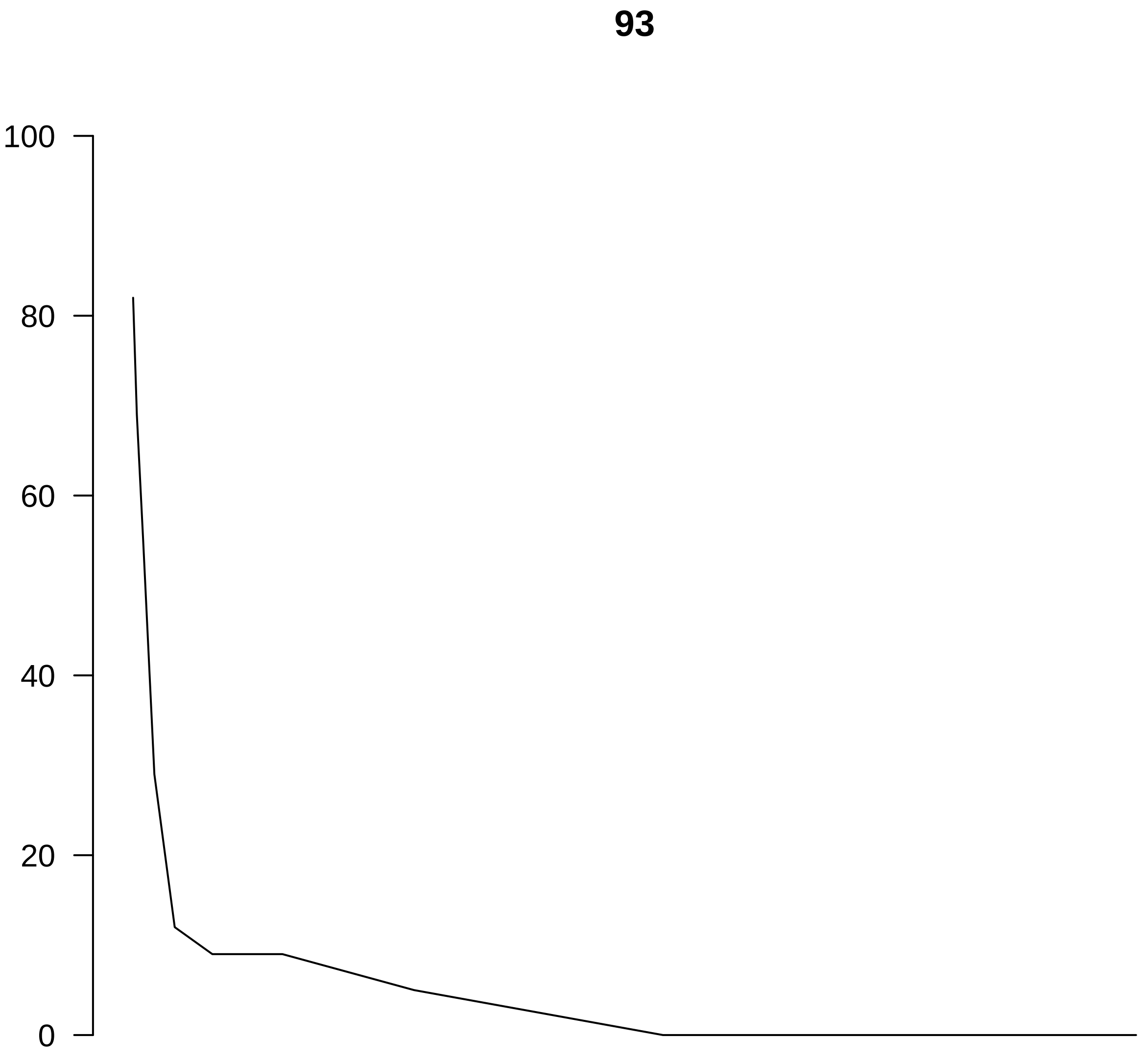

participant was in all at once BF condition
classified as exponential

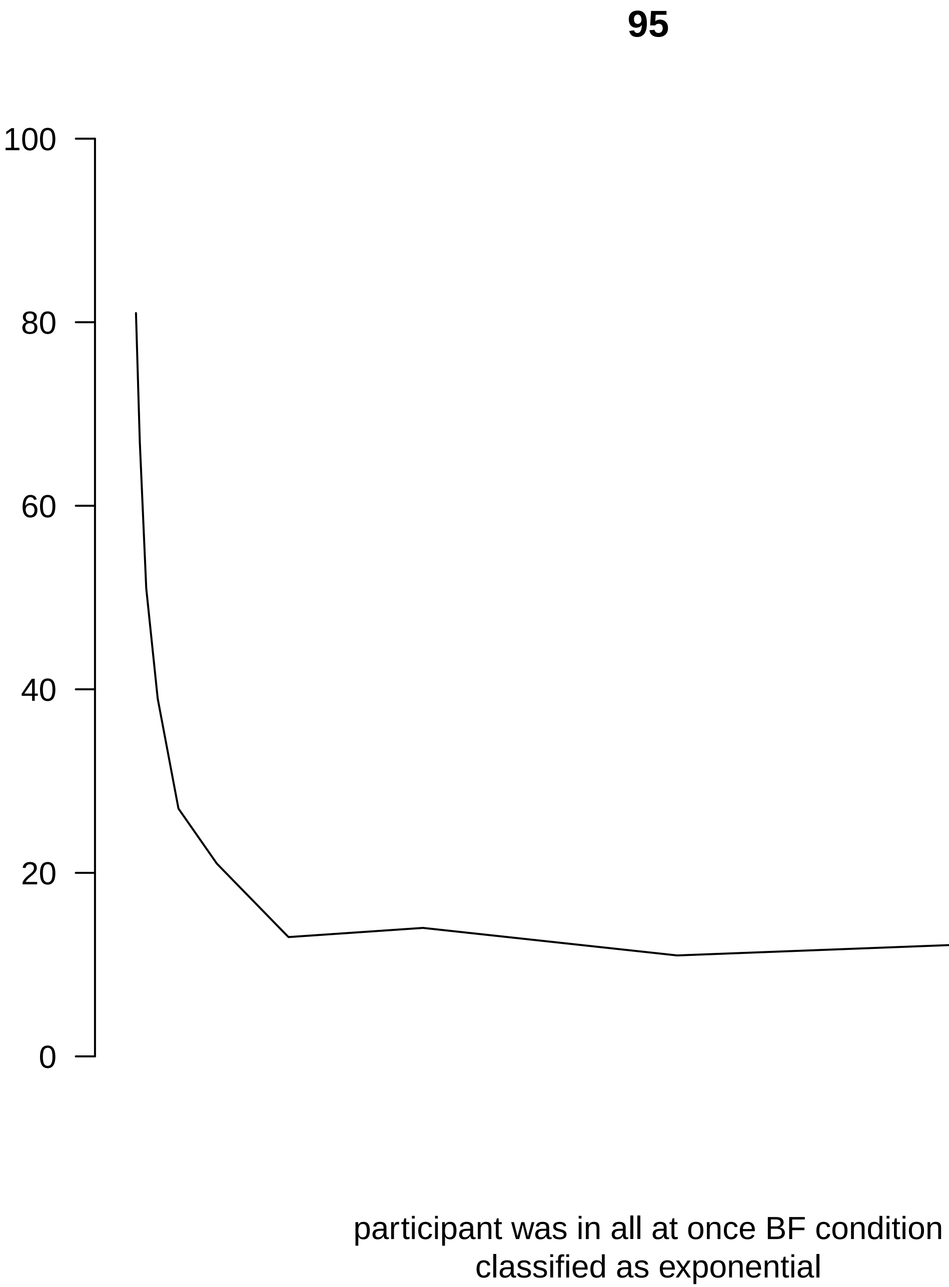
95
100
80
60
40
20
0
participant was in all at once BF condition
classified as exponential

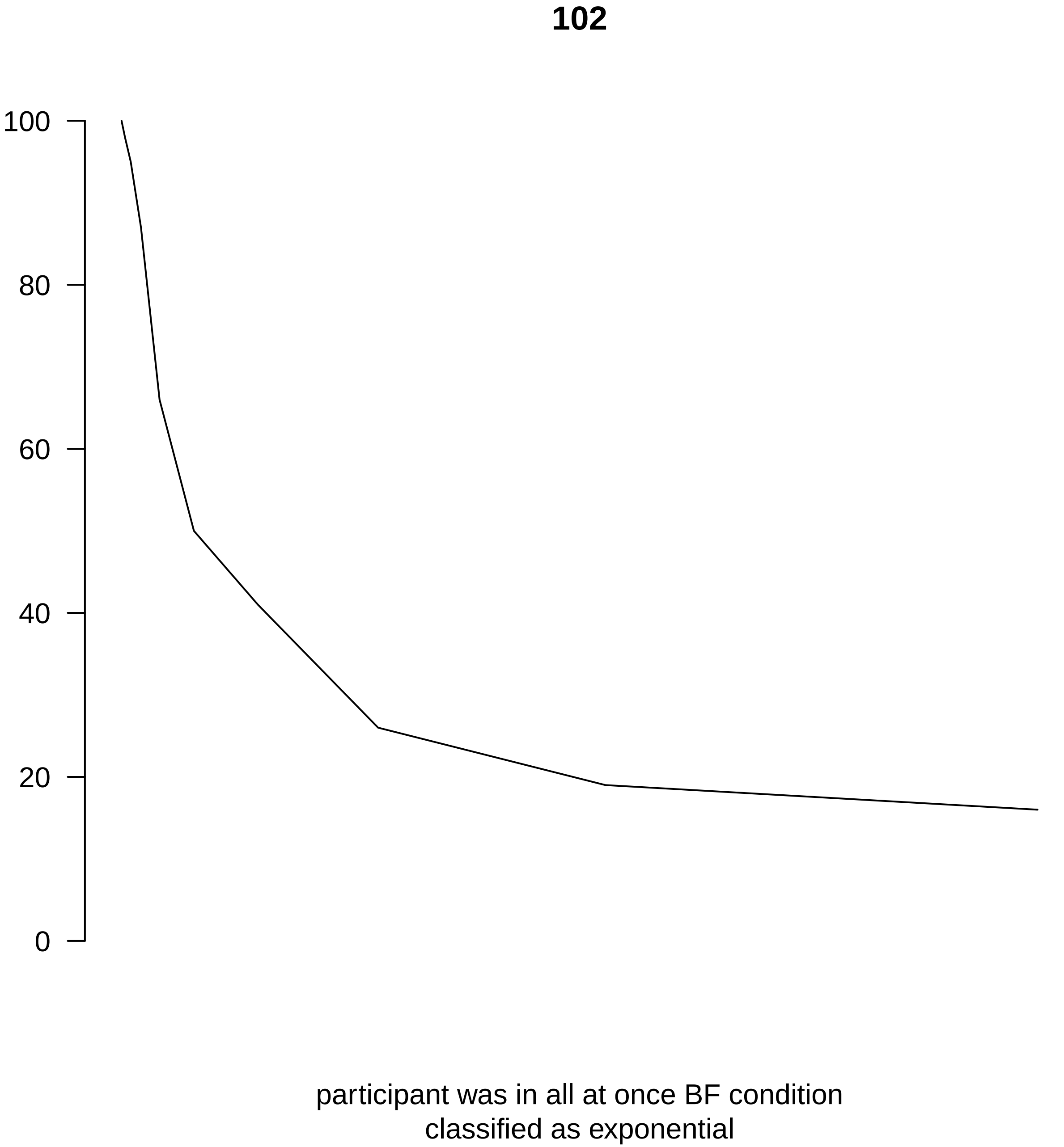
102
100
80
60
40
20
0
participant was in all at once BF condition
classified as exponential

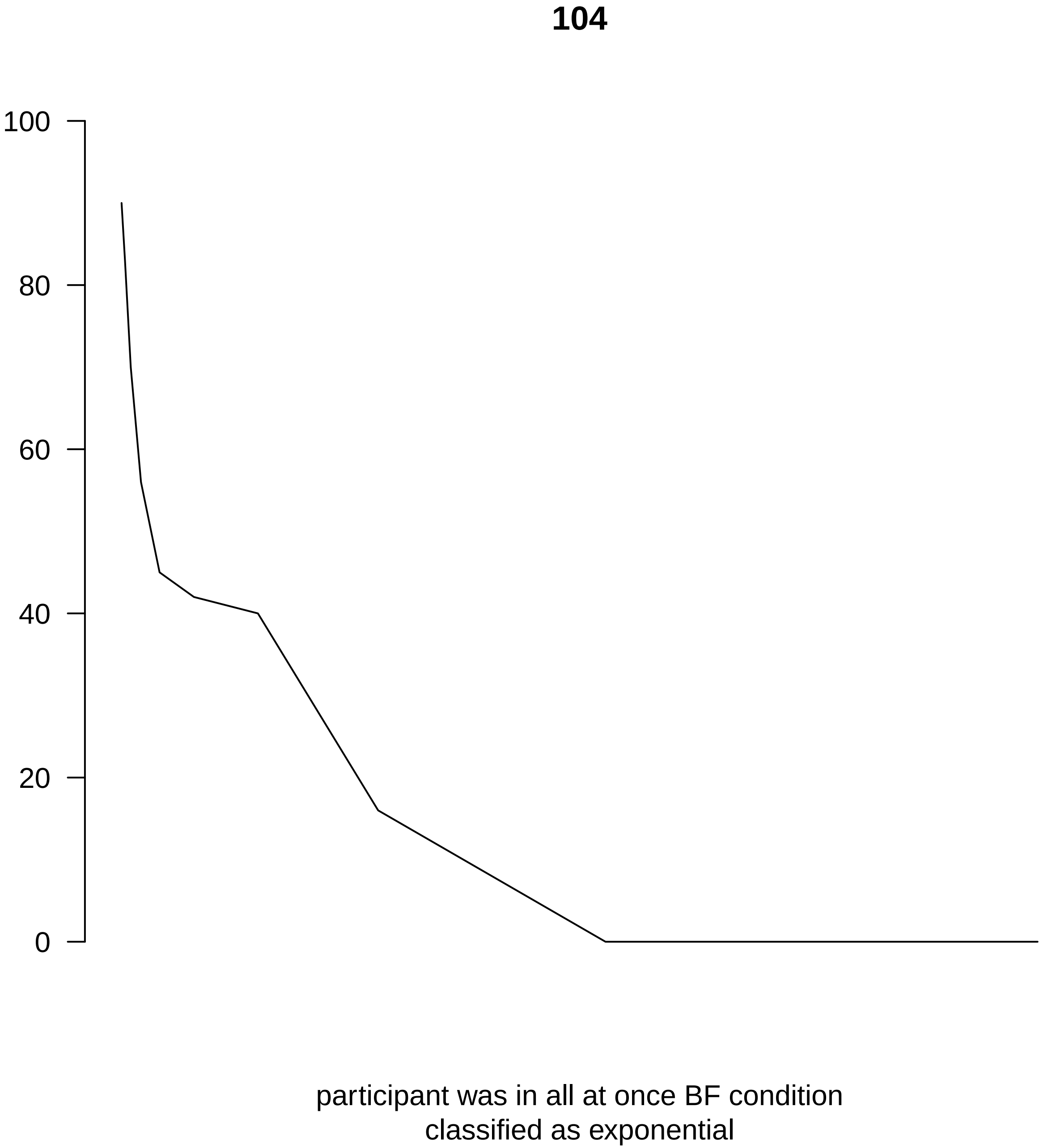
104
100
80
60
40
20
0
participant was in all at once BF condition
classified as exponential

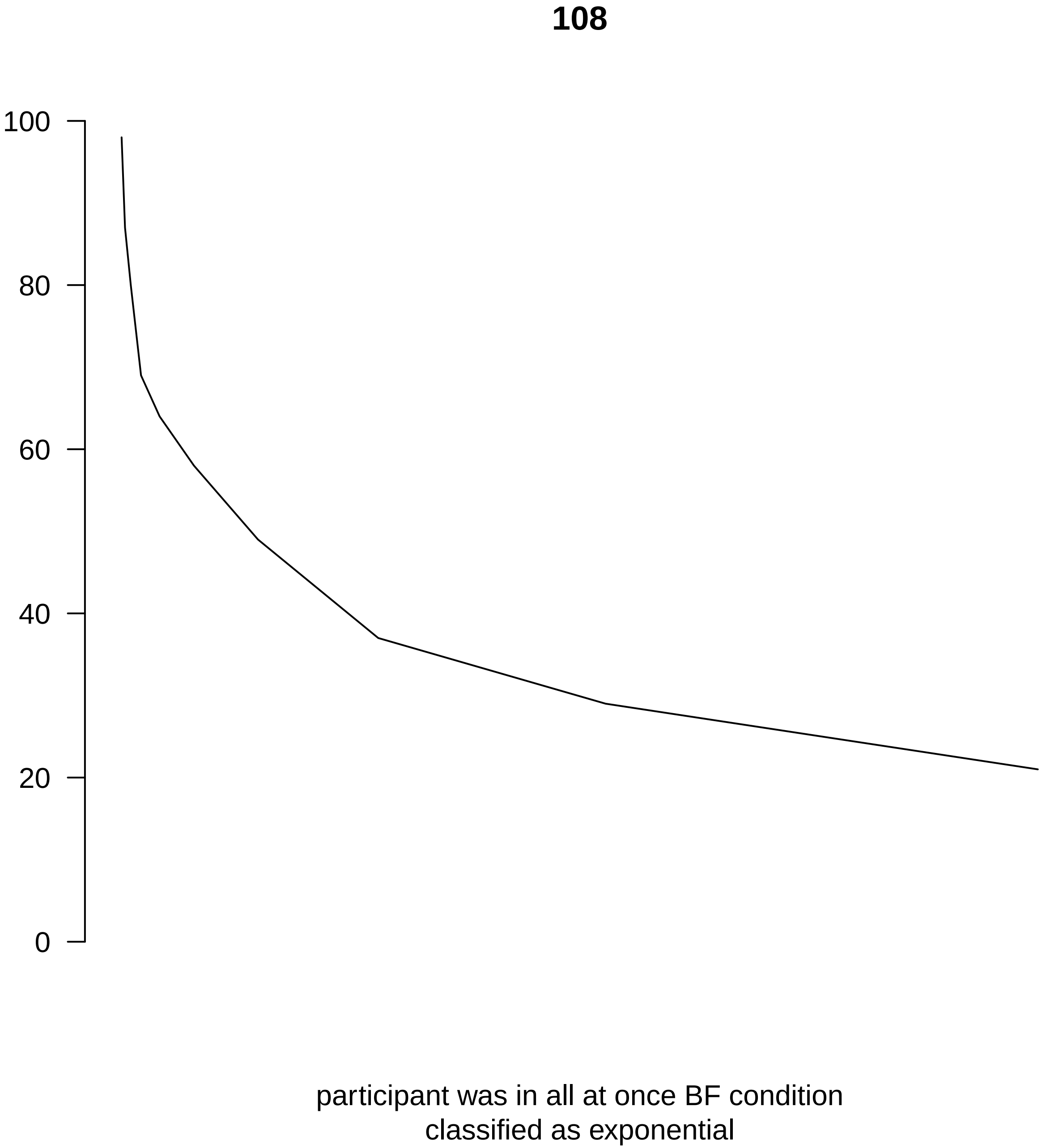
108
100
80
60
40
20
0
participant was in all at once BF condition
classified as exponential

112

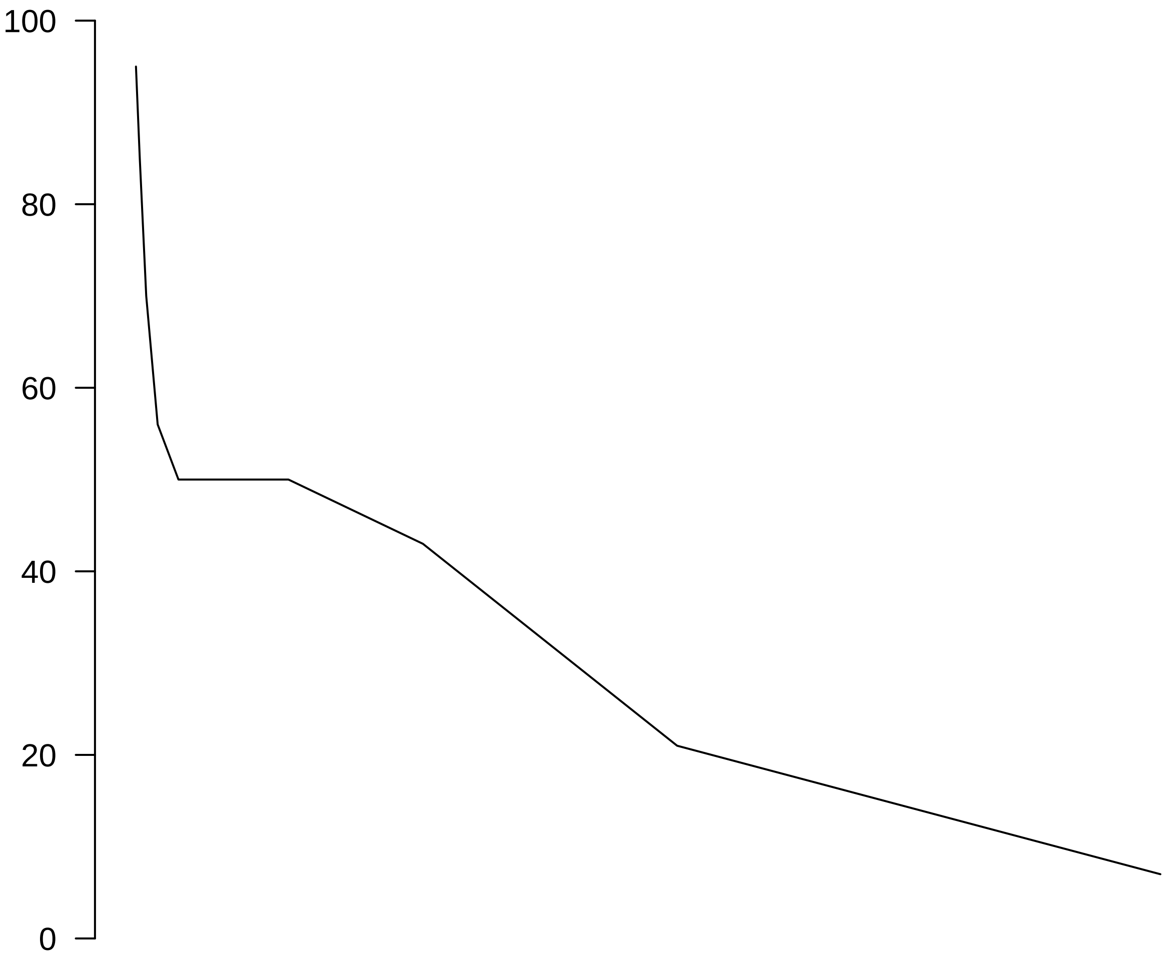

participant was in all at once BF condition
classified as exponential

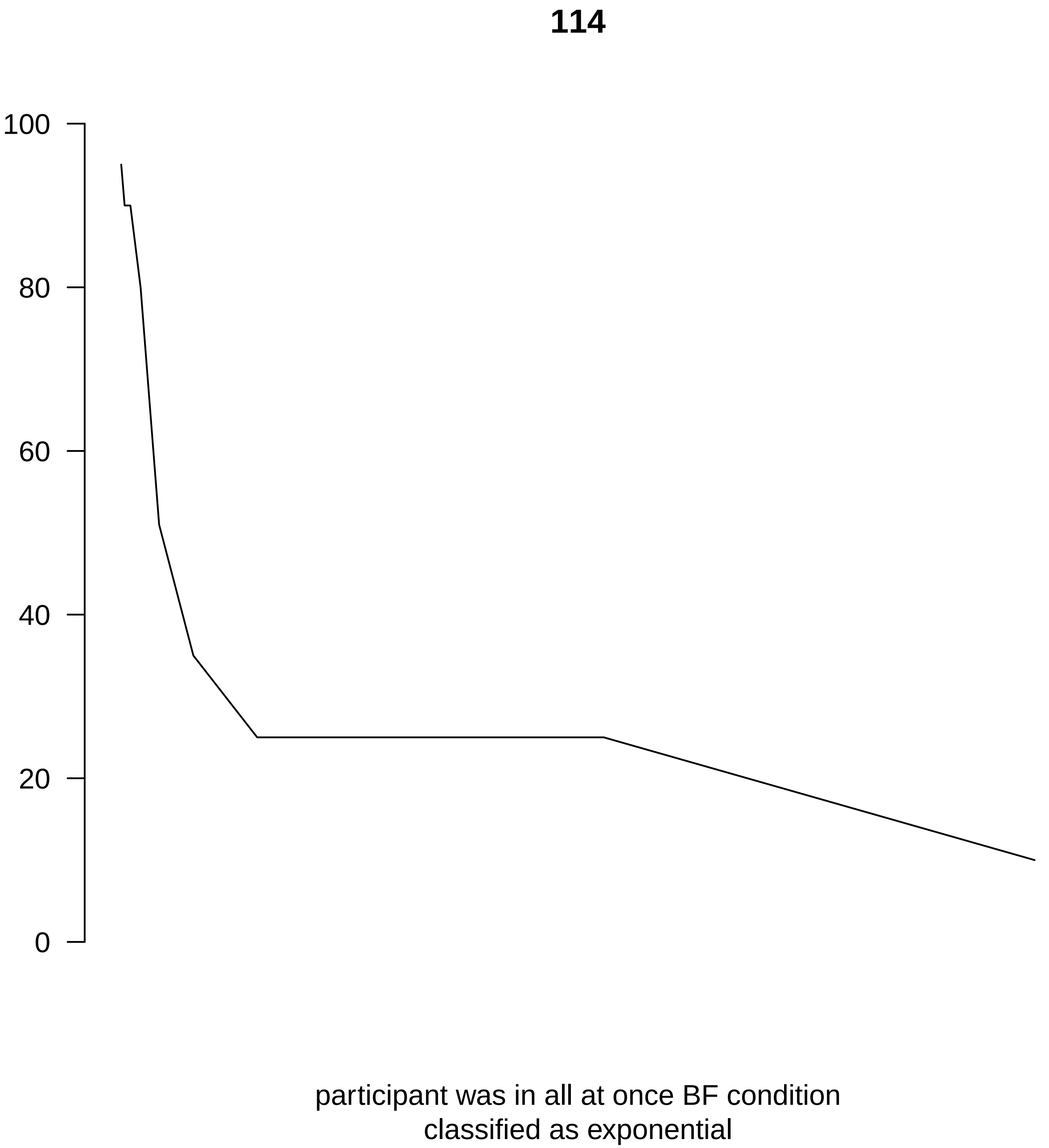
114
100
80
60
40
20
0
participant was in all at once BF condition
classified as exponential

121

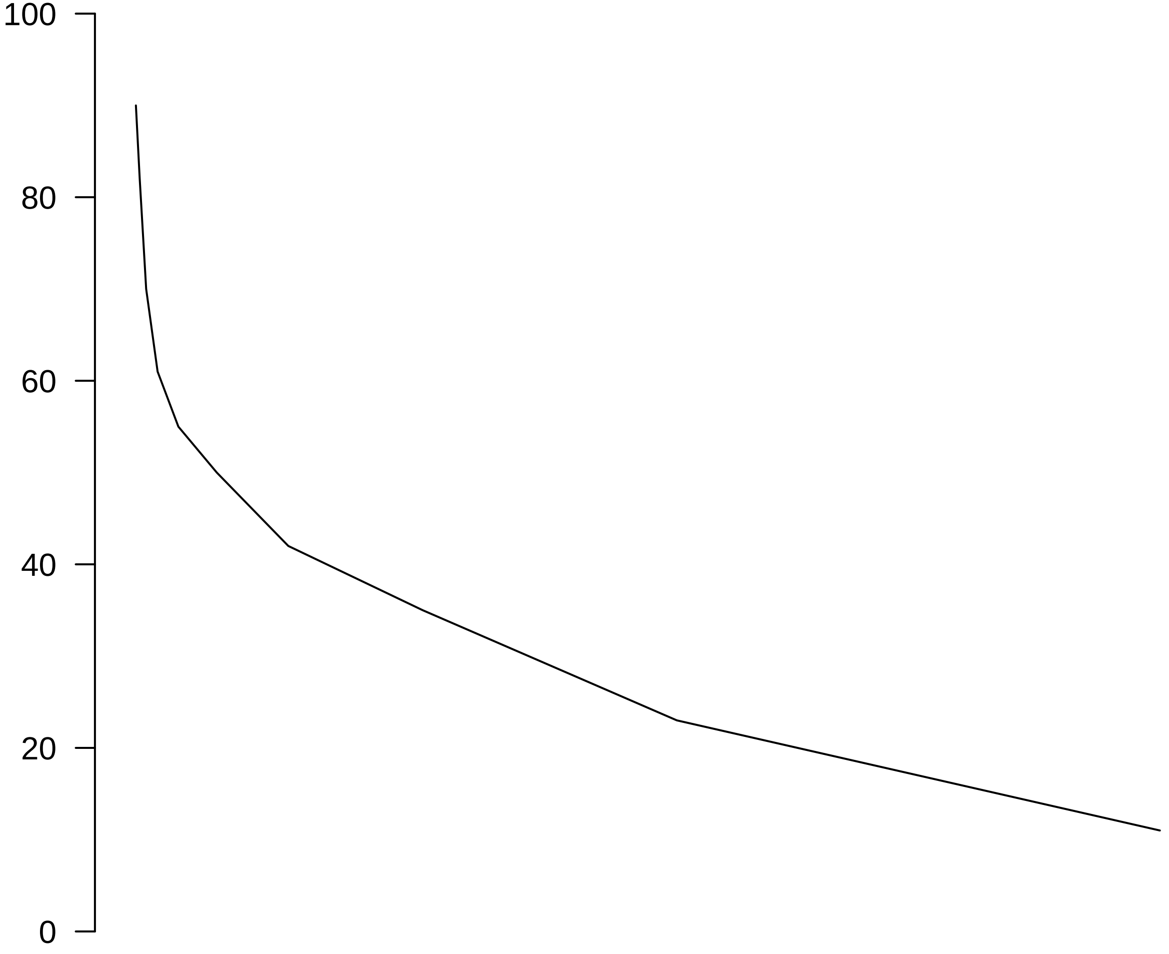

participant was in all at once BF condition
classified as exponential

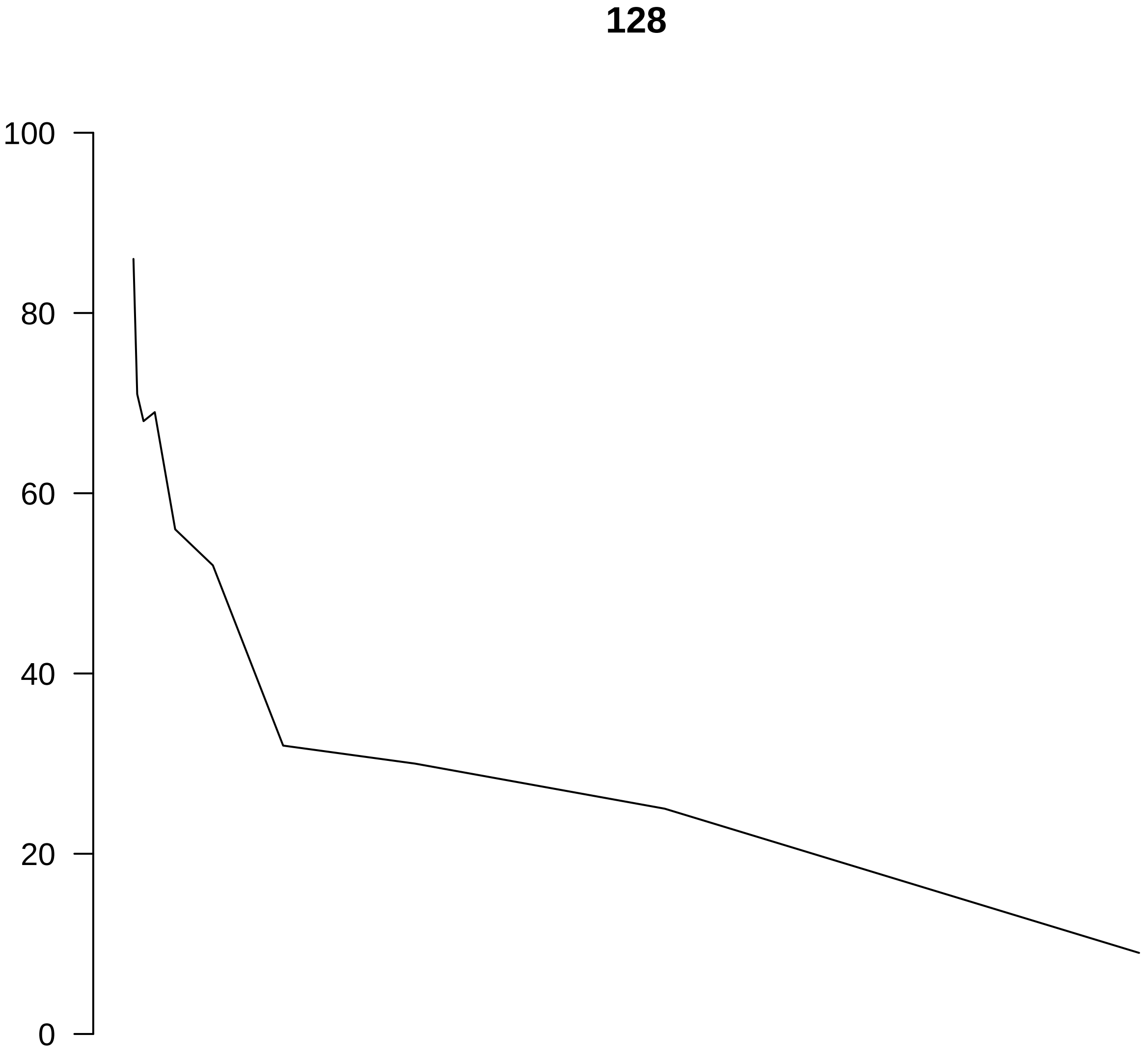

participant was in all at once BF condition
classified as exponential

131

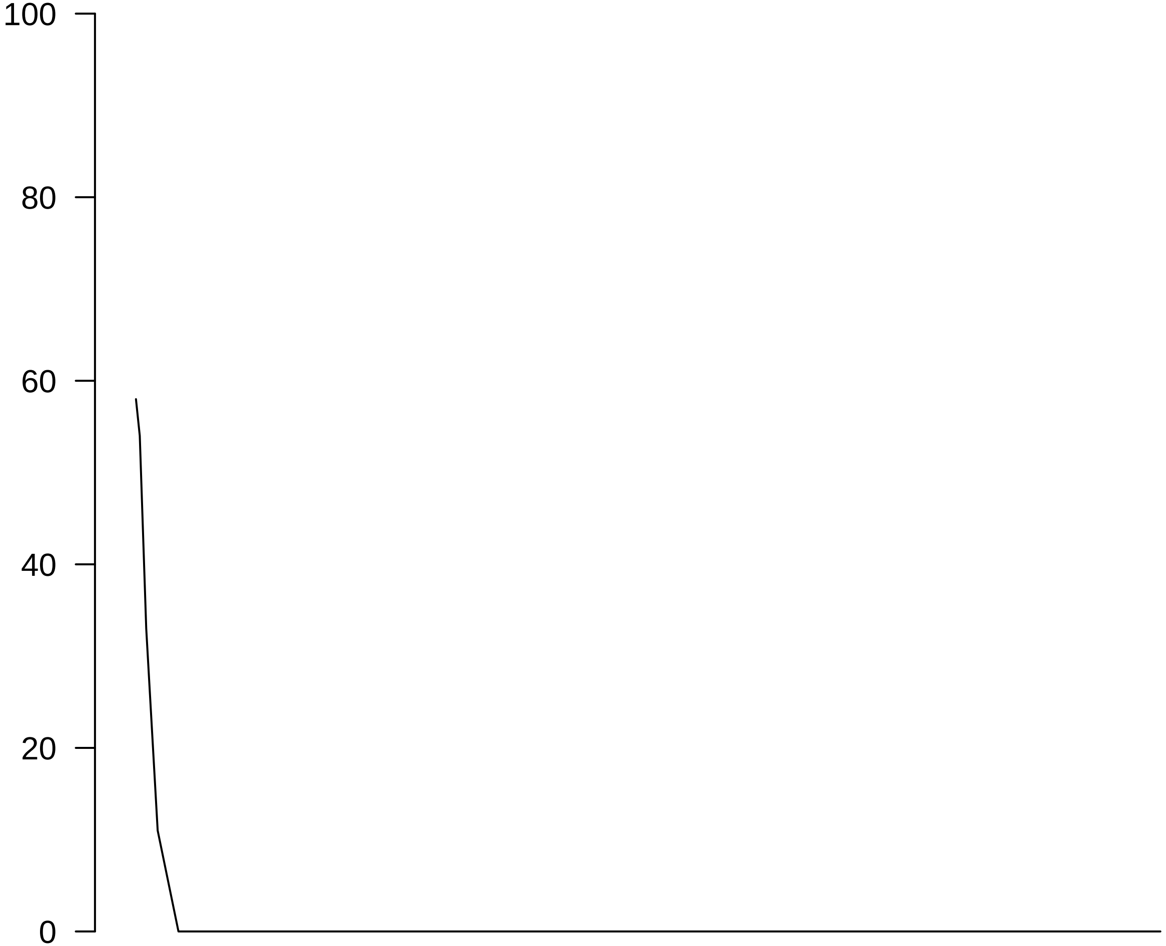

participant was in all at once BF condition
classified as exponential

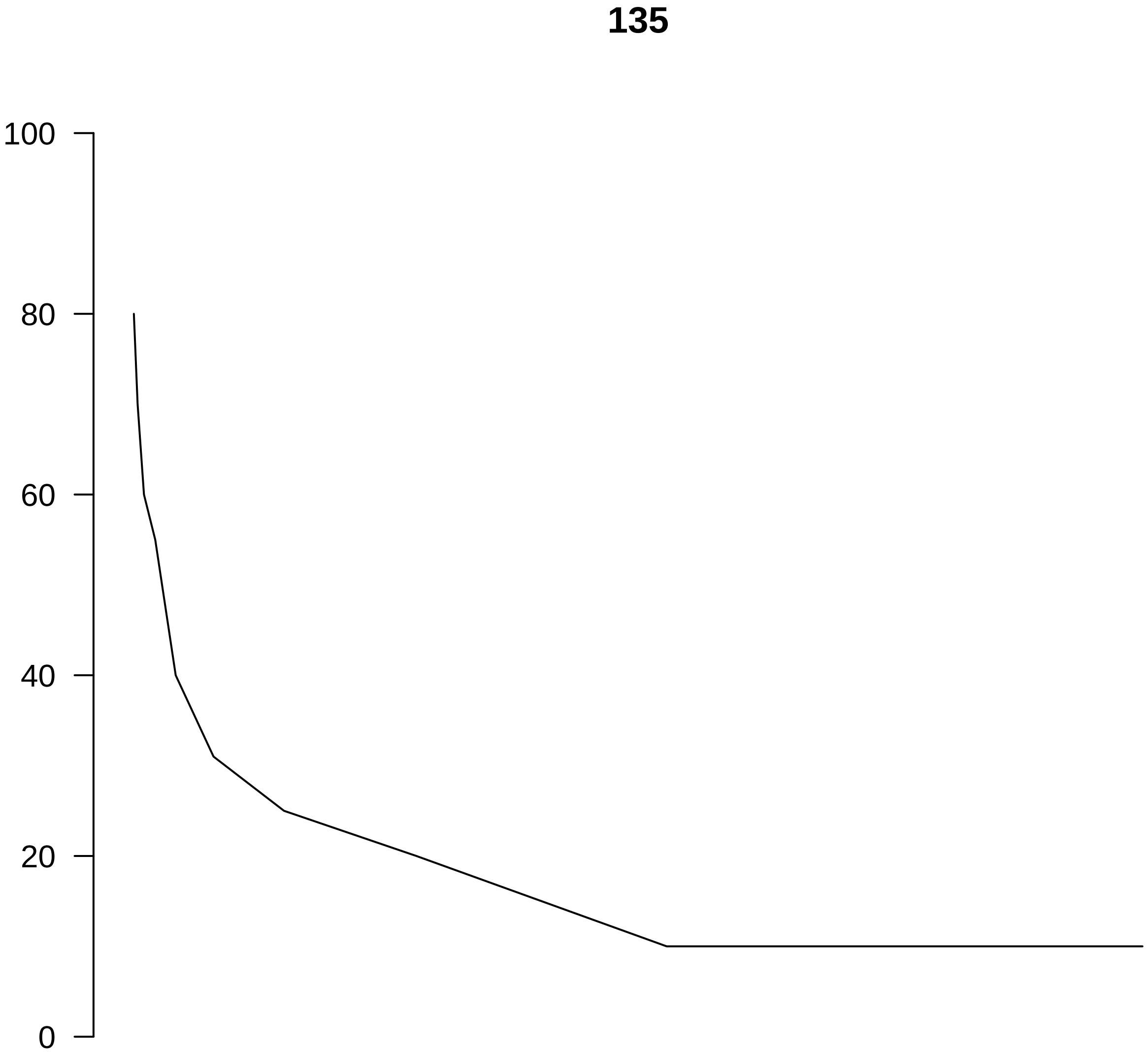

participant was in all at once BF condition
classified as exponential
