# Supplementary figures and images for: Comparing researchers’ degree of dichotomous thinking using frequentist versus Bayesian null hypothesis testing
Source: Sci Rep. 2024 May 27;14:12120. doi: 10.1038/s41598-024-62043-w (PMC11130270; doi:10.1038/s41598-024-62043-w)

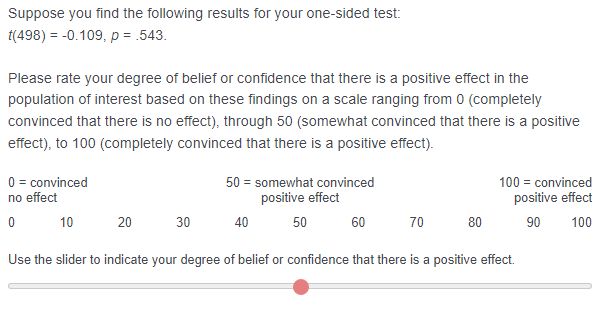


*S1*. Screenshot of part of the isolated *p*-value condition.

Supplement: Supplementary file 3 — Supplementary Information 3. [file 41598_2024_62043_MOESM3_ESM.docx]

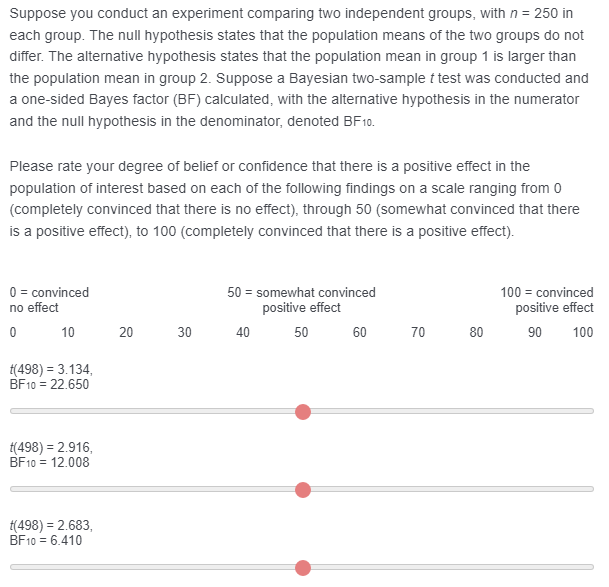


*S2*. Screenshot of part of the all at once BF condition.

Supplement: Supplementary file 4 — Supplementary Information 4. [file 41598_2024_62043_MOESM4_ESM.docx]
